# PPh_3_/Isocyanide and N_2_/Isocyanide Exchange: Pathways to Isolable Alkali Metal Keteniminyl Anions

**DOI:** 10.1002/anie.202504325

**Published:** 2025-03-22

**Authors:** Sunita Mondal, Yihao Zhang, Prakash Duari, Kai‐Stephan Feichtner, Arpan Das, Lili Zhao, Gernot Frenking, Viktoria H. Gessner

**Affiliations:** ^1^ Faculty of Chemistry and Biochemistry Ruhr‐University Bochum Universitätsstrasse 150 44801 Bochum Germany; ^2^ State Key Laboratory of Materials‐Oriented Chemical Engineering School of Chemistry and Molecular Engineering Nanjing Tech University Nanjing 211816 China; ^3^ Fachbereich Chemie Philipps‐Universität Marburg Hans‐Meerwein‐Strasse 4 35043 Marburg Germany

**Keywords:** Bonding analysis, Ligand exchange reaction, Metallated ketenimines, Synthetic strategies, Ylide chemistry

## Abstract

Keteniminyl anions hold significant promise for advancing ketenimine chemistry, yet their isolation has remained elusive until now. Drawing inspiration from recent advances in ligand exchange reactions at carbon, we report the successful synthesis of a series of isolable alkali metal keteniminyl anions through substitution of the phosphine ligand in metalated ylides or the N₂ ligand in diazomethanides with isocyanides. The exchange reactions were found to proceed more rapidly with aryl isocyanides than with the more electron‐rich alkyl‐substituted derivatives and were also more efficient when starting from the diazo compounds. The resulting keteniminyl anions exhibit bent geometries, which can be attributed to a donor–acceptor interaction with the isocyanide, giving rise to a carbone‐like structure. Electron‐withdrawing substituents on the nitrogen enhance backbonding, thus leading to larger bending angles and a more pronounced ynamide character as also evidenced by a blue‐shifted ketenimine stretch in the IR spectrum. The keteniminyl anions react efficiently with unsaturated compounds to form a diverse array of heterocycles, highlighting their potential as versatile building blocks in synthetic chemistry.

## Graphical abstract

The synthesis of isolable keteniminyl anions through substitution of the phosphine ligand in metalated ylides or the N₂ ligand in diazomethanides with isocyanides is reported. The anions feature a bent geometry, with the angle decreasing with increasing donor ability of the nitrogen substituent, and exhibit broad applicability in the formation of heterocycles via cycloaddition reactions.

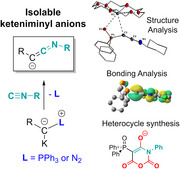

## Introduction

**Figure 1:**
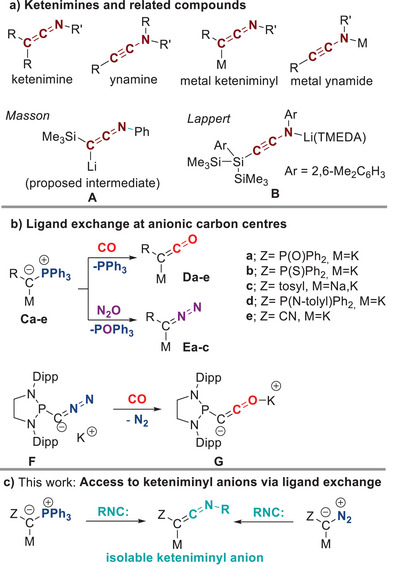
a) Ketenimines, ynamines, and their metalated derivatives;^[^ ^17^, ^25^ ^]^ b) examples of ligand exchange reaction in metalated ylides^[^ ^26^, ^27^, ^28^, ^29^, ^30^, ^31^, ^49^ ^]^ and diazomethanides;^[^ ^32^ ^]^ and c) synthesis of keteniminyl anions via PPh_3_/isocyanide and N_2_/isocyanide ligand exchange.

Since their first synthesis by Staudinger over a century ago,^[^ ^1^ ^]^ ketenimines (C═C═NR), the nitrogen analogs of ketenes, have transitioned from curiosities to key reagents in synthetic chemistry (Figure 1).^[^ ^2^, ^3^, ^4^, ^5^ ^]^ The intrinsic polarization of the C═C═N bond and the ability of ketenimines to participate in nucleophilic, electrophilic, and pericyclic reactions make them excellent reagents for synthesizing complex molecules, particularly through cycloadditions that yield diverse heterocyclic frameworks.^[^ ^3^, ^6^ ^]^ The reactivity of ketenimines can be fine‐tuned by modifying the substituents on the alkene or imine moiety, offering a high degree of synthetic flexibility. Ketenimines have been synthesized using a variety of different methods, with the Wittig reaction between phosphorus ylides and isocyanates, or between aza‐Wittig reagents and ketenes being among the most commonly employed approaches.^[^ ^7^, ^8^, ^9^ ^]^ Further methods involve the coupling of stable singlet or Fischer carbenes with isocyanides,^[^ ^10^, ^11^, ^12^ ^]^ or the rearrangement of ynamines through a 1,3‐shift of a leaving group from the nitrogen to the carbon atom.^[^ ^13^, ^14^, ^15^, ^16^ ^]^

s‐Block metal keteniminyl compounds represent promising reagents to access ketenimines via simple nucleophilic substitution reactions. However, these anions have only poorly been described in present literature. For example, Masson reported the in situ formation of the lithiated silylketenimine **A** through deprotonation of the corresponding ketenimine or elimination and subsequent deprotonation from an enaminate, but no spectroscopic data was provided.^[^ ^17^ ^]^ Keteniminyl complexes with other metals have been reported,^[^ ^18^, ^19^, ^20^, ^21^, ^22^, ^23^ ^]^ but with limited applications in synthesis. Metal ynamides represent the metallo tautomers of metal keteniminyls, in which the metal is bound to the nitrogen instead of the carbon end. Similar to keteniminyl anions, they have been proposed as intermediates in several transformations, but have been scarcely investigated as isolable reagents.^[^ ^24^ ^]^ The only example of an s‐block metal ynamide, lithium complex **B**, was prepared by Lappert through an unusual coupling of two isocyanide molecules with a silyllithium reagent.^[^ ^25^ ^]^

Recently, we reported on a new synthetic pathway to ketenes from isolable ketenyl anions **D** synthesized through the mild exchange of the phosphine ligand in metalated ylides **C** by carbon monoxide (Figure 1b).^[^ ^26^ ^]^ Using this approach, our group^[^ ^27^, ^28^, ^29^, ^30^ ^]^ and others^[^ ^31^ ^]^ have successfully isolated a series of phosphinoyl‐, tosyl‐, and cyano‐substituted ketenyl anions, which proved to be excellent precursors to ketenes and a large range of carbonyl‐containing compounds. Shortly after, Liu and coworkers reported the formation of the stable (phosphino)ketenyl anion **G** via an N_2_/CO exchange in diazomethanides **E** (Figure 1b),^[^ ^32^ ^]^ a strategy previously used in situ in ketene chemistry.^[^ ^33^ ^]^ Besides metalated ylides and diazomethanides, neutral diazoalkenes have also been reported by Hansmann and coworkers to undergo an exchange of the N_2_ moiety when treated with carbon monoxide or 2,6‐dimethylphenyl isocyanide.^[^ ^34^, ^35^, ^36^ ^]^ These exchange reactions are reminiscent of ligand exchange reactions known in transition metal chemistry for decades. While many low valent main group compounds like borylenes,^[^ ^37^, ^38^ ^]^ aluminylenes,^[^ ^39^ ^]^ or phosphinidenes^[^ ^40^, ^41^ ^]^ have also been reported to undergo similar ligand exchange reactions, this concept is still rarely applied to carbon compounds.

It is important to note that these reactions are conceptually distinct from typical nucleophilic substitution reactions. In this case, the strongly nucleophilic carbon attacks an ambiphilic reagent, where both its σ‐donor and π‐acceptor properties play a crucial role in enabling the reaction to proceed. These reactions align well with experimental^[^ ^21^ ^]^ and computational studies^[^ ^42^ ^]^ on the bonding in ylidic compounds, which led to a rethinking of carbon chemistry^[^ ^43^ ^]^ and the description of the phosphorus carbon bond as a dative interaction (C←:L) similar to the bonding interactions in transition metal phosphine complexes. This description of carbon compounds as coordination complexes was soon after applied to other carbon compounds with *N‐*heterocyclic carbenes (NHC) or carbon monoxide as ligands.^[^ ^44^, ^45^, ^46^, ^47^ ^]^

The N_2_/CO and PPh_3_/CO exchange reactions to ketenyl anions suggest that ligand exchange at carbon represents a broader strategy for generating metalated reagents. However, unlike CO, a straightforward PPh_3_/N_2_ exchange with dinitrogen was unsuccessful. This limitation could be overcome using nitrous oxide, which facilitated the reaction through phosphine oxide elimination.^[^ ^48^, ^49^ ^]^ These findings prompted us to explore the scope of phosphine exchange in ylidic compounds with other neutral ligands. Herein, we present the mild PPh_3_/isocyanide and N_2_/isocyanide exchange as a novel approach to access isolable alkali metal keteniminyl anions.^[^ ^50^ ^]^

## Results and Discussion

### Synthesis and Isolation of Alkali Metal Salts of Keteniminyl Anions

**Scheme 1:**
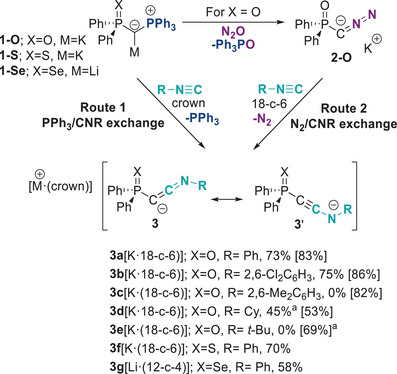
Synthetic routes to keteniminyl anions **3**. Synthesis of potassium keteniminyl anions via PPh_3_/isocyanide exchange in metalated ylides (route 1) and via N_2_/isocyanide exchange in diazomethanides formed from metalated ylides and nitrous oxide (route 2). Yields in brackets refer to route 2, other yields to route 1. **^a^** NMR yields; all other are yields are isolated yields.

Like CO, isocyanides (:CNR) are ambiphilic ligands with σ‐donor and π‐acceptor properties, which have been widely used in transition metal chemistry.^[^ ^51^ ^]^ We began our investigations with alkyl isocyanides and metalated ylide **1‐O** as model substrates. Unfortunately, treatment of in situ generated **1‐O** with *tert*‐butyl isocyanide resulted in no reaction to the targeted keteniminyl anion **3e**, even when the reaction was conducted at elevated temperatures (Scheme 1). However, when using cyclohexyl isocyanide, selective release of PPh_3_ (*δ*_P_ = −5.42 ppm) was observed as confirmed by ^31^P{^1^H} NMR spectroscopy, indicating a successful PPh_3_/:CNR exchange. Nonetheless, the reaction was extremely sluggish, achieving only 45% conversion after heating the reaction mixture at 80 °C for 1 week.

We hypothesized that electron‐poor isocyanides might enhance the ligand exchange due to their increased acceptor properties which could facilitate the attack of the nucleophilic metalated ylide. Therefore, we shifted our focus to aryl‐substituted isocyanides. To our delight, the addition of phenyl isocyanide to a toluene solution of **1‐O** at room temperature led to an immediate color change from orange to dark brown. Monitoring of the reaction by ^31^P{^1^H} NMR spectroscopy confirmed the liberation of PPh_3_ and the formation of the targeted keteniminyl anion **3a** as characterized by a signal at *δ*_P_ = 11.0 ppm, along with re‐formation of small amount of parent ylide **1‐O^H^** (Ph_2_P(O)CHPPh_3_). However, isolation of the keteniminyl salt **3a–K** from the reaction mixture proved challenging due to similar solubility of **3a–K** and **1‐O^H^**. To address this challenge, we repeated the reaction in the presence of 18‐crown‐6 (18‐c‐6) for complexation of the potassium cation. The 18‐c‐6 complex of keteniminyl anion **3a** turned out to be less soluble and precipitated from the reaction mixture, thus enabling its isolation as a brown solid in a high yield of 73%. The structure of **3a** could unambiguously be characterized by different spectroscopic and crystallographic methods (Table 1 and 2).

To establish the generality of this method, yldiides **1‐S** and **1‐Se** were likewise treated with phenyl isocyanide in the presence of crown ether to obtain **3f**[K·(18‐c‐6)] and **3g**[Li·(12‐c‐4)] in good yields of 70% and 58%, respectively. We also investigated the reaction of other aryl isocyanides with yldiide **1‐O**. Treatment of **1‐O** with 2,6‐dichlorophenyl isocyanide in the presence of 18‐c‐6 successfully generated the corresponding potassium complex of keteniminyl anion **3b** as brown solid in 75% isolated yield. However, attempts to obtain the 2,6‐dimethylphenyl substituted keteniminyl anion using this method were unsuccessful. Addition of Xyl‐NC (Xyl = 2,6‐dimethylphenyl isocyanide) to **1‐O** led to the protonation of the yldiide and re‐formation of the parent ylide. This observation can be explained by the high basicity of yldiide **1‐O**, which easily abstracts a proton from the ortho methyl group of the isocyanide.

To address the challenges observed in the PPh_3_/isocyanide exchange, we pursued an alternative synthetic strategy. We hypothesized that the use of less basic diazomethanide **2‐O** could mitigate undesired deprotonation, while providing an additional driving force for the exchange through the release of dinitrogen. Leveraging our recently developed method for synthesizing diazomethanides, we prepared diazo compound **2‐O** from the metalated ylide **1‐O** and N_2_O (Scheme 1).^[^ ^49^ ^]^ Addition of phenyl, xylyl, or 2,6‐dichlorophenyl isocyanide to a THF solution of **2‐O** in the presence of 18‐c‐6 led to the clean formation of the keteniminyl anions via N_2_/:CNR exchange. To our delight, the 18‐c‐6 complexes of **3a**, **3b**, and **3c** could be isolated in high yields of more than 80% through this protocol. We also tested the reaction of **2‐O** with alkyl isocyanides. Compared to aryl isocyanides, the reaction of alkyl isocyanides with **2‐O** is significantly slower, requiring several days of stirring at 45 °C to reach completion (4 days for cyclohexyl isocyanide and 14 days for *tert*‐butyl isocyanide). Nonetheless, the 18‐c‐6 complexes of **3d** could be isolated as crystalline solids in 53% yield, thus proving the versatility of the developed protocol. Compound **3e** was also formed in high yields but could not be cleanly isolated due to challenges in separating it from excess 18‐crown‐6. All keteniminyl anions are stable under an inert atmosphere at room temperature and show no tendency for decomposition or dimerization (e.g., to form 2‐iminoazetidines)^[^ ^52^, ^53^ ^]^ even after storage for weeks. Stability tests at elevated temperatures revealed that compound **3a**[K·(18‐c‐6)] remains stable even when kept in refluxing C_6_D_6_ for 24 h.

### Spectroscopic and Crystallographic Studies

With a series of keteniminyl anions in hand, we proceeded to investigate their structural and spectroscopic properties. Our primary focus was to determine whether these species are more accurately described as keteniminyl anions (**3**) or as ynamides (**3′**), with the metal preferentially binding to the nitrogen end (Scheme 1). In THF solution, the keteniminyl anions **3** exhibit signals at approx. 40 ppm in the ^13^C{^1^H} NMR spectrum corresponding to the *C*CN carbon atom (Table 1). This signal is significantly upfield shifted compared to neutral ketenimines (e.g., 76.2 ppm for (*i*Pr)_2_C═C═N*i*Pr)^[^ ^54^ ^]^ and ynamines (e.g., 73.0 ppm for Ph─C≡C─N(*i*Pr)CO_2_CH_2_CHCH_2_),^[^ ^55^ ^]^ arguing for the presence of a lone pair at this carbon atom. Within the series of phosphoryl keteniminyl anions **3a–3e**, this signal exhibits only minor shifts, with a tendency toward higher fields as the donor ability of the nitrogen substituents increases. The ^1^ *J*_P,C_ coupling constant varies more significantly and decreases from 218.9 to 133.8 Hz in the order of **3b** > **3a** > **3c** > **3d** ≈ **3e**. This suggests a reduced s‐character with increasing electron donation from the R group, thereby favoring the keteniminyl structure **3** (sp^2^ hybridization) over the ynamide structure **3′** (sp hybridization). This interpretation is further supported by infrared spectroscopy, which revealed characteristic bands for the C═C═N stretching vibration between 1968 and 2054 cm^−1^. These values are in‐between the regions reported for ketenimines (around 2000 cm^−1^)^[^ ^54^, ^56^ ^]^ and ynamines (around 2100 cm^−1^),^[^ ^57^ ^]^ and increase with the electron‐withdrawing ability of the N‐substituent. Thus, compound **3b**, with the 2,6‐dichlorophenyl substituent, exhibits the largest value, indicating the highest ynamide character.

**Table 1:** Comparison of the NMR and IR spectroscopic data of the keteniminyl anions 3 and the related ketenyl anion and diazomethanide 4 and 5.

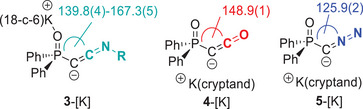				
	δ (P)	δ (C1)	^1^ *J*_PC_	IR
**3a**‐[K]	7.0	45.6	197.3	2046.5
**3b**‐[K]	5.7	44.5	218.9	2054.4
**3c**‐[K]	8.9	38.9	185.8	2037.2
**3d**‐[K]	15.3	40.2	133.8	1981.2
**3e**‐[K]	13.2	42.6	133.8	1968.3
**3f**‐[K]	19.6	44.8	92.9	2000.4
**3g**‐[Li]	3.88	46.2	162.0	2074.9
**4**‐K^a)^	13.2	3.1	209.6	2091.8
**5**‐[K]^b)^	17.2	19.8	57.6	1970.0

NMR shifts are given in ppm, coupling constants in Hz, and IR stretching frequencies in cm^−1^.

^a)^ NMR and IR data taken from Refs. [27].

^b)^ NMR and IR data taken from Ref. [49]. [Li] refers to the [Li·(12‐crown‐4)] cation, [K] = [K·(18‐c‐6)] except for **5** where [K] refers to K. [2,2,2]‐cryptand. For **4‐**K, the NMR and IR data were collected without additives.

Interestingly, the lithium salt of the phosphine selenide **3g** features an even higher C═C═N stretch at 2075 cm^−1^, suggesting a further shift toward resonance form **3′**. Due to the superior anion‐stabilizing ability of the P═Se moiety compared to P═O, the opposite effect was expected.^[^ ^27^ ^]^ However, this discrepancy was clarified through X‐ray diffraction (XRD) analysis of single crystals of **3g**, which revealed the coordination of the lithium cation to the nitrogen atom of the keteniminyl moiety (see Supporting Information for details). This contrasts with the potassium compounds, which exhibit no interaction between the metal cation and the keteniminyl unit (vide infra). The distinct coordination chemistry of alkali metal cations has recently been shown to also influence the structure of ketenyl anions, with lithium shifting the electronic structure toward the resonance form with a formal C≡C triple bond, analogous to our observations here.^[^ ^28^ ^]^

A comparison of the NMR and IR spectroscopic data of the keteniminyl anions with those of the corresponding ketenyl anion **4** and diazomethanide **5** reveals that the ^13^C{^1^H} NMR shift of the C1 carbon atom in **3** is deshielded relative to the other anions, indicating a reduced charge accumulation at this position. In contrast, the value for C═C═NR vibration falls in between the stretching frequency for the ketenyl and diazomethanide.

**Figure 2:**
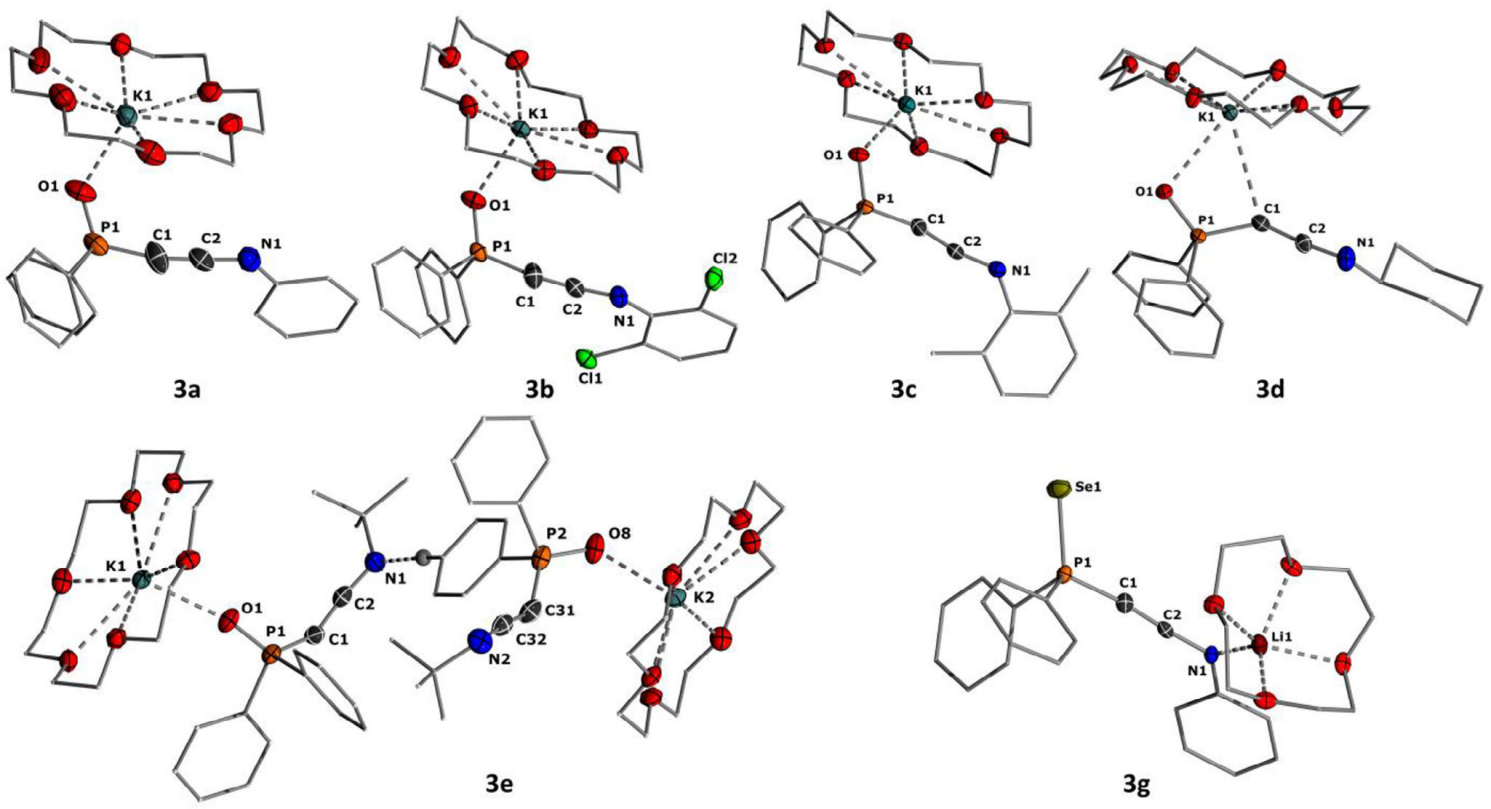
Molecular structures of potassium keteniminyl anions **3** as their potassium crown‐ether complexes in the solid state. Hydrogen atoms (except H40 in **3e**) and disordered parts in the molecules are omitted for clarity. Thermal ellipsoids are shown at the 50% probability level. Important bond lengths and angles are given in Table 2, and crystallographic details are given in Supporting Information.

Single crystals of the crown ether complexes of the keteniminyl anions were obtained by vapor diffusion of pentane (**3a**, **3b**, and **3c**) or hexane (**3d**) into concentrated THF solutions at room temperature (Figure 2). In the case of **3e**[K·(18‐c‐6)], single crystals were grown by slow evaporation of a saturated pentane solution.^[^ ^58^ ^]^ XRD analyses revealed that all compounds form monomeric structures, except for **3e**[(K·(18‐c‐6)], which features two molecules in the asymmetric unit, which are weakly bound to each other through intermolecular hydrogen bonding between the keteniminyl nitrogen and a *para*‐CH moiety.

In the aryl‐substituted keteniminyl anions **3a–3c** as well as in **3e**, the potassium cation is coordinated exclusively by the phosphinoyl group, whereas it is bound by both the phosphinoyl moiety and the C1 carbon atom in **3d**[(K·18‐c‐6)], reflecting the more pronounced keteniminyl character (**3**) in the alkyl‐substituted systems. Indeed, the K‐C distances in the monomeric complexes follow the same trend as observed by the NMR and IR spectroscopic studies. DOSY NMR spectroscopic studies of **3a**[(K·(18‐c‐6)] in C_6_D_6_ suggest that the contact ion pair is maintained in solution, enabling a direct comparison of the solid state and solution state properties (Figure S7). As such, K–C distances decrease from 4.218 Å in the electron‐poor dichloroaryl system **3b** to 3.048 Å in the cyclohexyl‐substituted keteniminyl **3d**. These pronounced differences in bond lengths clearly support the change in the bonding situation, with complex **3d** being best described as a keteniminyl anion, where the potassium cation is coordinated through the carbanionic center C1. In contrast, the structure of **3b** argues for a pronounced ynamide character with no bonding interaction between the cation and C1. The structure parameters further reflect this trend in the bonding situation (Table 2). Accordingly, the C─C bonds shorten in the order **3e** ≈ **3d** > **3c** > **3a** ≈ **3b**, whereas the C─N bonds lengthen in the same sequence. In addition, keteniminyl anions **3d** and **3e** with the alkyl substituent at nitrogen exhibit more acute P─C─C angles (**3d**: 139.3(4)°, **3e**: 132.2(1)°, and 137.2(2)°) compared to the aryl substituted analogs (**3a**: 167.3(4)°, **3b**: 166.8(2)°, and **3c** (159.5(1)°). Furthermore, selenide **3g** featured the largest P─C─C angle and the shortest C─C bond due to the additional bonding of lithium to the nitrogen of the keteniminyl anion, thus favoring resonance form **3′**.

**Table 2:** Comparison of the NMR and IR spectroscopic data of the keteniminyl anions 3 and the related ketenyl anion and diazomethanide 4 and 5.

	3a [K·(18‐c‐6)]	3b [K·(18‐c‐6)]	3c [K·(18‐c‐6)]	3d [K·(18‐c‐6)]	3e [K·(18‐c‐6)]	3g [Li·(12‐c‐4)]	4 [K·([2,2,2]crypt)]^a)^	5 [K·([2,2,2]crypt)]^b)^
P–O/Se [Å]	1.490(3)	1.490(2)	1.498(1)	1.497(3)	1.498(1), 1.499(1)	2.117(1)	1.496(1)	1.492(2)
P–C1 [Å]	1.694(5)	1.701(2)	1.693(2)	1.696(5)	1.699(2), 1.700(2)	1.710(5)	1.686(2)	1.710(3)
C1–C2 [Å]	1.221(6)	1.228(3)	1.239(2)	1.283(6)	1.271(2), 1.266(2)	1.212(8)	1.240(2)	–
C2–N1 [Å]	1.288(5)	1.281(3)	1.271(2)	1.246(6)	1.253(2), 1.259(2)	1.299(7)	–	–
K···C1 [Å]	4.068(5)	4.218(3)	3.722(2)	3.048(4)	5.162(2), 3.899(2)	–	–	–
P–C1–E1 (E1 = C or N) [°]	167.3(4)	166.8(2)	159.5(1)	139.3(4)	132.2(1), 137.2(1)	170.8(5)	148.9(1)	125.9(2)
C1–E1–E2 (E1 = C or N) (E2 = N or O) [°]	173.5(4)	168.0(2)	169.9(2)	172.6(5)	172.9(2), 172.7(2)	175.4(5)	175.1(2)	170.3(2)

^a)^ Structure parameters are taken from Refs. [27, 28].

^b)^ Structure parameters are taken from Ref. [49].

The comparison of the keteniminyl anions **3** with the ketenyl anion **4** and diazomethanide **5** reveals distinct differences in their P–C–C angles. The angle in ketenyl anion **4** is smaller than those of the aryl‐substituted keteniminyl anions **3a–c** but larger than those in the alkyl‐substituted systems **3d** and **3e** (Table 1). In contrast, the corresponding angle in the diazomethanide **5** amounts to 125.9(2), making it the most acute angle within the isoelectronic series of compounds **3–5**. In case of neutral “carbones,” the degree of bending has been attributed to the acceptor properties of the ligands, with stronger accepting capabilities resulting in larger angles.^[^ ^59^ ^]^ For instance, the dicarbonyl complex C(CO)_2_ (carbon suboxide C_3_O_2_) has a larger bending angle of 156° than the dinitrogen complex C(N_2_)_2_ (122°), because CO is a better π‐acceptor than N_2_.^[^ ^59^ ^]^ The same trend is observed for the anions [C(CN)(CO)]^−^ (166°) and [C(CN)(N_2_)]^−^ (134°)^[^ ^60^ ^]^ and for compounds **4** and **5**. Hence, the structures **3–5** reflect the decreasing accepting property of the ligands in the series::CNAr > CO > :CNAlk > N_2_. This aligns with the viability of the exchange reactions, which follows the relative stability order: [R–C–N_2_]^−^ < [R–C–PPh_3_]^−^ ≈ [RC–CNR_alkyl_]^−^ < [R–C–CO]^−^ < [RC–CNR_aryl_]^−^. The stronger the acceptor capacity of L, the higher the stability of the anion due to increased charge delocalization. This is in contrast to findings by Bertrand and coworkers on phosphinidenes, where stronger donor ligands formed more stable complexes.^[^ ^41^ ^]^

### Quantum Chemical Analysis of the Bonding in Keteniminyl Anions and Related Carbon Complexes

**Figure 3:**
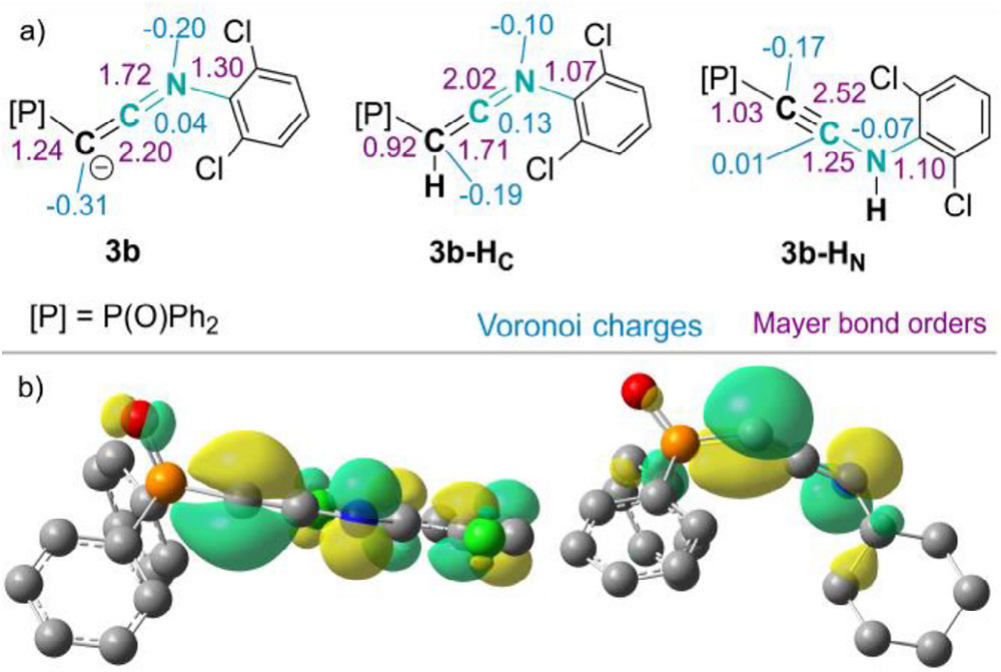
Bonding situation in the keteniminyl anions. a) Comparison of the Mayer bond orders (pink) and Voronoi charges (blue) in **3b** and its protonated derivatives **3‐H_C_** and **3‐H_N_**. b) Display of the contour plots of the HOMO of **3b** and **3d**.

To shed light on the bonding situation in the keteniminyl anions, we carried out DFT calculations at the BP86 + D3(BJ)/def2‐TZVPP level of theory. We analyzed the naked anions [Ph_2_OPC‐L2]^−^ with L2 = CNAr (**3b**) as an example of an electron‐poor and L2 = CNCy (**3d**) for an electron‐rich keteniminyl anion and compared their structures to the protonated congeners, with the proton being bound either to the C1 carbon atom (ketenimine, **3‐H_C_**) or the nitrogen atom (ynamine **3‐H_N_**). The calculated Mayer bond orders (MBO) of the newly formed C─C and C─N bonds confirm the multiple bond character of both bonds in **3b** and **3d** and suggest an intermediate bonding situation in the keteniminyl anions (Figure 3 and Table 3). These bond orders fall between those calculated for the protonated congeners, the ketenimine and ynamine. For instance, the C─C bond order for **3b** amounts to 2.20, while the double bond in ketenimine **3b‐H_C_** has a value of 1.71 and the triple bond in the ynamine **3b‐H_N_** a value of 2.52. The C─C bond order is higher in the phenyl derivative **3b** (2.20) than in the cyclohexyl system **3d** (1.85), which correlates with the increased ynamide character **3′** of electron‐poor keteniminyl anions as indicated by the spectroscopic and crystallographic studies. This trend is further evidenced by the significant reduction in the calculated bending angle at C1 (P1–C1–C2) from **3b** to **3d** as well as the shape of the highest occupied molecular orbital (HOMO), which is delocalized across the C–C–N linkage and the entire aryl substituent in **3b**, while it is mostly localized at the C1 carbon and nitrogen atom in **3d**. Accordingly, the charges at the C1 (*q*(C1) = −0.38) and nitrogen atom (*q*(N) = −0.27) in **3d** are more negative than the corresponding values (*q*(C1) = −0.31; *q*(N) = −0.20) in **3b**.

**Table 3:** Calculated Voronoi partial charges (*q*) and Mayer bond orders (MBO) of structures of the anions [C(POPh_2_)(L2)]^−^ **3b** (L2 = CNAr), **3d** (L2 = CNCy), **4** (L2 = CO), and **5** (L2 = N_2_) at the BP86 + D3(BJ)/def2‐TZVPP level.

Compound	Charges *q*		MBO	
**3b**	C1	−0.31	C1‐ POPh_2_	1.24
	POPh_2_	−0.27	C1–L2	2.20
	CNAr	−0.42		
**3d**	C1	−0.38	C1–POPh_2_	1.21
	POPh_2_	−0.36	C1–L2	1.85
	CNCy	−0.26		
**4**	C1	−0.39	C1–POPh_2_	1.26
	POPh_2_	−0.38	C1–L2	1.99
	CO	−0.23		
**5**	C1	−0.38	C1–POPh_2_	1.18
	POPh_2_	−0.40	C1–L2	1.62
	N_2_	−0.22		

The comparison of the structure parameters of **3** with the ketenyl (L2 = CO (**4**)) and diazomethanide anion (L2 = N_2_ (**5**)) confirms the trends in the bending angles observed in experiment (see Supporting Information). However, the bending potential at C1 toward a linear coordination is shallow, requiring less than 1 kcal for **3b** and **4** and 1.4 as well as 5.5 kcal/mol **3d** and **5**, respectively. Despite this, the significant deviation from the linear geometry at C1, which minimizes steric repulsion between ligands, supports the structural interpretation of these species as anionic carbones [(POPh_2_) → C ← L2]^−^. The calculated partial charges for the ligands P(O)Ph_2_ and L2 and for the C1 atom show that the negative charge of the anions is distributed across the three moieties (Table 3). The CNAr ligand of **3b** has a significantly higher negative partial charge than the other L2 ligands in **3d**, **4**, and **5**. It is surprising that N_2_ in **4** has nearly the same negative charge (−0.22 e) as CO in **5** (−0.23 e), despite CO being much more strongly bonded than N₂.

Further information about the chemical bonds is obtained from the energy decomposition analysis–natural orbital for chemical valence (EDA–NOCV) analysis of the anions. The intrinsic interaction energies Δ*E*_int_ between the frozen fragments [(POPh_2_)C]^−^ + L2 follow the order **3b** > **3d** > **4** >> **5** (Table S9), which is slightly different than the BDE values where **4** has a slightly higher D_e_ value than **3d**. It indicates that the:CNCy ligand of **3d** has a larger relaxation energy than CO in compound **4**, which appears reasonable in view of the different sizes of the ligands. The relevance of the relaxation energy of the fragments for the BDE values has been stressed by Bickelhaupt, who coined the term “activation stress” for the fragment relaxation.^[^ ^61^ ^]^

The breakdown of ΔE_int_ into the attractive components (∆*E*_orb_, ∆*E*_elstat_, and ∆*E*_disp_) and the Pauli repulsion ∆*E*_Pauli_ reveals the surprising result that the sum of the attractive forces in **5** between [(POPh_2_)C]^−^ and N_2_ are higher than in **3b** (L2 = CNAr) and **4** (L2 = CO). The significantly lower total attraction, however, results from the much higher Pauli repulsion in **5** than in the other molecules. It has been shown before that the Pauli repulsion between electrons with the same spin is a very important but often neglected energy term in chemical bonding. The bond lengths of chemical bonds are not determined by the maximum overlap of the bonding orbitals, which occurs at much shorter distances than the equilibrium length. Instead, they result from the strong influence of the Pauli repulsion on the electronic structure.^[^ ^62^ ^]^

**Figure 4:**
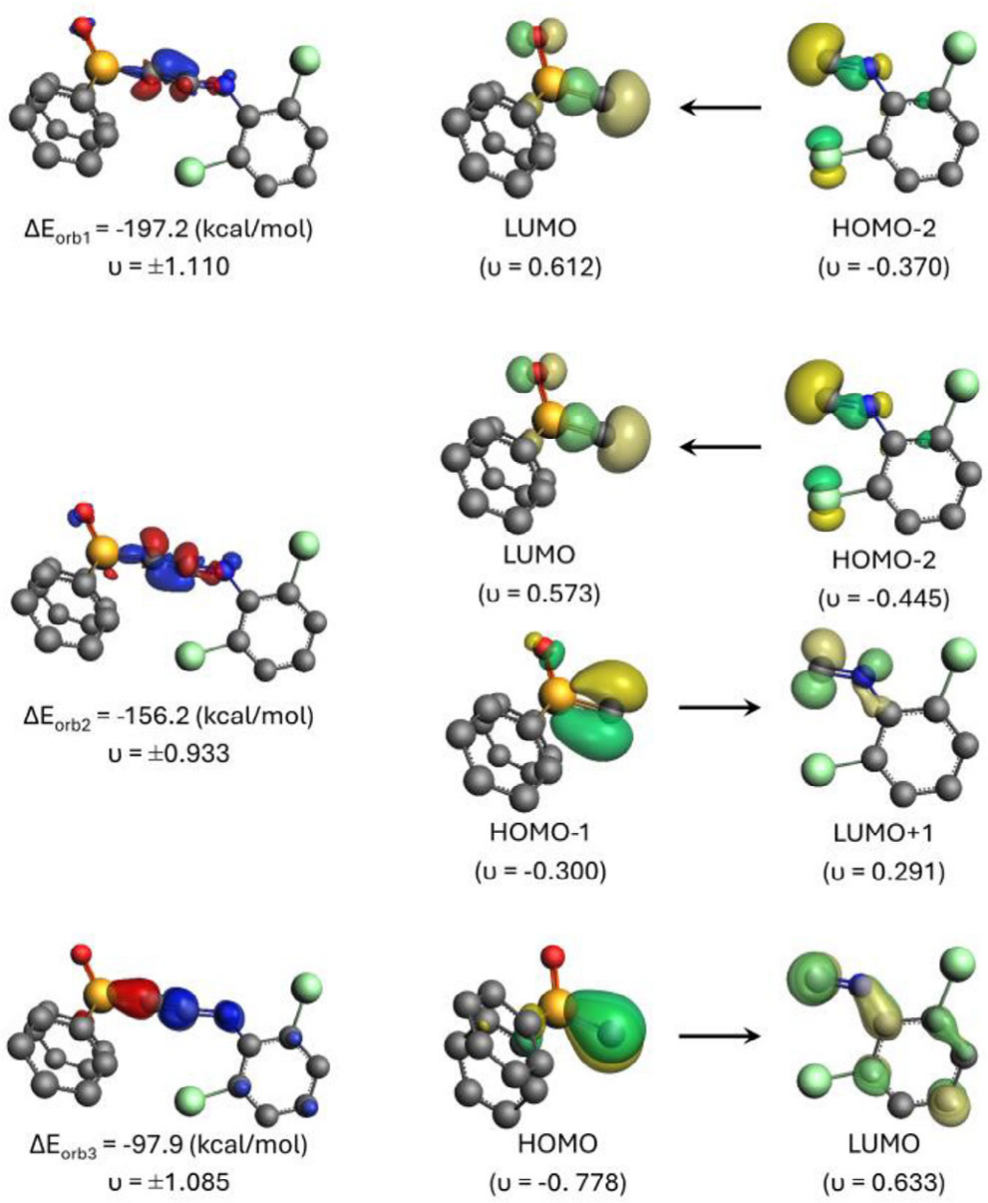
Contour plots of the deformation densities Δ*ρ* and the associated fragment orbitals of the orbital interactions ∆*E*_orb1_ – ∆*E*_orb3_ in **3b**. The eigenvalues *ν* indicate the amount of electronic charge flow.

The primary contribution to the bonding interactions between [(POPh_2_)C]^−^ and L2 arises from the covalent (orbital) term ∆*E*_orb_, which accounts for 65%–70% of the total attraction. There are three orbital pair interactions ∆*E*_orb1–3_, which account for over 90% of ∆*E*_orb_. The orbitals involved in these pairwise interactions can be identified through the shape of the deformation densities Δ*ρ*_1–3_ and the corresponding fragment orbitals associated with the orbital interactions ∆*E*_orb1–3_. These interactions in **3b** are depicted in Figure 4 (see Supporting Information for the other anions). The orbital interactions are dominated by the [(POPh_2_)C]^−^ ← L2 σ donation and the in‐plane and out‐of‐plane [(POPh_2_)C]^−^ → L2 π backdonation, in accordance with the classical Dewar–Chatt–Duncanson concept.^[^ ^63^, ^64^ ^]^ Note that the division of the orbital interactions into σ and π orbitals comes from the symmetry of the fragments, which is lost in the final molecules. This leads to a mixture of [(POPh_2_)C]^−^ ← L2 σ donation and the in‐plane [(POPh_2_)C]^−^ → L2 π backdonation in the compounds, which have approximately C_s_ symmetry.

### Reactivity Studies of Keteniminyl Anion

**Scheme 2:**
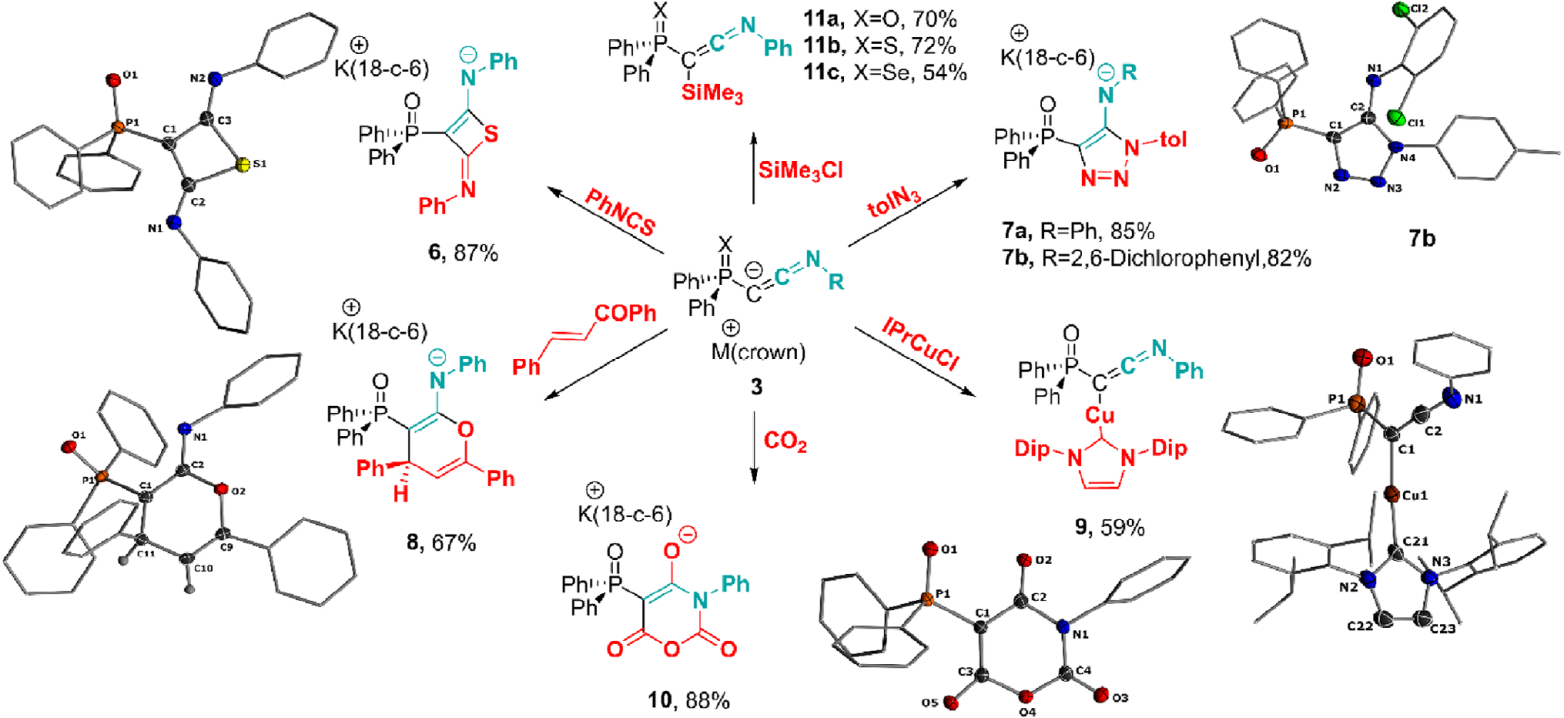
Reactivity studies of keteniminyl anions **3**. For details on reaction conditions, see the SI. For clarity, only the anionic components of the salts **6**, **7b**, **8**, and **10** are depicted. The cations [K·(18‐c‐6], solvents, and disordered parts in the solid‐state structures have been omitted for clarity. Thermal ellipsoids are shown at the 50% level.

Overall, the mild exchange of PPh_3_ or N_2_ by isocyanides represents a new method for accessing keteniminyl anions. Metallaketenimines have received surprisingly little attention in the past, with only a handful of isolated keteniminyl metal complexes reported, all involving transition metals. To the best of our knowledge, compounds **3a–3g** represent the first isolated s‐block metal keteniminyl complexes.^[^ ^50^ ^]^ Given the widespread use of alkali metal reagents in organic synthesis, the keteniminyl anions discussed here provide an intriguing alternative entry point into the versatile chemistry of ketenimines.^[^ ^2^, ^3^, ^4^, ^5^, ^6^ ^]^ To demonstrate the utility of keteniminyl anions in synthesis, we conducted preliminary reactivity studies. To our delight, **3a** readily undergoes transmetalation to form copper complex **9** which could be isolated as an off‐white solid in a good 59% yield (Scheme 2). As a result of the more covalent Cu─C bond (average: 1.928 Å), the C═C bond in **9** (average: 1.278 Å) elongates relative to **3a** (1.222(6) Å), while the C─N bond decreases from 1.288(5) to approximately 1.247 Å. Furthermore, the ^13^C{^1^H} NMR signal of the C1 carbon atom appears slightly downfield shifted (47.7 ppm) relative to the potassium compound.

The potassium salts of **3** were found to be highly reactive, easily engaging in reactions with unsaturated compounds to form highly functionalized heterocycles. For instance, reaction of **3a** or **3b** with phenyl isothiocyanate, tolyl azide, or the α,β‐unsaturated ketone chalcone led to the formation of thiete **6**, triazole **7**, and the 4‐hydropyran derivative **8** through a [2 + 2], [2 + 3] and [2 + 4] cycloaddition, respectively (Scheme 2). All reactions proceeded selectively and allowed the isolation of the heterocycles in good to high yields between 67 and 87%. The connectivity of the heterocycles could unambiguously be confirmed by XRD analysis (see Supporting Information for details). It is important to note that the formation of **7a** and **7b** represents an alternative pathway to mesoionic imines, which have been shown to serve as strong donor ligands.^[^ ^65^ ^]^ In addition, the core moiety in **8**, namely the 2‐amino‐4*H*‐pyran is present in various biologically active molecules,^[^ ^66^ ^]^ emphasizing the potential of keteniminyl anions to access important heterocycles. This is further demonstrated by the reaction of **3a** with carbon dioxide, which occurred in 1:2 stoichiometry and yielded the cyclic anhydride **10**. The 3‐oxauracil core moiety of compound **10** has also exhibited biological activity.^[^ ^67^ ^]^ Compound **10** could be isolated as a colorless solid in a very good yield of 88%. Albeit the mechanism to this novel heterocycle is unknown, it must proceed via the cleavage of one C═O double bond of CO_2_. The ease and high efficiency of these cyclization reactions clearly demonstrate the potential of the keteniminyl anions to access a wide range of cyclic scaffolds via a click chemistry‐like approach. Besides forming heterocycles, the keteniminyl anions also provide access to neutral ketenimines by simple salt metathesis. Treatment of the keteniminyl anions with trimethylchlorosilane selectively furnished the corresponding ketenimines **11a–c**, which could be isolated in moderate to good yields of 54%–72%.

## Conclusion

We have described the PPh_3_/isocyanide and N_2_/isocyanide exchange in metalated ylides and diazomethanides, respectively, as pathways to isolable keteniminyl anions. The ligand exchange reactions proceeded more efficiently with diazo compounds and at a faster rate for the more electron‐deficient aryl isocyanides compared to their alkyl counterparts. The alkali metal complexes of the keteniminyl anions could be isolated in good to excellent yields and spectroscopically as well as crystallographically characterized. All anions feature a bent geometry, with the bending angle decreasing with increasing donor ability of the nitrogen substituent. The structural features of the anions can be described by an intermediate bonding situation between that of a ketenimine and ynamine. While donating groups at nitrogen favor the keteniminyl structure, accepting substituents shifts the bonding situation towards the ynamide structure. Computational studies explain this trend through the π‐accepting properties of the isocyanide ligands in line with the Dewar–Chatt–Duncanson concept commonly applied in transition metal chemistry.

The keteniminyl anions proved to be versatile reagents for forming ketenimines as well as highly functionalized heterocycles by reaction with unsaturated compounds. [2 + 2], [2 + 3], and [2 + 4] cycloadditions allow access to a range of scaffolds, including novel heterocycles. These reactions underscore the potential of ligand exchange processes in generating functional building blocks for synthesis, offering opportunities for future expansion to other ylide precursors, isocyanides, and unsaturated carbon compounds.

## Supporting Information

The authors have cited additional references within the Supporting Information.^[^ ^68^, ^69^, ^70^, ^71^, ^72^, ^73^, ^74^, ^75^, ^76^, ^77^, ^78^, ^79^, ^80^, ^81^, ^82^, ^83^, ^84^, ^85^, ^86^, ^87^, ^88^, ^89^, ^90^, ^91^, ^92^ ^]^

## Author Contributions

S.M. performed all synthetic experiments and spectroscopic analyses, except for compounds **3g** and **11c**, which were synthesized by P.D. A.D. helped with the crystallization and structure analysis of compound **10**, K.‐S.F. with the refinement of all other XRD structures, V.H.G. designed and supervised the experimental part of the project. Y.Z. carried out the quantum chemical calculations. L.Z. and G.F. supervised the theoretical part of the work. The manuscript was written by S.M., G.F., and V.H.G.

## Conflict of Interests

The authors declare no conflict of interest.

## Supporting information



Supporting Information

## Data Availability

The data that support the findings of this study are available in the Supporting Information of this article.
