# Supplementary material for: PPh3/Isocyanide and N2/Isocyanide Exchange: Pathways to Isolable Alkali Metal Keteniminyl Anions
Source: Angew Chem Int Ed Engl. 2025 Mar 22;64(21):e202504325. doi: 10.1002/anie.202504325 (PMC12087817; doi:10.1002/anie.202504325)
Supplement: Supplementary file 1 — Supporting Information [file ANIE-64-e202504325-s001.pdf]

Supporting Information

# $PPh_3$/Isocyanide and $N_2$/Isocyanide Exchange: Pathways to Isolable Alkali Metal Keteniminyl Anions

Sunita Mondal,[1] Yihao Zhang,[2] Prakash Duari,[1] Kai-Stephan Feichtner,[1] Arpan Das,[1] Lili Zhao,[2] Gernot Frenking,[2,3]* Viktoria H. Gessner[1]*

[1]Faculty of Chemistry and Biochemistry, Ruhr-University Bochum, Universitätsstrasse 150, 44801 Bochum, Germany
[2]State Key Laboratory of Materials-Oriented Chemical Engineering, School of Chemistry and Molecular Engineering, Nanjing Tech University, Nanjing 211816, China
[3]Fachbereich Chemie, Philipps-Universität Marburg, Hans-Meerwein-Strasse 4, 35043 Marburg, Germany
*Correspondence: viktoria.gessner@rub.de; frenking@staff.uni-marburg.de

## Table of Contents

**1. Experimental Details ..... 3**
1.1. General experimental details ..... 3
1.2. Synthesis of compound 3a[K·(18-c-6)] ..... 4
1.3. Synthesis of compound 3b[K·(18-c-6)] ..... 4
1.4. Synthesis of compound 3c[K·(18-c-6)] ..... 5
1.5. Synthesis of compound 3d[K·(18-c-6)] ..... 6
1.6. Synthesis of compound 3e[K·(18-c-6)] ..... 6
1.7. Synthesis of compound 3f[K·(18-c-6)] ..... 7
1.8. Synthesis of compound 3g[Li·(12-c-4)] ..... 7
1.9. Synthesis of compound 6 ..... 8
1.10. Synthesis of compound 7a ..... 8
1.11. Synthesis of compound 7b ..... 9
1.12. Synthesis of compound 8 ..... 9
1.13. Synthesis of compound 9 ..... 10
1.14. Synthesis of compound 10 ..... 10
1.15. Synthesis of compound 11a ..... 11
1.16. Synthesis of compound 11b ..... 11
1.17. Synthesis of compound 11c ..... 12
**2. NMR and IR spectra ..... 13**
**3. Crystal structure determination ..... 71**
3.1. General information ..... 71
3.2. Molecular structure of 3a[K·(18-c-6)] ..... 76

3.3. Molecular structure of 3b[K·(18-c-6)] ..... 76
3.4. Molecular structure of 3c[K·(18-c-6)] ..... 77
3.5. Molecular structure of 3d[K·(18-c-6)] ..... 77
3.6. Molecular structure of 3e[K·(18-c-6)] ..... 78
3.7. Molecular structure of 3g[Li·(12-c-4)] ..... 79
3.8. Molecular structure of 6 ..... 80
3.9. Molecular structure of 7b ..... 80
3.10. Molecular structure of 8 ..... 81
3.11. Molecular structure of 9 ..... 81
3.12. Molecular structure of 10 ..... 82
**4. Computational details ..... 83**
4.1. General remarks ..... 83
4.2. Bonding analysis ..... 84
4.3. Cartesian Coordinates ..... 94
**5. References ..... 100**

# 1. Experimental Details

## 1.1. General experimental details

### Reagents

If not stated otherwise, all experiments were carried out using standard Schlenk techniques under an argon atmosphere, which was dry and free of oxygen. Argon (99.999%) was a product of *Air Liquide* and was used without any further drying. An MBraun SPS 800 was used to dry solvents before their usage (THF, toluene, DCM, ACN, *n*-pentane, *n*-hexane). All solvents were stored over molecular sieves under an argon atmosphere. Reagents were purchased from Sigma-Aldrich, ABCR, Acros Organics or TCI Chemicals and used without further purification if not stated otherwise.

**1-O$^{H}$** [$Ph_2P(O)CHPPh_3$], **1-S$^{H}$** [$Ph_2P(S)CHPPh_3$], **1-Se$^{H}$** [$Ph_2P(Se)CHPPh_3$] and **2-O** [K·$Ph_2P(O)CNN$] were synthesized following literature procedures.[1]

*Caution!* Strong bases such as organopotassium bases, especially as neat compounds, are severely air-/moisture-sensitive and pyrophoric organometallic compounds. These compounds need to be handled under an inert gas atmosphere to exclude reactions with oxygen and water. Guidelines for their handling can be found in literature: T. L. Rathman, J. A. Schwindeman, *Org. Process Res. Dev.* **2014**, *18*, 1192.

*Caution!* Carbon dioxide ($CO_2$) easily condensable gas. It should not be added while the reaction mixture is frozen to prevent overpressure inside the system. Removing the gas from the reaction should be done by purging the reaction vessel with Argon or $N_2$ inside a ventilated fume hood.

### Analytical methods

NMR Spectroscopy. $^{1}$H, $^{7}$Li, $^{13}$C{$^{1}$H}, $^{19}$Si{$^{1}$H} $^{31}$P{$^{1}$H} and $^{77}$Se NMR spectra were recorded on Avance-III-400 spectrometers at 22 °C if not stated otherwise. All values of the chemical shift are in ppm regarding the δ-scale. All spin-spin coupling constants (*J*) are printed in Hertz (Hz). To display multiplicities and signal forms correctly the following abbreviations were used: s = singlet, d = doublet, t = triplet m = multiplet, dd = doublet of doublet, br = broad signal. Signal assignment was supported by, HSQC ($^{1}$H / $^{13}$C), HMBC ($^{1}$H / $^{13}$C, $^{1}$H / $^{31}$P) correlation experiments. The measurement conditions for each spectrum are provided in a text box in the corresponding figures included in Chapter 2.

IR spectra were recorded on a Shimadzu IRSpirit with QATR-S module in an argon filled glovebox. Measurement and processing details for all spectra: Temperature: 22 °C; Apodization function – Happ-Genzel, No. of Scans – 40, Resolution – 2 $cm^{-1}$.

Elemental analyses were performed on an Elementar vario MICRO cube elemental analyzer in our in-house facility.

HRMS-LIFDI mass spectra were measured in our in-house facility on a JEOL AccuTof GCv (JMS-T100GCV) (JEOL, Tokyo, Japan) instrument equipped with a LIFDI source from Linden (CMS, Weyhe, Germany). The emitter heating current was set to 20 mA min-1 at a constant

rate.

XRD analyses. For details about the single-crystal Xray diffraction analyses, see chapter 3.

## 1.2. Synthesis of compound 3a[K·(18-c-6)]

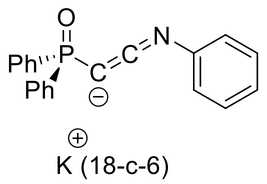

**Path A:** 300 mg (0.630 mmol) **1-O$^H$** and 83.6 mg (0.642 mmol) benzyl potassium were dissolved in 10 mL toluene and stirred for 30 min. After filtration, 166 mg (0.630 mmol) 18-crown-6 were added to the filtrate and stirred for further 10 min. To the resulting clear red solution, 68.3 mg (0.630 mmol) phenyl isocyanide were added and stirred for 2h. After concentrating the solution to half it's volume, 20 mL pentane was added, resulting in the precipitation of a solid. The solid was filtered and washed with pentane (2 x 20 mL). After drying in vacuo, **3a[K·(18-c-6)]** was obtained as brown solid (285 mg, 0.460 mmol, 73%).

**Path B:** 30 mg (0.107 mmol) compound **2-O,** 28.3 mg (0.107 mmol) 18-crown-6 and 11.6 mg (0.107 mmol) phenyl isocyanide were dissolved in 2ml THF. The reaction was stirred overnight at room temperature. The volatiles were removed in *vacuo* and the residue obtained was washed with pentane (2 x 5 mL). After drying the solid in *vacuo*, **3a[K·(18-c-6)]** was obtained as a brown solid (55 mg, 0.089 mmol, 83%).

Single crystals suitable for X-ray diffraction analysis were grown by slow vapor diffusion of pentane into a saturated solution of **3a[K·(18-c-6)]** in THF at -30 °C.

**$^{31}$P{$^1$H}-NMR** (162 MHz, THF-*d*$_8$): δ = 7.00 (s, *P*Ph$_2$O) ppm. **$^1$H-NMR** (400 MHz, THF-*d*$_8$): δ = 8.05 – 7.89 (m, 4H, PC*H*$_{Ph,ortho}$), 7.31 – 7.16 (m, 6H, PC*H*$_{Ph,meta,para}$), 6.95 – 6.82 (m, 4H, NC*H*$_{Ph,ortho,meta}$), 6.44 (t, $^3J_{HH}$ = 6.5 Hz, 1H, NC*H*$_{Ph,para}$), 3.55 (br, 24H, C*H*$_{2,crown}$ + residual THF) ppm.**$^{13}$C{$^1$H}-NMR** (101 MHz, THF-*d*$_8$): δ = 154.9 (d, $^4J_{CP}$ = 3.9 Hz, N*C*$_{Ph,ipso}$), 143.4 (d, $^1J_{CP}$ = 114.9 Hz, P*C*$_{Ph,ipso}$),135.3 (d, $^2J_{CP}$ = 41.9 Hz, PC*C*N), 132.1 (d, $^2J_{CP}$ = 10.1 Hz, P*C*H$_{Ph,ortho}$ ), 129.7 (d, $^4J_{CP}$ = 2.7 Hz, P*C*H$_{Ph,para}$),128.8 (s, N*C*H$_{Ph,ortho}$), 128.0 (d, , $^3J_{CP}$ = 12.0 Hz, P*C*H$_{Ph,meta}$), 121.5 (s, N*C*H$_{Ph,meta}$), 117.1 (s, N*C*H$_{Ph,para}$), 71.00 (s, *C*$_{crown}$), 45.6 (d, $^1J_{CP}$ = 197.3 Hz, P*C*CN) ppm. **FT-IR** (ATR, cm$^{-1}$): 3054.0 (bw), 2885.2 (bw), 2046.5 (s, CCN stretching), 1582.6 (s), 1489.3 (s), 1438.3 (m), 1351.4 (s), 1283.9 (w), 1103.0 (s), 992.4 (m),668.5 (s), 626.2 (s), 535.7 (s), 517.0(s) **Anal. Calcd** for $C_{32}H_{39}KNO_7P$: C, 62.02; H, 6.34; N, 2.26. Found: C, 62.32; H, 6.29; N, 2.52.

## 1.3. Synthesis of compound 3b[K·(18-c-6)]

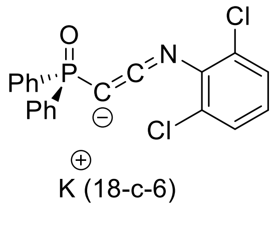

**Path A:** 350 mg (0.735 mmol) **1-O$^H$** and 97.6 mg (0.749 mmol) benzyl potassium were dissolved in 10 mL toluene and stirred for 30 min. After filtration, 194 mg (0.735 mmol) 18-crown-6 were added to the filtrate and further stirred for 10 min. To the resulting clear red solution, 126 mg (0.735 mmol) 2,6-dichlorophenyl isocyanide were added and stirred for 2h. After concentrating the mixture to half it's volume, 20 mL pentane were added resulting in the precipitation of a solid. The solid was filtered and washed

with pentane (2 x 20 mL). After drying in vacuo, **3b[K·(18-c-6)]** was obtained as brown solid (381 mg, 0.553 mmol, 75%).

**Path B:** 30 mg (0.107 mmol) compound **2-O,** 28.3 mg (0.107 mmol) 18-crown-6 and 18.8 mg (0.107 mmol) 2,6-dichlorophenyl isocyanide were dissolved in 2 ml THF. The reaction mixture was stirred for 1h at room temperature. All volatiles were removed in *vacuo* and the residue was washed with pentane (2 x 5 mL). After drying the solid in *vacuo*, **3b[K·(18-c-6)]** was obtained as a brown solid (63 mg, 0.092 mmol, 86%).

Single crystals suitable for X-ray diffraction analysis were grown by slow vapor diffusion of pentane into a saturated solution of **3b[K·(18-c-6)]** in THF at room temperature.

**$^{31}$P{$^{1}$H}-NMR** (162 MHz, THF-$d_8$): δ = 5.72 (s, *P*Ph$_2$O) ppm. **$^{1}$H-NMR** (400 MHz, THF-$d_8$): δ = 7.98 – 7.89 (m, 4H, C$H_{\text{Ph},ortho}$), 7.29 – 7.23 (m, 6H, C$H_{\text{Ph},meta,para}$), 6.94 (d, $^{3}J_{\text{HH}}$ = 7.8 Hz, 2H, C$H_{(2,6\text{-Cl2C6H3}),meta}$), 6.31 (t, $^{3}J_{\text{HH}}$ = 7.8 Hz, 1H, C$H_{(2,6\text{-Cl2C6H3}),para}$), 3.57 (br, 24H, C$H_{2,\text{crown}}$ + residual THF) ppm.**$^{13}$C{$^{1}$H}-NMR** (101 MHz, THF-$d_8$): δ = 145.9 (d, $^{4}J_{\text{CP}}$ = 2.2 Hz, $C_{(2,6\text{-Cl2C6H3}),ipso}$), 142.4 (d, $^{1}J_{\text{CP}}$ = 117.5 Hz, $C_{\text{Ph},ipso}$), 132.1 (d, $^{2}J_{\text{CP}}$ = 10.4 Hz, $C_{\text{Ph},ortho}$ ), 129.8 (d, $^{4}J_{\text{CP}}$ = 2.7 Hz, $C_{\text{Ph},para}$) , 128.6 (s, $C_{(2,6\text{-Cl2C6H3}),meta}$), 128.6 (s, $C_{(2,6\text{-Cl2C6H3}),ortho}$), 128.0 (d, ,$^{3}J_{\text{CP}}$ = 12.2 Hz, $C_{\text{Ph},meta}$), 126.9 (d, $^{2}J_{\text{CP}}$ = 48.7 Hz; PC*C*N), 116.4 (s, $C_{(2,6\text{-Cl2C6H3}),para}$) 71.1 (s, $C_{\text{crown}}$), 44.5 (d, $^{1}J_{\text{CP}}$ = 218.9 Hz, P*C*CN) ppm. **FT-IR** (ATR, cm$^{-1}$): 3051.1 (bw), 2892.4 (bw), 2054.4 (s, CCN stretching), 1567.6 (m), 1506.5 (s), 1385.2 (m), 1103.7 (s), 998.1 (s), 831.5 (m), 708.0 (s), 617.5 (w), 537.8 (s), 525.6(s) **Anal. Calcd** for $C_{32}H_{37}PO_7NCl_2K$: C, 55.81; H, 5.42; N, 2.03. Found: C, 56.38; H, 4.80; N, 2.42.

## 1.4. Synthesis of compound 3c[K·(18-c-6)]

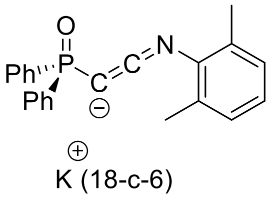


**Path B:** 50 mg (0.178 mmol) compound **2-O,** 47.1 mg (0.178 mmol) 18-crown-6 and 23.9 mg (0.178 mmol) 2,6-dimethylphenyl isocyanide were dissolved in 2 ml THF. The reaction mixture was stirred overnight at room temperature. All volatiles were removed in *vacuo* and the residue obtained was washed with pentane (2 x 5 mL). After drying the solid in vacuo, **3c[K·(18-c-6)]** was obtained as a yellow solid (95 mg, 0.147 mmol, 82%). Single crystals suitable for X-ray diffraction analysis were grown by slow vapor diffusion of pentane into a saturated solution of **3c[K·(18-c-6)]** in THF at room temperature.

**$^{31}$P{$^{1}$H}-NMR** (162 MHz, THF-$d_8$): δ = 8.91 (s, *P*Ph$_2$O) ppm. **$^{1}$H-NMR** (400 MHz, THF-$d_8$): δ = 7.97 – 7.85 (m, 4H, C$H_{\text{Ph},ortho}$), 7.29 – 7.17 (m, 6H, C$H_{\text{Ph},meta,para}$), 6.65 (d, $^{3}J_{\text{HH}}$ = 7.3 Hz, 2H, C$H_{2,6\text{-xyl},meta}$), 6.36 (t, $^{3}J_{\text{HH}}$ = 7.3 Hz, 1H, C$H_{2,6\text{-xyl},para}$), 3.55 (br, 24H, C$H_{2,\text{crown}}$ + residual THF), 2.23 (s, 6H, C$H_3$) ppm. **$^{13}$C{$^{1}$H}-NMR** (101 MHz, THF-$d_8$): δ = 148.2 (d, $^{4}J_{\text{CP}}$ = 3.3 Hz, $C_{2,6\text{-xyl},ipso}$), 143.8 (d, $^{1}J_{\text{CP}}$ = 112.8 Hz, $C_{\text{Ph},ipso}$), 137.2 (d, $^{2}J_{\text{CP}}$ = 41.5 Hz, PC*C*N), 132.1 (d, $^{2}J_{\text{CP}}$ = 9.9 Hz, $C_{\text{Ph},ortho}$ ), 131.2 (s, $C_{2,6\text{-xyl},ortho}$), 129.3 (d, $^{4}J_{\text{CP}}$ = 2.7 Hz, $C_{\text{Ph},para}$) , 128.1 (s, $C_{2,6\text{-xyl},meta}$), 127.83 (d, $^{3}J_{\text{CP}}$ = 11.8 Hz, $C_{\text{Ph},meta}$), 118.2 (s, $C_{2,6\text{-xyl},para}$), 71.0 (s, $C_{\text{crown}}$), 39.0 (d, $^{1}J_{\text{CP}}$ = 185.8 Hz, P*C*CN), 20.5 (s, *C*H$_3$) ppm. **FT-IR** (ATR, cm$^{-1}$): 3049.7 (bw), 2888.1 (bw), 2037.2 (s, CCN stretching), 1587.0 (m), 1488.6 (m), 1350.7 (s), 1100.8 (s), 962.2 (s), 839.4 (m), 696.5 (s), 653.4 (m), 608.2 (w), 532.8 (s). **Anal. Calcd** for $C_{34}H_{43}PO_7NK$: C, 63.04; H, 6.69; N, 2.16. Found: C, 62.73; H, 6.59; N, 2.56.

## 1.5. Synthesis of compound 3d[K·(18-c-6)]

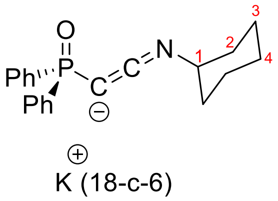


**Path B:** 40 mg (0.143 mmol) compound **2-O** and 37.7 mg (0.143 mmol) 18-crown-6 were dissolved in 2 ml THF and stirred. After 10 min, 15.6 mg (0.143 mmol) cyclohexyl isocyanide was added and the solution was stirred at 45 ˚C for 4d. Vapour diffusion of hexane into reaction mixture resulted in formation of colourless crystals of **3d[K·(18-c-6)]**, which were filtered off and dried in *vacuo* (47 mg, 0.075 mmol, 53%).

**$^{31}$P{$^{1}$H}-NMR** (162 MHz, THF-$d_8$): δ = 15.31 (s, *P*Ph$_2$O) ppm. **$^{1}$H-NMR** (400 MHz, THF-$d_8$): δ = 7.94 – 7.82 (m, 4H, C*H*$_{Ph,ortho}$), 7.23 – 7.13 (m, 6H, C*H*$_{Ph,meta,para}$), 3.60 (br, 24H, C*H*$_{2,crown}$ + residual THF), 3.00 (br, 1H, C*H*$_{cy}$), 1.78-1.67 (m, 4H, C*H*$_{2cy}$ + residual THF), 1.40 – 1.50 (m, 1H, C*H*$_{2cy}$), 1.27 – 1.17(m, 5H, C*H*$_{2cy}$) ppm.**$^{13}$C{$^{1}$H}-NMR** (101 MHz, THF-$d_8$): δ = 151.4 (d, $^{2}J_{CP}$ = 29.1 Hz, PC*C*N), 145.1 (d, $^{1}J_{CP}$ = 103.8 Hz, *C*$_{Ph,ipso}$), 132.1 (d, $^{2}J_{CP}$ = 9.6 Hz, *C*$_{Ph,ortho}$ ), 128.8 (d, $^{4}J_{CP}$ = 2.6 Hz, *C*$_{Ph,para}$) , 127.5 (d, $^{3}J_{CP}$ = 11.3 Hz, *C*$_{Ph,meta}$), 71.0 (s, *C*$_{crown}$), 59.2 (d, $^{4}J_{CP}$ = 5.3 Hz, *C*$_{cy,C1}$), 40.2 (d, $^{1}J_{CP}$ = 133.8 Hz, P*C*CN), 36.9 (s, *C*$_{cy}$), 27.41 (s, *C*$_{cy}$), 26.4 (s, *C*$_{cy}$) ppm. **FT-IR** (ATR, cm$^{-1}$): 3051.8 (bw), 2896.7 (bw), 2852.9 (bw), 1981.2 (s, CCN stretching), 1585.5 (bw), 1434.7 (m), 1348.5 (m), 1104.4 (s), 965.8 (s), 889.0 (w), 710.2 (s), 672.1 (m), 555.7 (m), 528.5 (s). Our repeated attempts on obtaining satisfactory elemental analysis data were unsuccessful.

## 1.6. Synthesis of compound 3e[K·(18-c-6)]

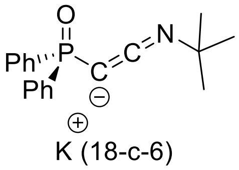


**Path B:** 40 mg (0.143 mmol) compound **2-O** and 37.7 mg (0.143 mmol) 18-crown-6 were dissolved in 2ml of THF and stirred for 10 min. After addition of 18.2 mg (0.214 mmol) *tert*-butyl isocyanide, the reaction was stirred at 45 °C for 2 weeks. All the volatiles were removed in *vacuo* and the brown oil obtained was dissolved in 4ml pentane and 0.5ml toluene mixture. After filtration, the volatiles were removed from the filtrate to obtain the mixture of desired product **3e[K·(18-c-6)**] and excess 18-crown-6 as yellow oil.

Single crystals suitable for X-ray diffraction analysis were grown by slow evaporation of saturated pentane solution of yellow oil {**3e[K·(18-c-6)]** + excess crown} at room temperature.

**$^{31}$P{$^{1}$H}-NMR** (162 MHz, THF-$d_8$): δ = 13.19 (s, *P*Ph$_2$O) ppm. **$^{1}$H-NMR** (400 MHz, THF-$d_8$): δ = 7.93 – 7.84 (m, 4H, C*H*$_{Ph,ortho}$), 7.23 – 7.15 (m, 6H, C*H*$_{Ph,meta,para}$), 3.58 (br, C*H*$_{2,crown}$), 1.09 (s, 9H, C*H*$_3$) ppm.**$^{13}$C{$^{1}$H}-NMR** (101 MHz, THF-$d_8$): δ = 149.9 (d, $^{2}J_{CP}$ = 28.8 Hz, PC*C*N), 145.1 (d, $^{1}J_{CP}$ = 103.6 Hz, *C*$_{Ph,ipso}$), 132.1 (d, $^{2}J_{CP}$ = 9.5 Hz, *C*$_{Ph,ortho}$ ), 128.8 (d, $^{4}J_{CP}$ = 2.6 Hz, *C*$_{Ph,para}$) , 127.5 (d, $^{3}J_{CP}$ = 11.4 Hz, *C*$_{Ph,meta}$), 71.3 (s, *C*$_{crown}$), 53.2 (d, $^{4}J_{CP}$ = 4.5 Hz, *C*(CH$_3$)$_3$, 42.6 (d, $^{1}J_{CP}$ = 133.8 Hz, P*C*CN), 32.4 (d, $^{5}J_{CP}$ = 1.8 Hz, *C*H$_3$) ppm. **FT-IR** (ATR, cm$^{-1}$): 3051.1 (bw), 2886.0 (bw), 2278.5 (bw), 1968.3 (s, CCN stretching), 1578.3(bw), 1350.7 (s), 1103.0 (s), 960.8 (s), 838.0 (m), 696.5 (s), 556.5 (s), 530.7 (s).

## 1.7. Synthesis of compound 3f[K·(18-c-6)]

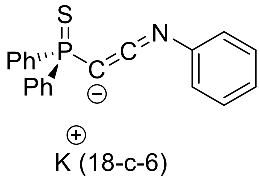

**Path A:** 150 mg (0.305 mmol) **1-S$^{H}$** and 43.6 mg (0.335 mmol) benzyl potassium were dissolved in 10 mL toluene and stirred for 30 min. After filtration, 80.5 mg (0.305 mmol) 18-crown-6 were added to the filtrate and further stirred for 10 min. To the resulting clear red solution, 33.1 mg (0.305 mmol) phenyl isocyanide were added and stirred for 2h. After concentrating the mixture to half it's volume, 20 mL pentane were added resulting in the precipitation of a solid. The solid was filtered and washed with pentane (2 x 10 mL) and diethyl ether (2 x 5 mL). After drying in vacuo, **3f[K·(18-c-6)]** was obtained as brown solid (135 mg, 0.212 mmol, 70%).

**$^{31}$P{$^{1}$H}-NMR** (162 MHz, THF-$d_8$): δ = 19.06 (s, *P*Ph$_2$S) ppm. **$^{1}$H-NMR** (400 MHz, THF-$d_8$): δ = 8.17 – 8.11 (m, 4H, PC$H_{\text{Ph},ortho}$), 7.25 – 7.16 (m, 6H, PC$H_{\text{Ph},meta,para}$), 6.95 – 6.91 (m, 4H, NC$H_{\text{Ph},ortho,meta}$), 6.48 – 6.44 (m, 1H, NC$H_{\text{Ph},para}$), 3.54 (br, 24H, C$H_{2,\text{crown}}$) ppm. **$^{13}$C{$^{1}$H}-NMR** (101 MHz, THF-$d_8$): δ = 155.0 (s, N$C_{\text{Ph},ipso}$), 143.9 (d, $^{1}J_{CP}$ = 92.9 Hz, P$C_{\text{Ph},ipso}$),137.0 (s, PC*C*N), 131.9 (d, $^{2}J_{CP}$ = 11.3 Hz, P*C*H$_{\text{Ph},ortho}$ ), 129.4 (d, $^{4}J_{CP}$ = 2.9 Hz, P*C*H$_{\text{Ph},para}$),128.9 (s, N*C*H$_{\text{Ph,ortho}}$), 127.8 (d, $^{3}J_{CP}$ = 12.5 Hz, P*C*H$_{\text{Ph},meta}$), 121.5 (s, N*C*H$_{\text{Ph},meta}$), 117.2 (s, N*C*H$_{\text{Ph},para}$), 71.1 (s, $C_{\text{crown}}$), 44.8 (d, $^{1}J_{CP}$ = 166.5 Hz, P*C*CN) ppm. **FT-IR** (ATR, cm$^{-1}$): 2886.6 (bw), 2000.4 (s, CCN stretching), 1584.8 (s), 1488.7 (s), 1098.8 (s), 959.2 (s), 835.5 (m), 691.5 (s), 643.5 (w), 540.0 (w), 500.1 (s). Our repeated attempts on obtaining satisfactory elemental analysis data were unsuccessful

## 1.8. Synthesis of compound 3g[Li·(12-c-4)]

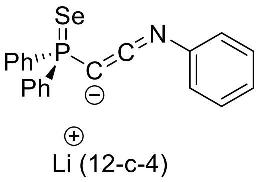

50 mg (0.093 mmol) of **1-Se$^{H}$** and 11.3 mg (0.102 mmol) of LDA were dissolved in 2 mL tol/thf(9:1) mixture. The solution was stirred for 20 min. After filtration, 16.3 mg (0.093 mmol) 12-crown-4 was added and stirred for 10 min. To the resulting solution, 10.1 mg (0.093 mmol) phenyl isocyanide was added and stirred for 2h. The solution was filtered and kept at room temperature for overnight to get off-white precipitation. The solid was washed with toluene (2 times) and pentane (2 times). After drying in vacuo, lithium keteniminyl **3g[Li·(12-c-4)]** was obtained as off-white solid (30 mg, 0.053 mmol, 58 %). Single crystals suitable for X-ray diffraction analysis were grown by slow vapor diffusion of pentane into a saturated solution of **3g[Li·(12-c-4)]** in THF at room temperature.

**$^{31}$P{$^{1}$H}-NMR** (162 MHz, THF-$d_8$): δ = 3.88 (s, *P*Ph$_2$Se) ppm. **$^{1}$H-NMR** (400 MHz, THF-$d_8$): δ = 8.19 – 8.09 (m, 4H, PC$H_{\text{Ph},ortho}$), 7.32 – 7.24 (m, 6H, PC$H_{\text{Ph},meta,para}$), 7.03 – 6.93 (m, 4H, NC$H_{\text{Ph},ortho,meta}$), 6.57 (t, $^{3}J_{HH}$ = 7.1 Hz, $^{4}J_{HH}$ = 1.4 Hz, NC$H_{\text{Ph},para}$), 3.63 (br, 16H, C$H_{2,\text{crown}}$) ppm. **$^{13}$C{$^{1}$H}-NMR** (101 MHz, THF-$d_8$): δ = 154.0 (d, $^{4}J_{CP}$ = 4.2 Hz, N$C_{\text{Ph},ipso}$), 141.4 (d, $^{1}J_{CP}$ = 86.6 Hz, P$C_{\text{Ph},ipso}$),135.2 (d, $^{2}J_{CP}$ = 35.5 Hz, PC*C*N), 132.2 (d, $^{2}J_{CP}$ = 11.7 Hz, P*C*H$_{\text{Ph},ortho}$ ), 130.2 (d, $^{4}J_{CP}$ = 3.0 Hz, P*C*H$_{\text{Ph},para}$),129.4 (s, N*C*H$_{\text{Ph,ortho}}$), 128.2 (d, , $^{3}J_{CP}$ = 12.7 Hz, P*C*H$_{\text{Ph},meta}$), 120.6 (s, N*C*H$_{\text{Ph},meta}$), 118.7 (s, N*C*H$_{\text{Ph},para}$), 71.1 (s, $C_{\text{crown}}$), 46.2 (d, $^{1}J_{CP}$ = 162.0 Hz, P*C*CN) ppm. **$^{7}$Li NMR** (156 MHz, THF-$d_8$) δ = 0.26. **$^{77}$Se-NMR** (76 MHz, THF-$d_8$): δ = –168.44 (d, $^{1}J_{PSe}$ = 708.3 Hz, PPh$_2$*Se*) ppm. **FT-IR** (ATR, cm$^{-1}$): 2074.9 (s, CCN stretching), 1588.5 (m), 1486.0 (m), 1432.8 (m), 1375.4 (bw), 1283.1 (w), 1240.3 (w), 1186.5 (m), 1166.9 (m), 1083.3 (s), 1017.7 (s), 991.7 (w), 927.7 (m), 861.4 (w), 835.7 (w), 823.3 (w), 750.4 (m), 708.8 (s), 688.7

(s), 631.4 (m), 609.0 (m), 500.4 (s), 476.1 (bs) 428.5 (s). **Anal. Calcd.** for $C_{28}H_{31}Li_1N_1O_4P_1Se_1$ : C, 59.79; H, 5.56; N, 2.49. Found: C, 59.54, H, 5.45; N, 2.69.

### 1.9. Synthesis of compound 6

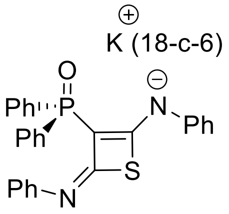


50 mg (0.081 mmol) compound **3a[K·(18-c-6)]** and 11.1 mg (0.081 mmol) phenyl isothiocyanate were dissolved in 2 ml THF and stirred for 1h. The clear red solution obtained was concentrated to half it's volume and 5 mL pentane was added, which resulted in the precipitation of a solid. The solid was filtered and washed with pentane (2 x 5 mL). After drying in vacuo, **6** was obtained as a yellow colour solid (52.8 mg, 0.070 mmol, 87%). Single crystals suitable for X-ray diffraction analysis were grown by slow vapor diffusion of pentane into a saturated solution of **6** in THF at room temperature.

**$^{31}$P{$^1$H}-NMR** (162 MHz, THF-$d_8$): δ = 8.43 (s, *P*Ph$_2$O) ppm. **$^1$H-NMR** (400 MHz, THF-$d_8$): δ = 8.15 – 8.05 (m, 4H, PC*H*$_{Ph,ortho}$), 7.34 – 7.29 (m, 6H, PC*H*$_{Ph,meta,para}$), 7.08 – 7.02 (m, 4H, NC*H*$_{Ph,ortho}$), 6.99 – 6.89 (m, 4H, NC*H*$_{Ph,meta}$), 6.44 (t, $^3J_{HH}$ = 7.3 Hz, 2H, NC*H*$_{Ph,para}$), 3.50 (br, 24H, C*H*$_{2,crown}$) ppm.**$^{13}$C{$^1$H}-NMR** (101 MHz, THF-$d_8$): δ = 155.5 (s, PC*C*N), 153.0 (s, N*C*$_{Ph,ipso}$), 139.6 (d, $^1J_{CP}$ = 105.3 Hz, P*C*$_{Ph,ipso}$), 132.73 (d, $^2J_{CP}$ = 10.5 Hz, P*C*H$_{Ph,ortho}$ ), 130.4 (s, P*C*H$_{Ph,para}$),128.7 (s, N*C*H$_{Ph,ortho}$), 128.1 (d, , $^3J_{CP}$ = 12.1 Hz, P*C*H$_{Ph,meta}$), 123.0 (s, N*C*H$_{Ph,meta}$), 122.2 (s, N*C*H$_{Ph,para}$), 70.9 (s, *C*$_{crown}$), not observed (P*C*CN) ppm. **FT-IR** (ATR, cm$^{-1}$): 3049.0 (bw), 2888.8 (bw), 2827.1 (bw), 1671.7(w), 1536.7 (s), 1103.7 (s), 960.8 (m), 754.0 (m), 694.4 (s), 536.4 (s), 524.9 (s). **Anal. Calcd** for $C_{39}H_{44}KN_2O_7PS$: C, 62.05; H, 5.87; N, 3.71; S, 4.25 Found: C,62.69; H, 5.86; N, 3.86; S,3.10.

### 1.10. Synthesis of compound 7a

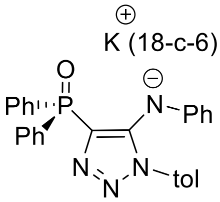


35 mg (0.057 mmol) compound **3a[K·(18-c-6)]** and 7.52 mg (0.057 mmol) p-tolyl azide were dissolved in 2 ml THF and stirred for 1h. After concentrating the obtained clear red solution to half it's volume, 5 mL pentane was added, resulting in the precipitation of a solid. The solid was filtered and washed with pentane (2 x 5 mL). After drying in *vacuo*, **7a** was obtained as a yellow colour solid (36.2 mg, 0.048 mmol, 85%).

**$^{31}$P{$^1$H}-NMR** (162 MHz, THF-$d_8$): δ = 17.93 (s, *P*Ph$_2$O) ppm. **$^1$H-NMR** (400 MHz, THF-$d_8$): δ = 8.06 – 7.96 (m, 4H, PC*H*$_{Ph,ortho}$), 7.79 – 7.65 (m, 2H, C*H*$_{tol,ortho}$), 7.28 – 7.17 (m, 6H, PC*H*$_{Ph,meta,para}$), 6.92 (d, $^3J_{HH}$ = 8.1 Hz, 2H, C*H*$_{tol,meta}$), 6.54 – 6.46 (m, 2H, NC*H*$_{Ph,ortho}$), 6.36 (d, $^3J_{HH}$ = 7.8 Hz, 2H, NC*H*$_{Ph,meta}$), 6.03 (t, $^3J_{HH}$ = 7.2 Hz, 2H, NC*H*$_{Ph,para}$), 3.57 (br, 24H, C*H*$_{2,crown}$ + residual THF), 2.21 (s, 3H, C*H*$_3$) ppm.**$^{13}$C{$^1$H}-NMR** (101 MHz, THF-$d_8$): δ = 155.7 (s, N*C*$_{Ph,ipso}$), 153.6 (d, $^2J_{CP}$ = 32.32 Hz, PC*C*N), 138.7 (s, *C*$_{tol,ipso}$), 138.2 (d, $^1J_{CP}$ = 107.1 Hz, P*C*$_{Ph,ipso}$), 134.0 (s, *C*$_{tol,para}$), 132.8 (d, $^2J_{CP}$ = 10.1 Hz, P*C*H$_{Ph,ortho}$ ), 130.5 (s, P*C*H$_{Ph,para}$), 128.7 (s, *C*$_{tol,meta}$), 128.3 (s, N*C*H$_{Ph,ortho}$), 128.0 (d, $^3J_{CP}$ = 12.1 Hz, P*C*H$_{Ph,meta}$), 121.8 (s, *C*$_{tol,ortho}$),120.4 (s, N*C*H$_{Ph,meta}$), 118.8 (d, $^1J_{CP}$ = 155.9 Hz, P*C*CN), 114.4 (s, N*C*H$_{Ph,para}$), 71.0 (s, *C*$_{crown}$), 21.1 (s, *C*H$_3$) ppm. **FT-IR** (ATR, cm$^{-1}$): 3049.7 (bw), 2887.4 (bw), 1522.3 (s), 1502.2 (s), 1484.3 (s), 1103.0 (s), 960.8 (s), 817.9 (m), 691.5 (s), 560.1 (s), 529.2 (s). **Anal. Calcd** for $C_{39}H_{46}KN_4O_7P$: C, 62.22; H, 6.16; N, 7.44. Found: C, 62.11; H, 6.46; N, 7.25.

## 1.11. Synthesis of compound 7b

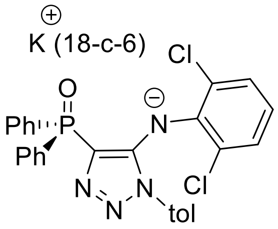

50 mg (0.073 mmol) compound **3b[K·(18-c-6)]** and 9.67 mg (0.073 mmol) p-tolyl azide were dissolved in 2ml THF and stirred for overnight. The next day brown solution obtained was concentrated to half it's volume and 5 mL pentane was added, which resulted in the precipitation of a solid. The solid was filtered and washed with pentane (2 x 5 mL). After drying in *vacuo*, **7b** was obtained as a off-white colour solid (49.1 mg, 0.060 mmol, 82%). Single crystals suitable for X-ray diffraction analysis were grown by slow vapor diffusion of pentane into a saturated solution of **7b** in THF at room temperature.

**$^{31}$P{$^1$H}-NMR** (162 MHz, THF-$d_8$): δ = 20.07 (s, *P*Ph$_2$O) ppm. **$^1$H-NMR** (400 MHz, THF-$d_8$): δ = 8.48 – 8.29 (m, 4H, C$H_{Ph,ortho}$), 7.36 – 7.26 (m, 6H, C$H_{Ph,meta,para}$), 7.12 (d, $^3J_{HH}$ = 8.1 Hz, 2H, C$H_{tol,ortho}$), 6.80 (d, $^3J_{HH}$ = 8.1 Hz, 2H, C$H_{tol,meta}$), 6.70 (d, $^3J_{HH}$ = 7.8 Hz, 2H, C$H_{(2,6\text{-}Cl2C6H3),meta}$), 6.06 (t, $^3J_{HH}$ = 7.8 Hz, 1H, C$H_{(2,6\text{-}Cl2C6H3),para}$), 3.59 (br, 24H, C$H_{2,crown}$ + residual THF), 2.15 (s, 3H, C$H_3$) ppm.**$^{13}$C{$^1$H}-NMR** (101 MHz, THF-$d_8$): δ = 151.3 (d, $^2J_{CP}$ = 31.1 Hz, PCCN), 149.7 (s, $C_{(2,6\text{-}Cl2C6H3),ipso}$), 138.2 (d, $^1J_{CP}$ = 108.0 Hz, $C_{Ph,ipso}$), 137.1 (s, $C_{tol,ipso}$), 135.5 (s, $C_{tol,para}$), 132.9 (d, $^2J_{CP}$ = 10.1 Hz, $CH_{Ph,ortho}$ ), 131.0 (d, $^4J_{CP}$ = 2.8 Hz, $C_{Ph,para}$), 128.32 (s, $C_{tol,meta}$), 128.1 (d, $^3J_{CP}$ = 12.5 Hz, $C_{Ph,meta}$), 127.9 (s, $C_{(2,6\text{-}Cl2C6H3),meta}$), 127.80 (s, $C_{(2,6\text{-}Cl2C6H3),ortho}$), 124.6 (s, $C_{tol,ortho}$), 118.93 (d, $^1J_{CP}$ = 149.7 Hz, P*C*CN), 115.6 (s, $C_{(2,6\text{-}Cl2C6H3),para}$), 71.1 (s, $C_{crown}$), 21.1 (s, *C*H$_3$) ppm. **FT-IR** (ATR, cm$^{-1}$): 3052.6 (bw), 2896.7 (bw), 1584.8 (m), 1542.4 (s), 1434.0 (m), 1105.8 (s), 820.8 (m), 754.7 (m), 609.6 (w), 566.6 (s), 533.5 (s). **Anal. Calcd** for $C_{39}H_{44}Cl_2KN_4O_7P$: C, 57.00; H, 5.40; N, 6.82. Found: C, 57.22; H, 5.09; N, 6.92.

## 1.12. Synthesis of compound 8

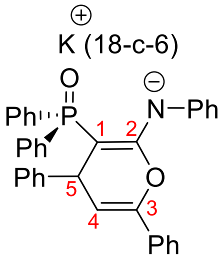

50 mg (0.081 mmol) compound **3a[K·(18-c-6)]** and 16.8 mg (0.081 mmol) of trans-Chalkon were dissolved in 2ml THF and stirred for 1h. After concentrating the obtained brown solution to half it's volume, 5 mL hexane was added, resulting in the precipitation of a solid. After filtration , filtrate was collected in a vial. Slow evaporation of filtrate yielded the product as yellow block shape crystals (45.8 mg, 0.054 mmol, 67% ). The single crystals were used for X-ray diffraction analysis.

**$^{31}$P{$^1$H}-NMR** (162 MHz, $C_6D_6$): δ = 27.82 (s, *P*Ph$_2$O) ppm. **$^1$H-NMR** (400 MHz, $C_6D_6$): δ = 8.33 – 8.27 (m, 2H, C$H_{Ph}$), 7.96 – 7.94 (m, 2H, C$H_{Ph}$), 7.85 – 7.83 (m, 2H, C$H_{Ph}$), 7.46 – 7.43 (m, 2H, C$H_{Ph}$), 7.22 – 7.08 (m, 14H, C$H_{Ph}$), 6.85 – 6.83 (m, 2H, C$H_{Ph}$), 6.79 (t, $^3J_{HH}$ = 7.2 Hz, 1H, C$H_{Ph}$), 5.94 (dd, $^4J_{PH}$ = 4.1 Hz , $^3J_{HH}$ = 6.4 Hz, 1H, C$H_{pyran,sp2}$), 4.31 – 4.28 (m, 1H, C$H_{pyran,sp3}$), 3.18 (br, 24H, C$H_{2,crown}$) ppm. **$^{13}$C{$^1$H}-NMR** (101 MHz, $C_6D_6$): 159.1 (d, $^2J_{CP}$ = 16.0 Hz, PCCN), 152.9 (s), 152.04 (s), 148.6 (s, $C_{pyran,C3}$), 142.9 (d, $^1J_{CP}$ = 113.4 Hz, P$C_{Ph,ipso}$), 139.7 (d, $^1J_{CP}$ = 99.1 Hz, P$C_{Ph,ipso}$), 136.40 (s), 133.0 (d, $J_{CP}$ = 8.1 Hz), 132.8 (d, $J_{CP}$ = 8.7 Hz), 129.2 (d, $J_{CP}$ = 2.4 Hz), 128.7 (d, $J_{CP}$ = 2.7 Hz), 128.4 (d, $^2J_{CP}$ = 10.2 Hz, PC$H_{Ph,ortho}$), 127.9 (s, PC$H_{Ph,para}$), 127.7 (s), 127.6 (d, $^3J_{CP}$ = 14.5 Hz, PC$H_{Ph,meta}$), 125.7 (s), 125.3 (s), 124.9 (s), 117.5 (s), 105.2 (d, $^4J_{CP}$ = 10.5 Hz, $CH_{pyran,C4}$), 69.9 (s, $C_{crown}$), 60.3 (d, $^1J_{CP}$ = 140.6 Hz, P*C*CN), 41.9 (d, $^3J_{CP}$ = 12.7 Hz, $CH_{pyran,C5}$). **FT-IR** (ATR, cm$^{-1}$): 3051.8 (bw), 2884.5 (bw), 1660.2 (w), 1530.9 (s), 1486.4 (s), 1350.7 (m), 1105.1 (s), 960.1 (m), 753.3 (m), 692.2 (s),

556.5 (s), 523.5 (s). **Anal. Calcd** for $C_{47}H_{51}KNO_8P$: C, 68.18; H, 6.21; N, 1.69. Found: C, 68.19; H, 5.89; N, 1.91.

## 1.13. Synthesis of compound 9

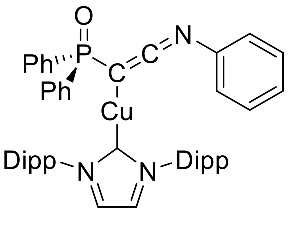


20 mg (0.032 mmol) of **3a[K·(18-c-6)]** and 15.7 mg (0.032 mmol) IPrCuCl were dissolved in 2 ml toluene and stirred overnight. Next day, all the volatiles were removed in *vacuo* and the resulting solid was washed with hexane (6 x 5 mL). This yielded **9** as an off-white colour solid (14.6 mg, 0.019 mmol, 59%). Single crystals suitable for X-ray diffraction analysis were grown by slow vapor diffusion of pentane into a saturated solution of **9** in benzene at room temperature.

**$^{31}$P{$^1$H}-NMR** (162 MHz, THF-$d_8$): δ = 21.71 (s, *P*$Ph_2O$) ppm. **$^1$H-NMR** (400 MHz, THF-$d_8$): δ = 7.53 – 7.44 (m, 6H,$C_{sp2}H$), 7.41 – 7.34 (m, 3H, $C_{sp2}H$), 7.27 – 7.20 (m, 4H, $C_{sp2}H$), 7.18 – 7.16 (m, 2H, $C_{sp2}H$), 7.10 – 7.03 (m, 5H, $C_{sp2}H$), 6.91 – 6.88 (m, 1H, $C_{sp2}H$), 6.65 (d, $^2J_{HH}$ = 7.9 Hz, 2H, $C_{sp2}H$), 2.59 (septet, 4H, $^3J_{HH}$ = 6.9 Hz, C*H*($CH_3$)$_2$), 1.32 – 1.13 (m, 24H, C*H*$_3$) ppm. **$^{13}$C{$^1$H}-NMR** (101 MHz, THF-$d_8$): δ = 182.5 (s, Cu$C_{NHC}$), 168.9 (d, $^2J_{CP}$ = 6.3 Hz, PC*C*N), 146.6 (s, $C_{sp2}$), 146.0 (d, $^4J_{CP}$ = 5.9 Hz N$C_{Ph,ipso}$), 141.2 (d, $^1J_{CP}$ = 103.8 Hz, P$C_{Ph,ipso}$), 135.8 (s, $C_{sp2}$), 131.7 (d, $^2J_{CP}$ = 9.0 Hz, P$CH_{Ph,ortho}$), 131.1 (s, $C_{sp2}$), 130.0 (d, $^4J_{CP}$ = 2.7 Hz, P$CH_{Ph,para}$), 129.2 (s, $C_{sp2}$), 128.14 (d, $^2J_{CP}$ = 11.6 Hz, P$CH_{Ph,meta}$), 124.8 (s, $C_{sp2}$), 124.5 (s, $C_{sp2}$), 124.0 (s, $C_{sp2}$), 123.8 (s, $C_{sp2}$), 47.8 (d, $^1J_{CP}$ = 84.3 Hz, P*C*CN), 29.7 (s, *C*H($CH_3$)$_2$), 25.3 (s, $CH_3$), 24.08 (s, $CH_3$) ppm. **FT-IR** (ATR, $cm^{-1}$): 3053.3 (bw), 2961.4 (m), 1951.0 (s, CCN stretching), 1592.0 (m), 1528.8 (m), 1468.5 (s), 1112.3 (s), 946.4 (w), 802.1 (m), 756.1 (s), 693.7 (s), 539.3 (s). **HRMS-LIFDI** (m/z): [M] $^{\cdot+}$ calcd for $C_{47}H_{51}CuN_3OP$, 767.3066; found, 767.3060.

## 1.14. Synthesis of compound 10

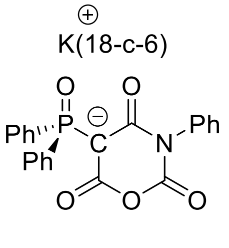


30 mg (0.048 mmol) of **3a[K·(18-c-6)]** was dissolved in 3ml THF. The atmosphere in the flask was changed from argon to $CO_2$ and the reaction was stirred for 24 hours. The clear red solution obtained was concentrated to 1 mL and 5 mL pentane was added to get precipitation. After filtration, solid obtained was washed with pentane (3 x 5 mL). The solid was dried to yield **10** as an off-white colour solid. (30 mg, 0.042 mmol, 88 %). In J-young NMR tube **10** was taken and dissolved in 1:1 mixture of benzene/pentane. To the turbid solution few drops of THF was added to obtain clear solution. After 2 days on walls of the NMR tube single crystals appeared, which were used for X-ray diffraction analysis.

**$^{31}$P{$^1$H}-NMR** (162 MHz, $C_6D_6$): δ = 27.78(s, *P*$Ph_2O$) ppm. **$^1$H-NMR** (400 MHz, THF-$d_8$): δ = 7.90 – 7.81 (m, 4H, PC$H_{Ph,ortho}$), 7.33 – 7.23 (m, 8H, PC$H_{Ph,meta,para}$ + NC$H_{Ph,ortho}$), 7.19 – 7.13 (m, 3H, NC$H_{Ph,meta,para}$), 3.51 (br, 24H, C$H_{2,crown}$) ppm.**$^{13}$C{$^1$H}-NMR** (101 MHz, THF-$d_8$): δ = 165.5 (d, $^2J_{CP}$ = 11.4 Hz, PC*C*(O)O), 161.3 (d, $^2J_{CP}$ = 14.4 Hz, PC*C*(O)N), 151.1 (s, O*C*(O)N), 138.9 (d, $^1J_{CP}$ = 109.2 Hz, P$C_{Ph,ipso}$), 138.6 (s, N$C_{Ph,ipso}$), 132.8 (d, $^2J_{CP}$ = 9.9 Hz, P$CH_{Ph,ortho}$ ), 130.6 (s, P$CH_{Ph,para}$),130.6 (s, N$CH_{Ph,ortho}$), 128.7 (s, N$CH_{Ph,meta}$), 127.9 (d, $^3J_{CP}$ = 12.3 Hz, P$CH_{Ph,meta}$), 127.5 (s, N$CH_{Ph,para}$), 71.0 (s, $C_{crown}$), 70.5 (d, $^1J_{CP}$ = 133.3 Hz, P$C^-$) ppm. **FT-IR** (ATR, $cm^{-1}$): 3051.8 (bw), 2888.1 (bw), 1748.5 (s), 1683.9 (m), 1641.5 (s), 1545.3 (w), 1301.9

(s),1105.1 (s), 960.8 (s), 837.3 (w), 766.9 (m), 692.2 (s), 527.8 (s). **Anal. Calcd** for $C_{34}H_{39}KNO_{11}P$: C, 57.70; H, 5.55; N, 1.98. Found: C, 57.79; H, 5.50; N, 2.36.

## 1.15. Synthesis of compound 11a

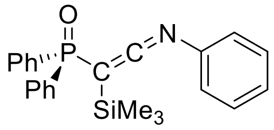

50 mg (0.081 mmol) compound **3a[K·(18-c-6)]** was dissolved in 2 mL THF. To the resulting solution, 11.4 µL (0.086 mmol) chlorotrimethylsilane was added at –30 °C and stirred for 10 min. The volatiles were removed in vacuo and the residue was extracted with 5 mL pentane. Filtration and subsequent removal of the volatiles in vacuo yielded **11a** as a yellow oil. (22 mg, 0.056 mmol, 70 %).

**$^{31}$P{$^{1}$H}-NMR** (162 MHz, THF-$d_8$): δ = 22.66 (s, *P*Ph$_2$O) ppm. **$^{1}$H-NMR** (400 MHz, THF-$d_8$): δ = 7.82 – 7.77 (m, 4H, PC$H_{\text{Ph,}ortho}$), 7.43 – 7.39 (m, 6H, PC$H_{\text{Ph,}meta,para}$), 7.27 – 7.22 (m, 2H, NC$H_{\text{Ph,}meta}$), 7.18 – 7.14 (m, 1H, NC$H_{\text{Ph,}para}$), 6.85 (d, $^3J_{HH}$ = 7.3 Hz, 2H, NC$H_{\text{Ph,}ortho}$), 0.21 (s, 9H, C$H_3$) ppm.**$^{13}$C{$^{1}$H}-NMR** (101 MHz, THF-$d_8$): δ = 139.7 (d, $^4J_{CP}$ = 5.0 Hz, N$C_{\text{Ph,}ipso}$), 137.3 (d, $^1J_{CP}$ = 105.7 Hz, P$C_{\text{Ph,}ipso}$), 132.1 (s, P$C$H$_{\text{Ph,}para}$),132.0 (d, $^2J_{CP}$ = 9.9 Hz, P$C$H$_{\text{Ph,}ortho}$ ), 130.3 (s, N$C$H$_{\text{Ph,meta}}$), 129.1 (d, $^3J_{CP}$ = 12.1 Hz, P$C$H$_{\text{Ph,}meta}$), 127.8 (s, N$C$H$_{\text{Ph,}para}$), 124.2 (d, $^5J_{CP}$ = 1.9 Hz, N$C$H$_{\text{Ph,}ortho}$), 47.5 (d, $^1J_{CP}$ = 81.5 Hz, P$C$CN), 0.48 (s, $C$H$_3$) not observed (PC$C$N) ppm. **$^{29}$Si NMR** shifts were extracted from the indirect dimension of a $^1$H, $^{29}$Si HMBC experiment: 3.20 ppm. **FT-IR** (ATR, cm$^{-1}$): 2955.6 (bw), 2011.3 (s, CCN stretching), 1976.2 (bw),1590.6 (m), 1528.1 (w), 1490.0 (m), 1460.6 (s), 1437.6 (bw), 1361.5 (s), 1016.1 (w), 839.4 (s), 750.4 (m), 719.5 (m), 689.4(s), 653.5(w), 510.5(s). **HRMS-LIFDI** (m/z): [M] $^{\bullet+}$ calcd for $C_{23}H_{24}NPOSi$, 389.1365; found, 389.1439.

## 1.16. Synthesis of compound 11b

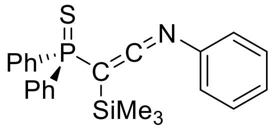

50 mg (0.079 mmol) compound **3f[K·(18-c-6)]** was dissolved in 2 mL THF. To the resulting solution, 11.2 µL (0.086 mmol) chlorotrimethylsilane was added and stirred for 10 min. The volatiles were removed *in vacuo* and the residue was extracted with 5 mL pentane. Filtration and subsequent removal of the volatiles *in vacuo* yielded **11b** as a yellow oil. (23 mg, 0.057 mmol, 72 %).

**$^{31}$P{$^{1}$H}-NMR** (162 MHz, THF-$d_8$): δ = 40.37 (s, *P*Ph$_2$S) ppm. **$^{1}$H-NMR** (400 MHz, THF-$d_8$): δ = 7.94 – 7.89 (m, 4H, PC$H_{\text{Ph,}ortho}$), 7.45 – 7.35 (m, 6H, PC$H_{\text{Ph,}meta,para}$), 7.27 – 7.22 (m, 2H, NC$H_{\text{Ph,}meta}$), 7.19 – 7.15 (m, 1H, NC$H_{\text{Ph,}para}$), 6.85 (d, $^3J_{HH}$ = 7.8 Hz, 2H, NC$H_{\text{Ph,}ortho}$), 0.23 (s, 9H, C$H_3$) ppm.**$^{13}$C{$^{1}$H}-NMR** (101 MHz, THF-$d_8$): δ = 139.4 (d, $^4J_{CP}$ = 5.2 Hz, N$C_{\text{Ph,}ipso}$), 136.5 (d, $^1J_{CP}$ = 86.2 Hz, P$C_{\text{Ph,}ipso}$),134.4 (d, $^2J_{CP}$ = 10.3 Hz, PC$C$N), 132.4 (d, $^2J_{CP}$ = 10.7 Hz, P$C$H$_{\text{Ph,}ortho}$ ), 132.0 (d, $^4J_{CP}$= 3.1 Hz, P$C$H$_{\text{Ph,}para}$),130.3 (s, N$C$H$_{\text{Ph,}meta}$), 129.0 (d, $^3J_{CP}$ = 12.7 Hz, P$C$H$_{\text{Ph,}meta}$), 128.0 (s, N$C$H$_{\text{Ph,}para}$), 124.3 (d, $^5J_{CP}$ = 1.8 Hz, N$C$H$_{\text{Ph,}ortho}$), 48.9 (d, $^1J_{CP}$ = 61.6 Hz, P$C$CN), 0.69 (s, $C$H$_3$), ppm. **$^{29}$Si NMR** shifts were extracted from the indirect dimension of a $^1$H, $^{29}$Si HMBC experiment: 2.91 ppm **FT-IR** (ATR, cm$^{-1}$): 2954.2 (bw), 2002.7 (s, CCN stretching), 1967.5 (bw),1591.3 (m), 1490.0 (m), 1452.7 (w), 1436.2 (s), 1248.7 (s), 1179.8 (w), 1030.9 (s), 838.7 (s), 784.1 (w), 745.4 (m), 655.6(s), 547.2(m), 476.1(m). **HRMS-LIFDI** (m/z): [M] $^{\bullet+}$ calcd for $C_{23}H_{24}NPSSi$, 405.1136; found, 405.1113.

## 1.17. Synthesis of compound 11c

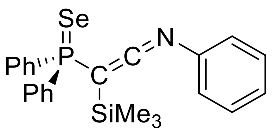

30 mg (0.053 mmol) compound **3g[Li·(12-c-4)]** was dissolved in 2 mL THF. To the resulting solution 7.6 µL (0.058 mmol) chlorotrimethylsilane was added and stirred for 10 min. The volatiles were removed *in vacuo* and the residue was extracted with 5 mL pentane. Filtration and subsequent removal of the volatiles *in vacuo* yielded **11c** as a yellow oil. (13 mg, 0.029 mmol, 54 %).

**$^{31}$P{$^{1}$H}-NMR** (162 MHz, THF-$d_8$): δ = 28.77 (s, *P*$Ph_2$Se) ppm. **$^{1}$H-NMR** (400 MHz, THF-$d_8$): δ = 7.98 – 7.88 (m, 4H, PC$H_{Ph,ortho}$), 7.44 – 7.35 (m, 6H, PC$H_{Ph,meta,para}$), 7.29 – 7.23 (m, 2H, NC$H_{Ph,meta}$), 7.20 – 7.15 (m, 1H, NC$H_{Ph,para}$), 6.87 (d, $^{3}J_{HH}$ = 7.5 Hz, NC$H_{Ph,ortho}$), 0.24 (s, Si(C$H_3$)$_3$). **$^{13}$C{$^{1}$H}-NMR** (101 MHz, THF-$d_8$): δ = 139.5 (d, $^{4}J_{CP}$ = 5.8 Hz, N$C_{Ph,ipso}$), 135.2 ( d, $^{1}J_{CP}$ = 77.1 Hz, P$C_{Ph,ipso}$), 134.1 (d, $^{2}J_{CP}$ = 20.7 Hz, PC*C*N), 133.1 (d, $^{2}J_{CP}$ = 11.7 Hz, P*C*$H_{Ph,ortho}$ ), 132.2 (d, $^{4}J_{CP}$ = 3.0 Hz, P*C*$H_{Ph,para}$), 130.4 (s, N*C*$H_{Ph,meta}$), 129.2 ( $^{3}J_{CP}$ = 12.6 Hz, P*C*$H_{Ph,meta}$), 128.2 ( s, N*C*$H_{Ph,para}$), 124.5 (d, $^{5}J_{CP}$ = 2.1 Hz, N*C*$H_{Ph,ortho}$), 48.1 (d, $^{2}J_{CP}$ = 58.1 Hz, P*C*CN), 0.8 (s, Si(*C*$H_3$)$_3$). **$^{29}$Si NMR** shifts were extracted from the indirect dimension of a $^{1}$H, $^{29}$Si HMBC experiment: 3.06 ppm. **FT-IR** (ATR, cm$^{-1}$): 2952.7 (bw), 2233.2 (bw), 2001.3 (s, CCN stretching), 1964.7 (bw),1591.3 (m), 1563.3 (w), 1490.0 (m), 1435.4 (s), 1307.6 (w), 1286.8 (m), 900.5 (s), 838.7 (w), 784.1 (s), 687.2 (s), 518.4(m), 474.7(m). **HRMS-LIFDI** (m/z): [M] $^{•+}$ calcd for $C_{23}H_{24}NPSeSi$, 453.0581; found, 453.0575

## 2. NMR and IR spectra

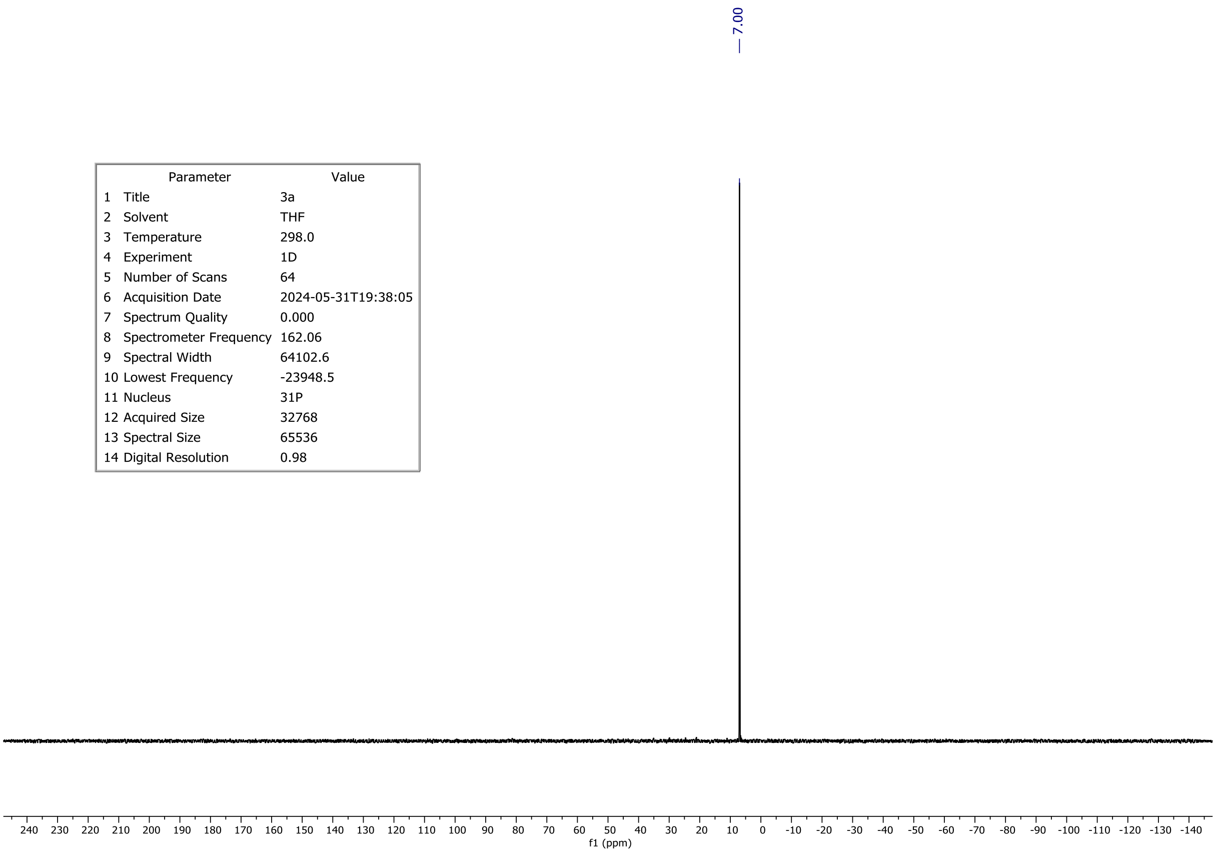


**Figure S1** $^{31}P\{^{1}H\}$ NMR spectrum of compound **3a**[K·(18-c-6)] in THF-$d_8$.

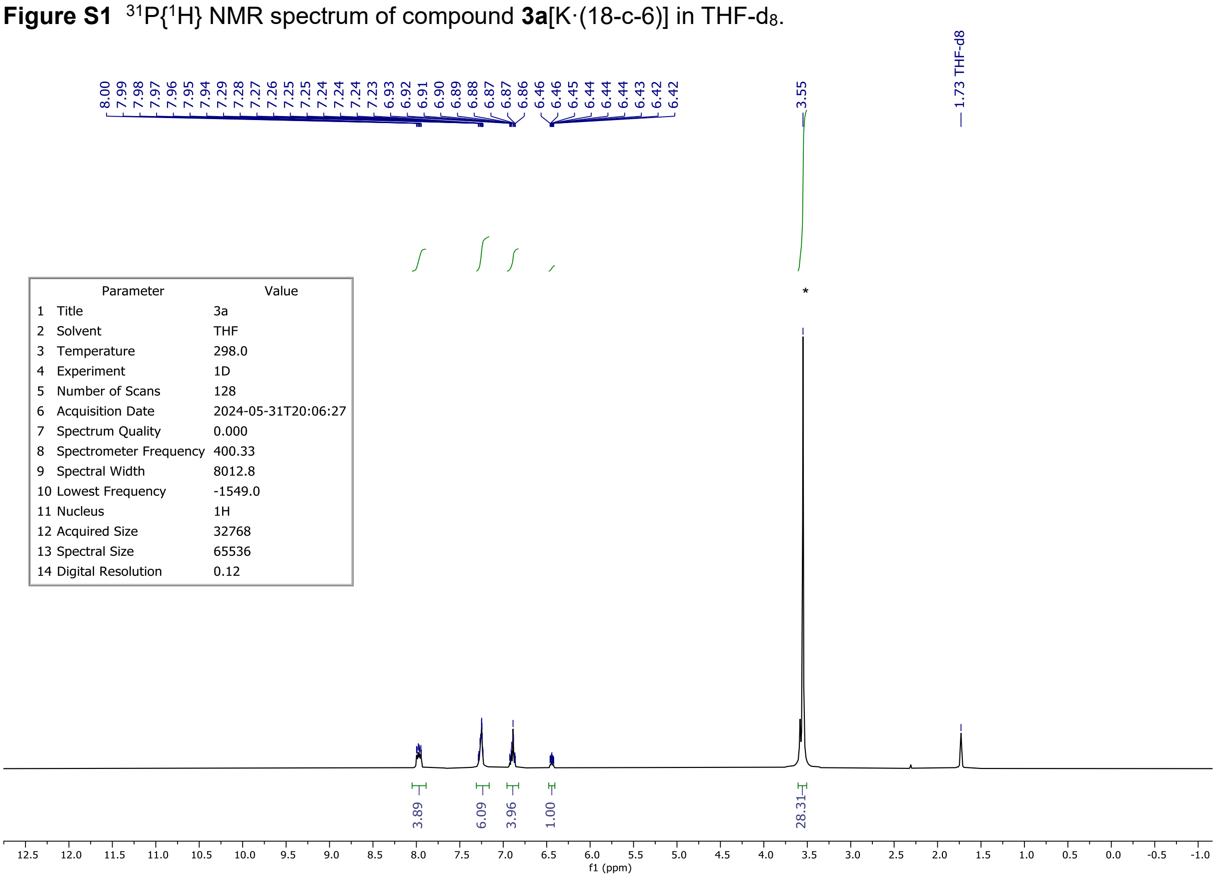


**Figure S2** $^{1}H$ NMR spectrum of compound **3a**[K·(18-c-6)] in THF-$d_8$ (* = residual THF + 18-c-6).

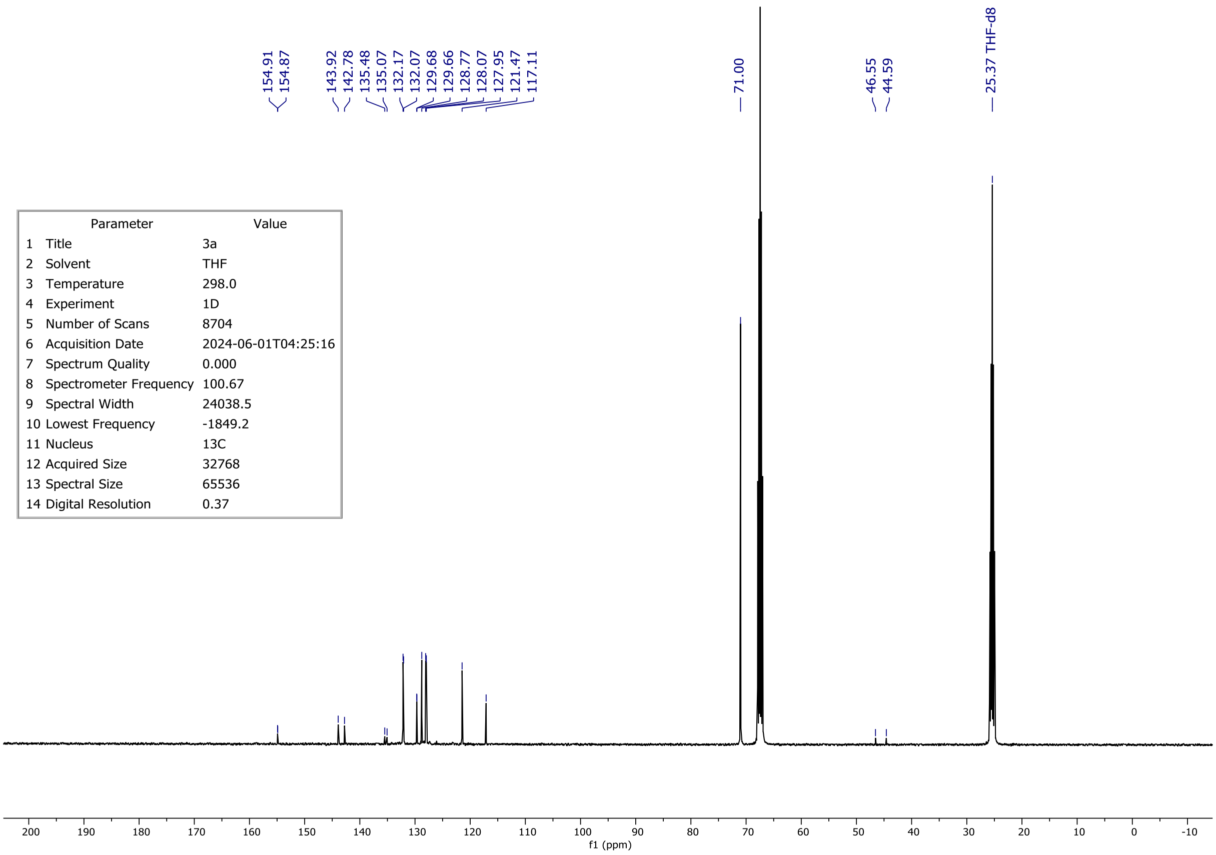


**Figure S3** $^{13}C\{^{1}H\}$ NMR spectrum of compound **3a**[K·(18-c-6)] in THF-$d_8$.

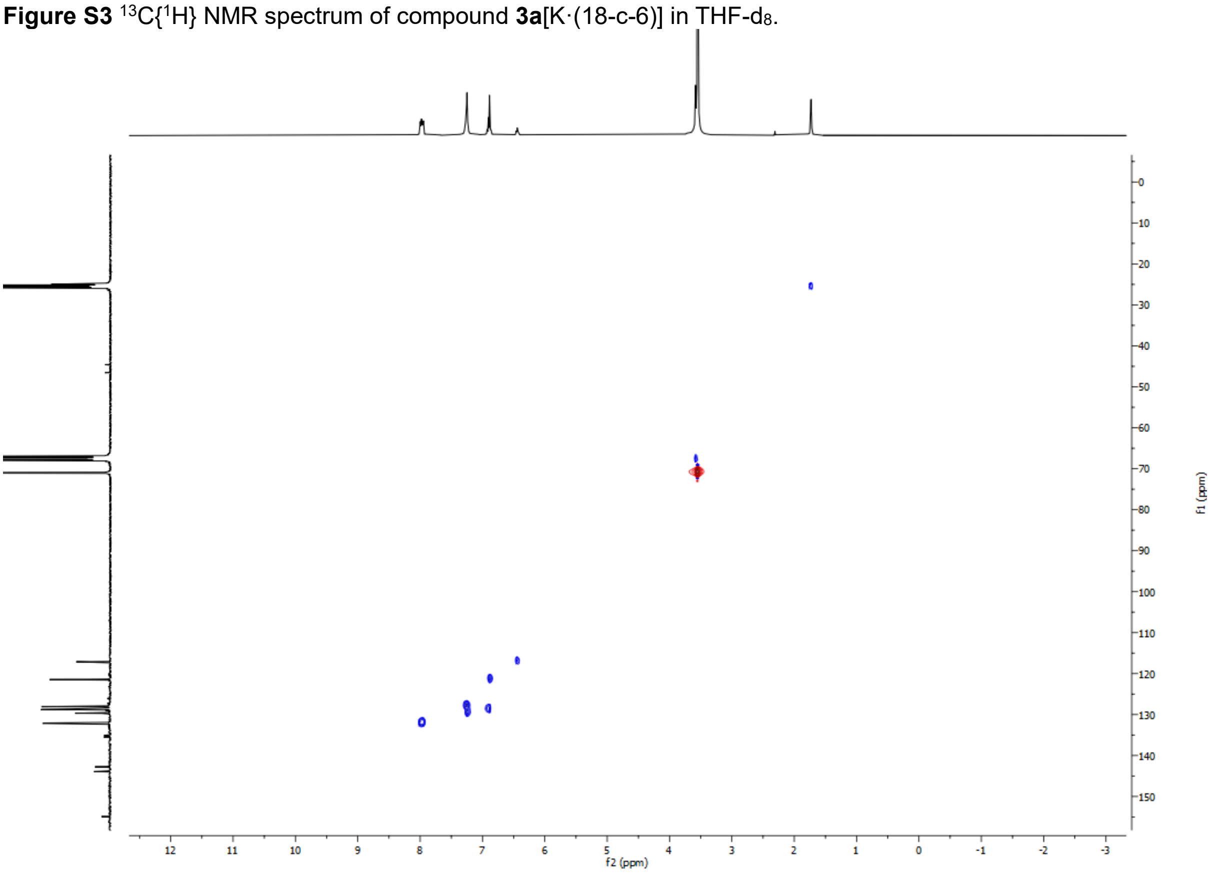


**Figure S4** $^{1}H$-$^{13}C$ HSQC NMR spectrum of compound **3a**[K·(18-c-6)] in THF-$d_8$.

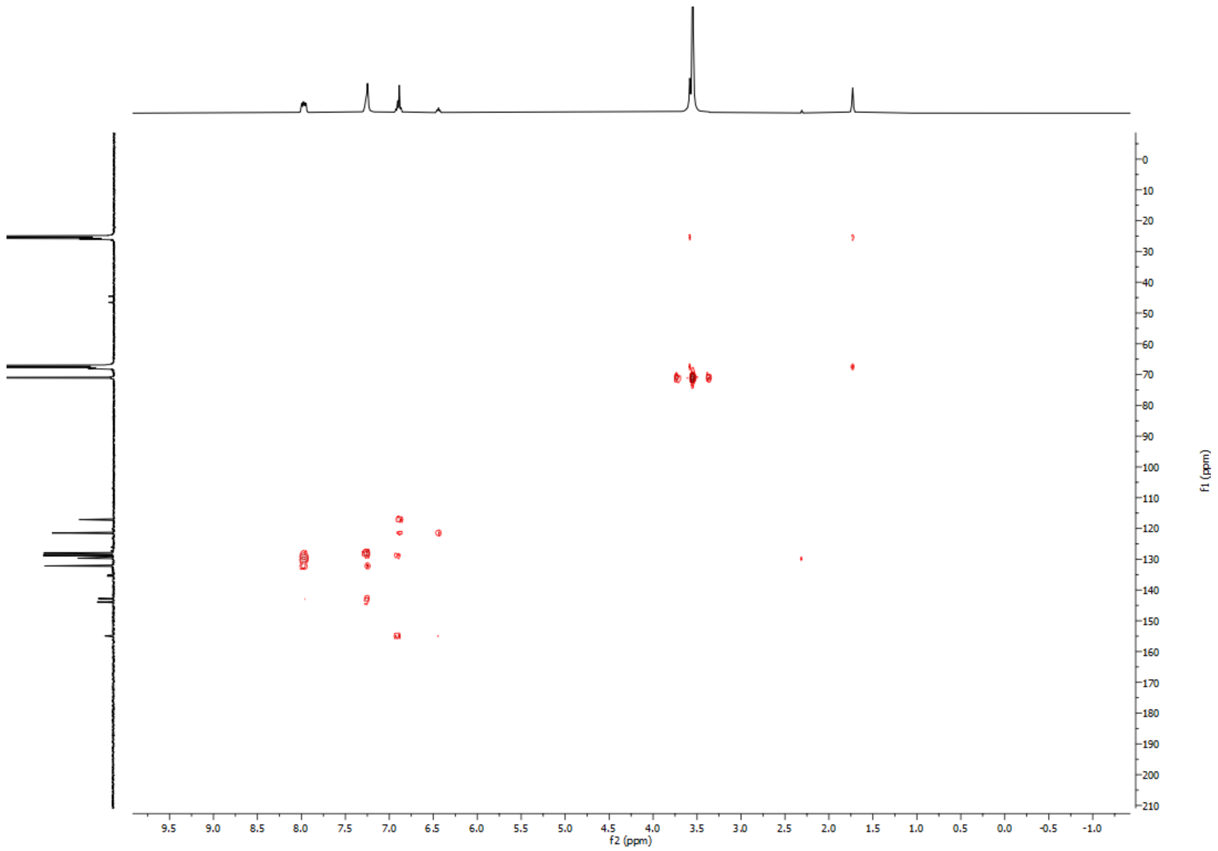


**Figure S5** $^{1}H$-$^{13}C$ HMBC NMR spectrum of compound **3a**[K·(18-c-6)] in THF-$d_8$.

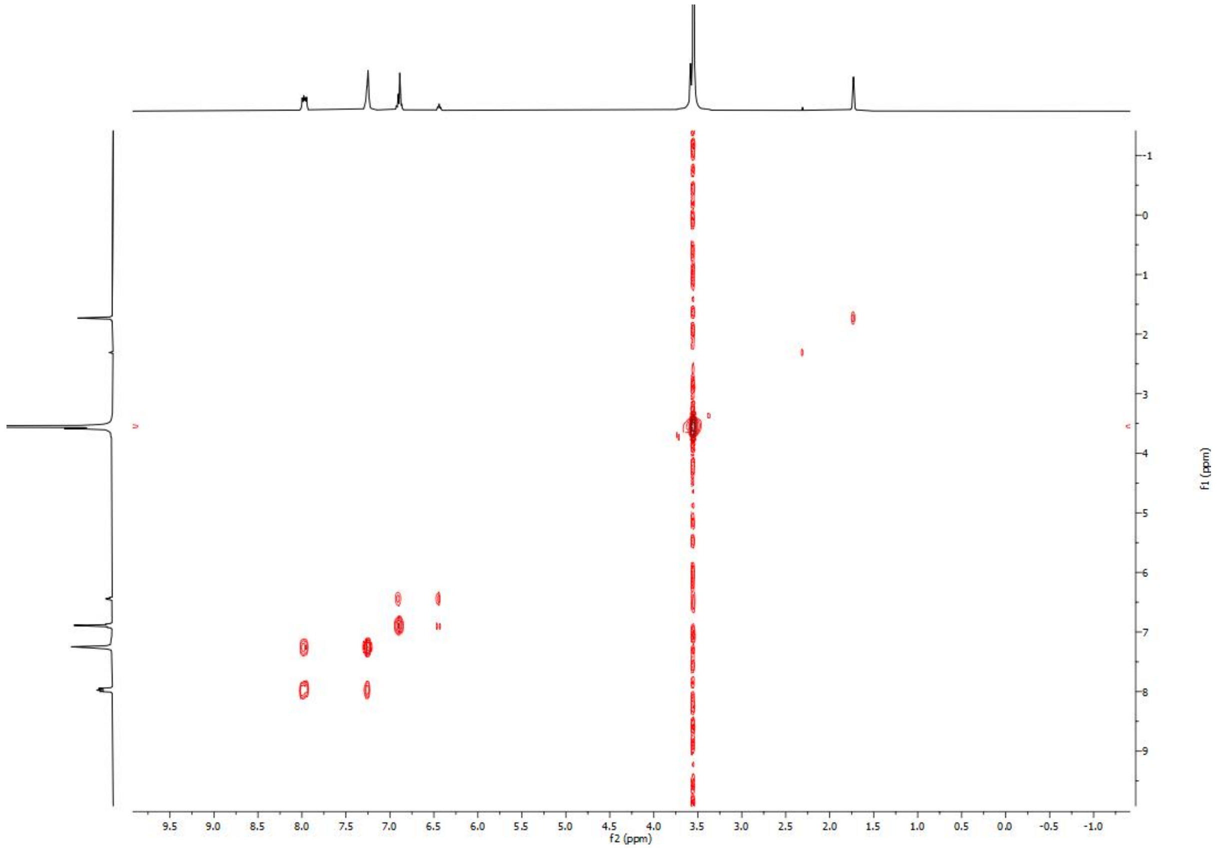


**Figure S6** $^{1}H$-$^{1}H$ COSY NMR spectrum of compound **3a**[K·(18-c-6)] in THF-$d_8$.

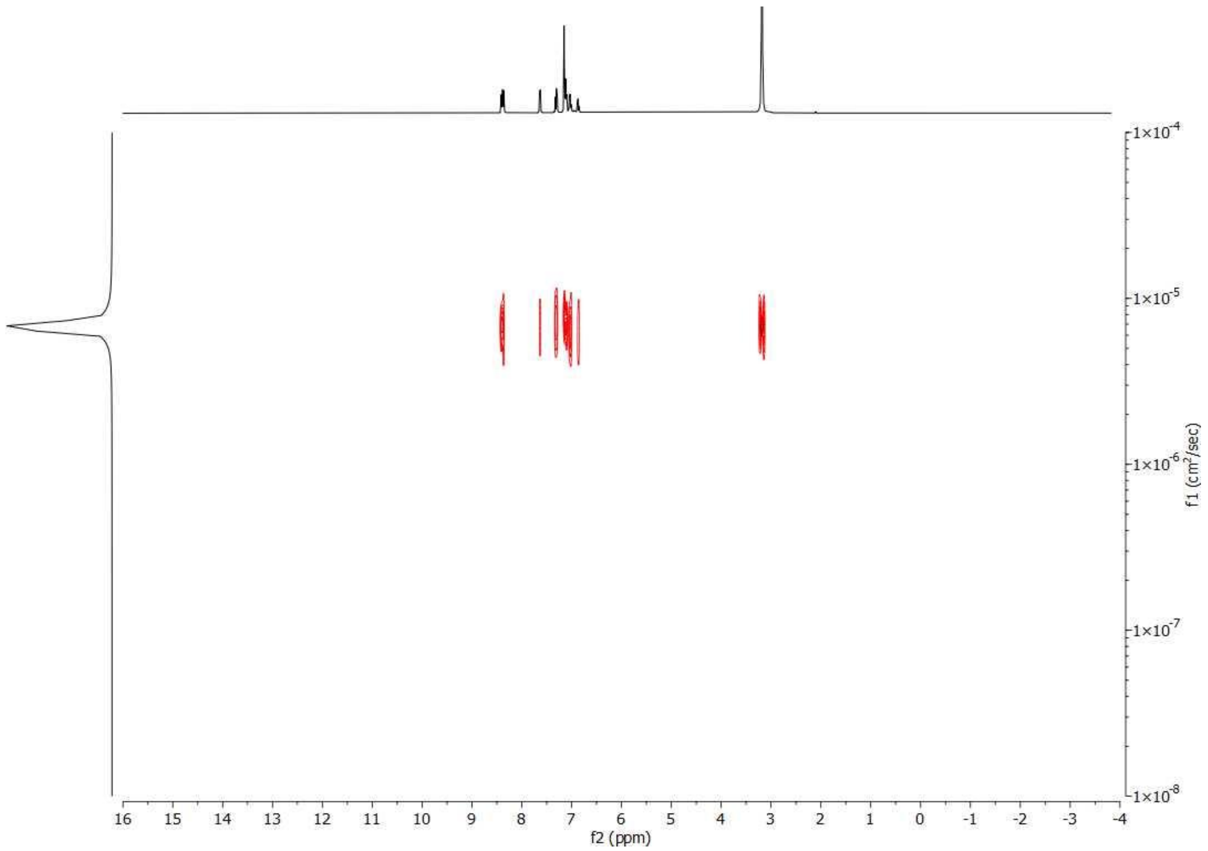


**Figure S7** $^{1}$H-DOSY NMR spectrum of compound **3a**[K·(18-c-6)] in $C_6D_6$ with chemical shifts (ppm) on the f2 axis and diffusion coefficients ($cm^2$/sec) on the f1 axis. DOSY spectrum produced via MestreNova software with Bayesian method (Resolution factor: 1; Repetitions: 0).

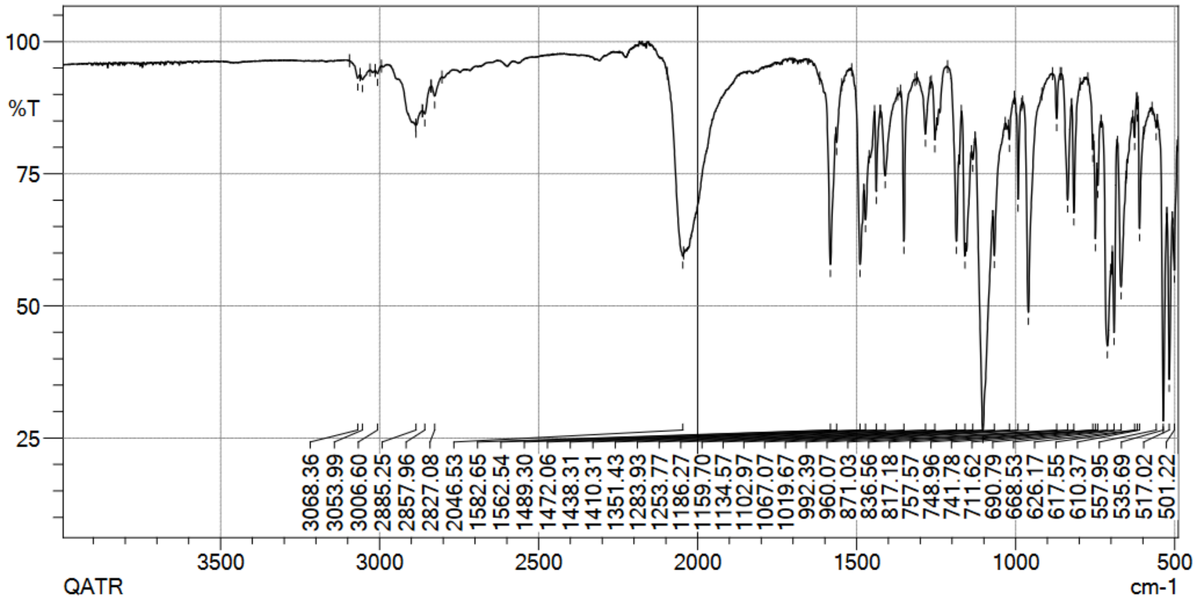


**Figure S8** IR spectrum of compound **3a**[K·(18-c-6)] (solid state).

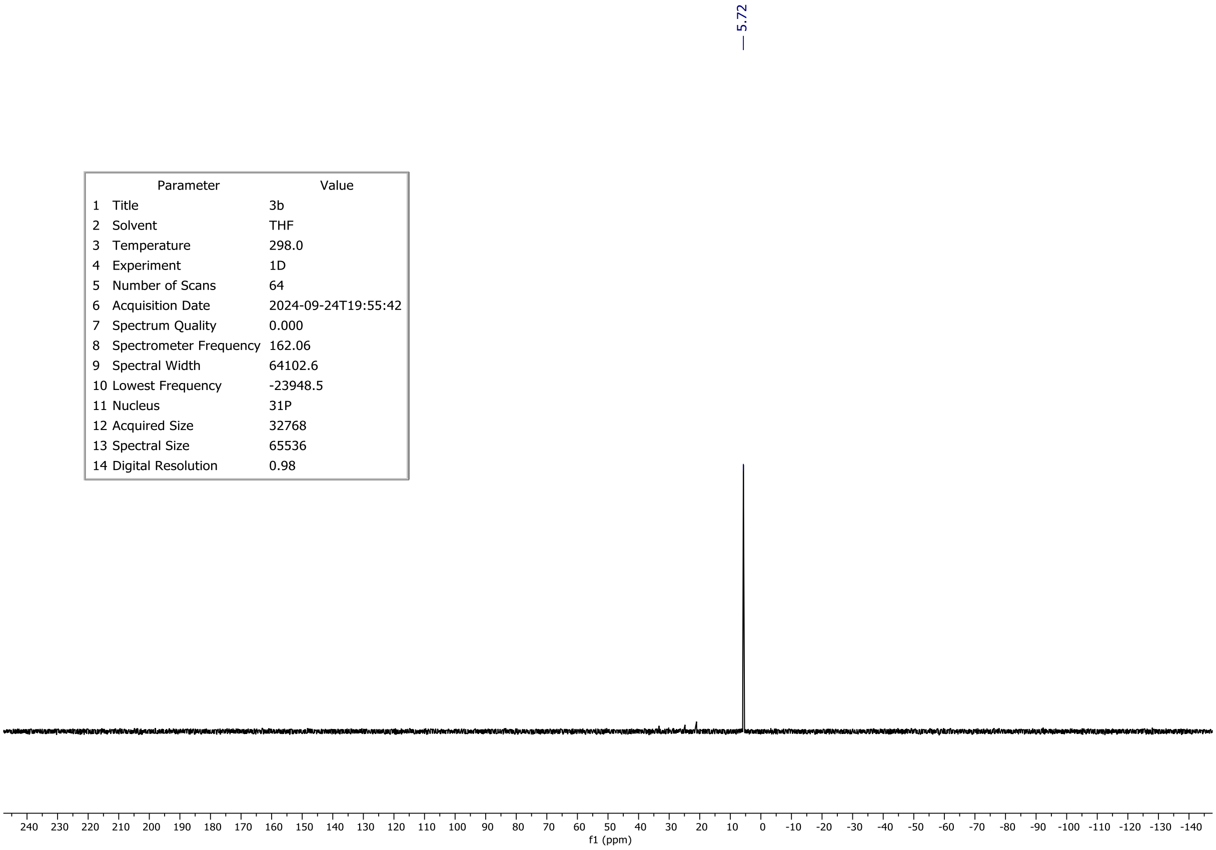


**Figure S9** $^{31}P\{^{1}H\}$ NMR spectrum of compound **3b**[K·(18-c-6)] in THF-$d_8$.

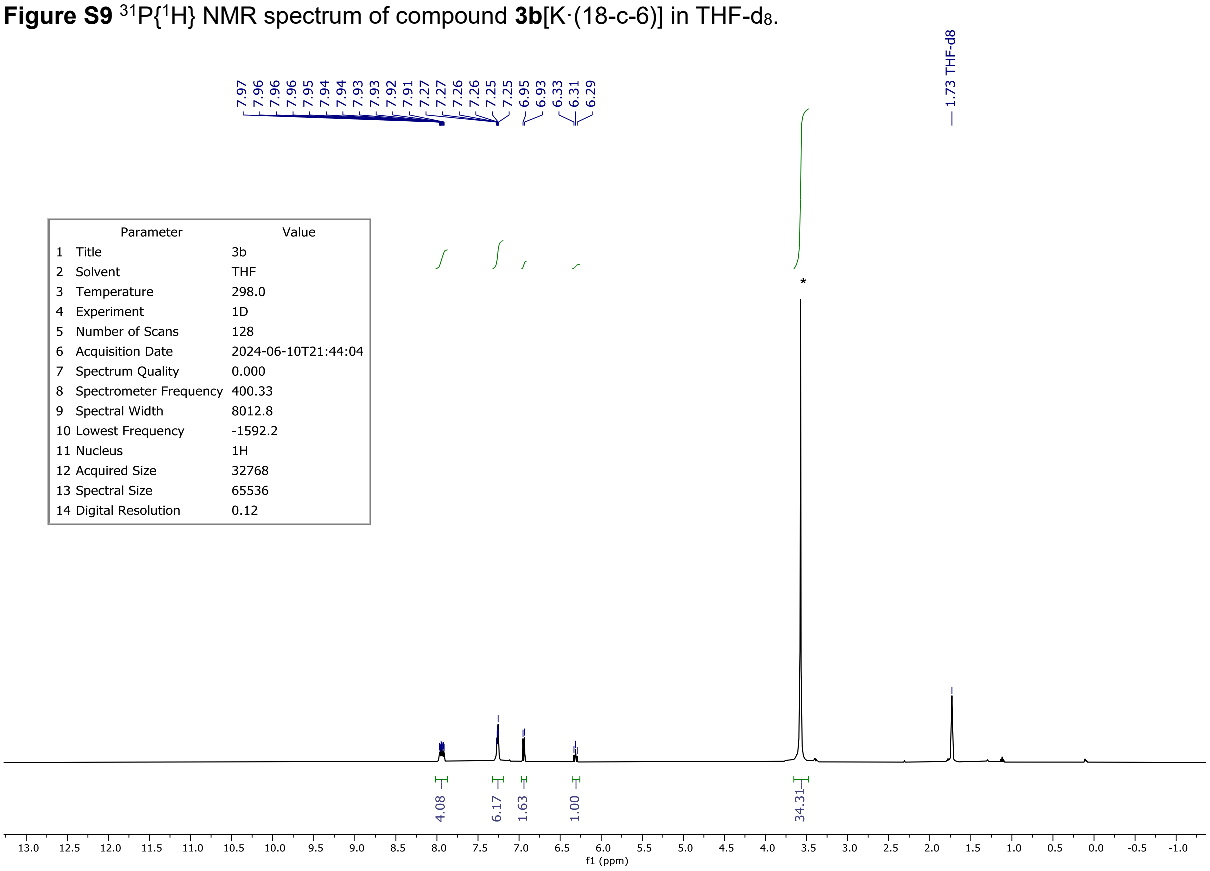


**Figure S10** $^{1}H$ NMR spectrum of compound **3b**[K·(18-c-6)] in THF-$d_8$ (* = residual THF + 18-c-6)

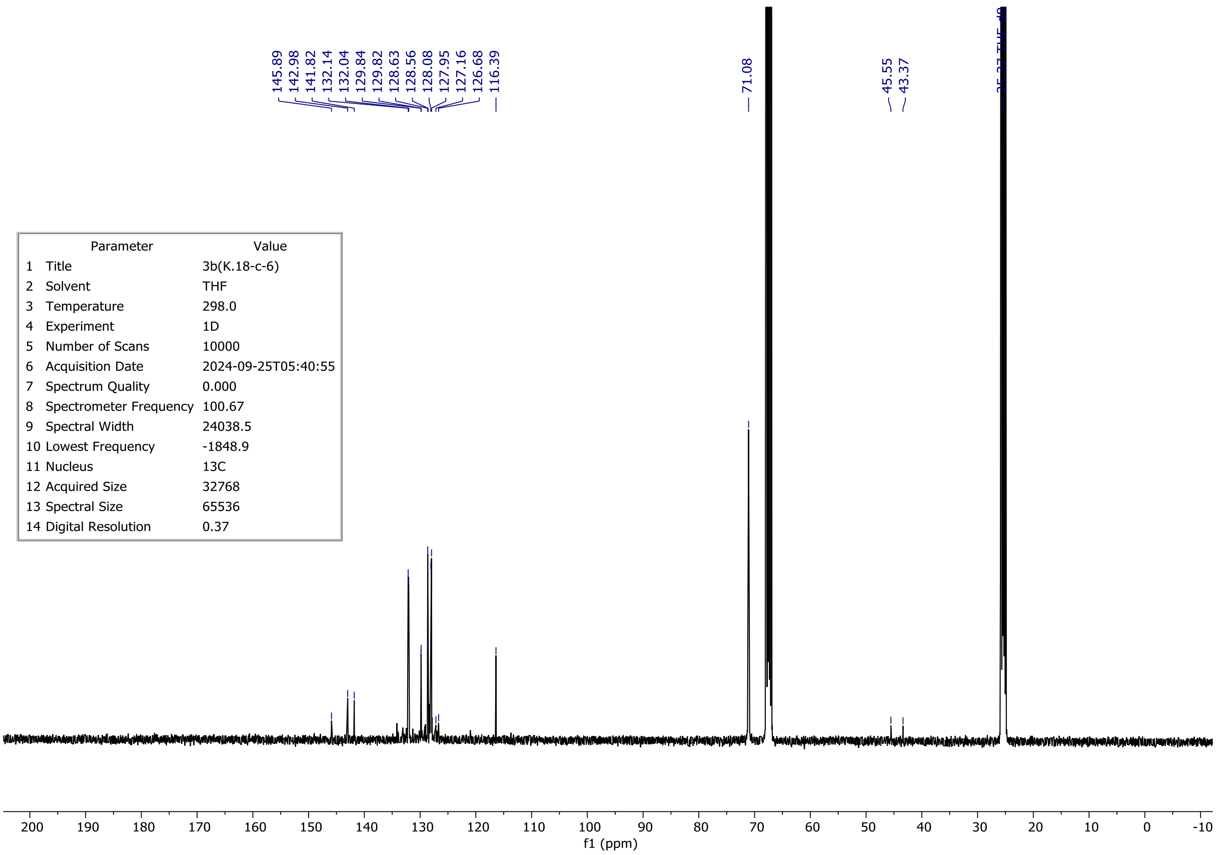


**Figure S11** $^{13}C\{^{1}H\}$ NMR spectrum of compound **3b**[K·(18-c-6)] in THF-d$_8$.

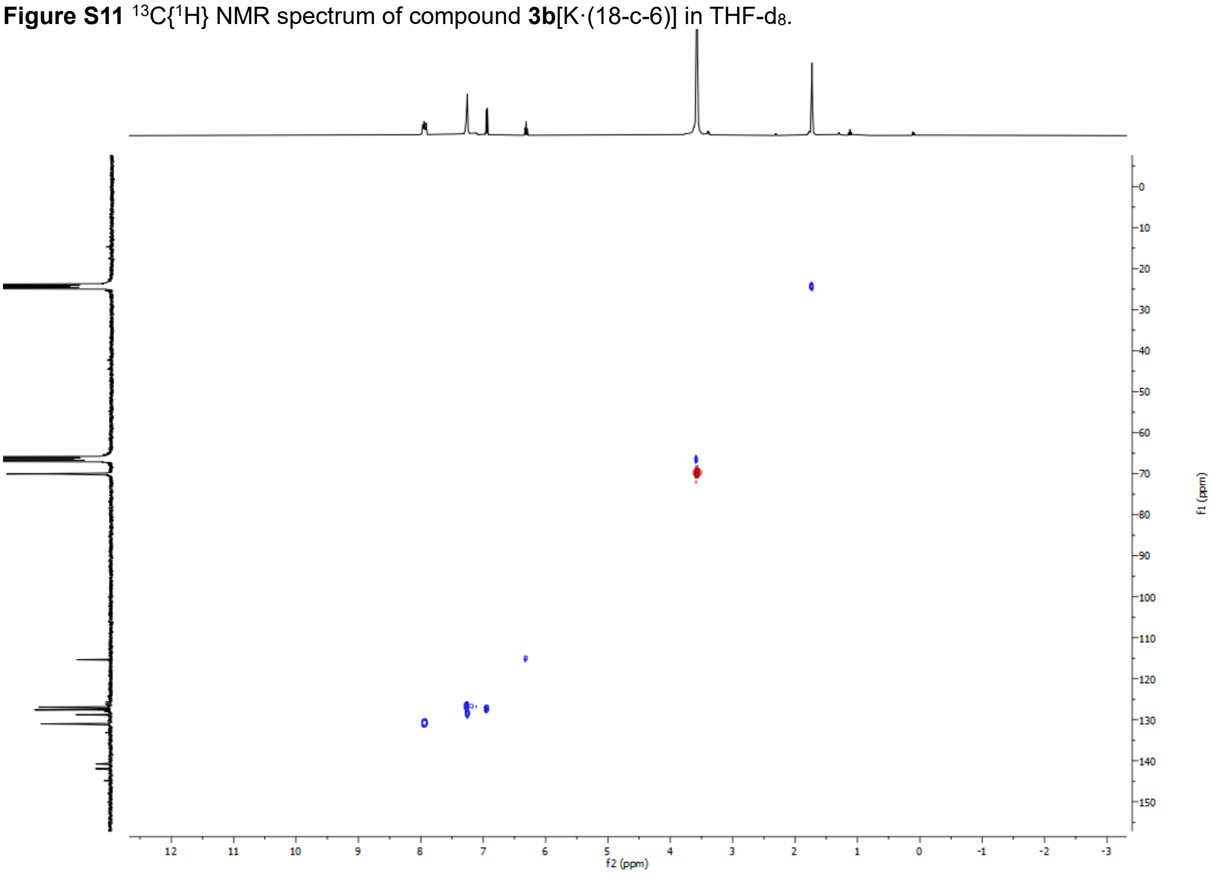


**Figure S12** $^{1}H$-$^{13}C$ HSQC NMR spectrum of compound **3b**[K·(18-c-6)] in THF-d$_8$.

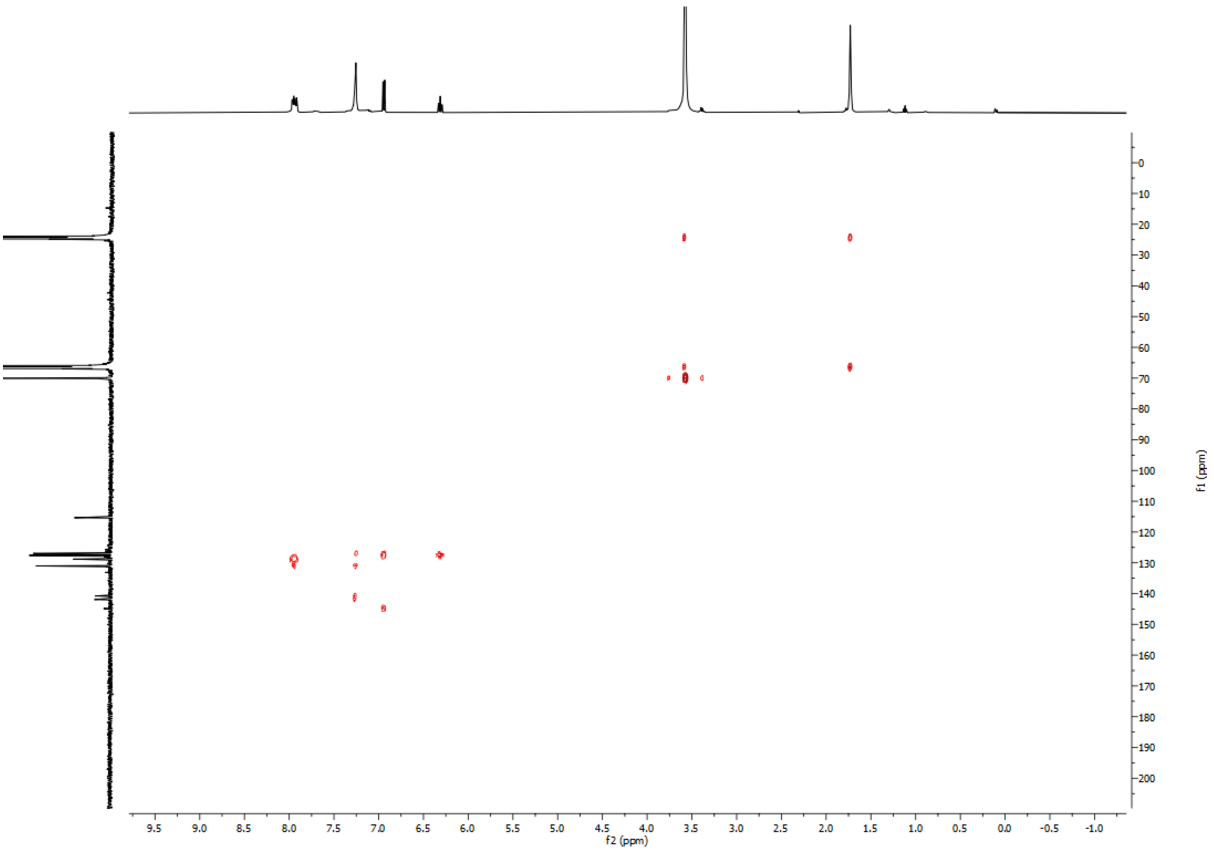


**Figure S13** $^{1}H$-$^{13}C$ HMBC NMR spectrum of compound **3b**[K·(18-c-6)] in THF-$d_8$.

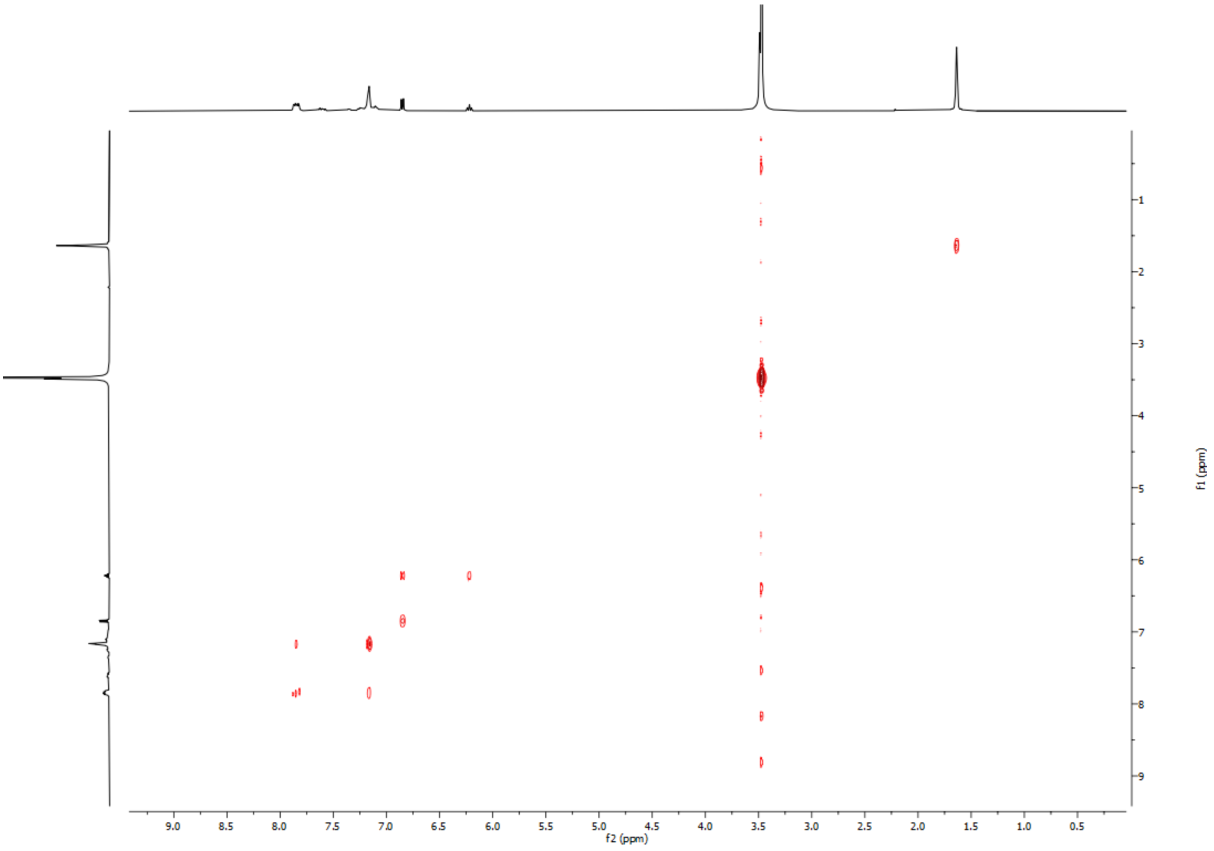


**Figure S14** $^{1}H$-$^{1}H$ COSY NMR spectrum of compound **3b**[K·(18-c-6)] in THF-$d_8$.

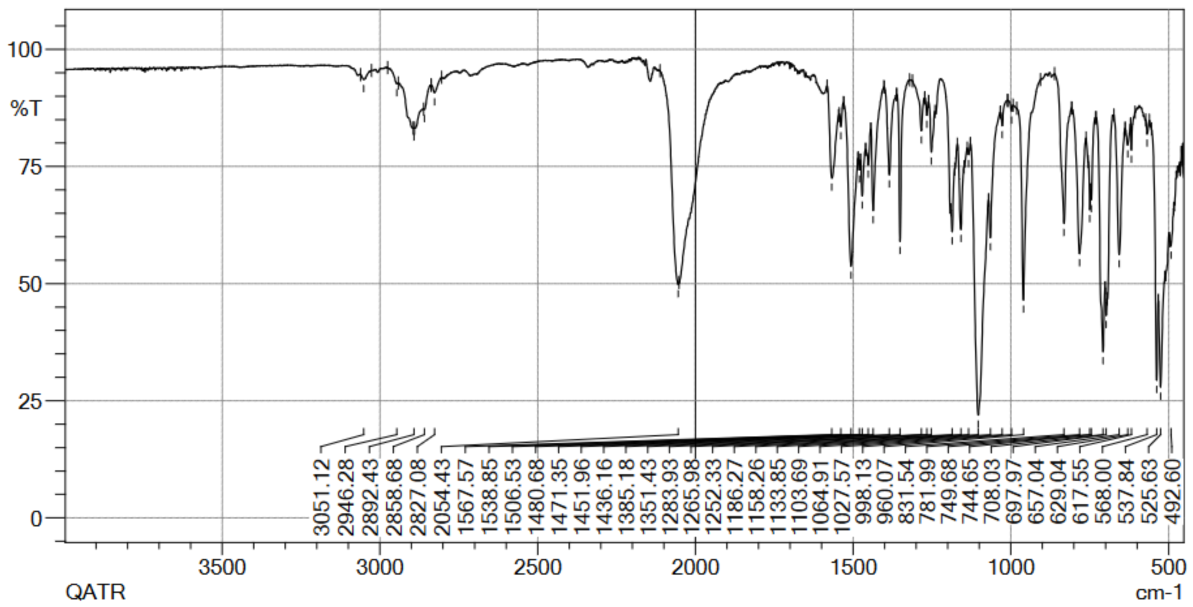


**Figure S15** IR spectrum of compound **3b**[(K·(18-c-6)] (solid state).

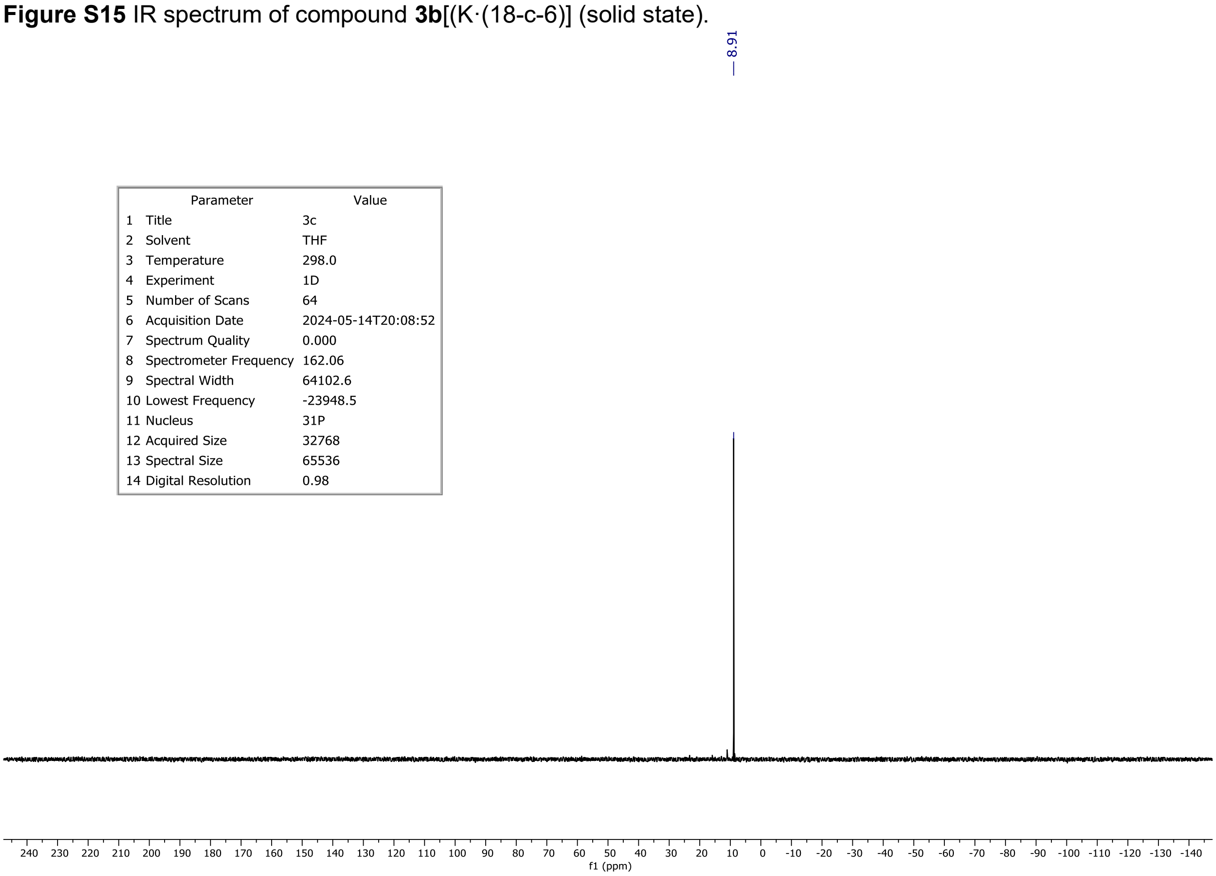


**Figure S16** $^{31}P\{^{1}H\}$ NMR spectrum of compound **3c**[K·(18-c-6)] in THF-$d_8$.

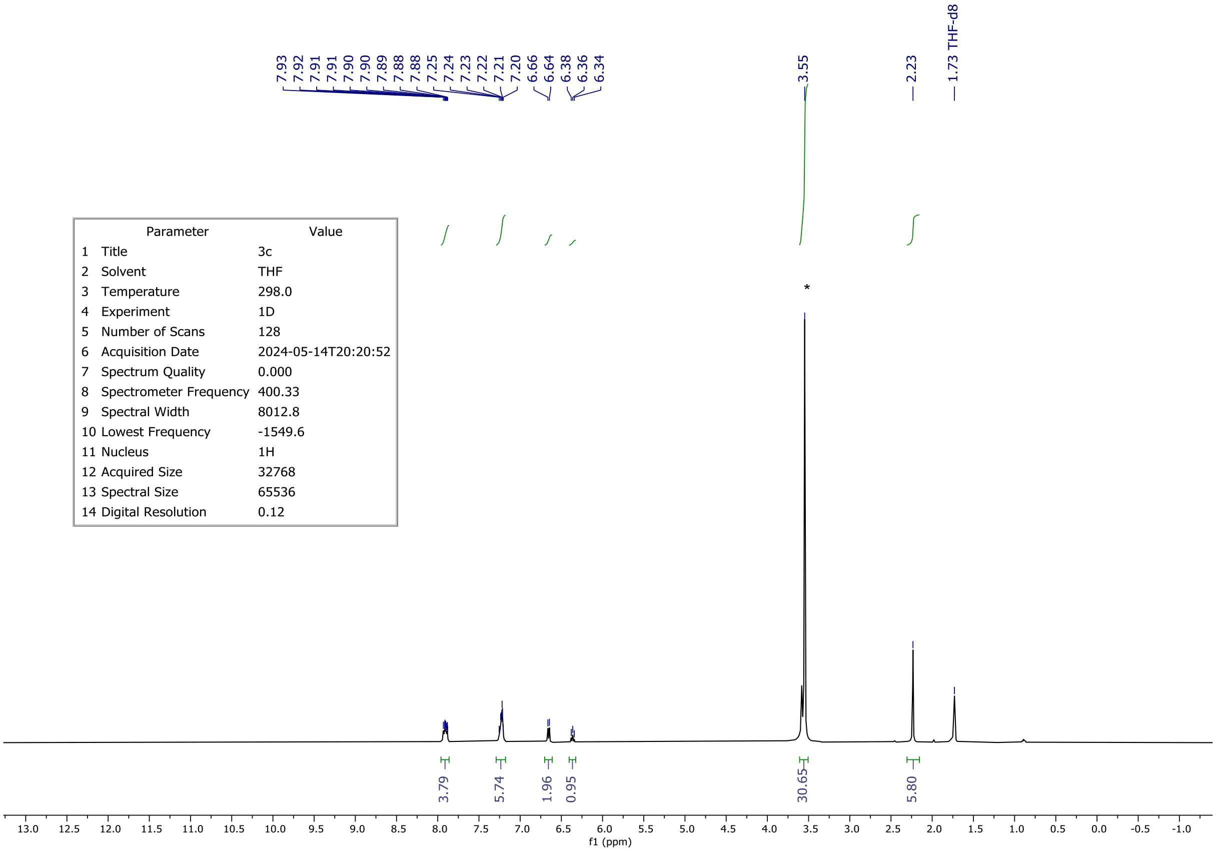


**Figure S17** $^{1}$H NMR spectrum of compound **3c**[(K·(18-c-6)] in THF-d$_8$ (* = residual THF + 18-c-6).

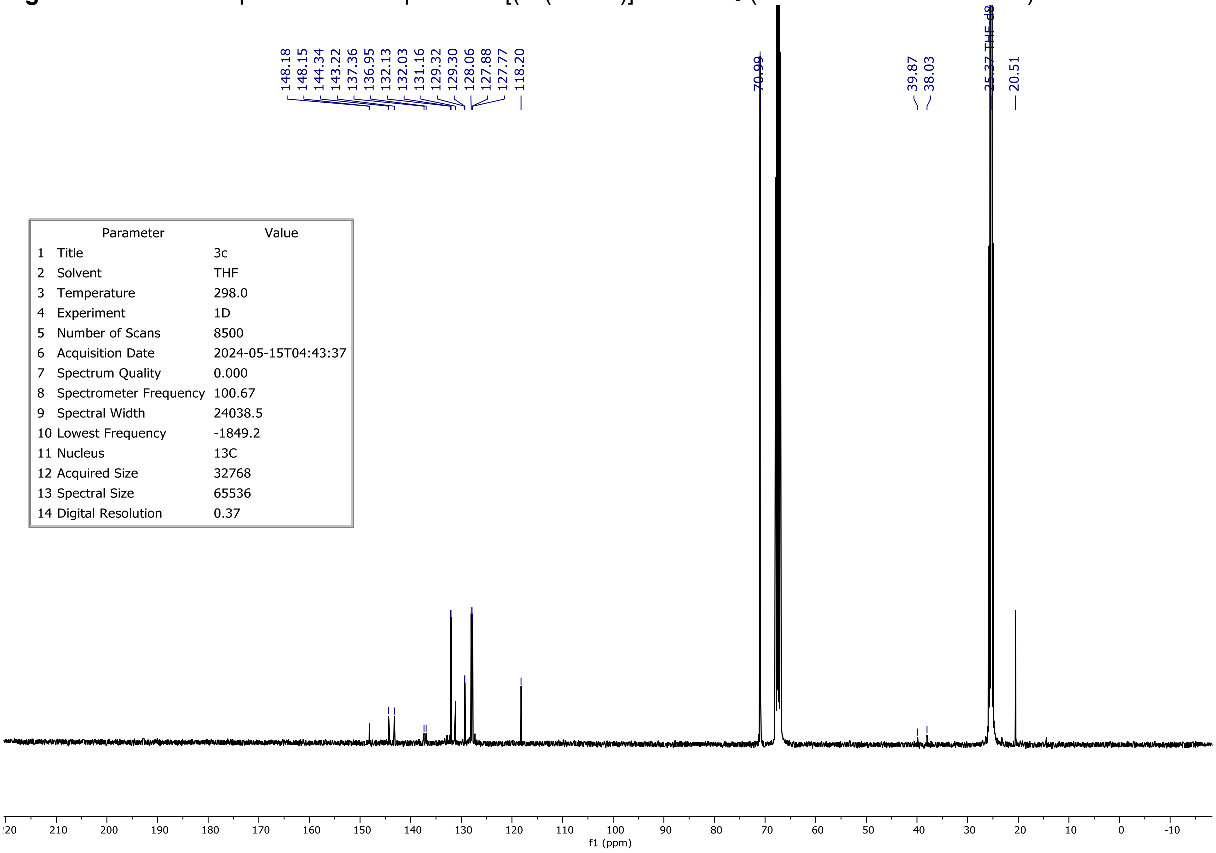


**Figure S18** $^{13}$C{$^{1}$H} NMR spectrum of compound **3c**[K·(18-c-6)] in THF-d$_8$.

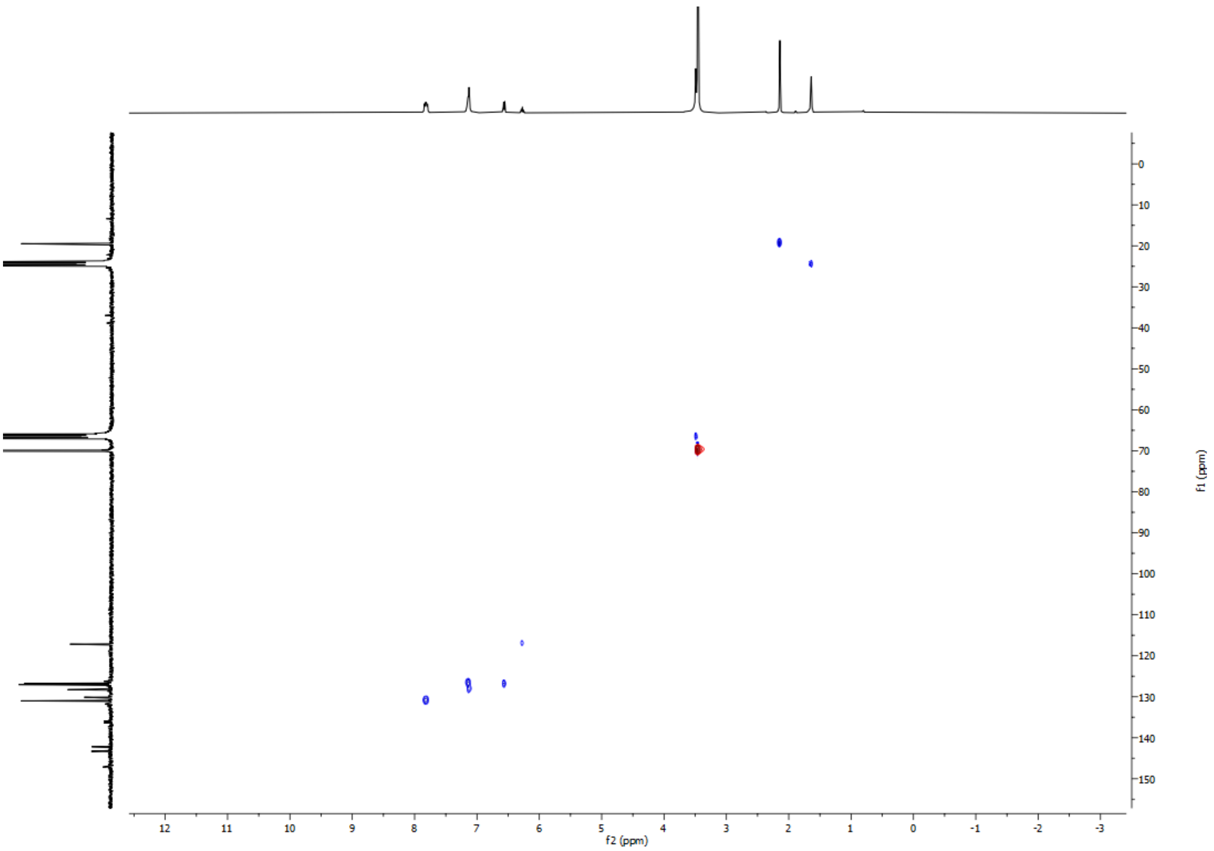


**Figure S19** $^1$H-$^{13}$C HSQC NMR spectrum of compound **3c**[K·(18-c-6)] in THF-$d_8$.

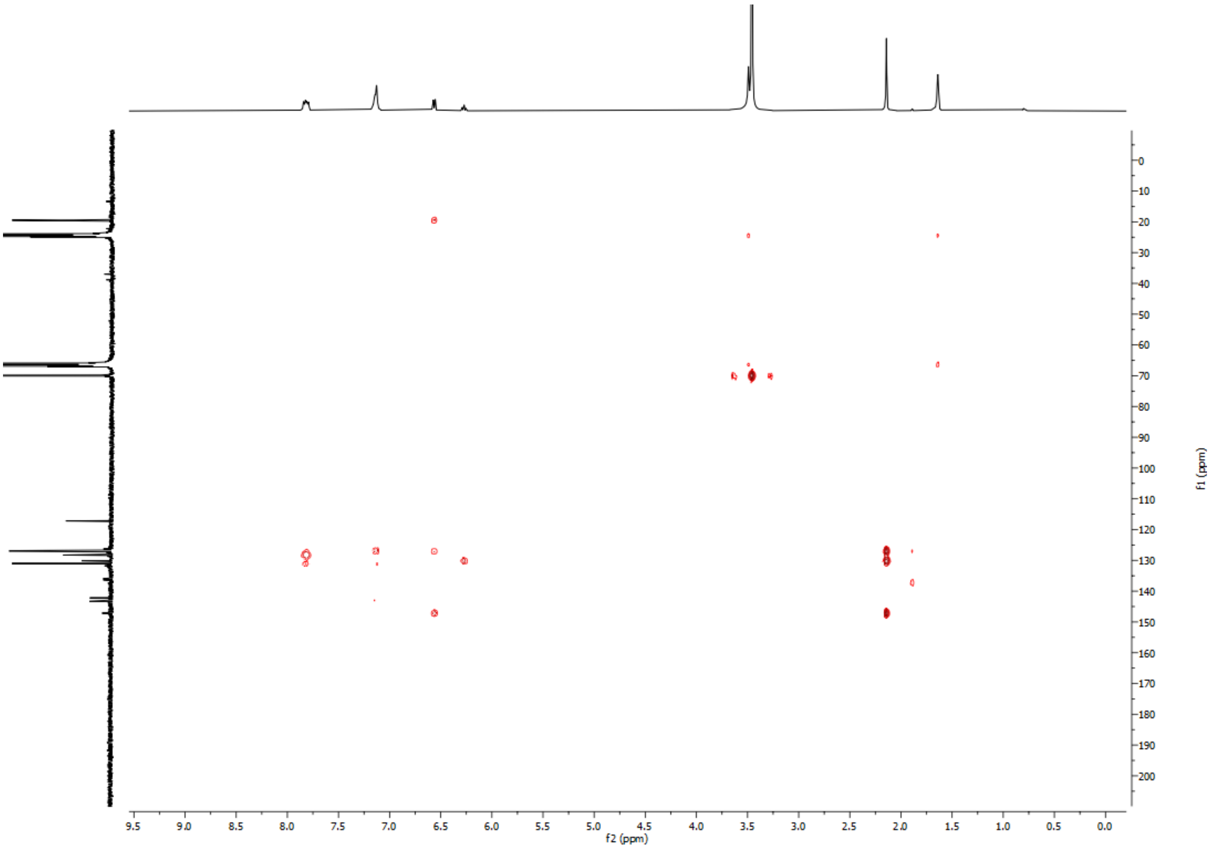


**Figure S20** $^1$H-$^{13}$C HMBC NMR spectrum of compound **3c**[K·(18-c-6)] in THF-$d_8$.

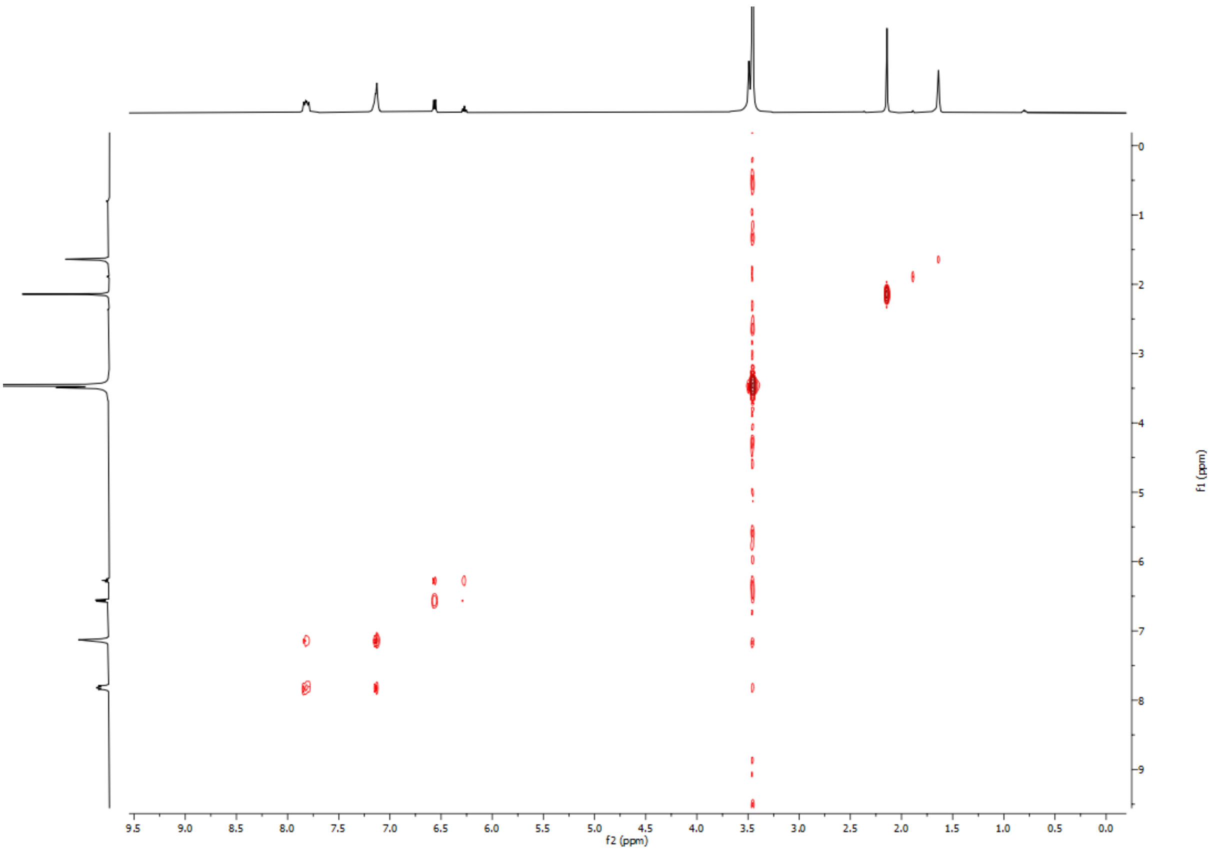

**Figure S21** $^{1}H$-$^{1}H$ COSY NMR spectrum of compound **3c**[K·(18-c-6)] in THF-$d_8$.

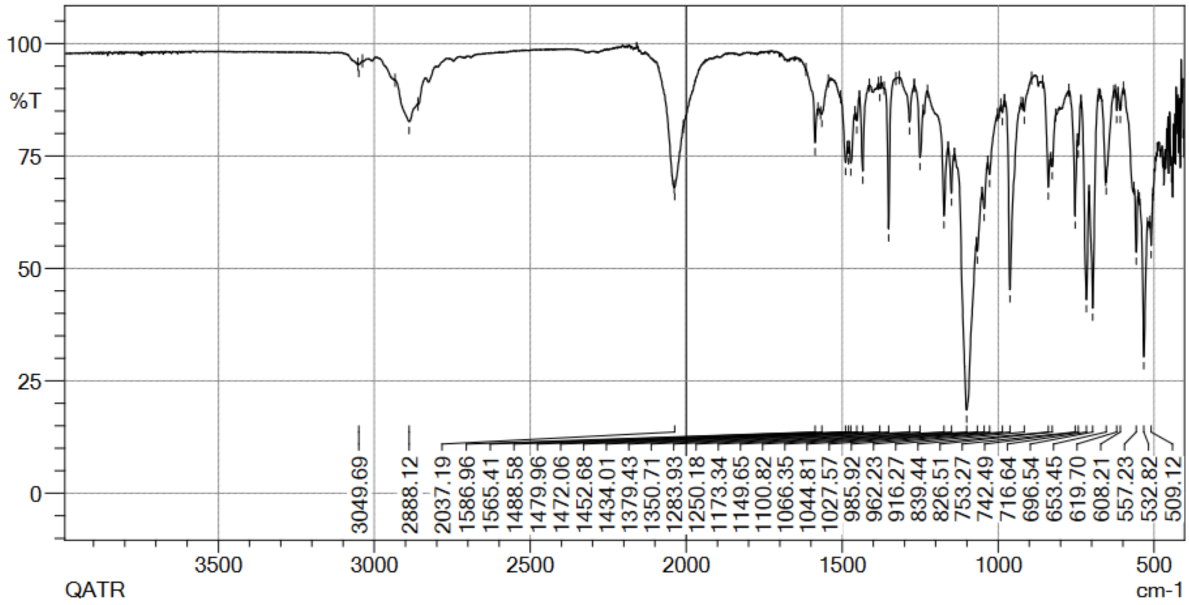

**Figure S22** IR spectrum of compound **3c**[K·(18-c-6)] (solid state).

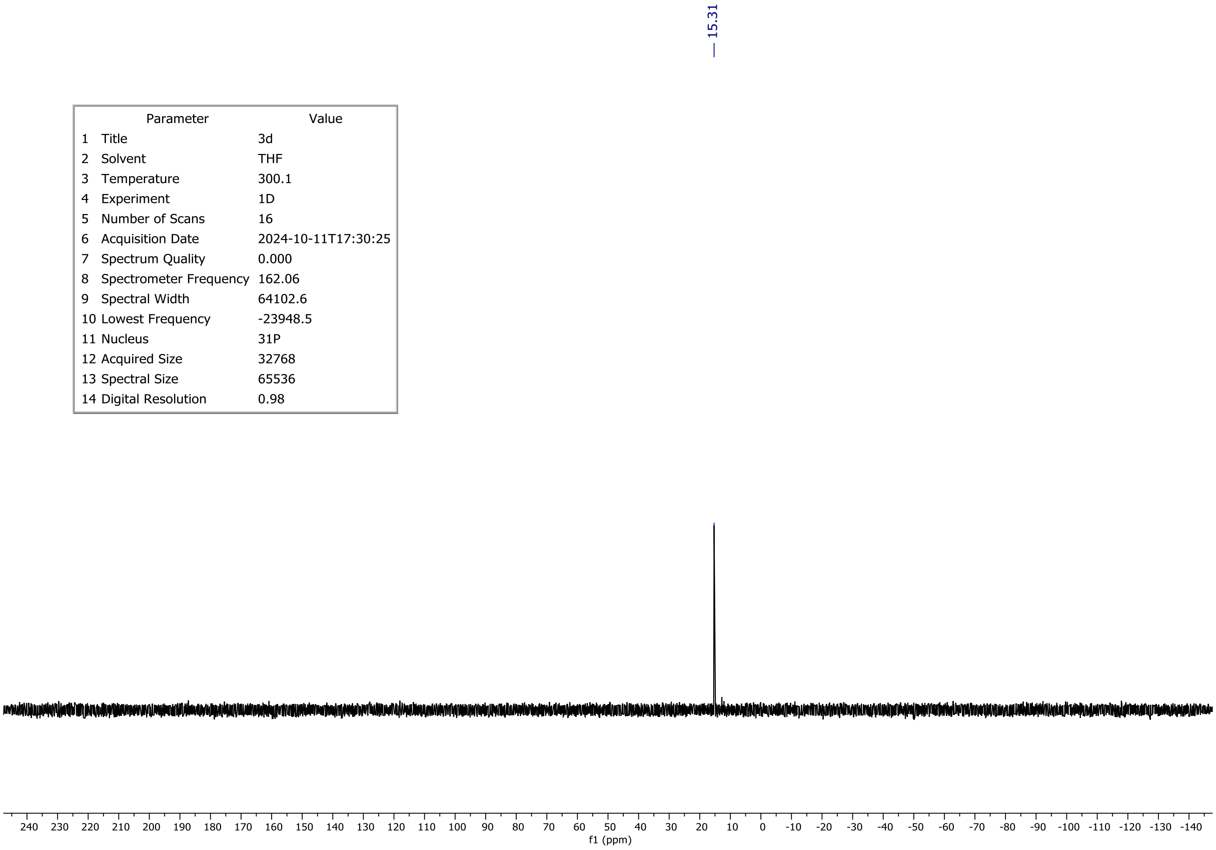


**Figure S23** $^{31}P\{^{1}H\}$ NMR spectrum of compound **3d**[K·(18-c-6)] in THF-$d_8$.

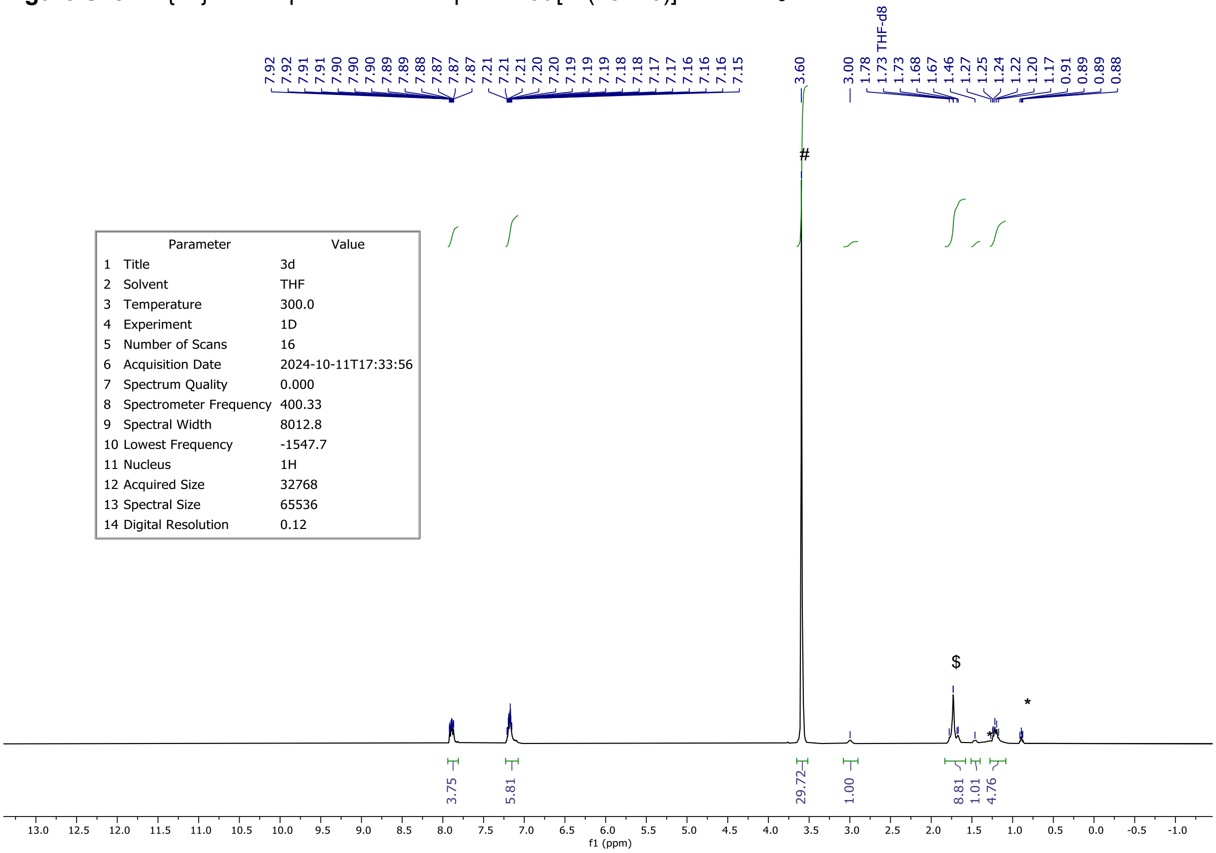


**Figure S24** $^{1}H$ NMR spectrum of compound **3d**[K·(18-c-6)] in THF-$d_8$ (* = residual pentane, # = residual THF + 18-c-6, $ = residual THF + 4H from Cy group).

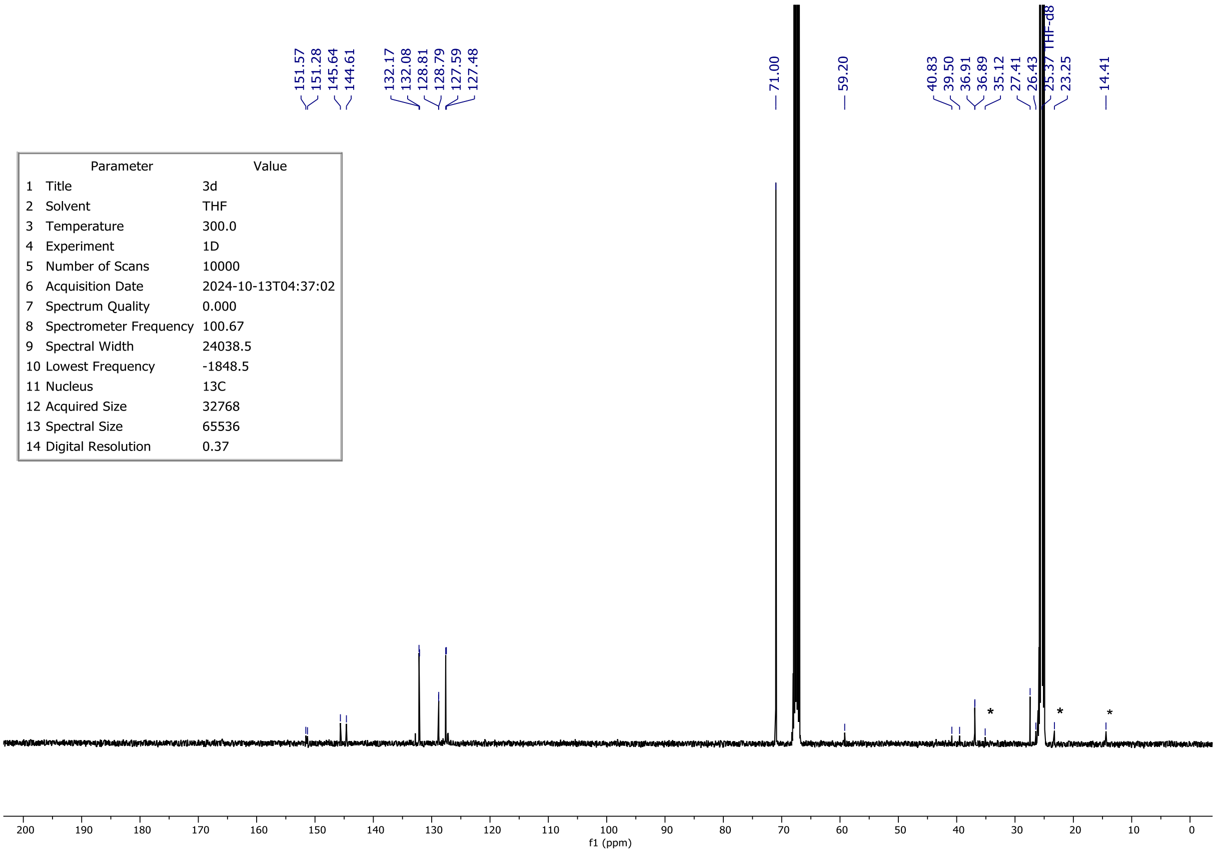


**Figure S25** $^{13}C\{^{1}H\}$ NMR spectrum of compound **3d**[K·(18-c-6)] in THF-$d_8$ (* = residual pentane).

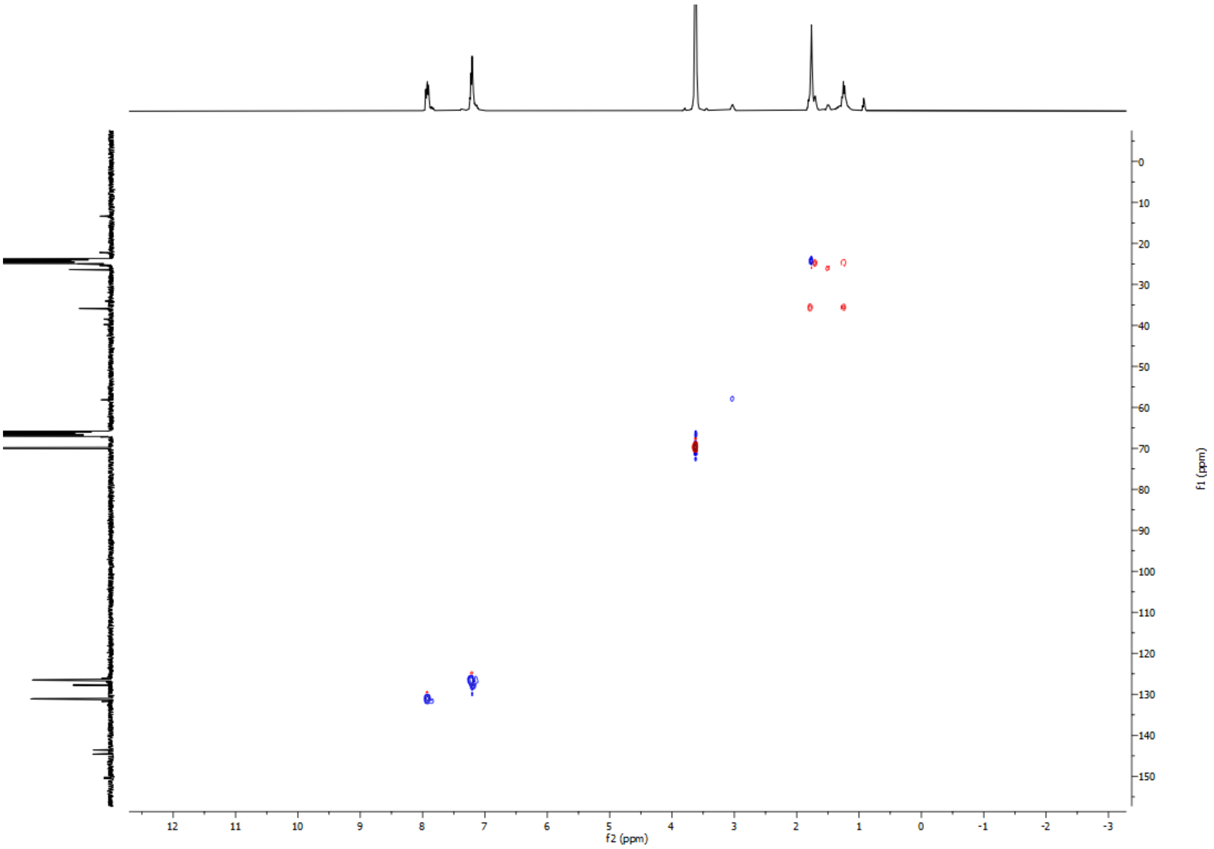


**Figure S26** $^{1}H$-$^{13}C$ HSQC NMR spectrum of compound **3d**[K·(18-c-6)] in THF-$d_8$.

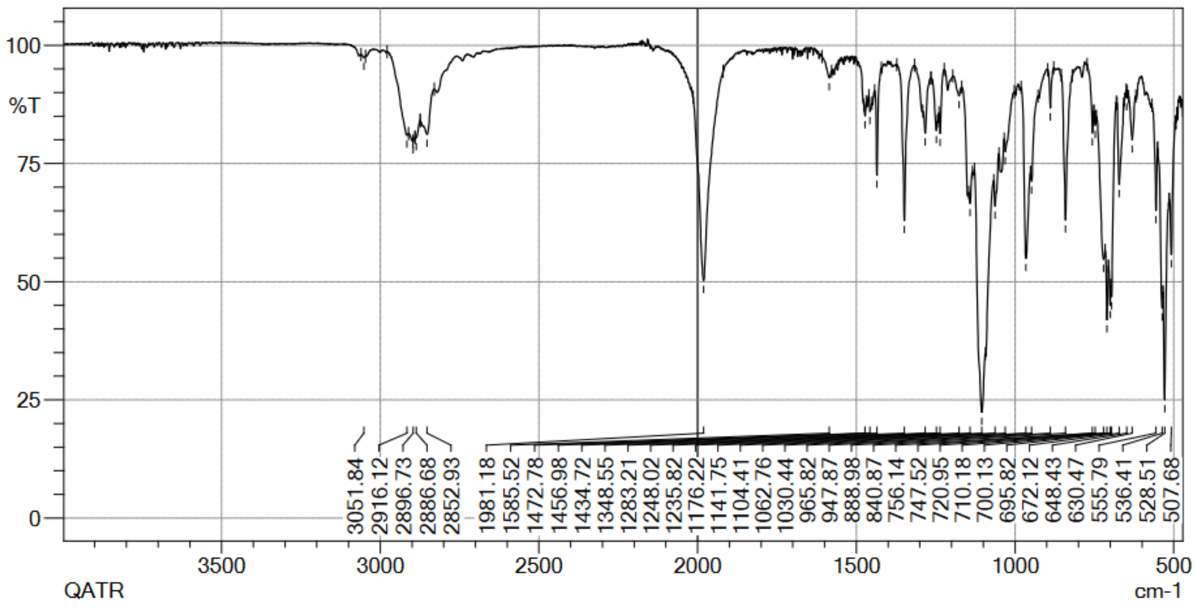


**Figure S27** IR spectrum of compound **3d**[K·(18-c-6)] (solid state).

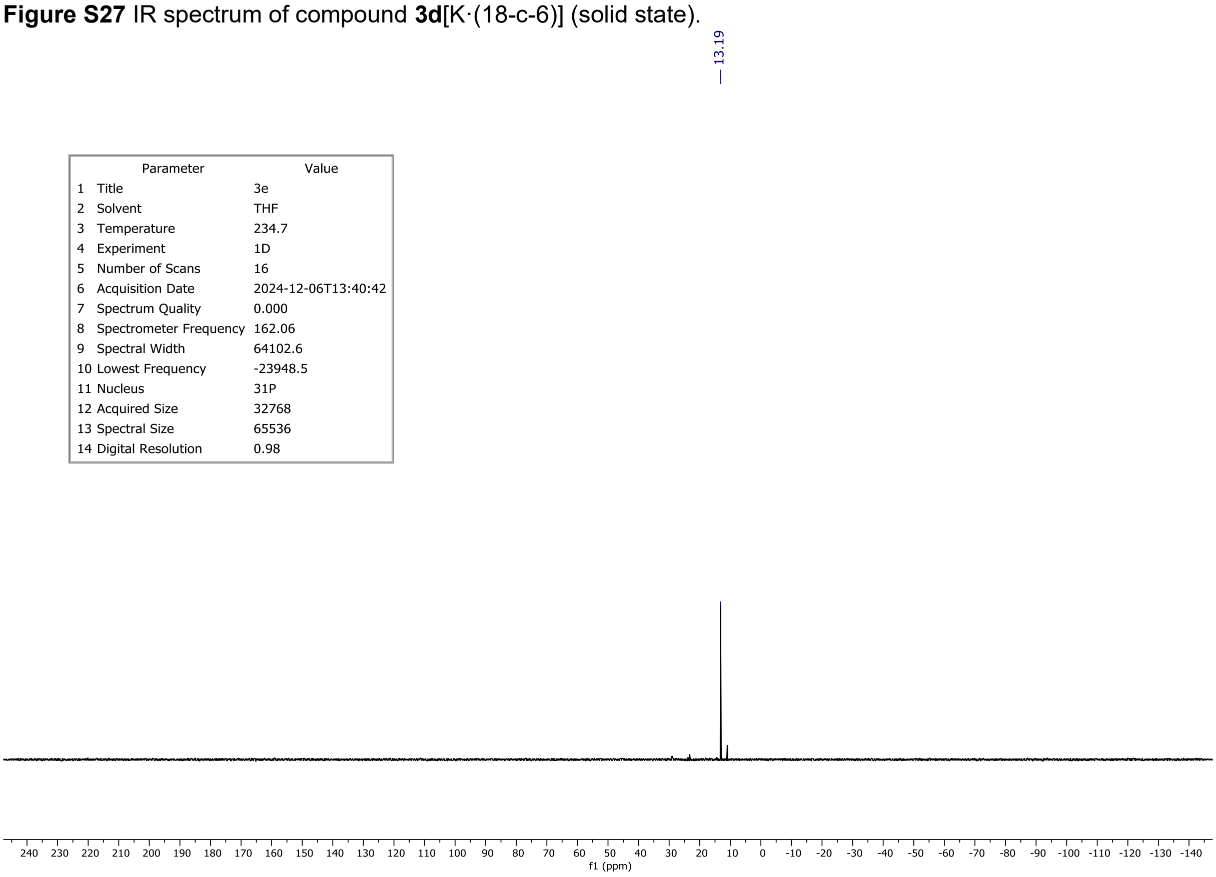


| | Parameter | Value |
|---|---|---|
| 1 | Title | 3e |
| 2 | Solvent | THF |
| 3 | Temperature | 234.7 |
| 4 | Experiment | 1D |
| 5 | Number of Scans | 16 |
| 6 | Acquisition Date | 2024-12-06T13:40:42 |
| 7 | Spectrum Quality | 0.000 |
| 8 | Spectrometer Frequency | 162.06 |
| 9 | Spectral Width | 64102.6 |
| 10 | Lowest Frequency | -23948.5 |
| 11 | Nucleus | 31P |
| 12 | Acquired Size | 32768 |
| 13 | Spectral Size | 65536 |
| 14 | Digital Resolution | 0.98 |

**Figure S28** $^{31}P\{^{1}H\}$ NMR spectrum of compound **3e**[K·(18-c-6)] + excess (18-c-6) in THF-$d_8$.

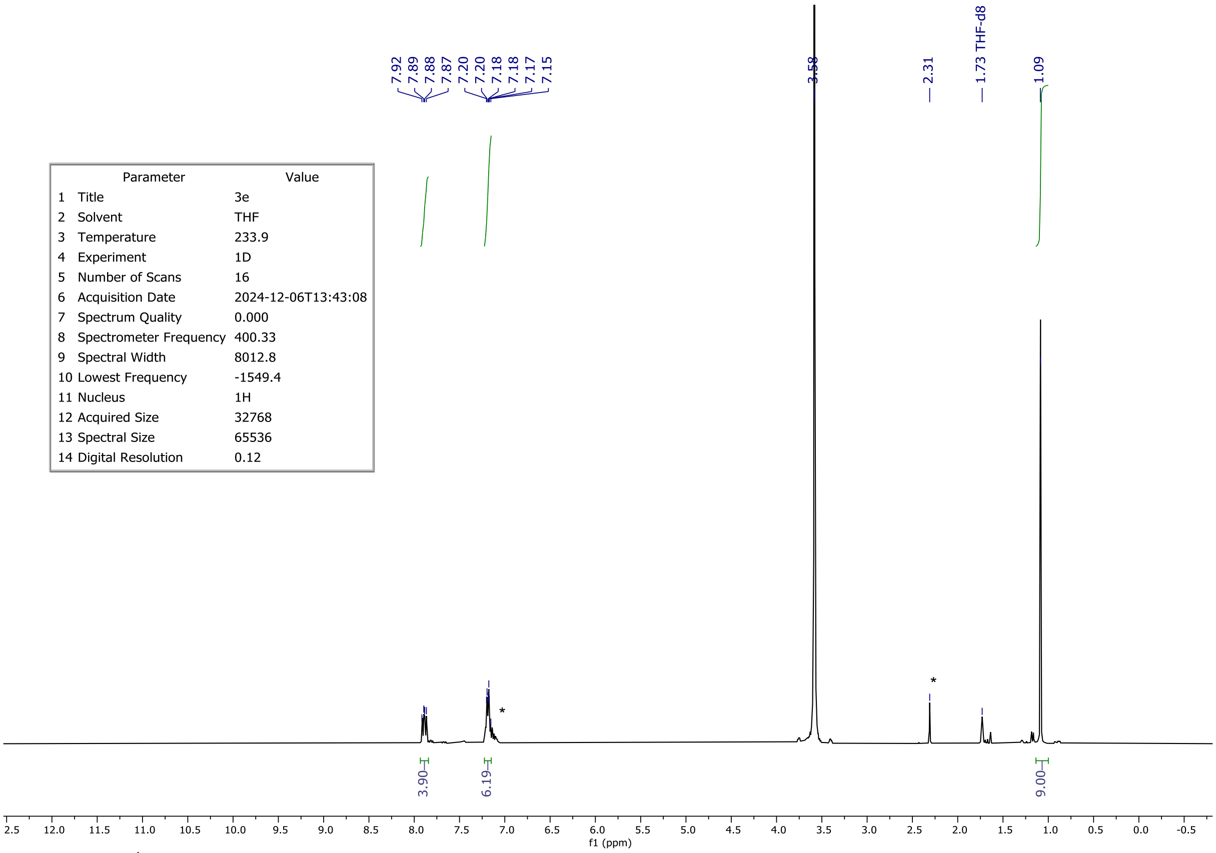


**Figure S29** $^{1}H$ NMR spectrum of compound **3e**[K·(18-c-6)] + excess (18-c-6) in THF-$d_8$ (* = residual toluene).

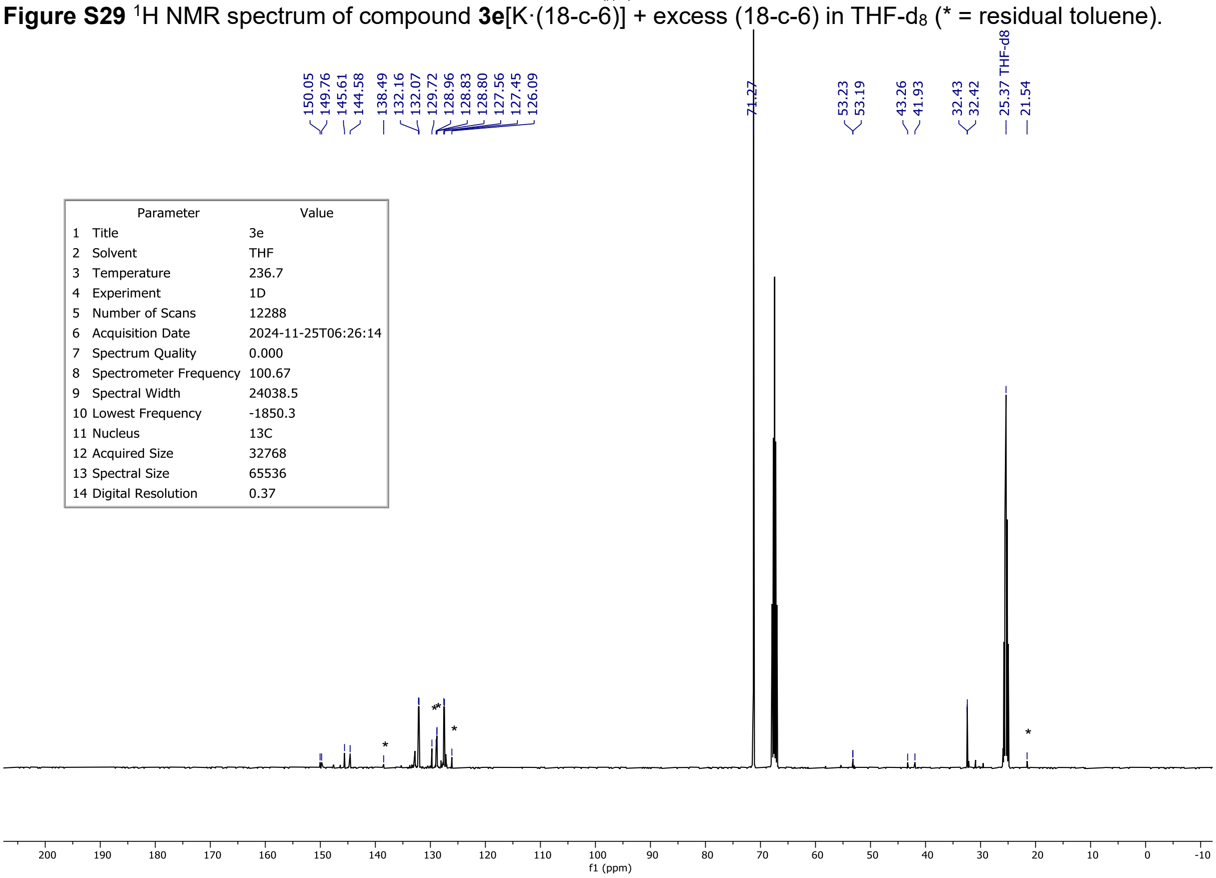


**Figure S30** $^{13}C\{^{1}H\}$ NMR spectrum of compound **3e**[K·(18-c-6)] + excess 18-c-6 in THF-$d_8$ (* = residual toluene).

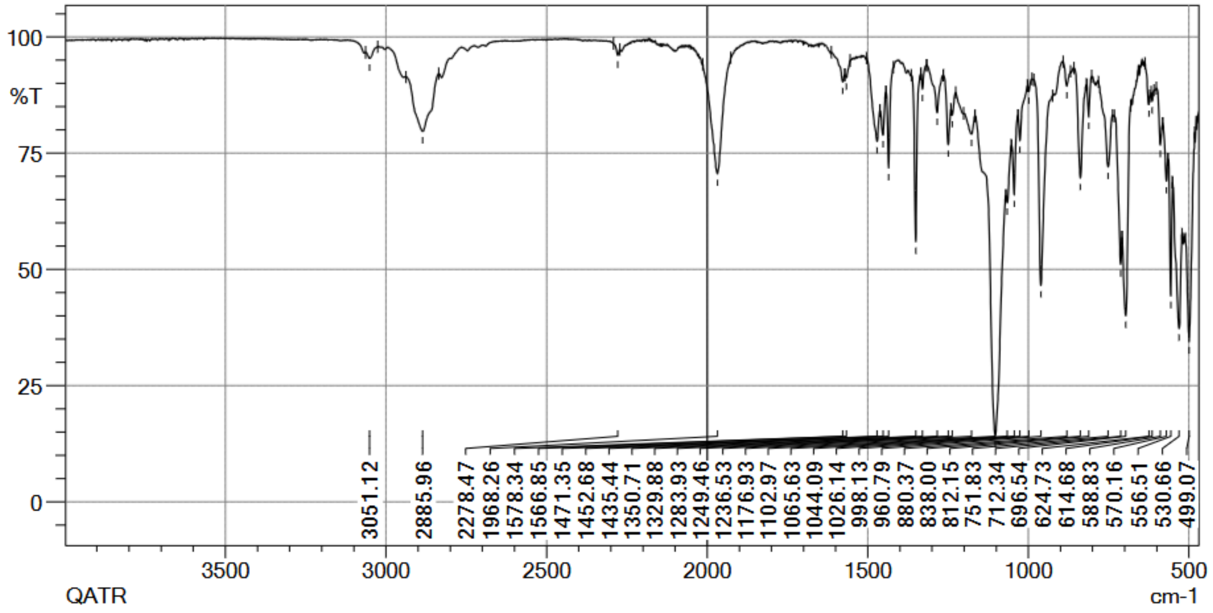


**Figure S31** IR spectrum of compound **3e**[K·(18-c-6)] (solid state).

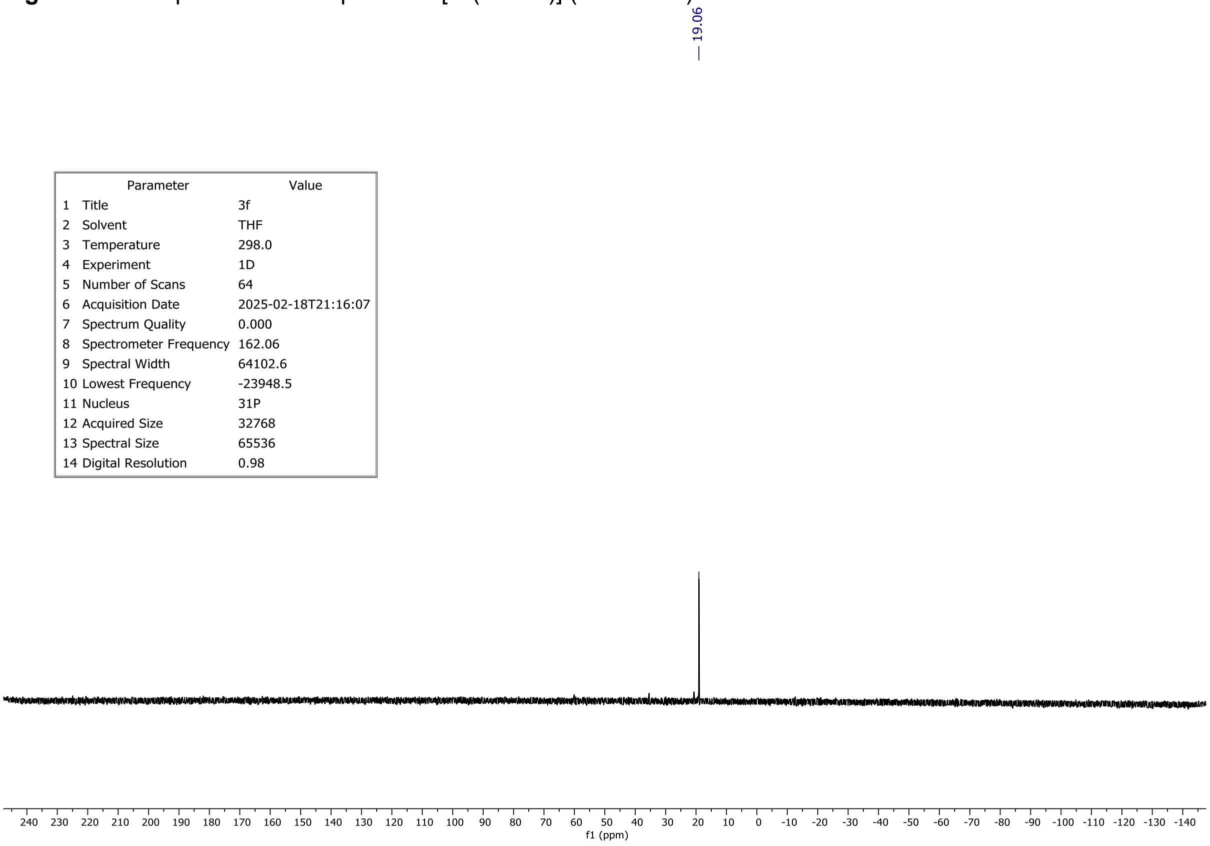


| | Parameter | Value |
|---|---|---|
| 1 | Title | 3f |
| 2 | Solvent | THF |
| 3 | Temperature | 298.0 |
| 4 | Experiment | 1D |
| 5 | Number of Scans | 64 |
| 6 | Acquisition Date | 2025-02-18T21:16:07 |
| 7 | Spectrum Quality | 0.000 |
| 8 | Spectrometer Frequency | 162.06 |
| 9 | Spectral Width | 64102.6 |
| 10 | Lowest Frequency | -23948.5 |
| 11 | Nucleus | 31P |
| 12 | Acquired Size | 32768 |
| 13 | Spectral Size | 65536 |
| 14 | Digital Resolution | 0.98 |

**Figure S32** $^{31}P\{^{1}H\}$ NMR spectrum of compound **3f**[K·(18-c-6)] in THF-$d_8$.

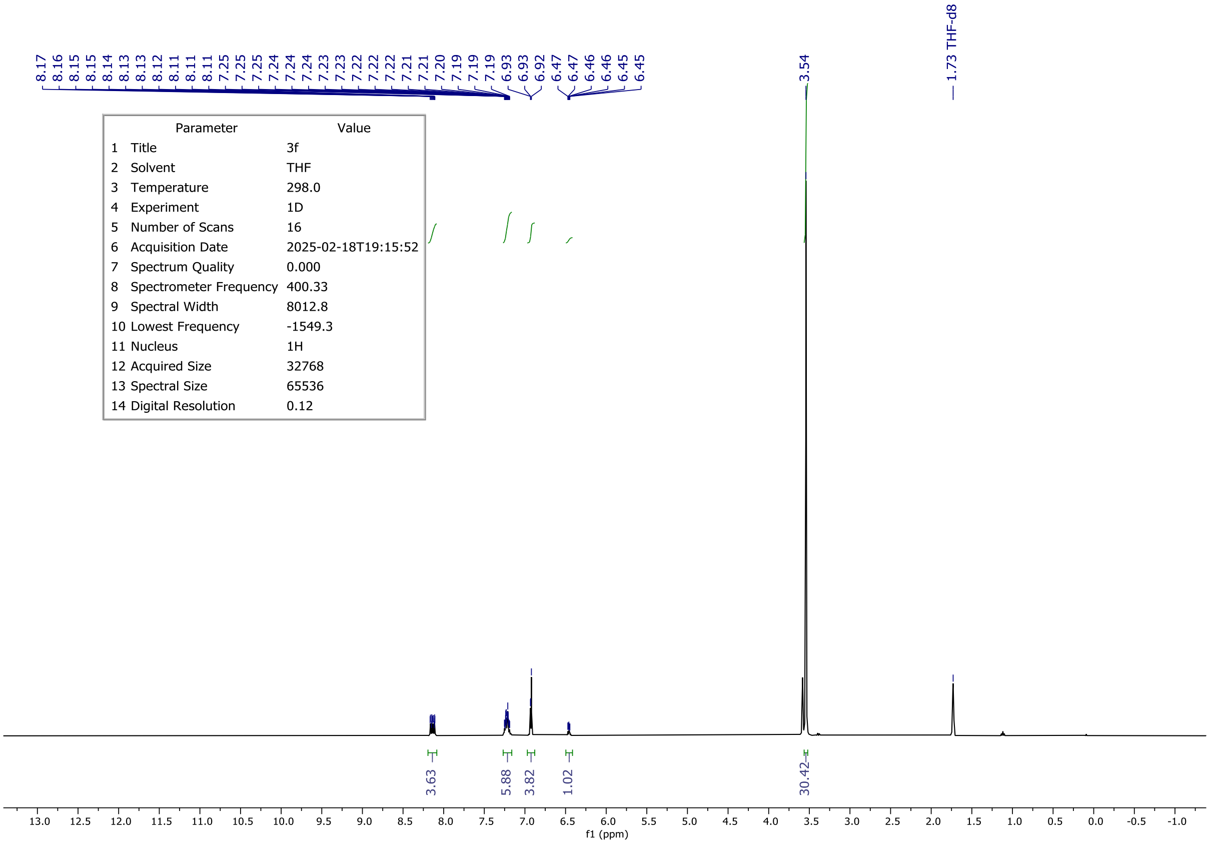


**Figure S33** $^{1}$H NMR spectrum of compound **3f**[K·(18-c-6)]in THF-d$_8$.

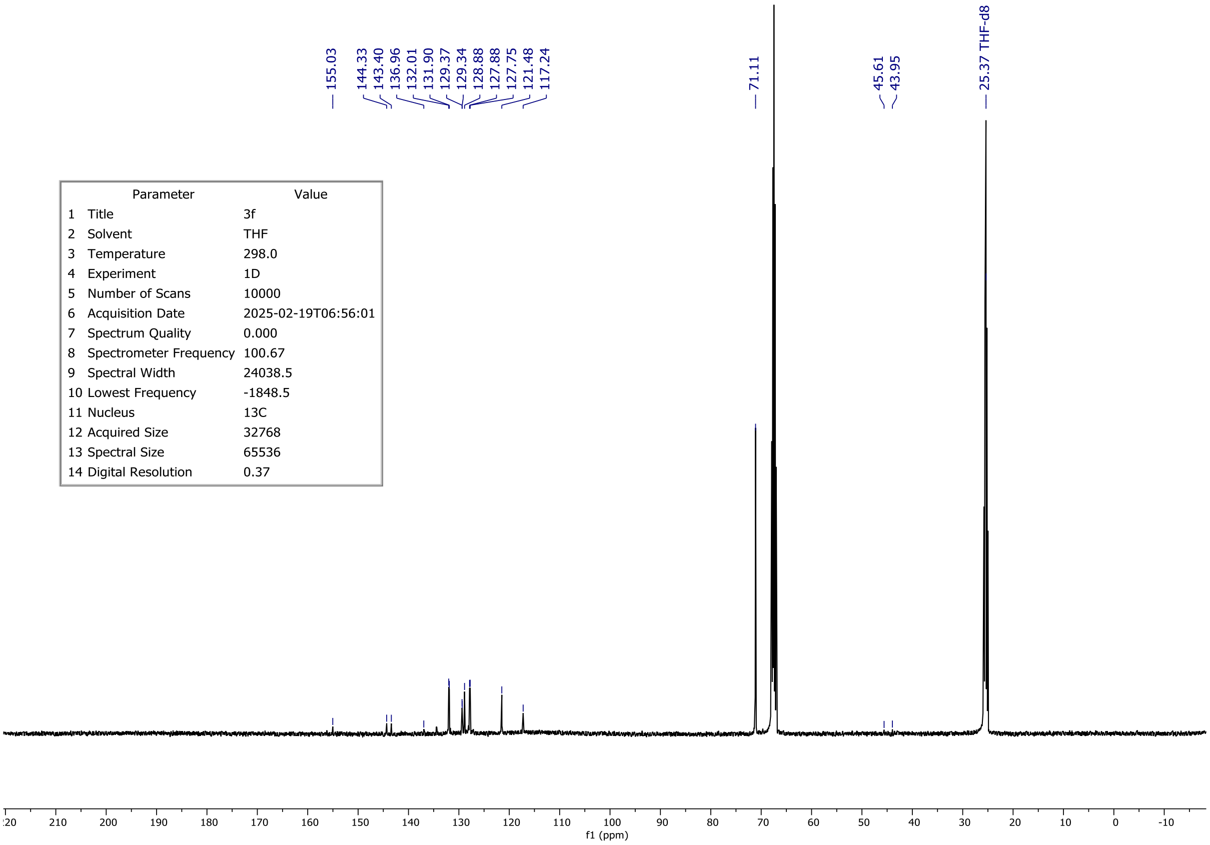


**Figure S34** $^{13}$C{$^{1}$H} NMR spectrum of compound **3f**[K·(18-c-6)] in THF-d$_8$.

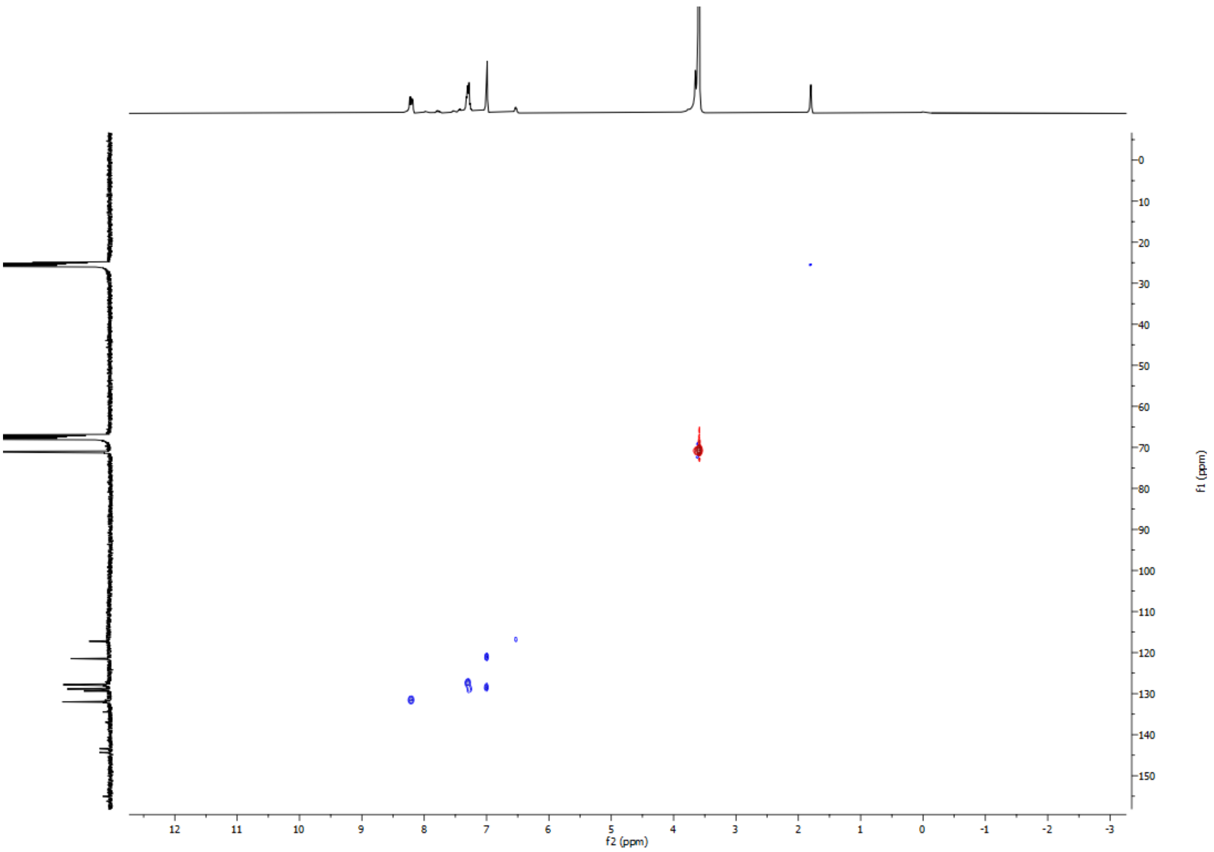


**Figure S35** $^{1}H$-$^{13}C$ HSQC NMR spectrum of compound **3f**[K·(18-c-6)] in THF-$d_8$.

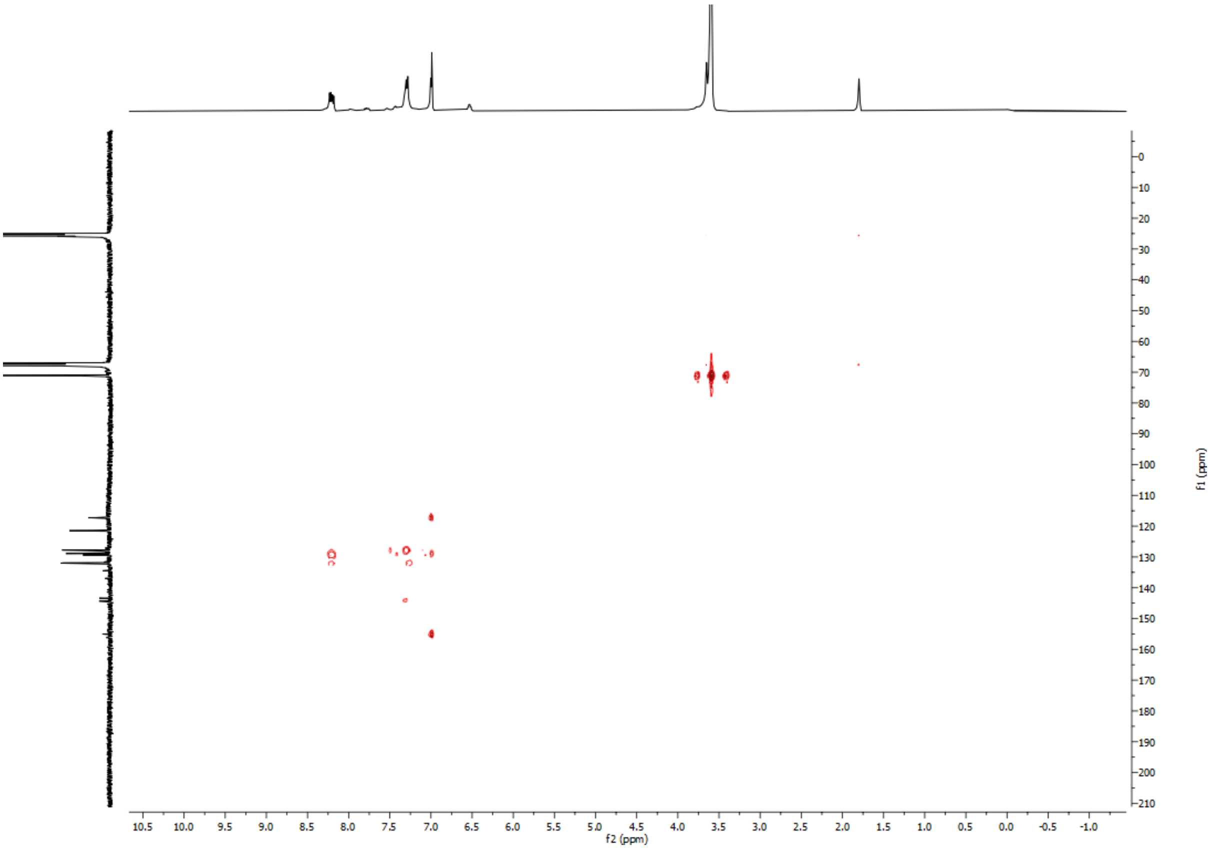


**Figure S36** $^{1}H$-$^{13}C$ HMBC NMR spectrum of compound **3f**[K·(18-c-6)] in THF-$d_8$.

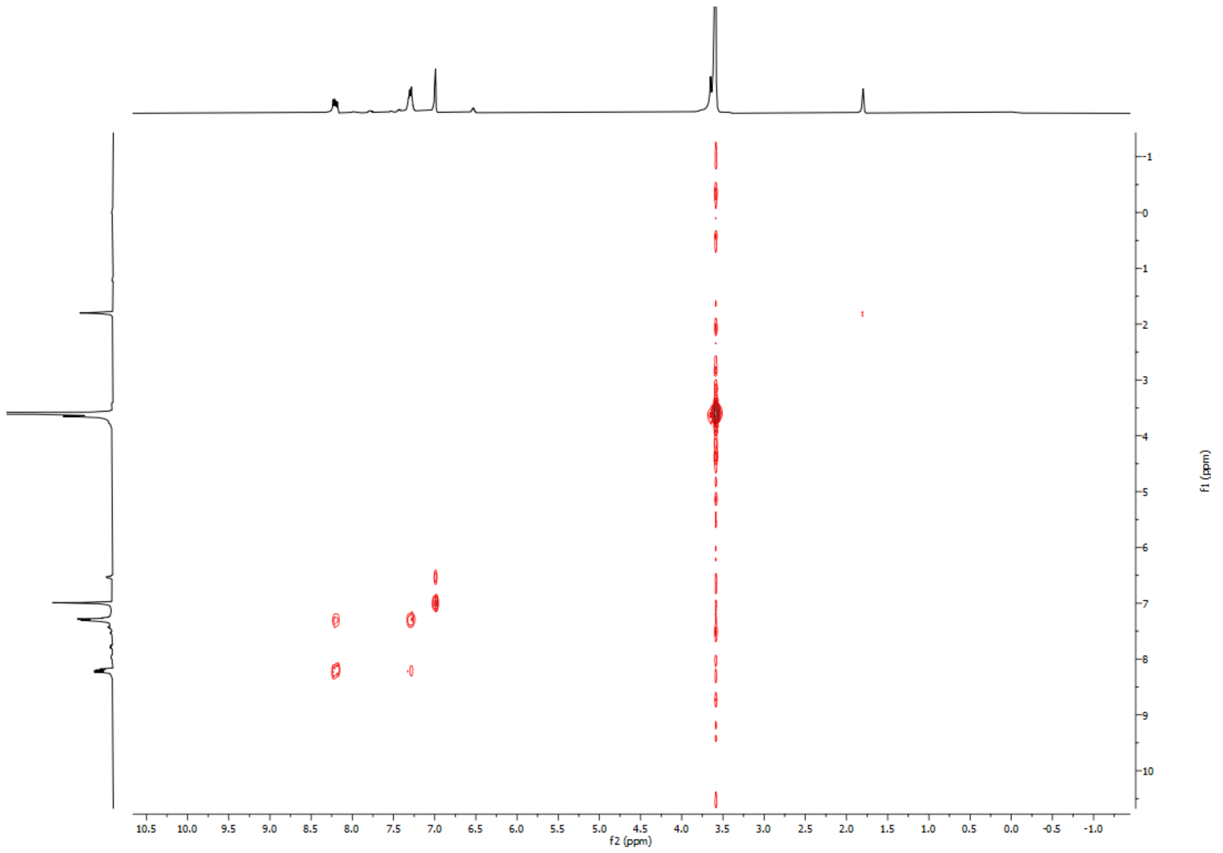


**Figure S37** $^{1}H$-$^{1}H$ COSY NMR spectrum of compound in **3f**[K·(18-c-6)] THF-$d_8$.

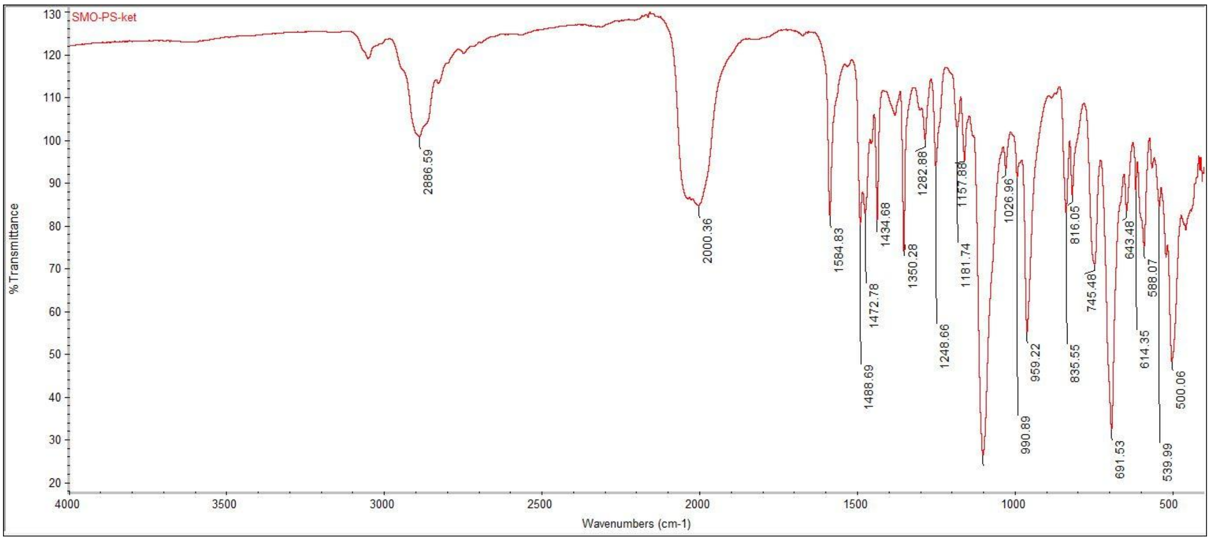


**Figure S38** IR spectrum of compound **3f**[K·(18-c-6)] (solid state).

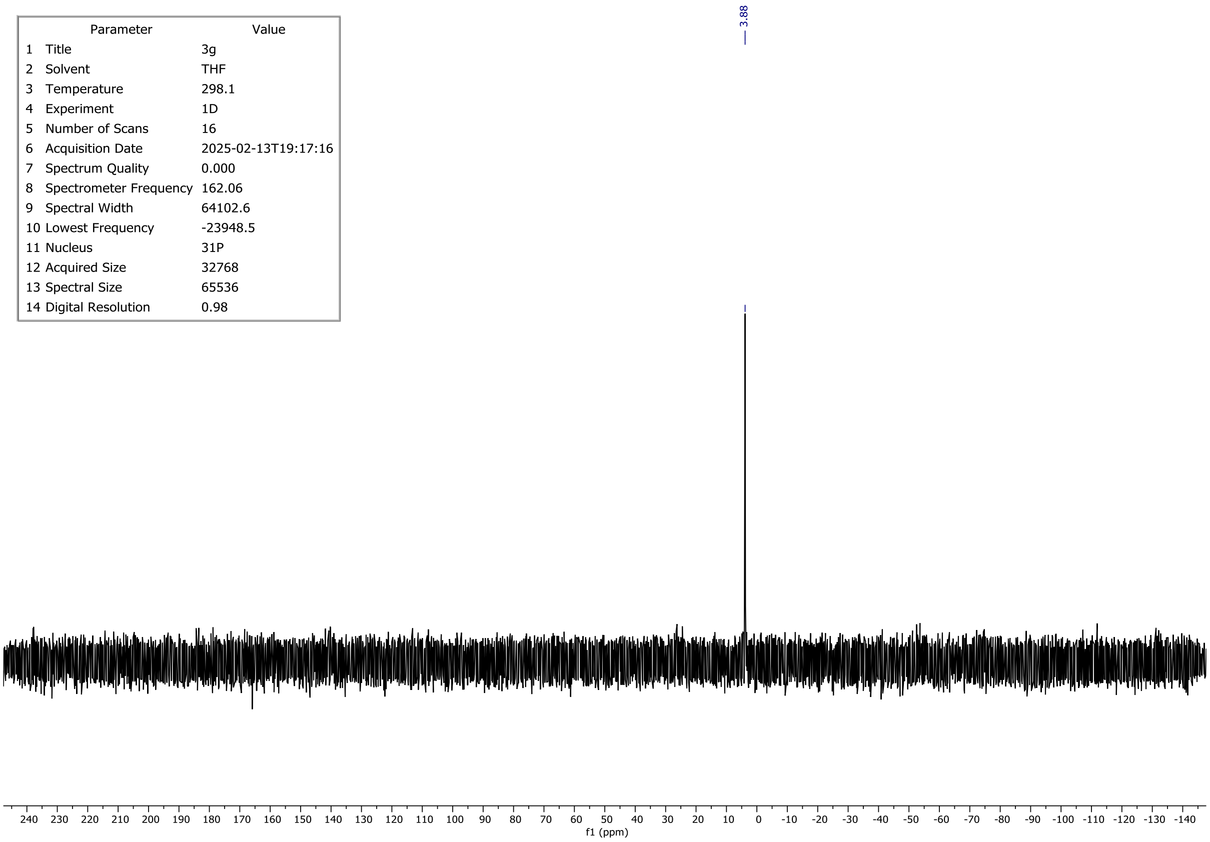


**Figure S39** $^{31}P\{^{1}H\}$ NMR spectrum of compound **3g**[Li·(12-c-4)] in THF-$d_8$.

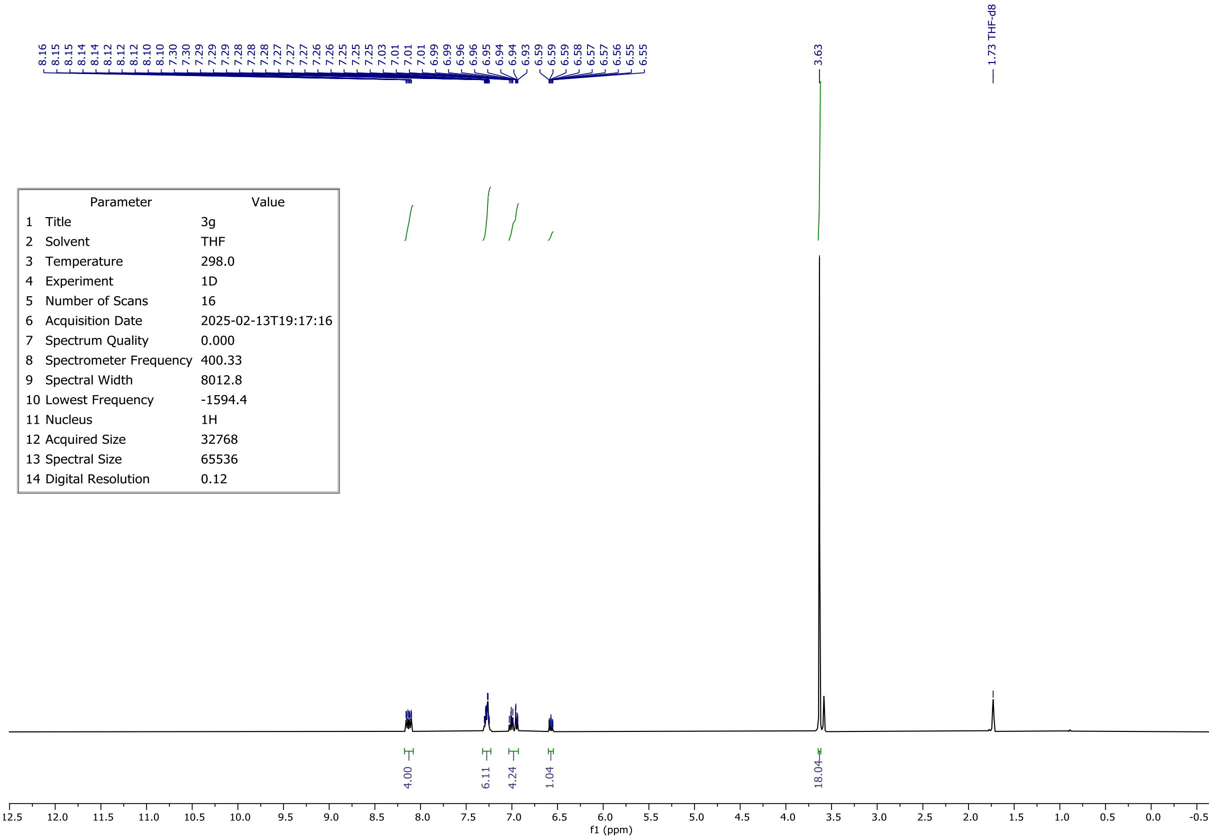


**Figure S40** $^{1}H$ NMR spectrum of compound **3g**[Li·(12-c-4)]in THF-$d_8$.

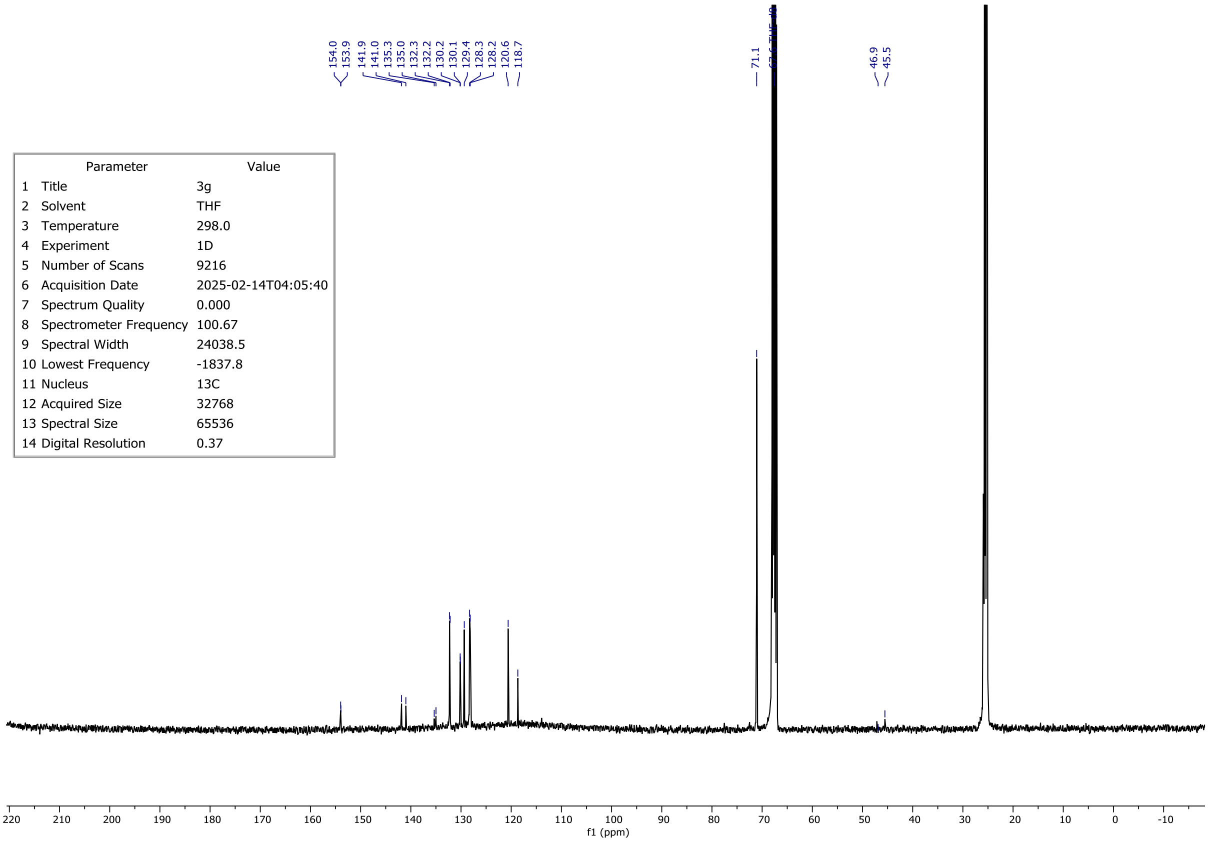


**Figure S41** $^{13}C\{^{1}H\}$ NMR spectrum of compound **3g**[Li·(12-c-4)] in THF-$d_8$.

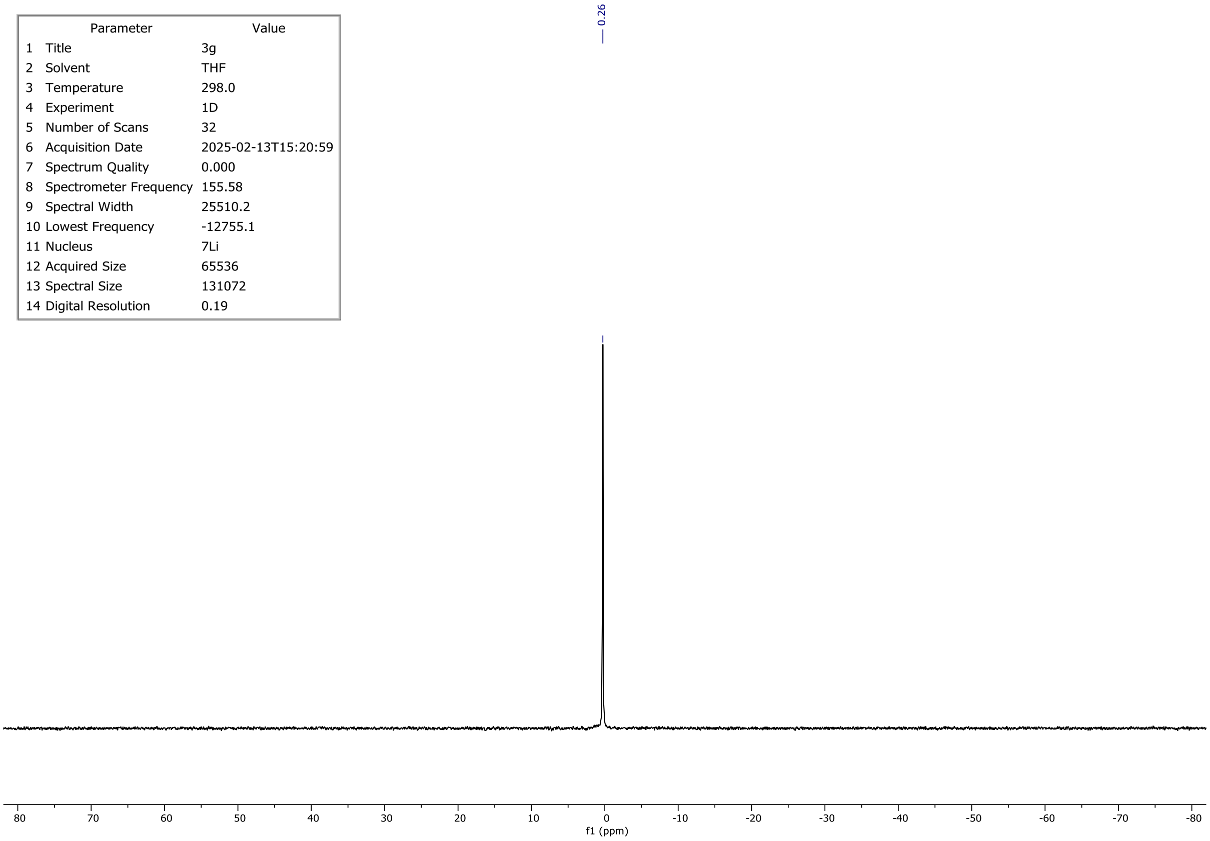


**Figure S42** $^{7}Li$ NMR spectrum of compound **3g**[Li·(12-c-4)]in THF-$d_8$.

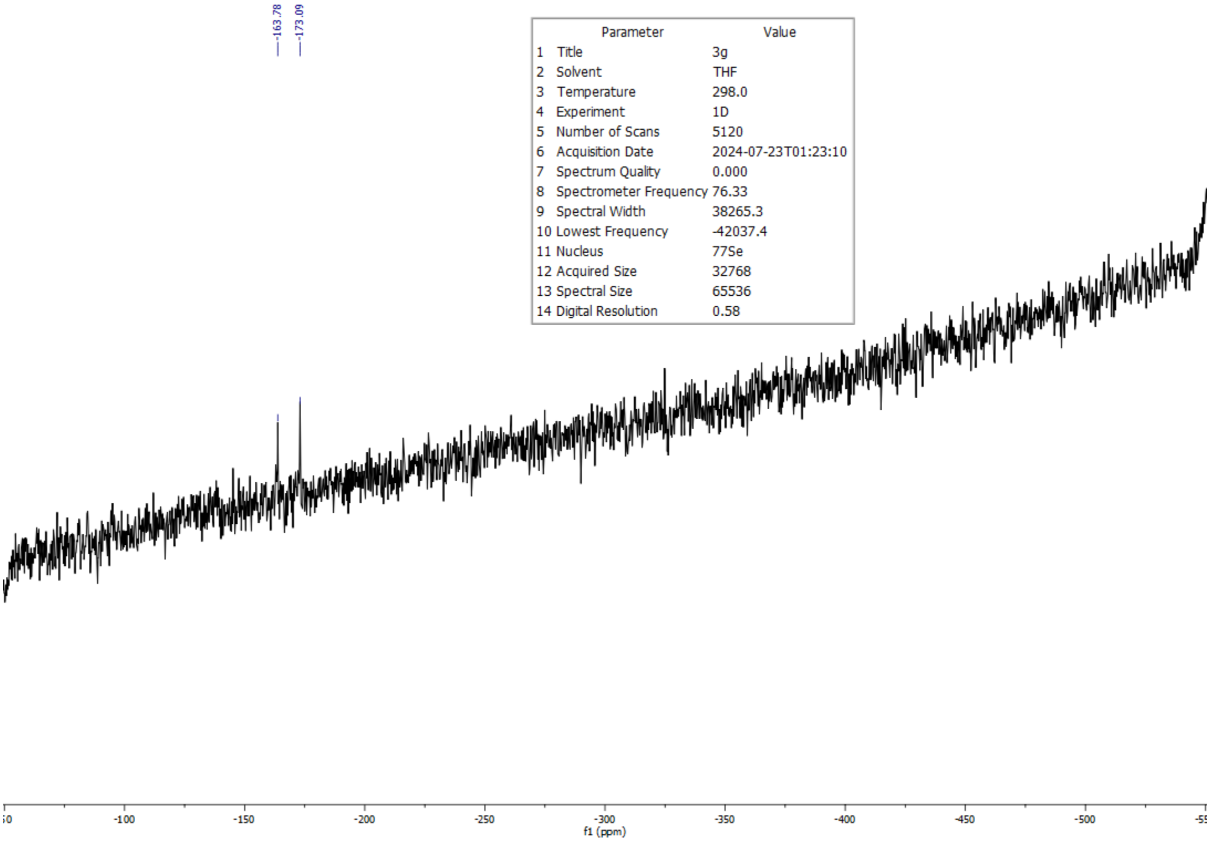


**Figure S43** $^{77}$Se NMR spectrum of compound **3g**[Li·(12-c-4)]in THF-d$_8$.

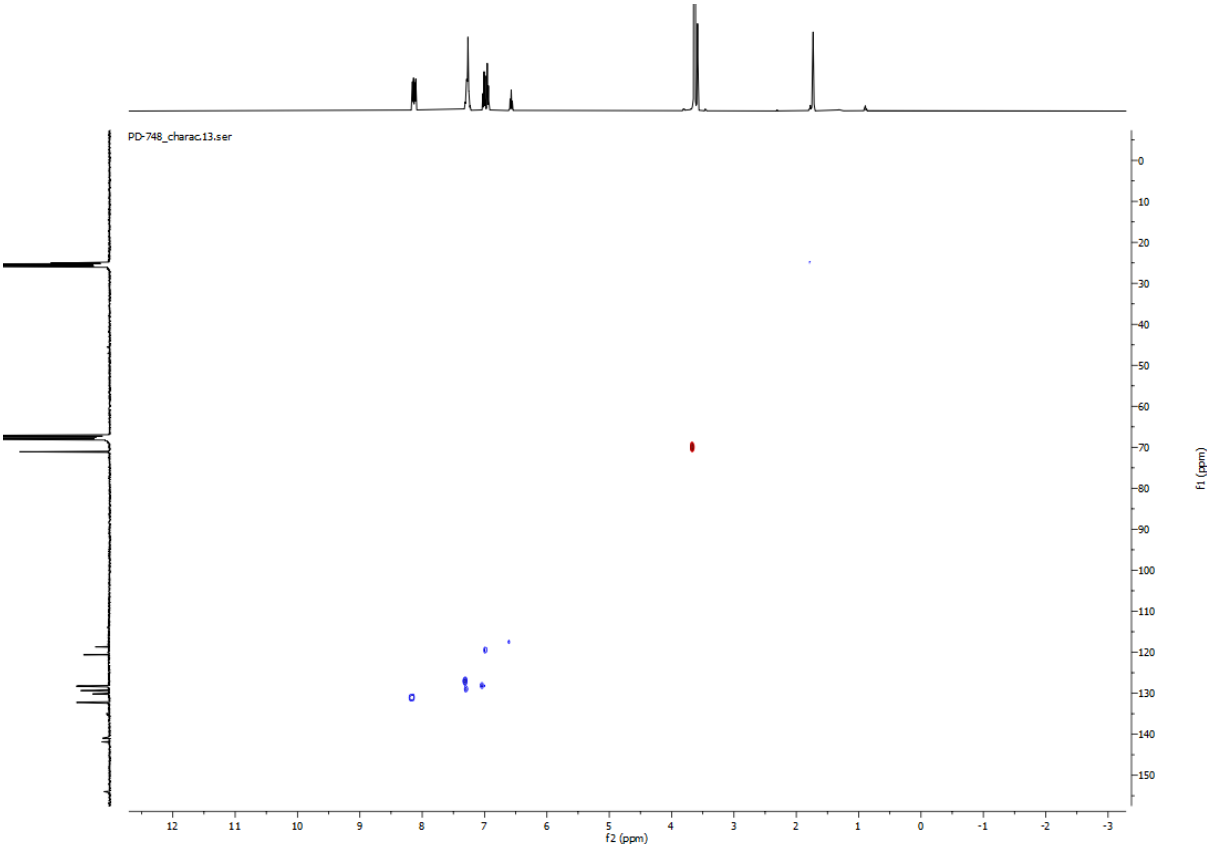


**Figure S44** $^{1}$H-$^{13}$C HSQC NMR spectrum of compound **3g**[Li·(12-c-4)] in THF-d$_8$.

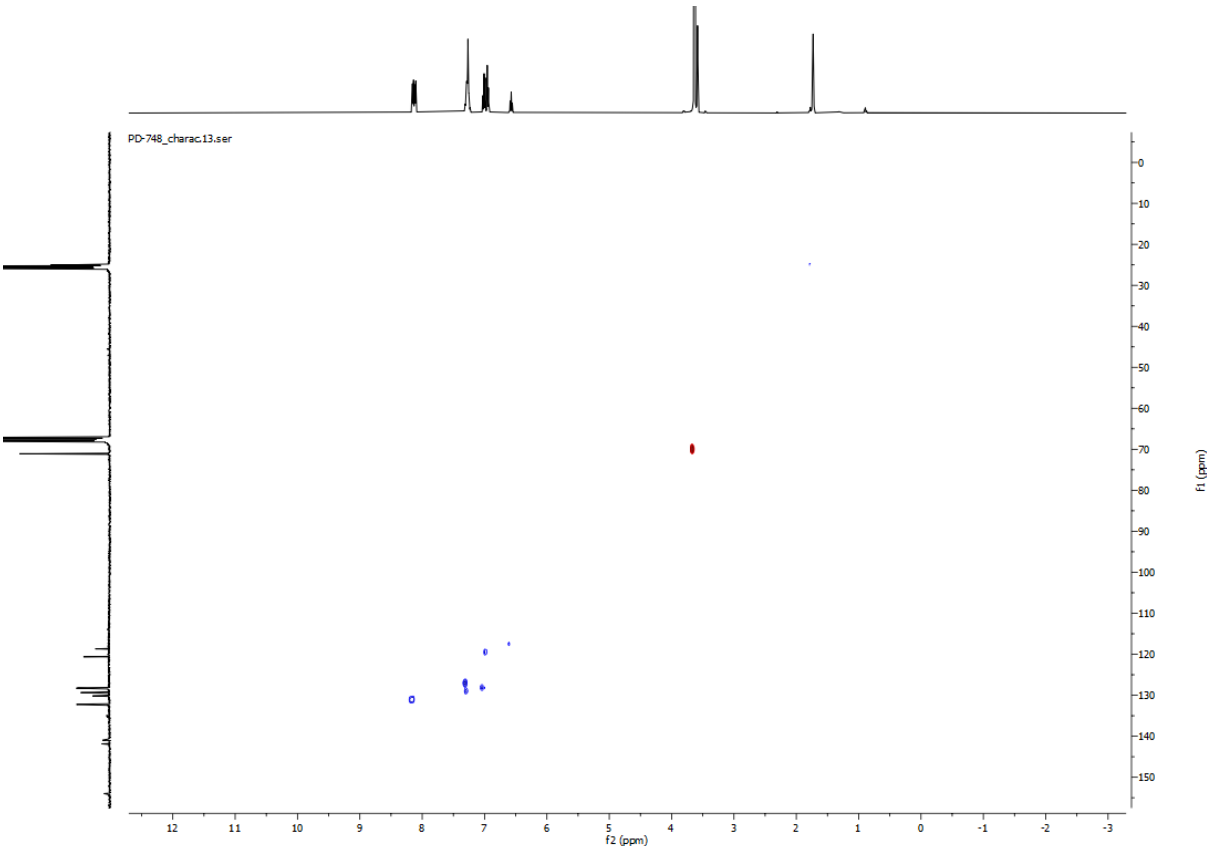


**Figure S 45** $^{1}H$-$^{13}C$ HMBC NMR spectrum of compound **3g**[Li·(12-c-4)] in THF-$d_8$.

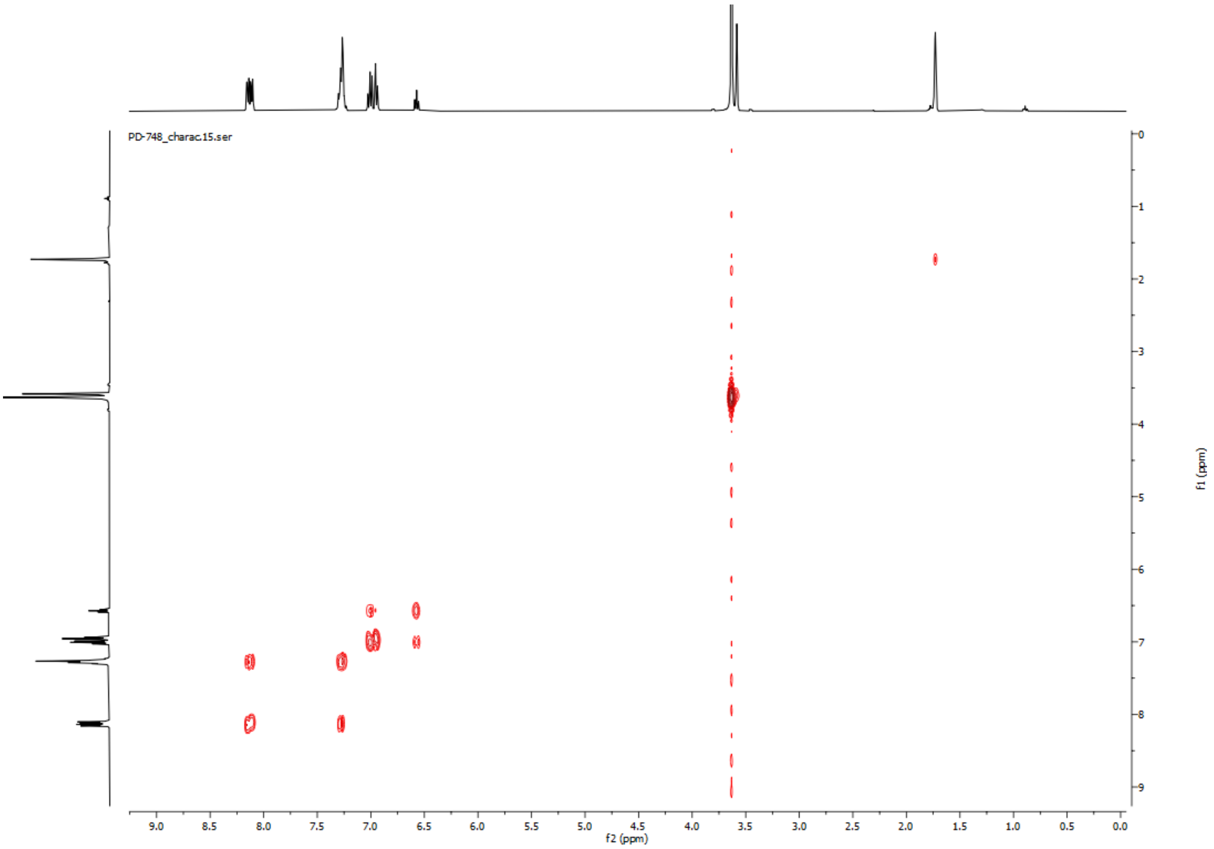


**Figure S46** $^{1}H$-$^{1}H$ COSY NMR spectrum of compound in **3g**[Li·(12-c-4)] THF-$d_8$.

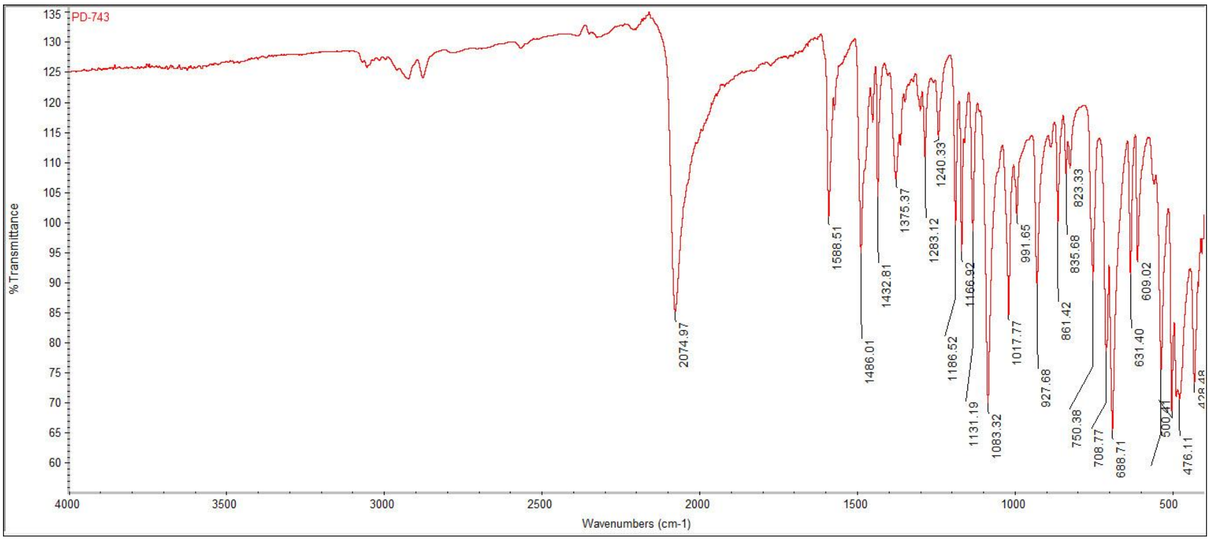


**Figure S47** IR spectrum of compound **3g**[Li·(12-c-4)] (solid state).

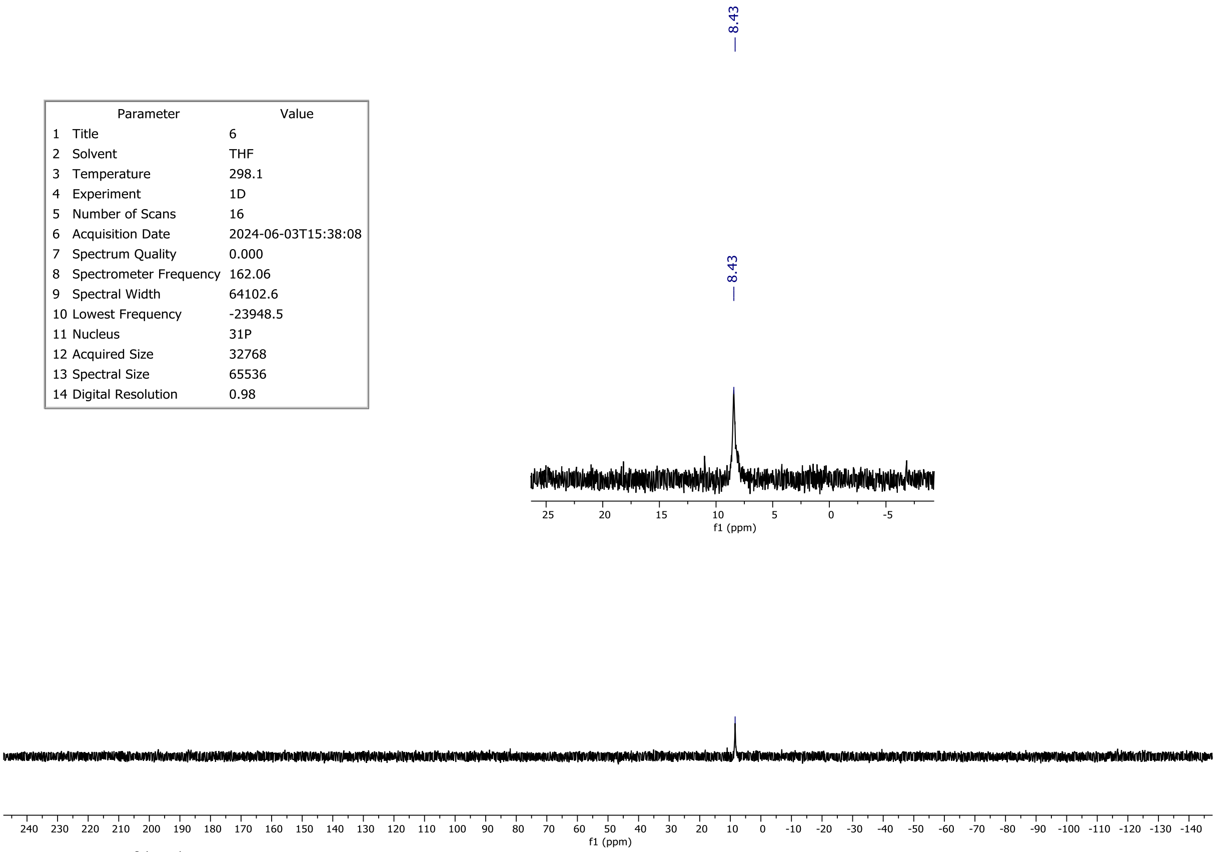


| | Parameter | Value |
|---|---|---|
| 1 | Title | 6 |
| 2 | Solvent | THF |
| 3 | Temperature | 298.1 |
| 4 | Experiment | 1D |
| 5 | Number of Scans | 16 |
| 6 | Acquisition Date | 2024-06-03T15:38:08 |
| 7 | Spectrum Quality | 0.000 |
| 8 | Spectrometer Frequency | 162.06 |
| 9 | Spectral Width | 64102.6 |
| 10 | Lowest Frequency | -23948.5 |
| 11 | Nucleus | 31P |
| 12 | Acquired Size | 32768 |
| 13 | Spectral Size | 65536 |
| 14 | Digital Resolution | 0.98 |

**Figure S48** $^{31}P\{^{1}H\}$ NMR spectrum of compound **6** in THF-$d_8$.

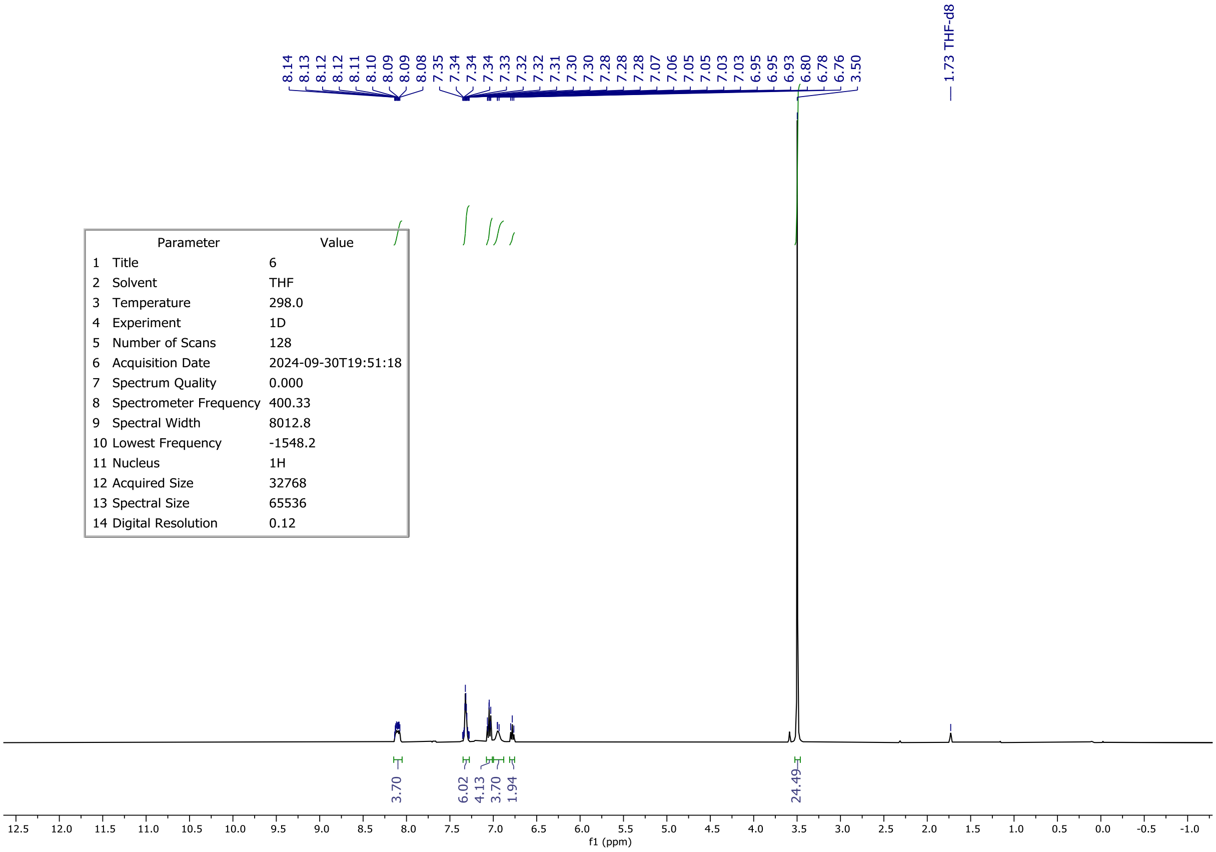


**Figure S49** $^{1}$H NMR spectrum of compound **6** in THF-d$_8$.

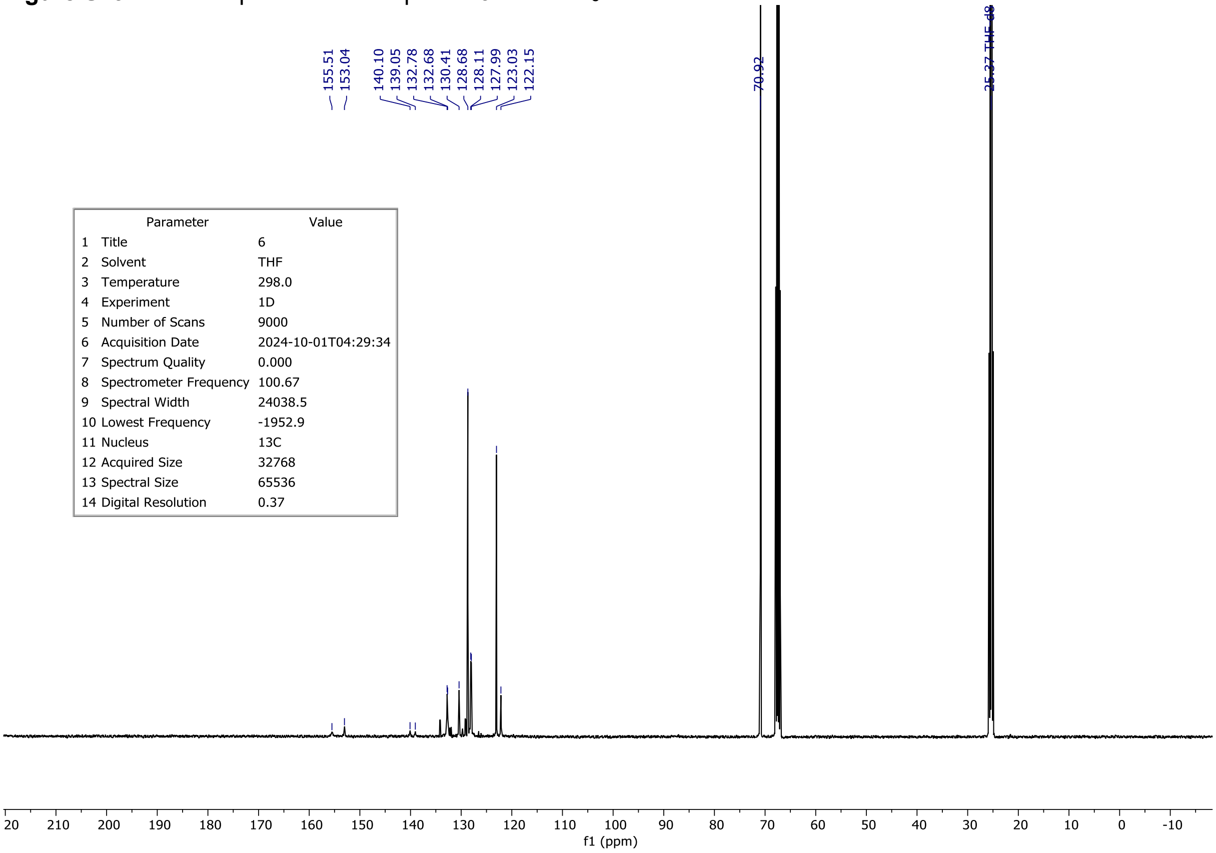


**Figure S50** $^{13}$C{$^{1}$H} NMR spectrum of compound **6** in THF-d$_8$.

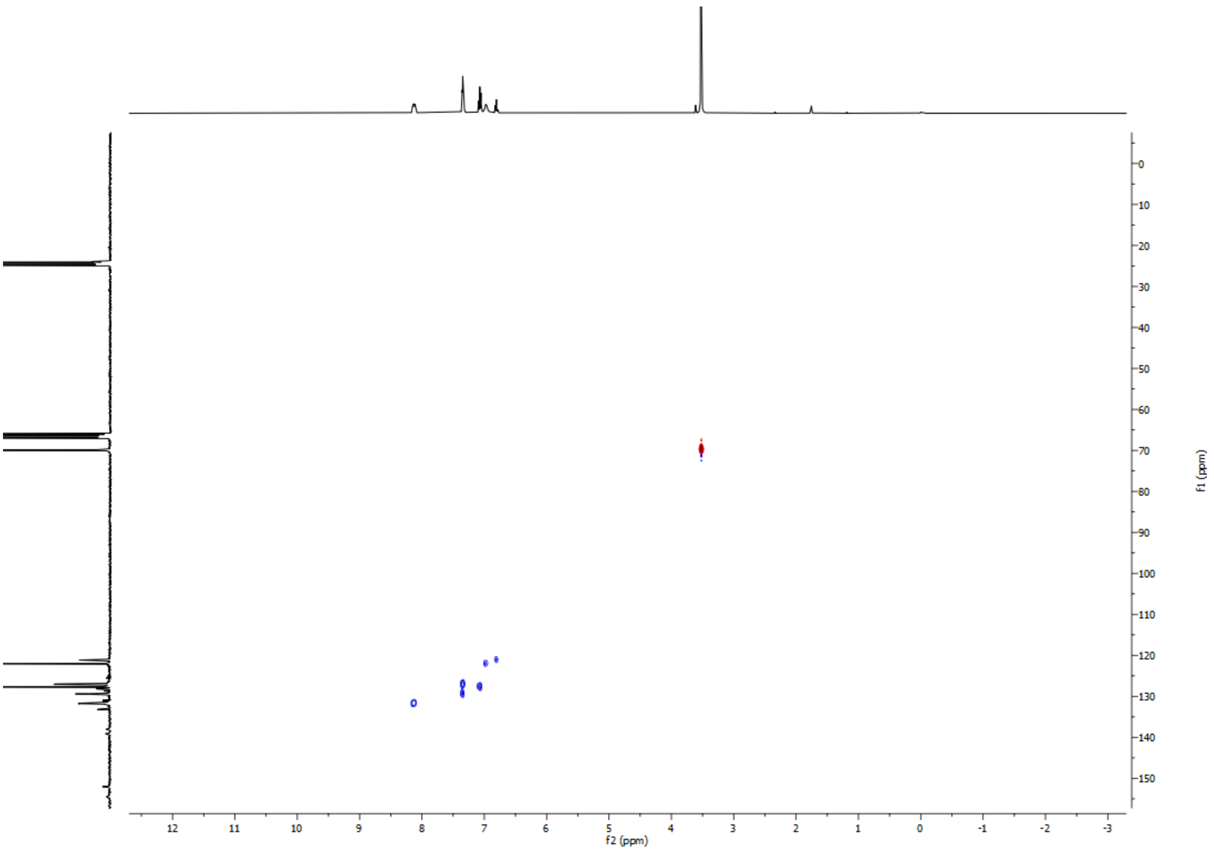

**Figure S51** $^{1}H$-$^{13}C$ HSQC NMR spectrum of compound **6** in THF-$d_8$.

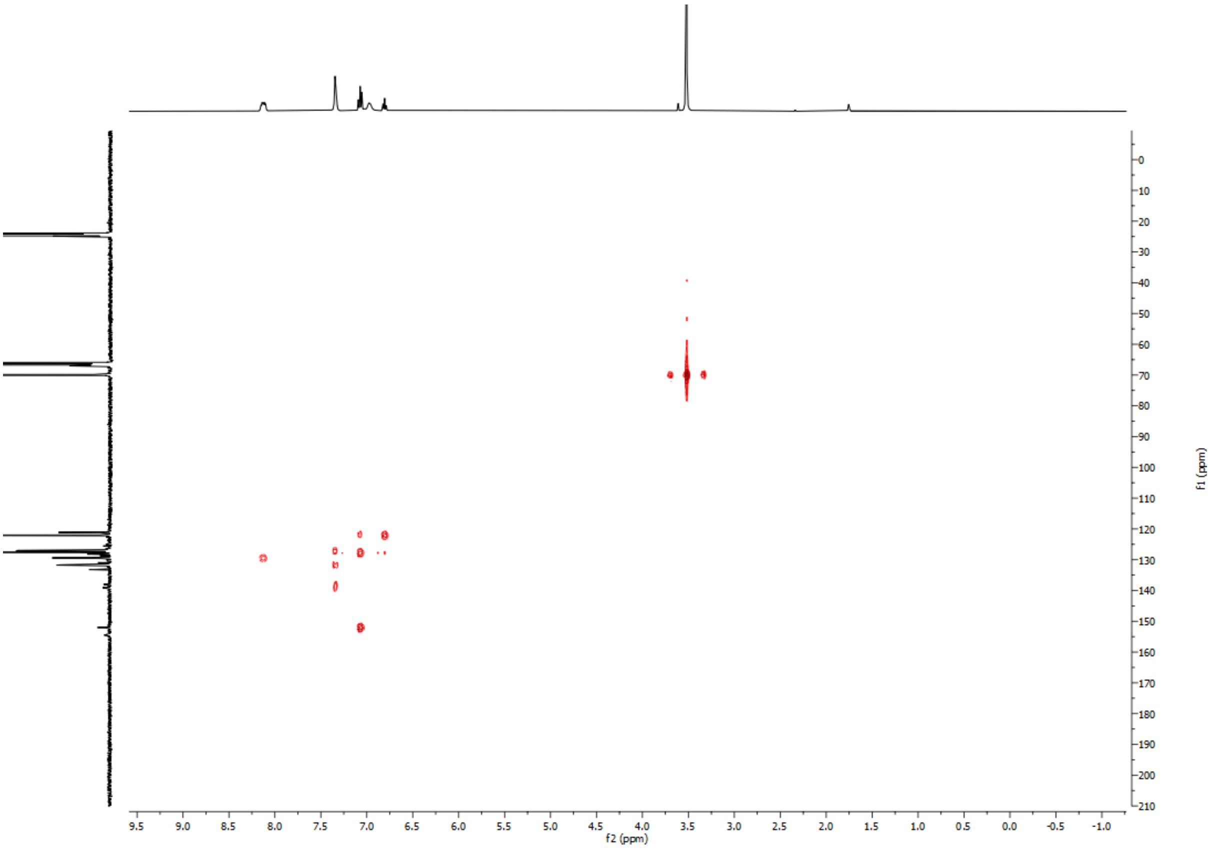

**Figure S52** $^{1}H$-$^{13}C$ HMBC NMR spectrum of compound **6** in THF-$d_8$.

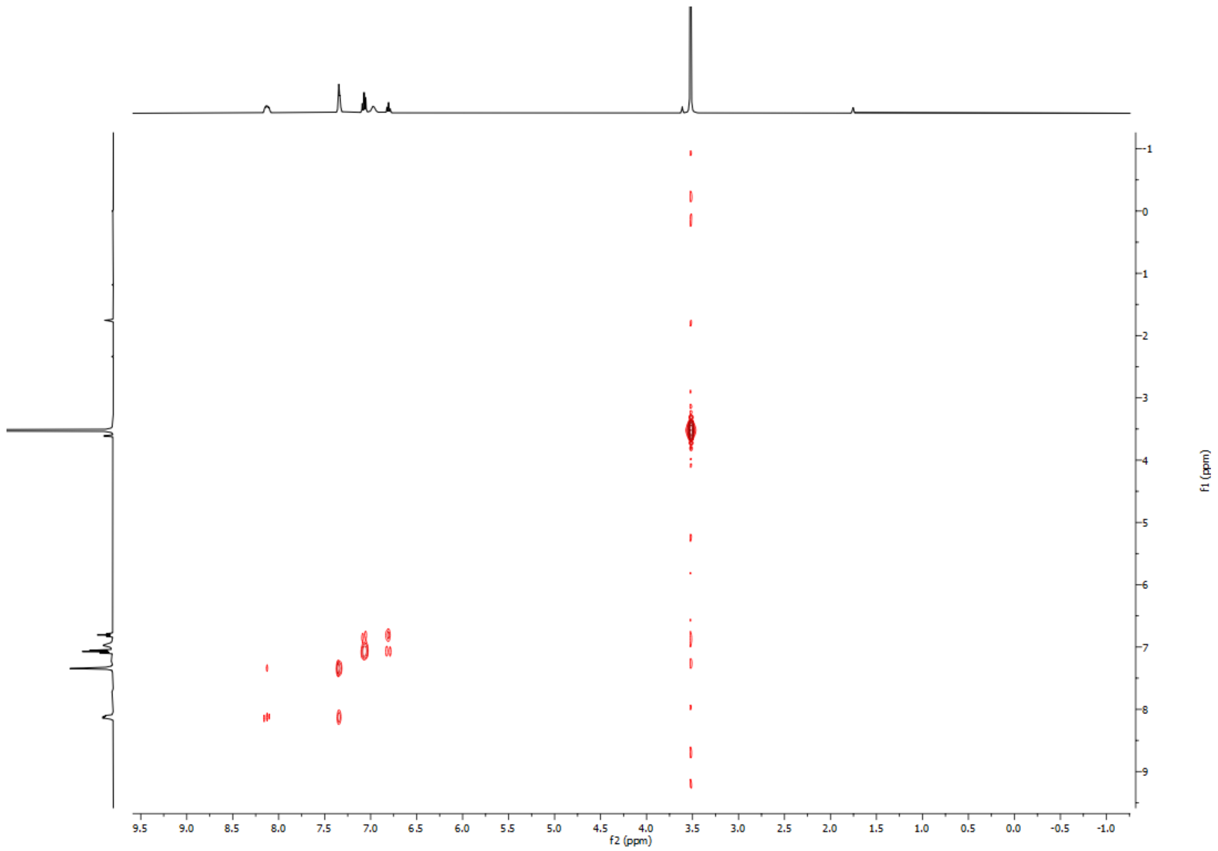


**Figure S53** $^{1}H$-$^{1}H$ COSY NMR spectrum of compound **6** in THF-$d_8$.

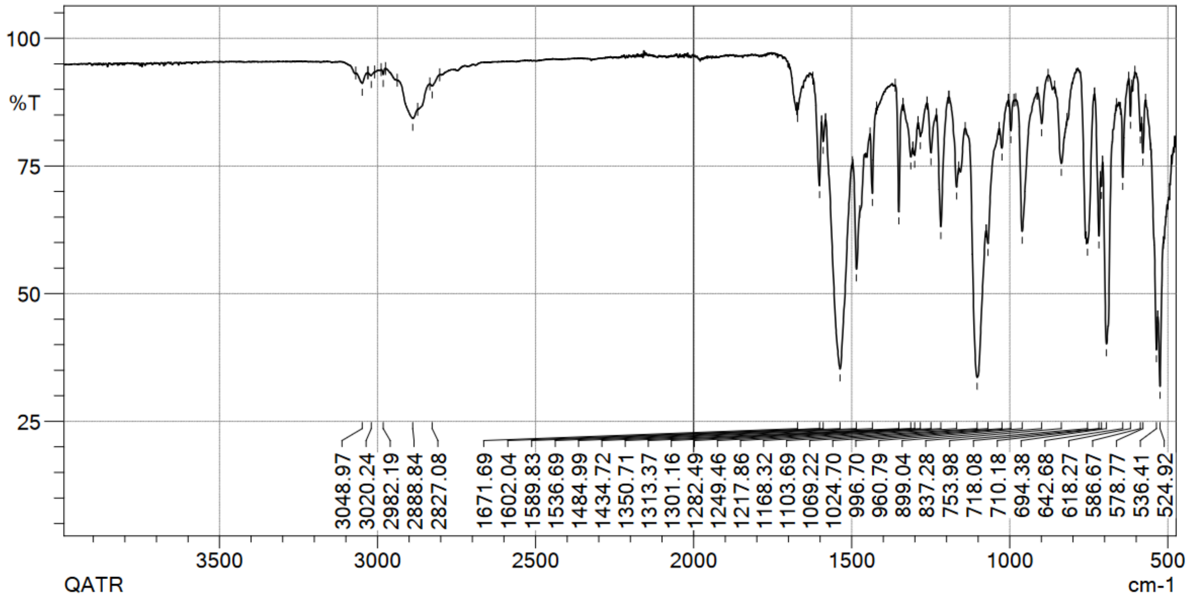


**Figure S54** IR spectrum of compound **6** (solid state).

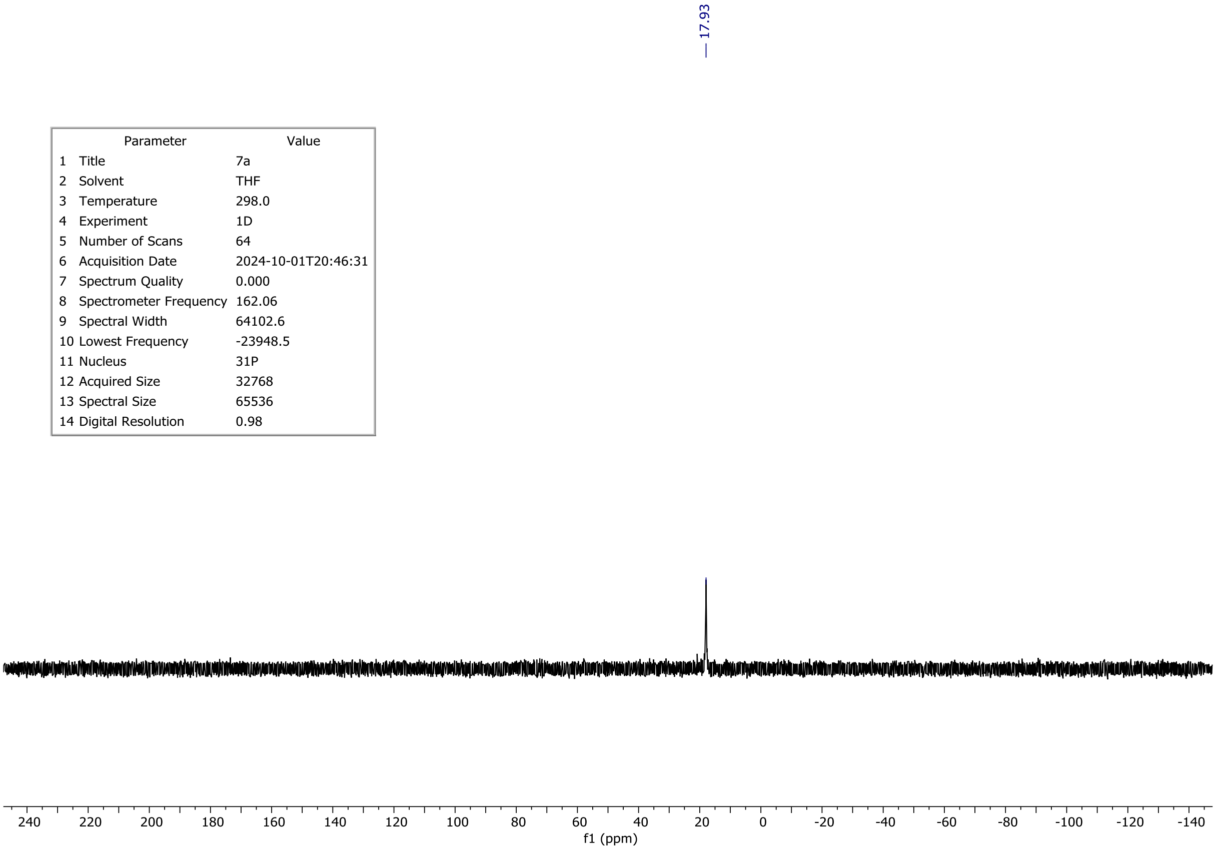


**Figure S55** $^{31}P\{^{1}H\}$ NMR spectrum of compound **7a** in THF-$d_8$.

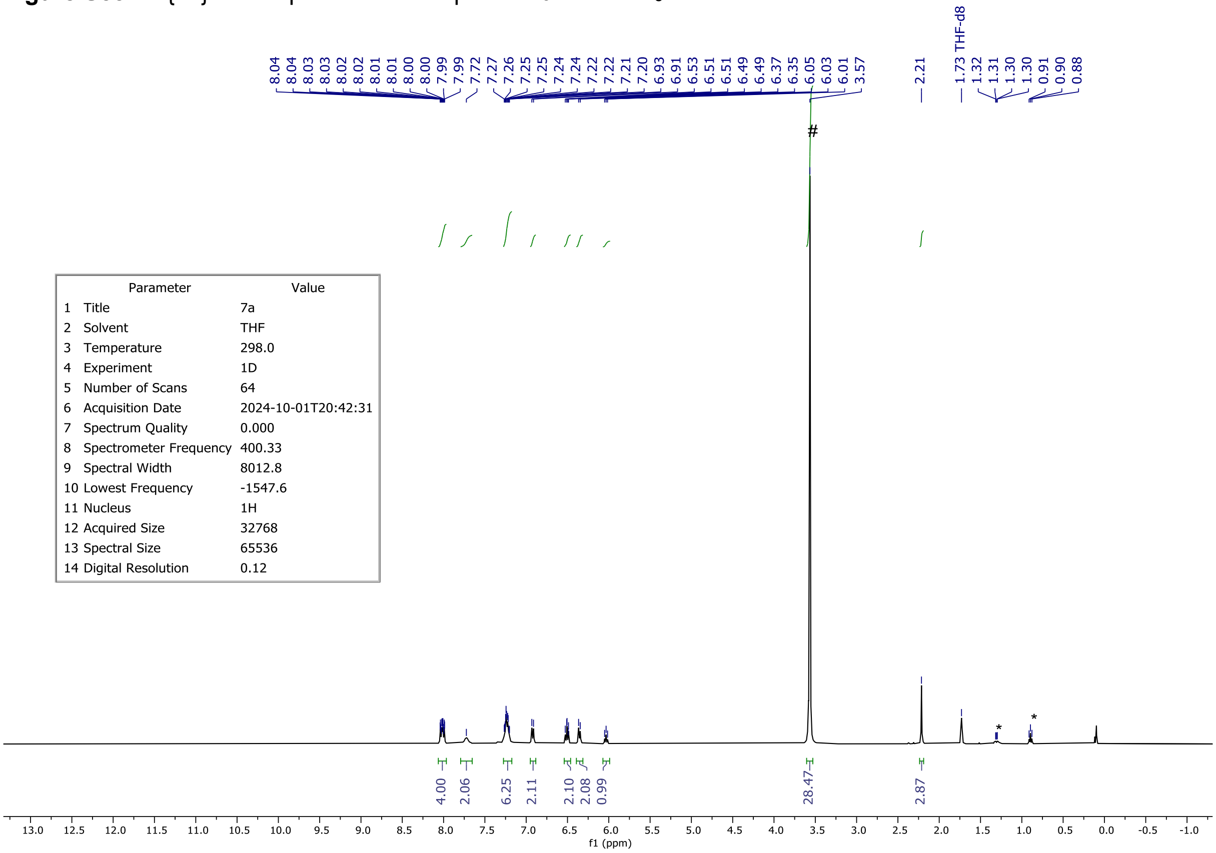


**Figure S56** $^{1}H$ NMR spectrum of compound **7a** in THF-$d_8$ (* = residual pentane, # = residual THF + 18-c-6).

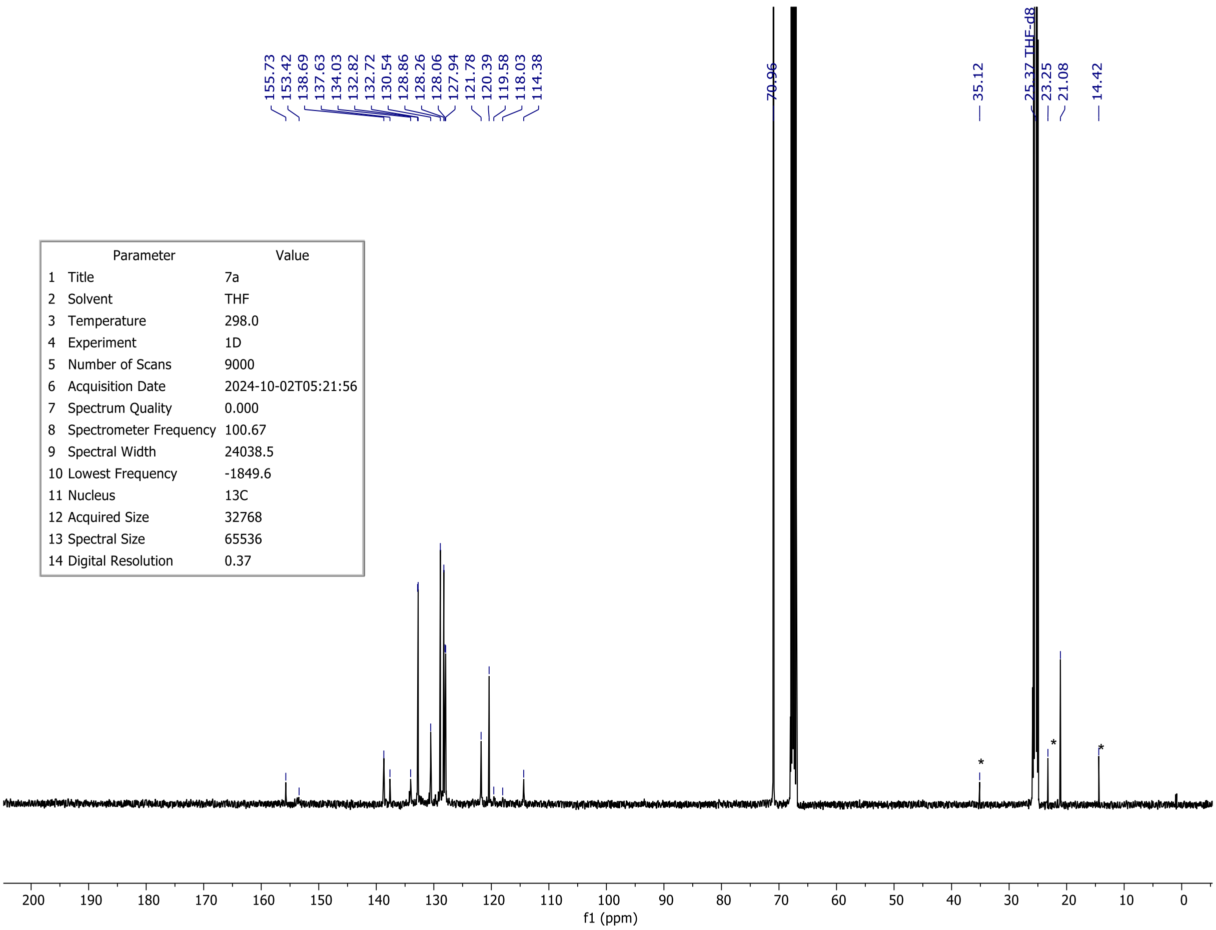


| Parameter | | Value |
|---|---|---|
| 1 | Title | 7a |
| 2 | Solvent | THF |
| 3 | Temperature | 298.0 |
| 4 | Experiment | 1D |
| 5 | Number of Scans | 9000 |
| 6 | Acquisition Date | 2024-10-02T05:21:56 |
| 7 | Spectrum Quality | 0.000 |
| 8 | Spectrometer Frequency | 100.67 |
| 9 | Spectral Width | 24038.5 |
| 10 | Lowest Frequency | -1849.6 |
| 11 | Nucleus | 13C |
| 12 | Acquired Size | 32768 |
| 13 | Spectral Size | 65536 |
| 14 | Digital Resolution | 0.37 |

**Figure S57** $^{13}C\{^{1}H\}$ NMR spectrum of compound **7a** in THF-d8 (* = residual pentane).

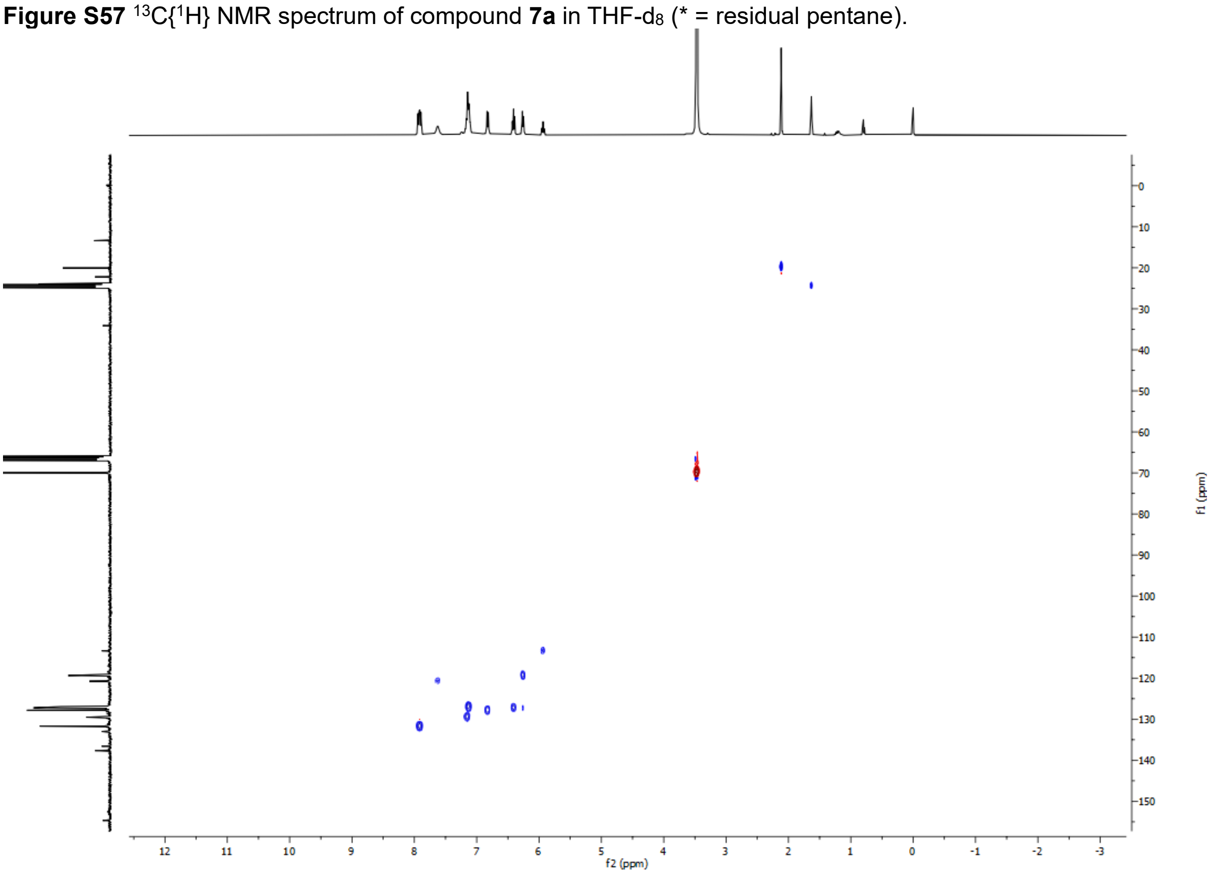


**Figure S58** $^{1}H$-$^{13}C$ HSQC NMR spectrum of compound **7a** in THF-d8.

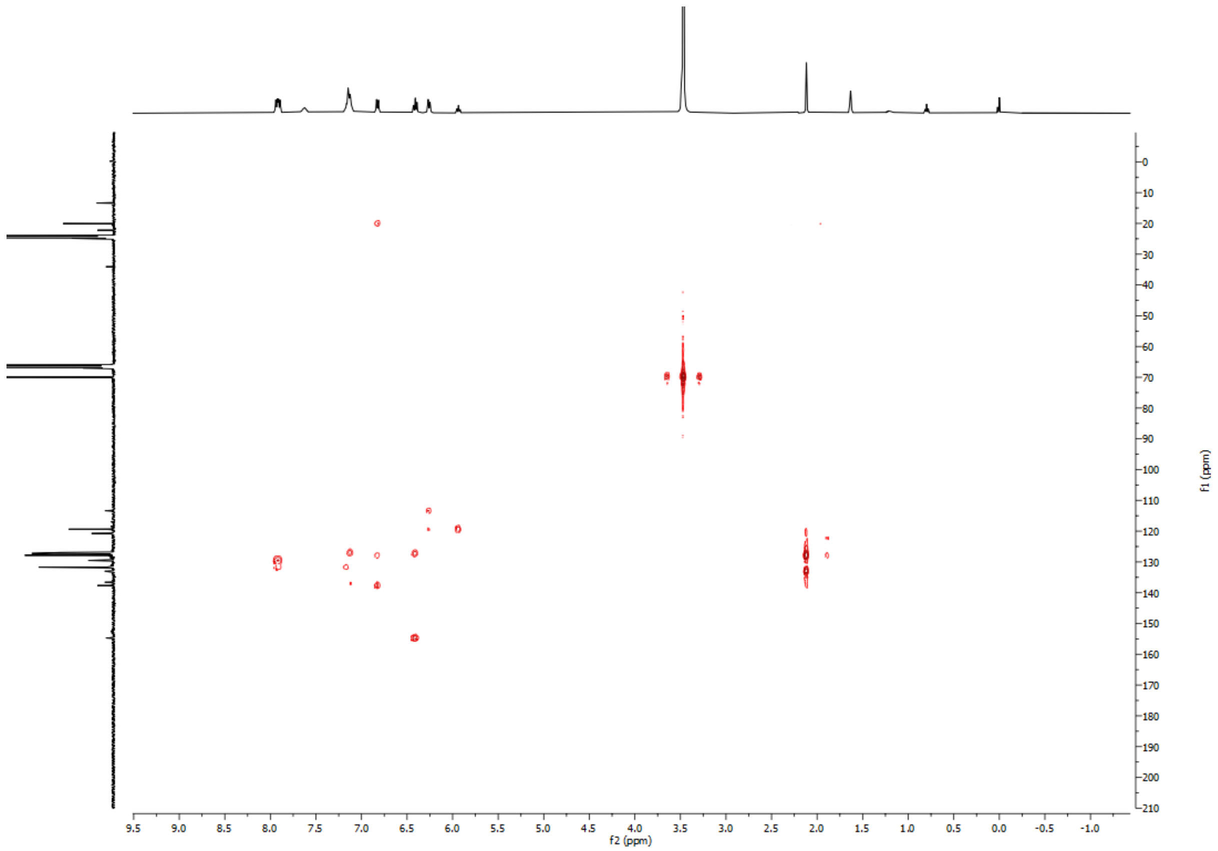

**Figure S59** $^{1}H$-$^{13}C$ HMBC NMR spectrum of compound **7a** in THF-$d_8$.

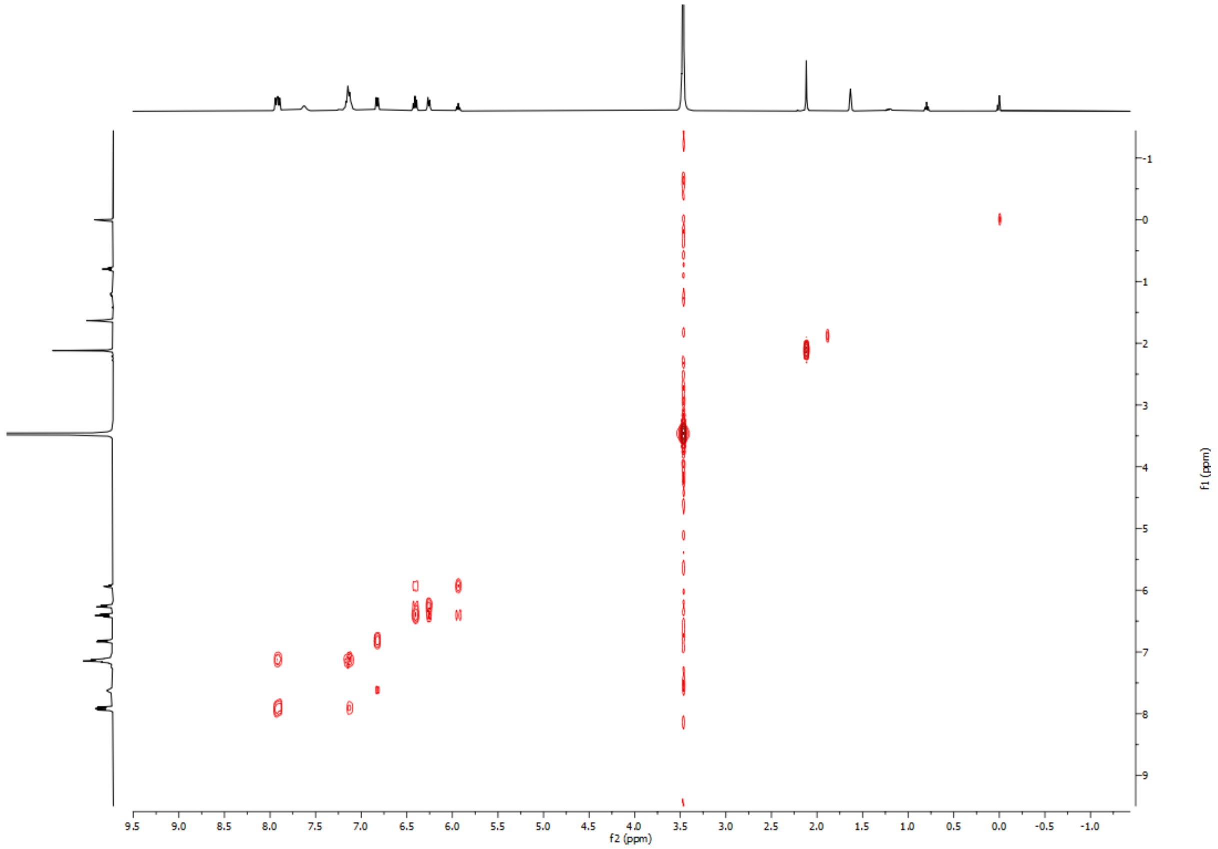

**Figure S60** $^{1}H$-$^{1}H$ COSY NMR spectrum of compound **7a** in THF-$d_8$.

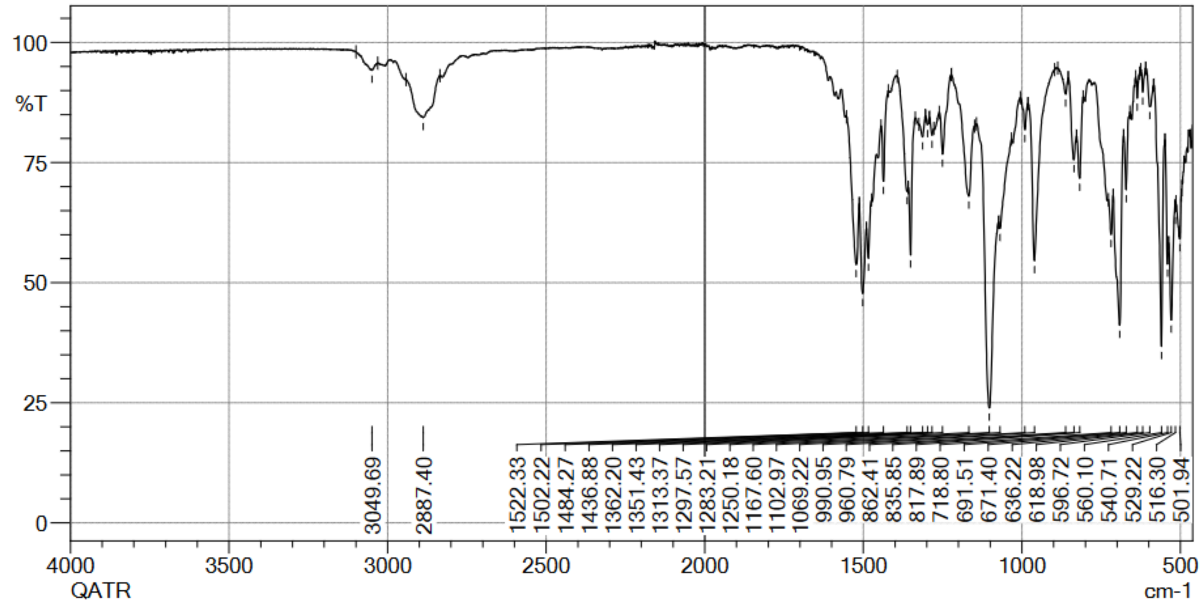


**Figure S61** IR spectrum of compound **7a** (solid state).

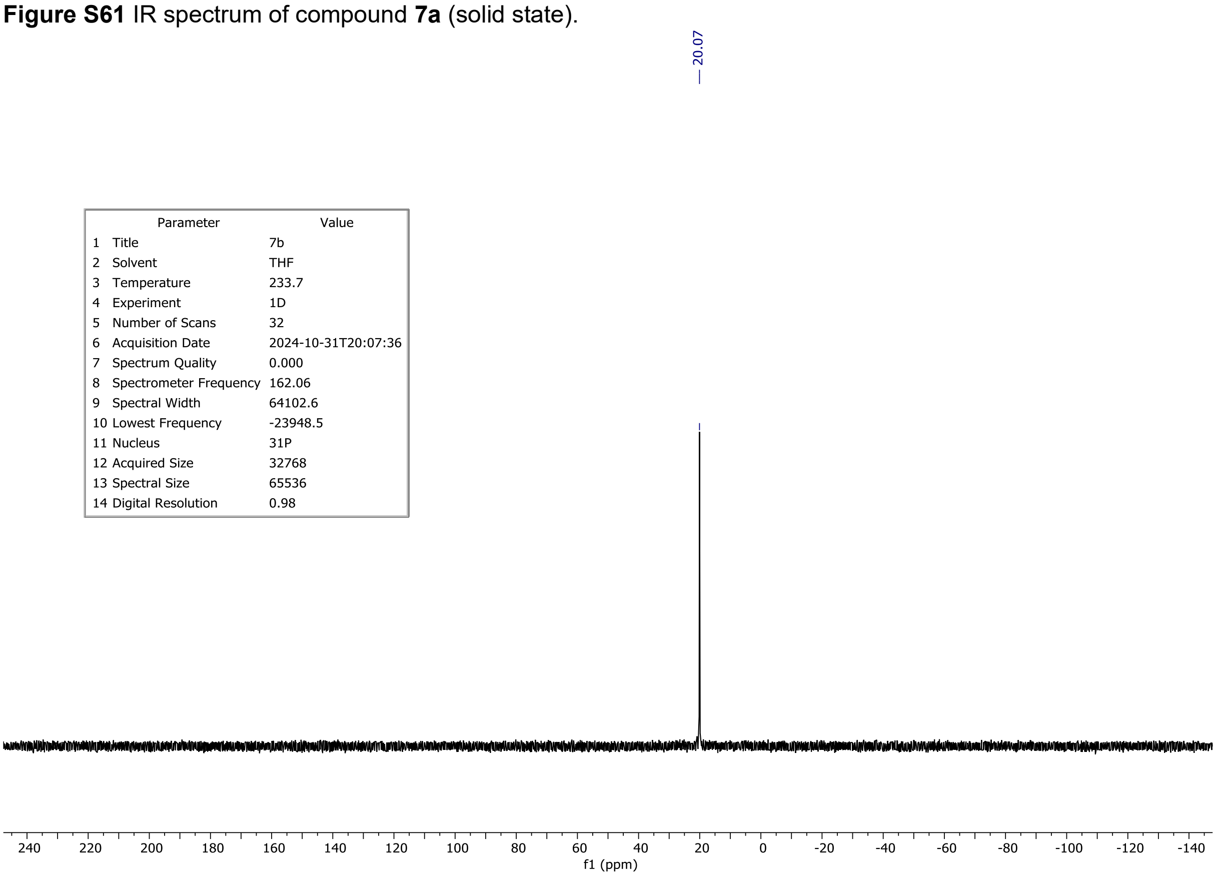


**Figure S62** $^{31}P\{^{1}H\}$ NMR spectrum of compound **7b** in THF-$d_8$.

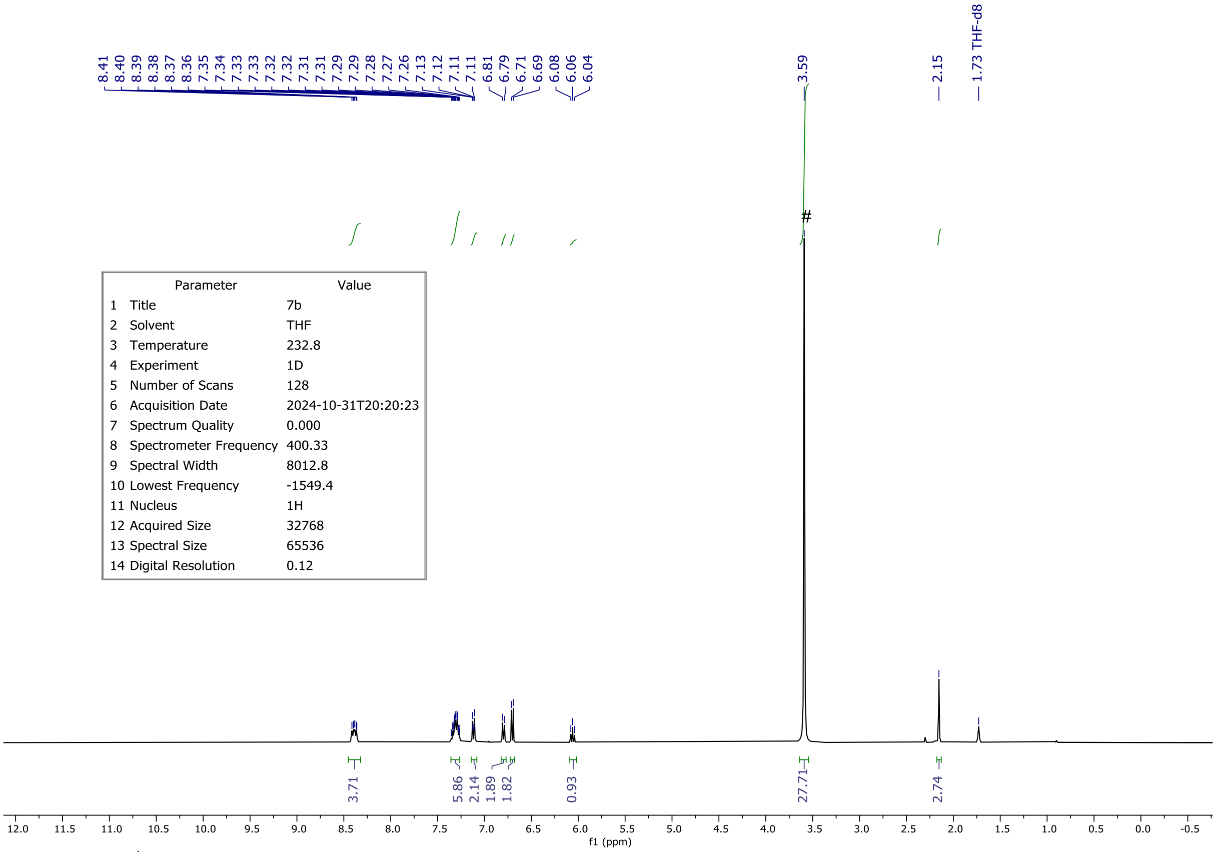


**Figure S63** $^{1}H$ NMR spectrum of compound **7b** in THF-$d_8$ (# = residual THF + 18-c-6).

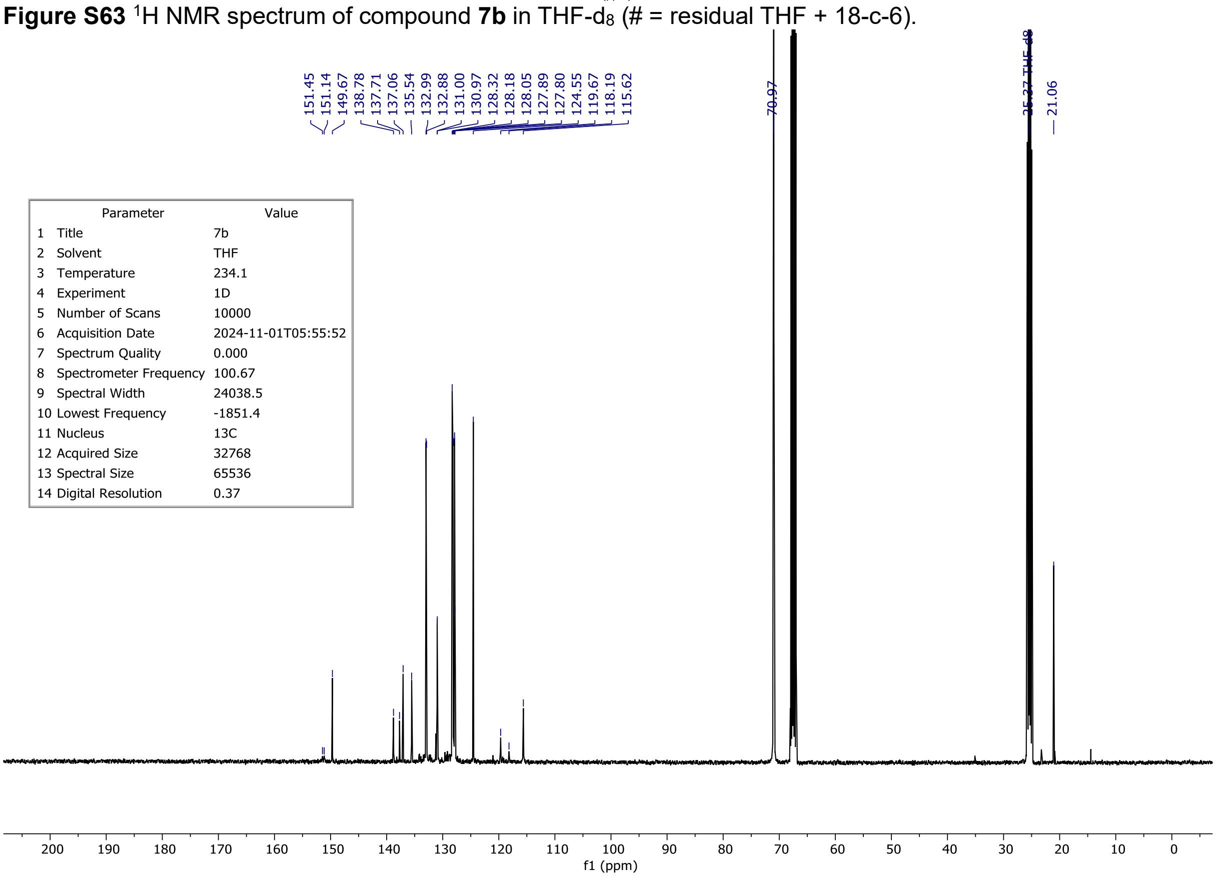


**Figure S64** $^{13}C\{^{1}H\}$ NMR spectrum of compound **7b** in THF-$d_8$.

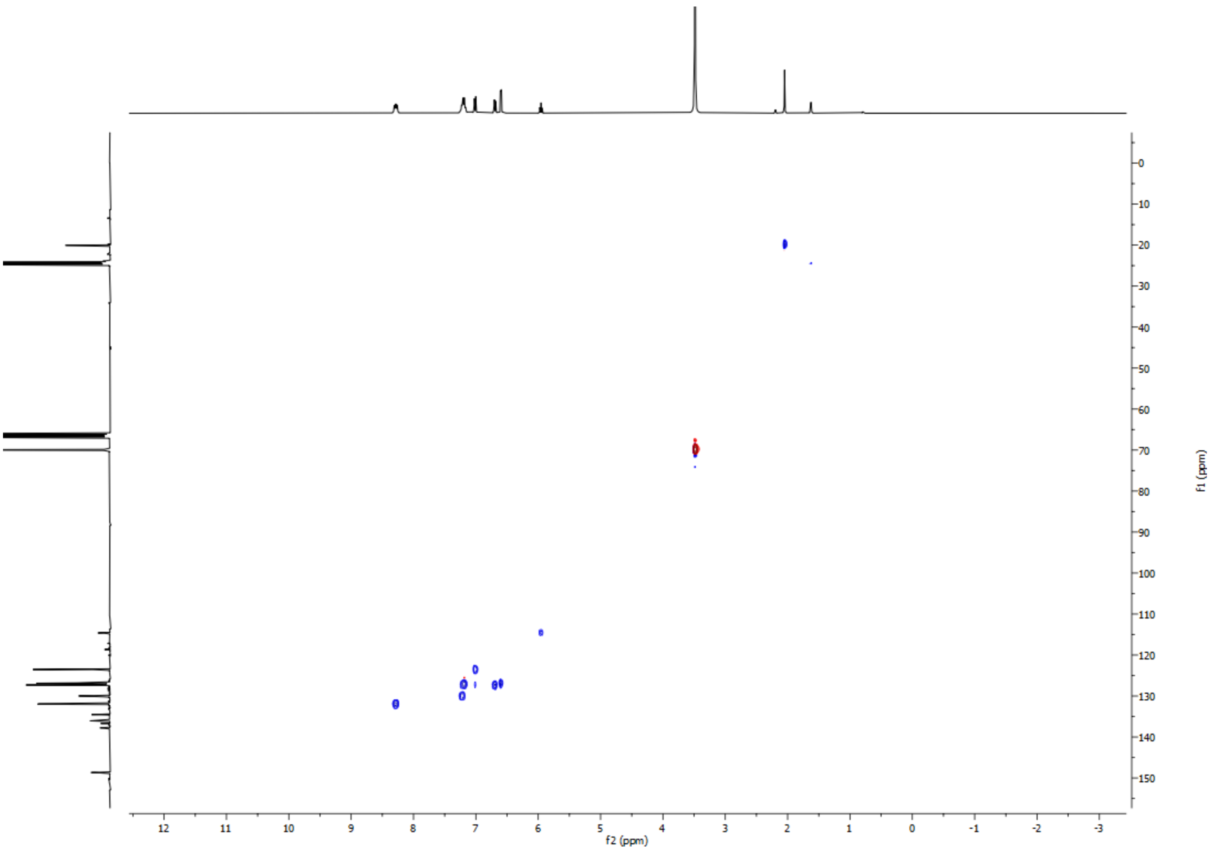

**Figure S65** $^{1}H$-$^{13}C$ HSQC NMR spectrum of compound **7b** in THF-$d_8$.

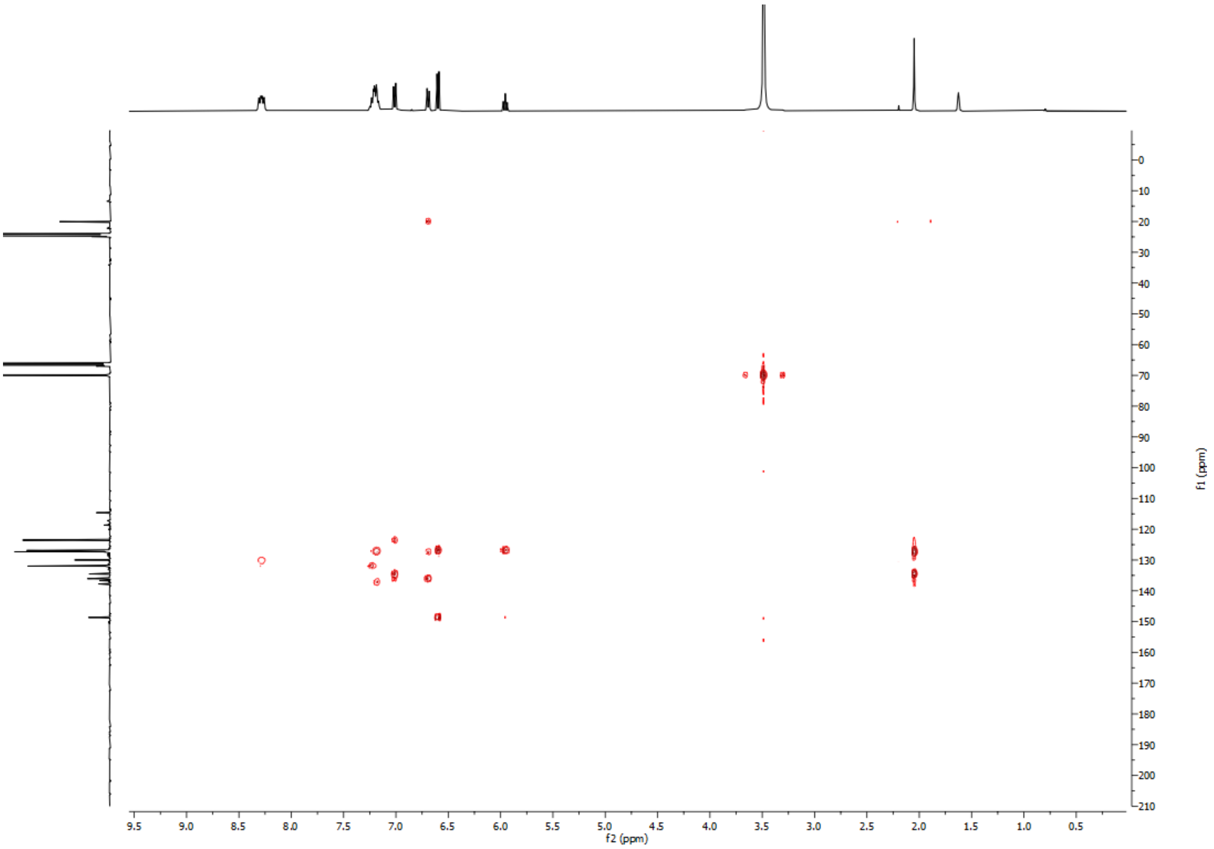

**Figure S66** $^{1}H$-$^{13}C$ HMBC NMR spectrum of compound **7b** in THF-$d_8$.

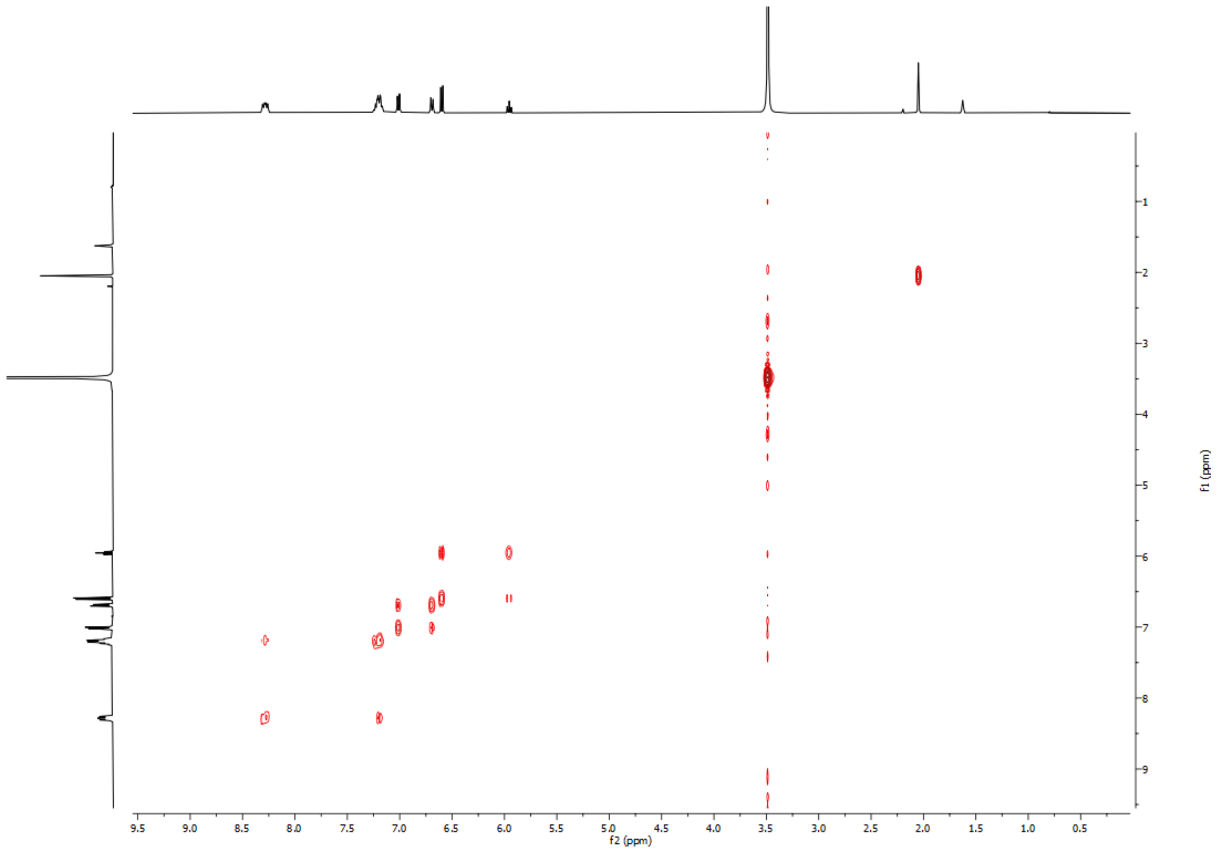


**Figure S67** $^1$H-$^1$H COSY NMR spectrum of compound **7b** in THF-$d_8$.

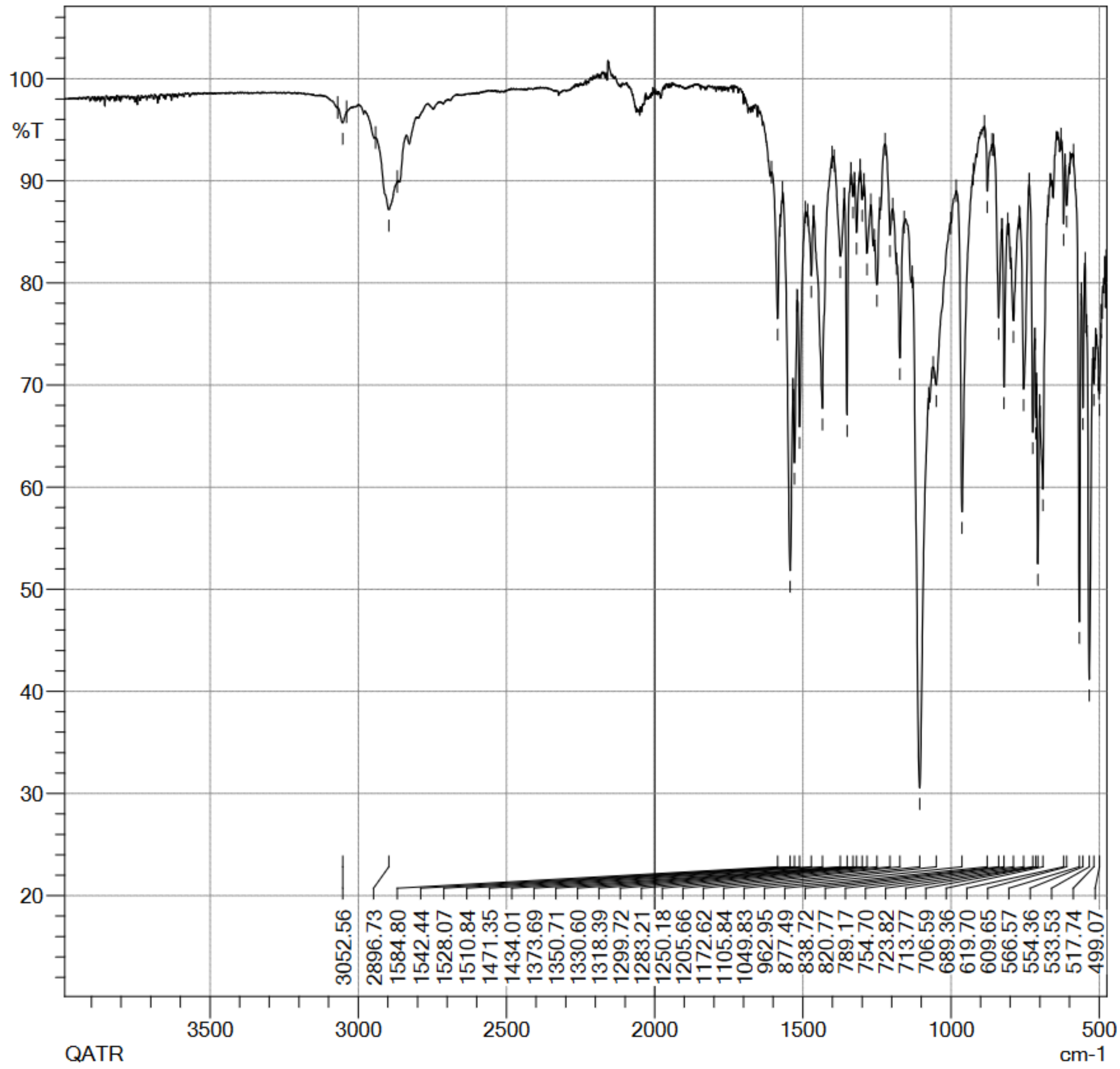


**Figure S68** IR spectrum of compound **7b** (solid state).

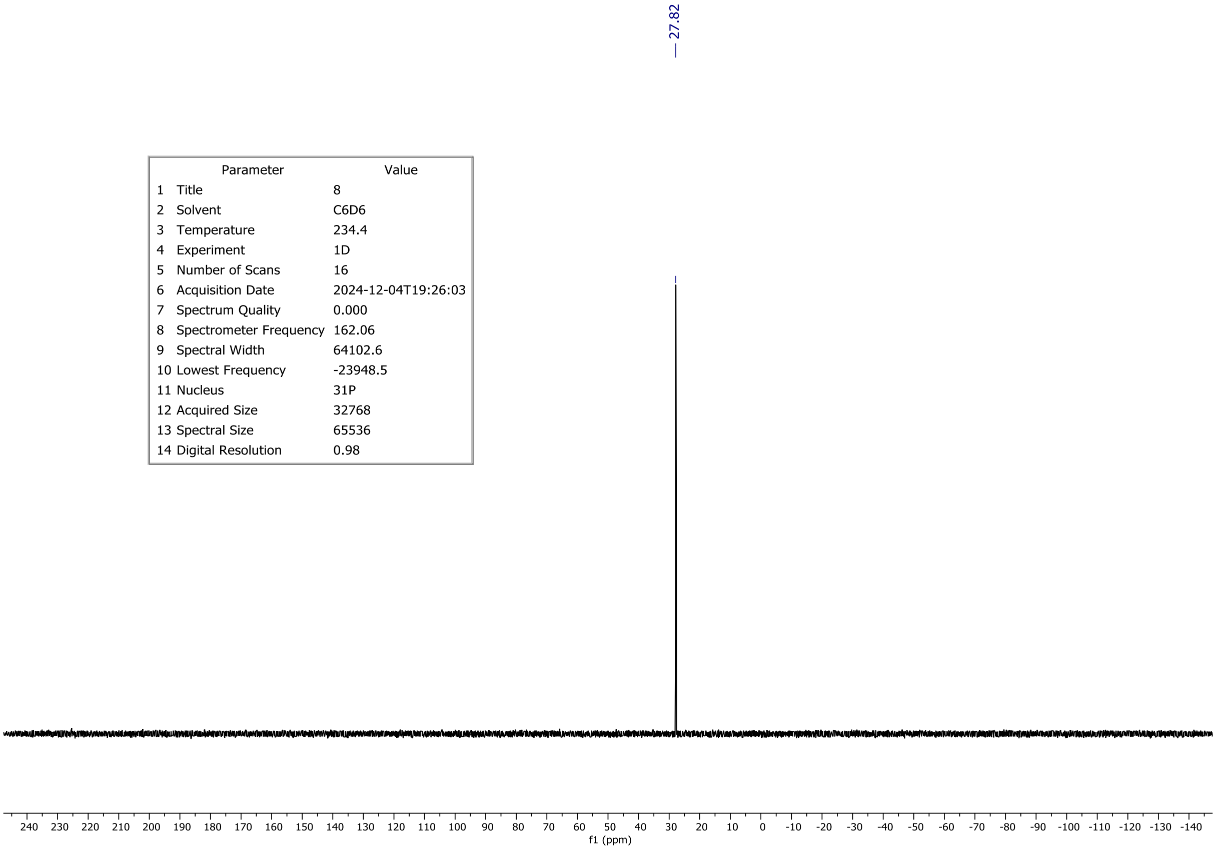


**Figure S69** $^{31}P\{^1H\}$ NMR spectrum of compound **8** in $C_6D_6$.

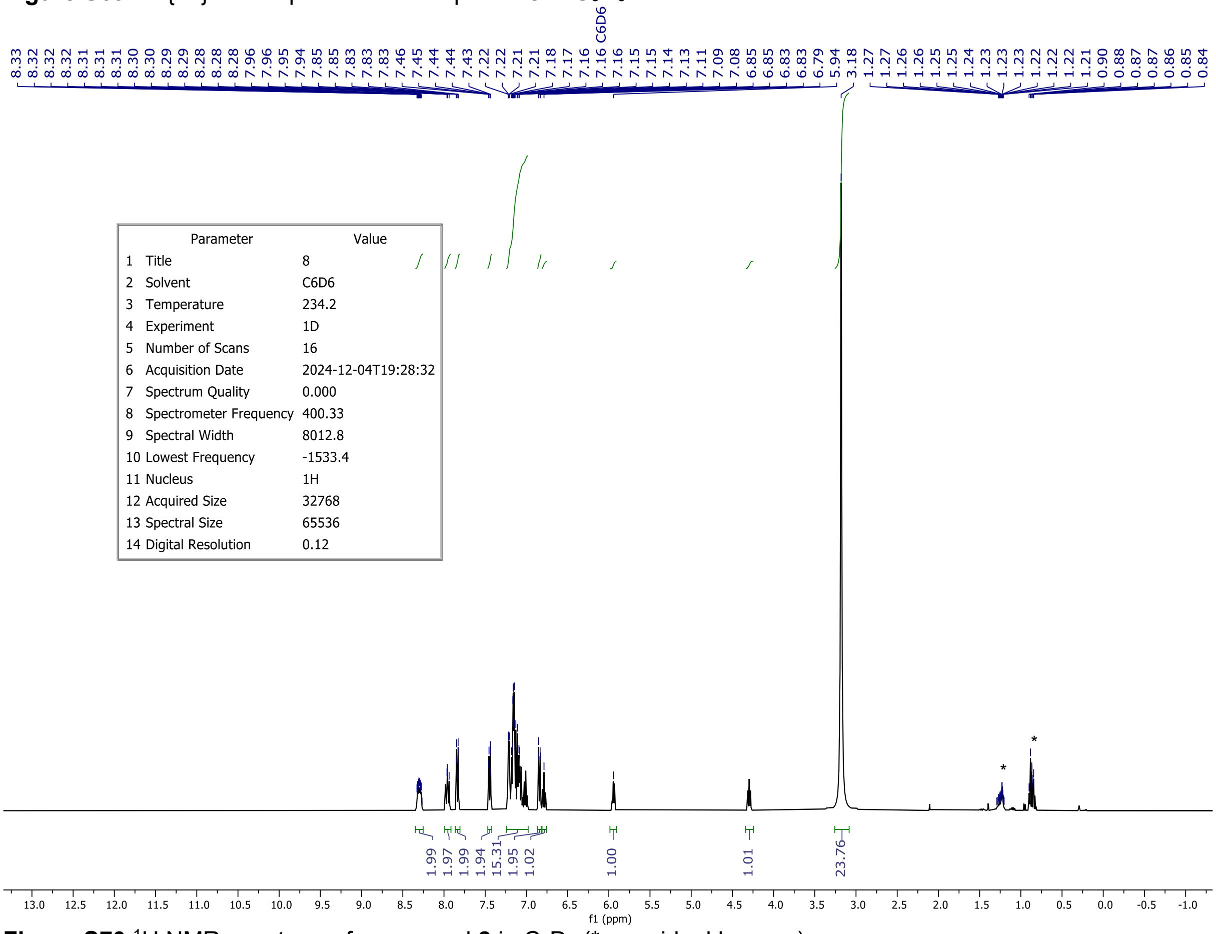


**Figure S70** $^1H$ NMR spectrum of compound **8** in $C_6D_6$ (* = residual hexane).

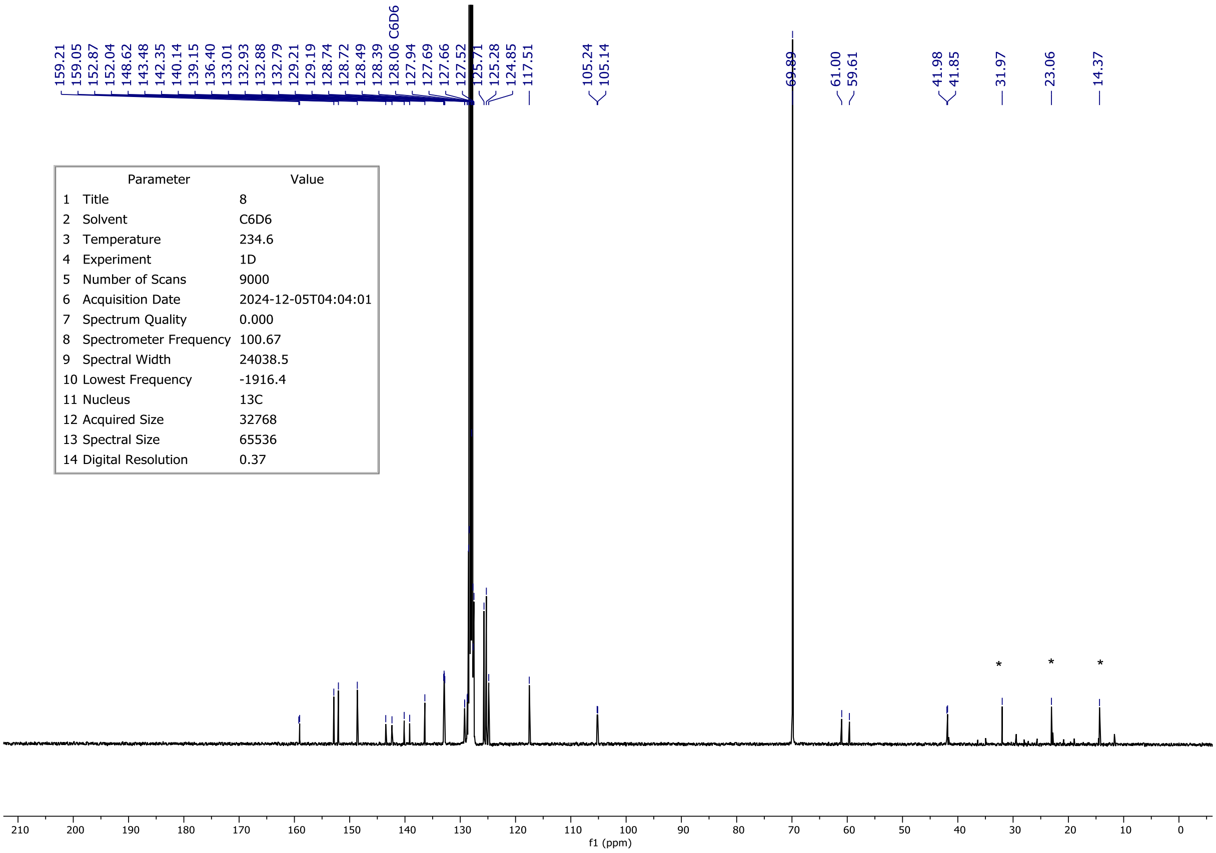


**Figure S71** $^{13}C\{^{1}H\}$ NMR spectrum of compound **8** in $C_6D_6$ (* = residual hexane).

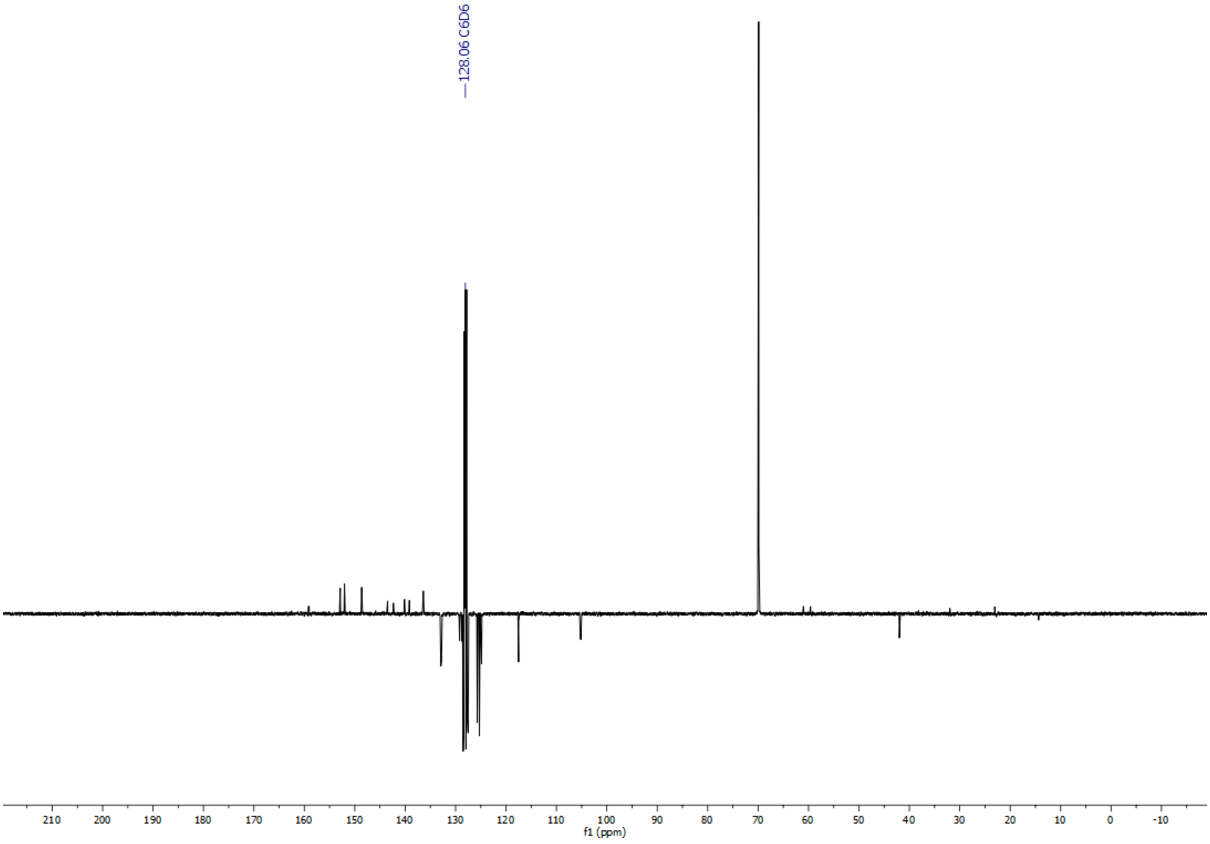


**Figure S72** $^{13}C$ APT NMR spectrum of compound **8** in $C_6D_6$

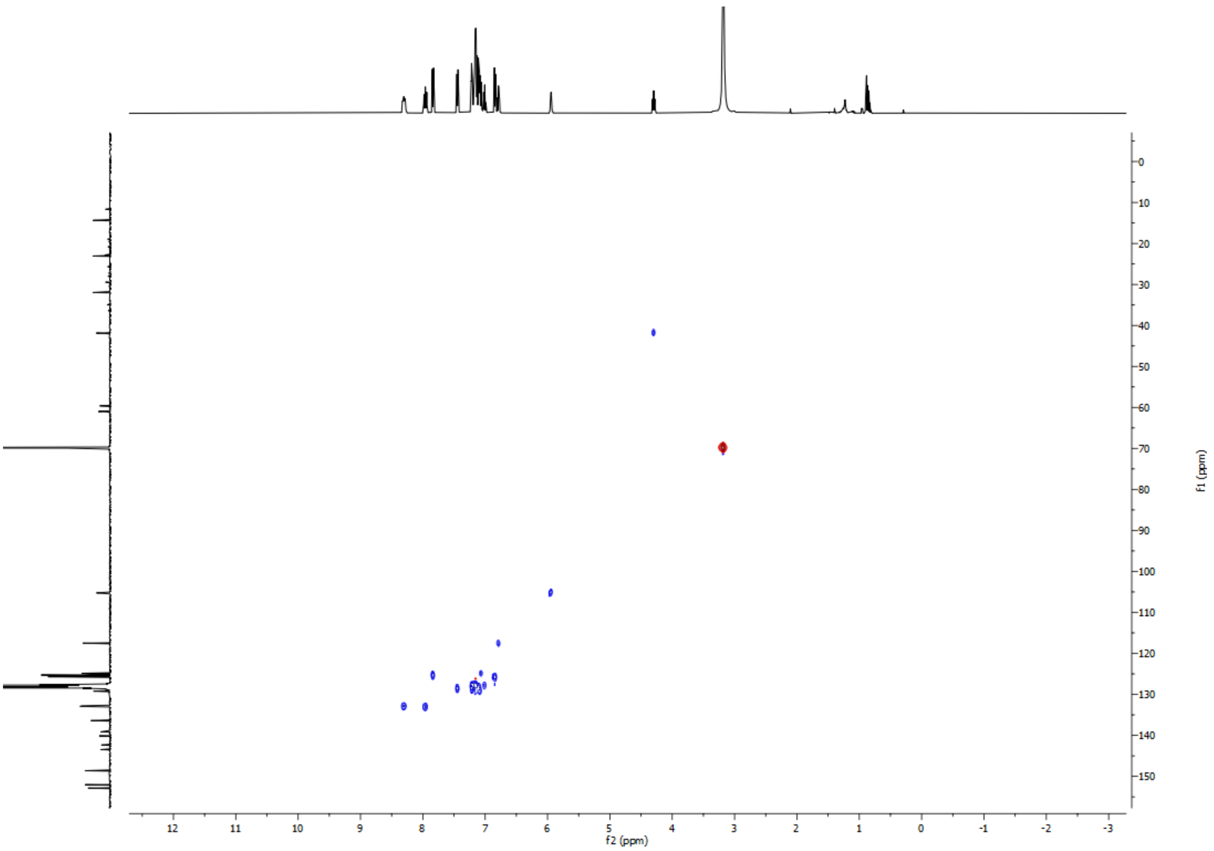


**Figure S73** $^{1}H$-$^{13}C$ HSQC NMR spectrum of compound **8** in $C_6D_6$.

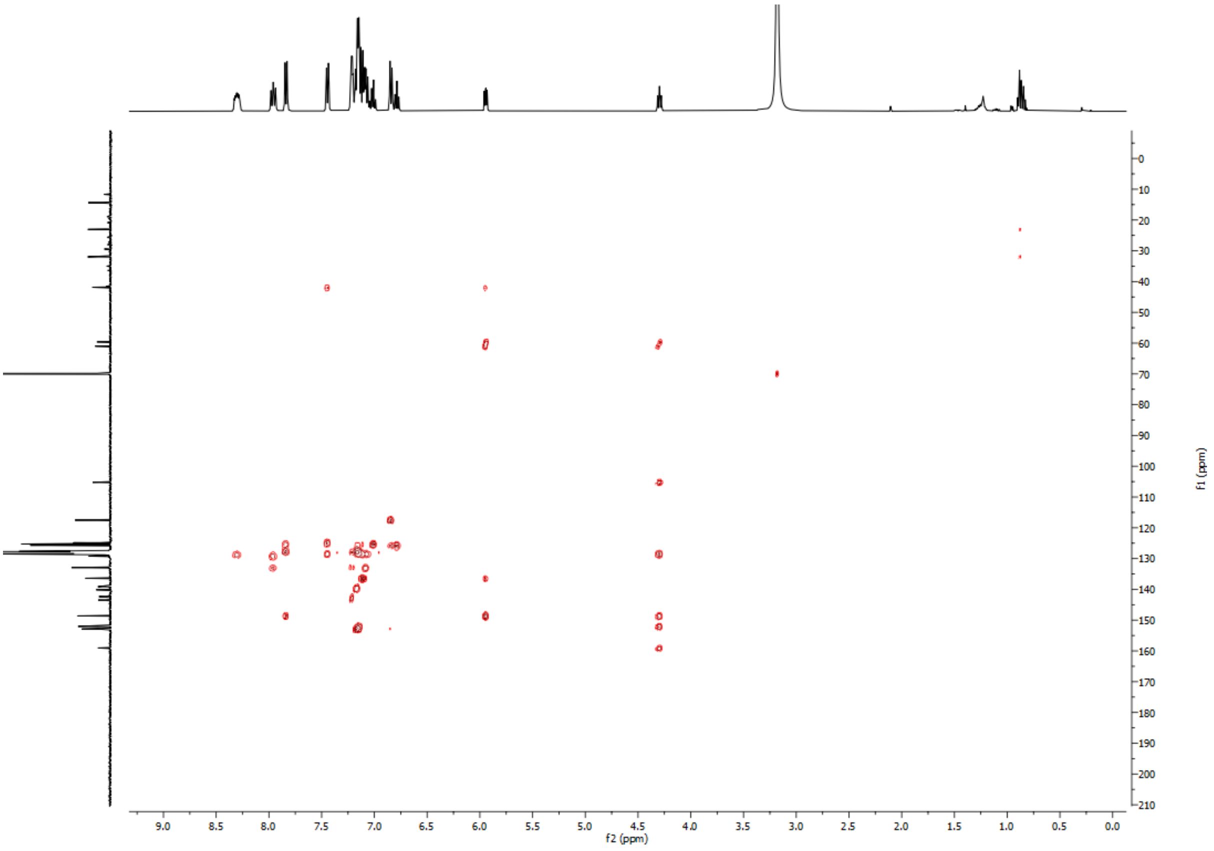


**Figure S74** $^{1}H$-$^{13}C$ HMBC NMR spectrum of compound 8 in $C_6D_6$.

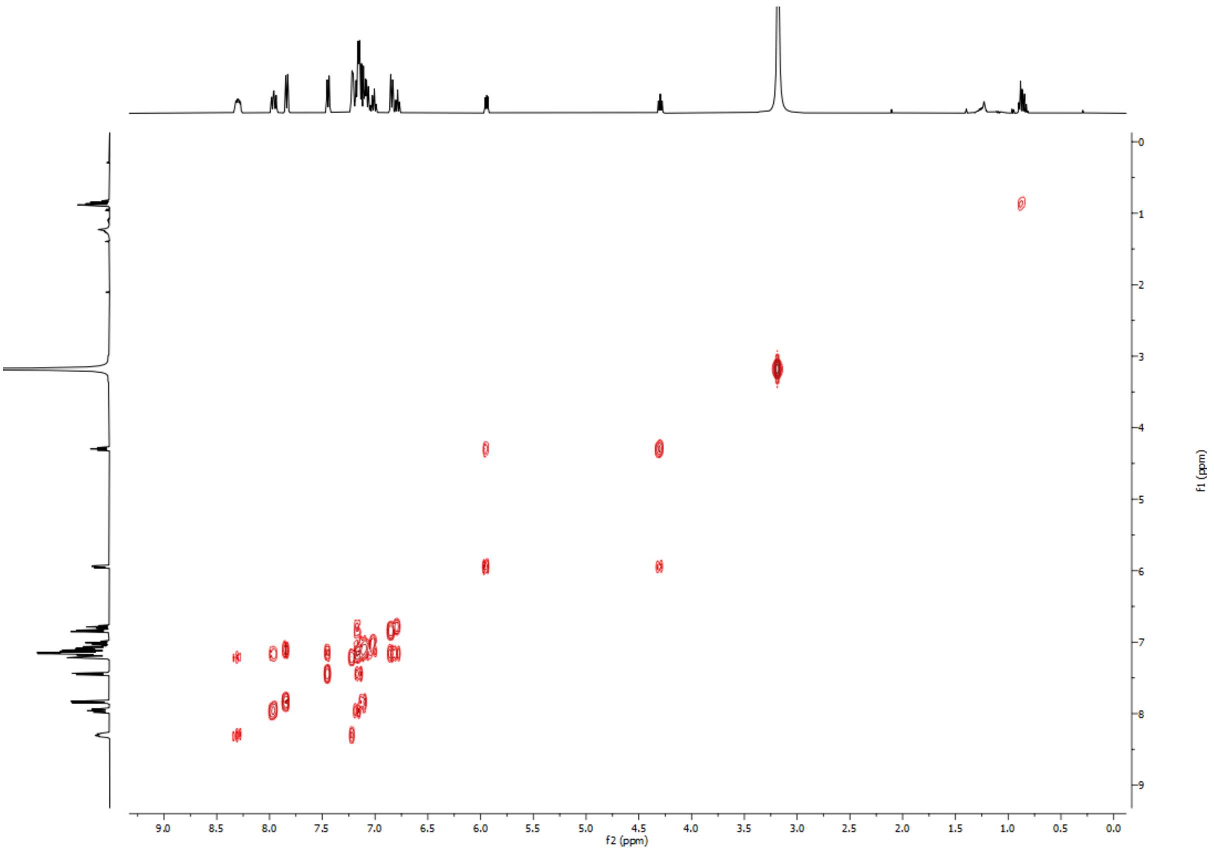


**Figure S75** $^{1}H$-$^{1}H$ COSY NMR spectrum of compound 8 in $C_6D_6$.

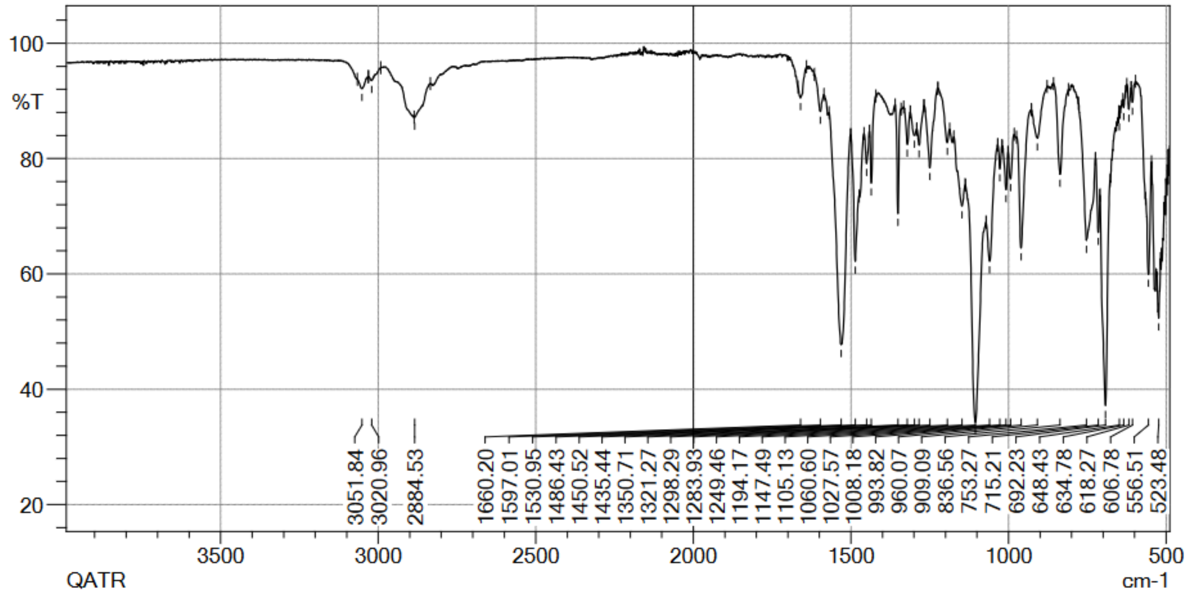


**Figure S76** IR spectrum of compound **8** (solid state).

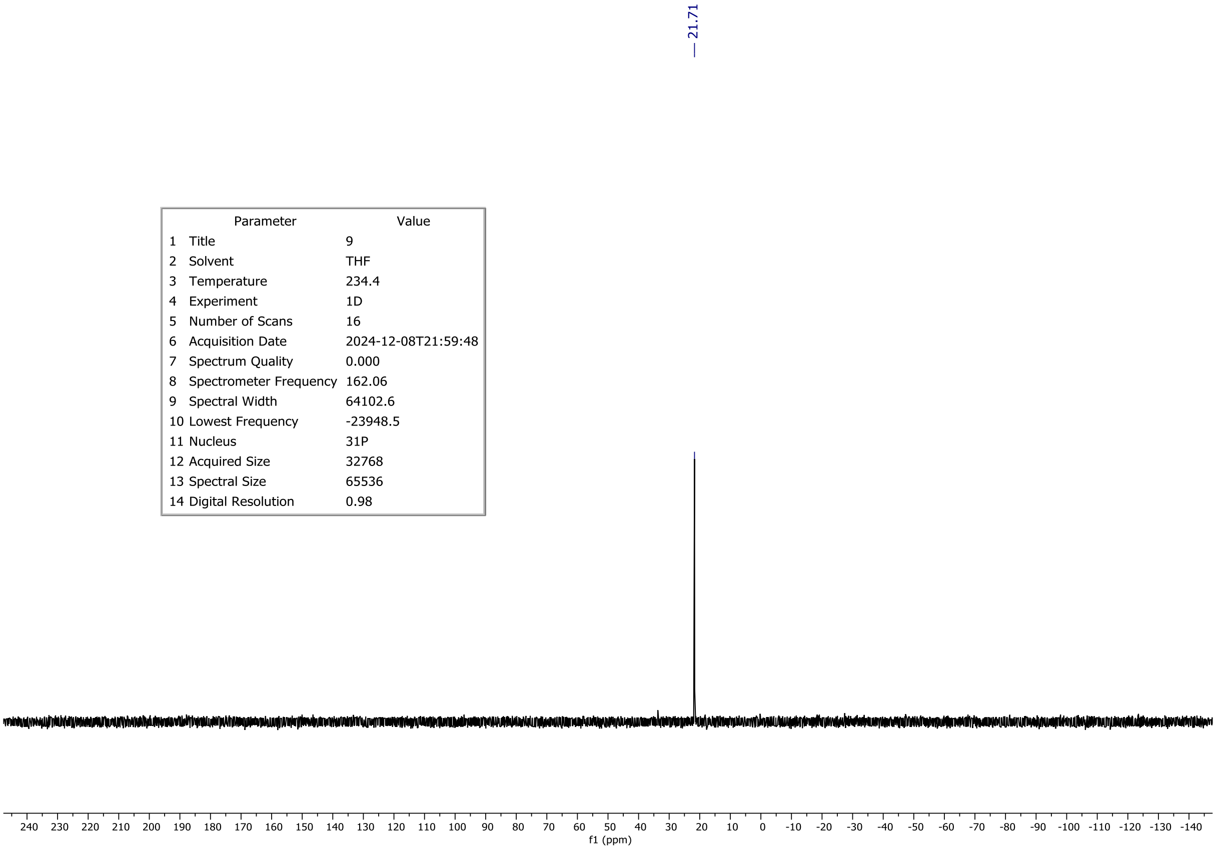


**Figure S77** $^{31}P\{^{1}H\}$ NMR spectrum of compound **9** in THF-$d_8$.

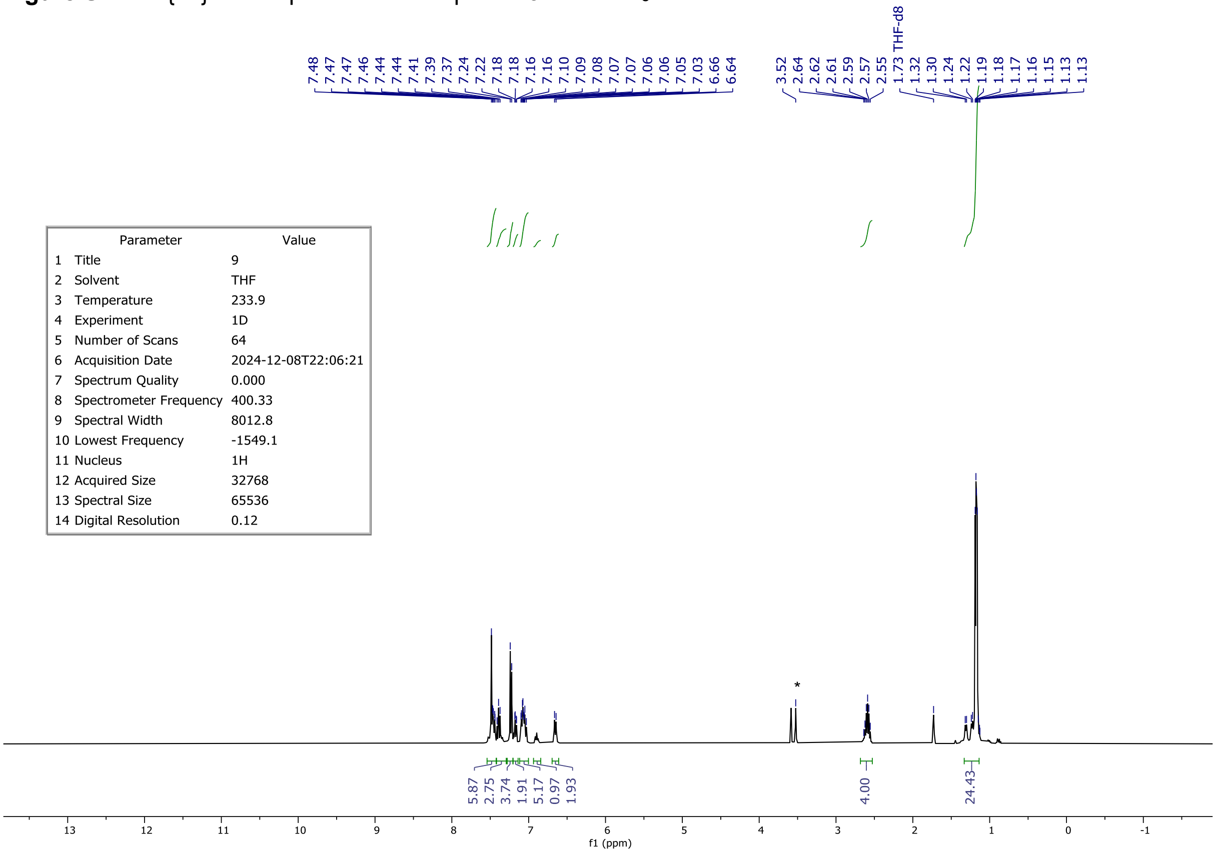


**Figure S78** $^{1}H$ NMR spectrum of compound **9** in THF-$d_8$ (* = residual K(18-c-6)Cl).

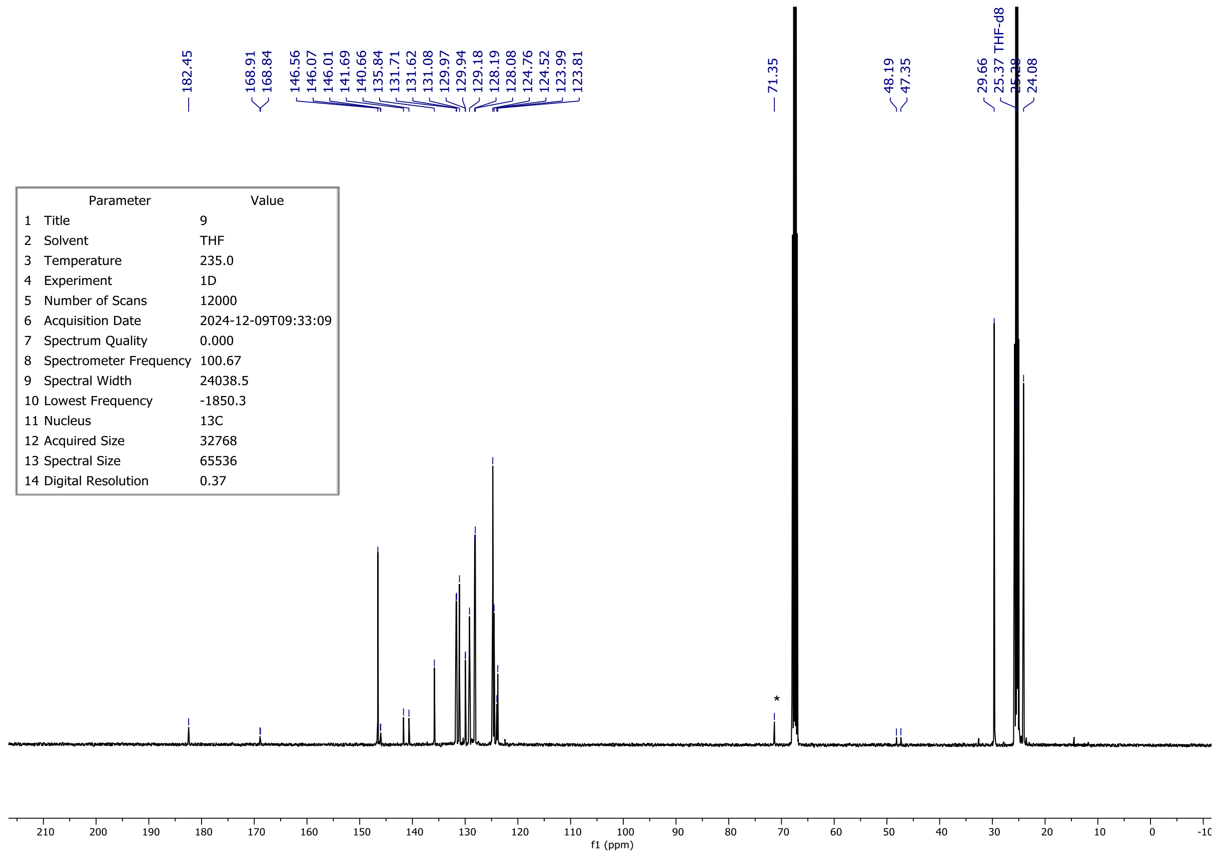


**Figure S79** $^{13}C\{^{1}H\}$ NMR spectrum of compound **9** in THF-$d_8$ (* = residual K(18-c-6)Cl).

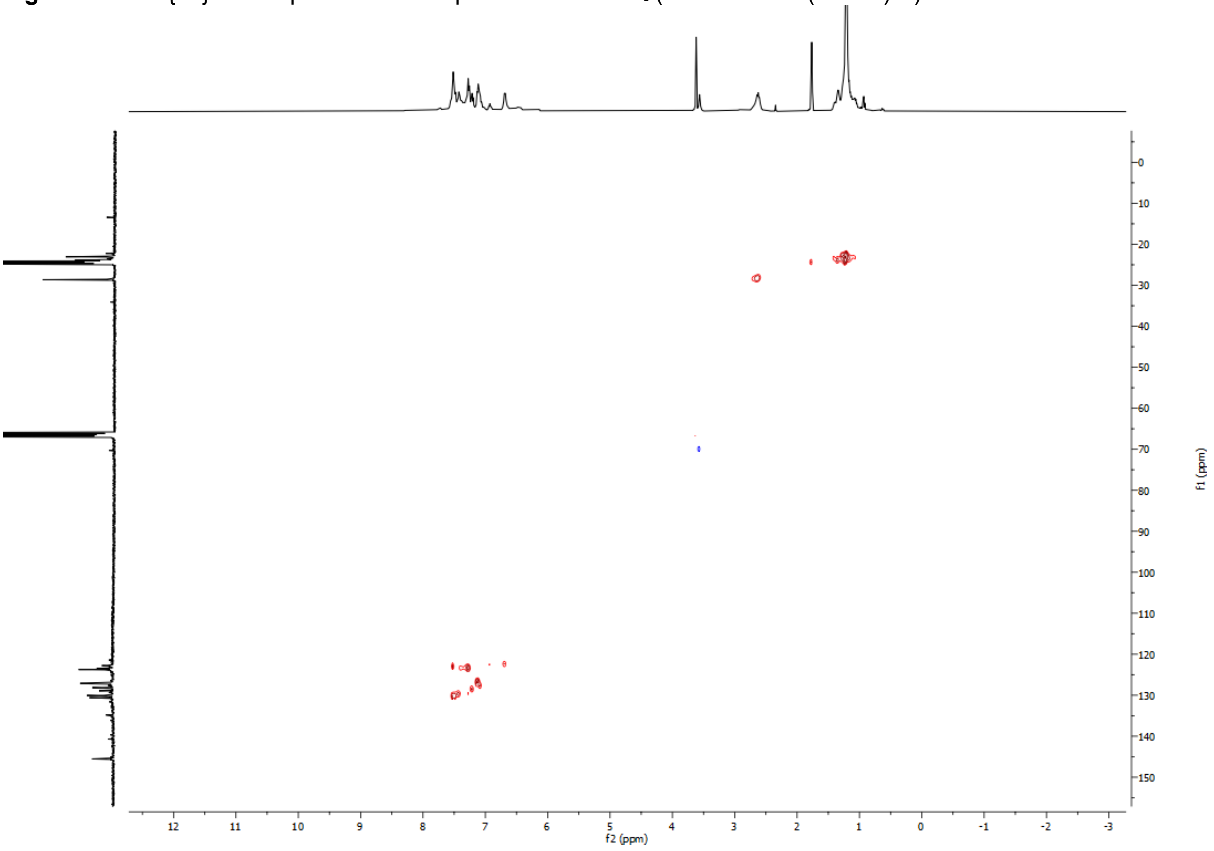


**Figure S80** $^{1}H$-$^{13}C$ HSQC NMR spectrum of compound **9** in THF-$d_8$.

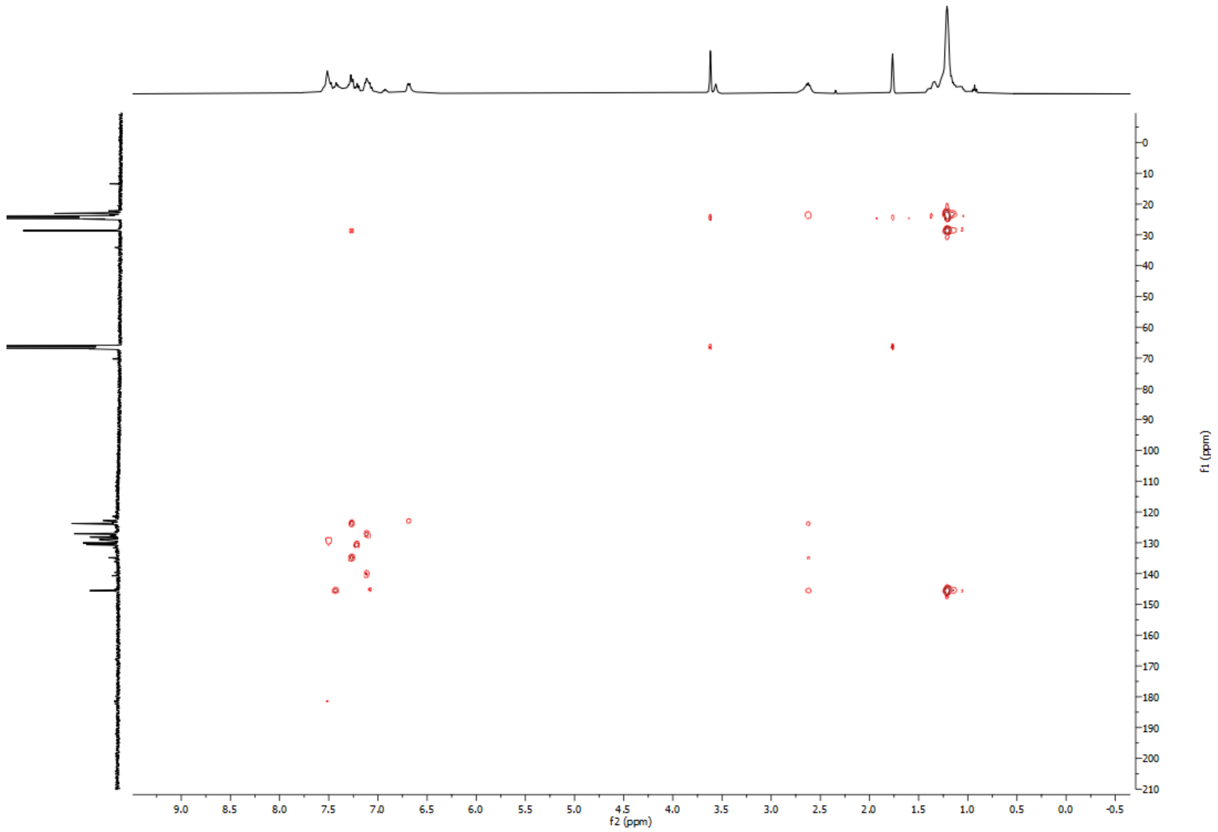


**Figure S81** $^1$H-$^{13}$C HMBC NMR spectrum of compound **9** in THF-$d_8$.

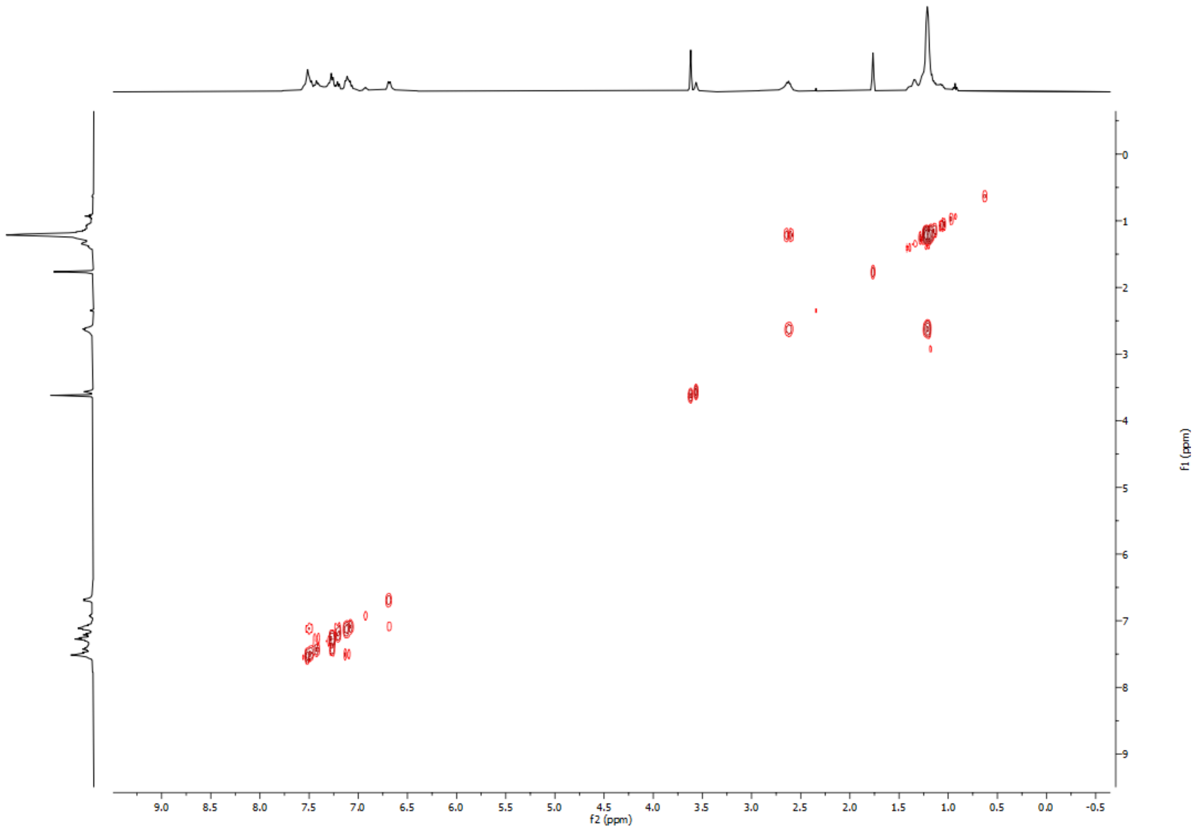


**Figure S82** $^1$H-$^1$H COSY NMR spectrum of compound **9** in THF-$d_8$.

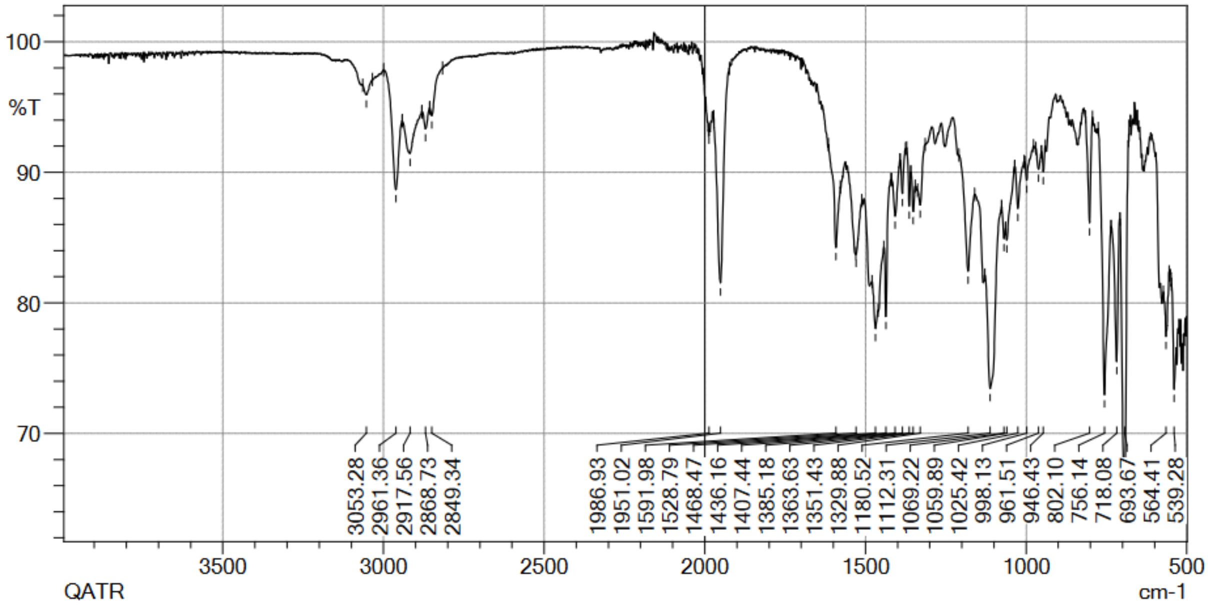


**Figure S83** IR spectrum of compound **9** (solid state).

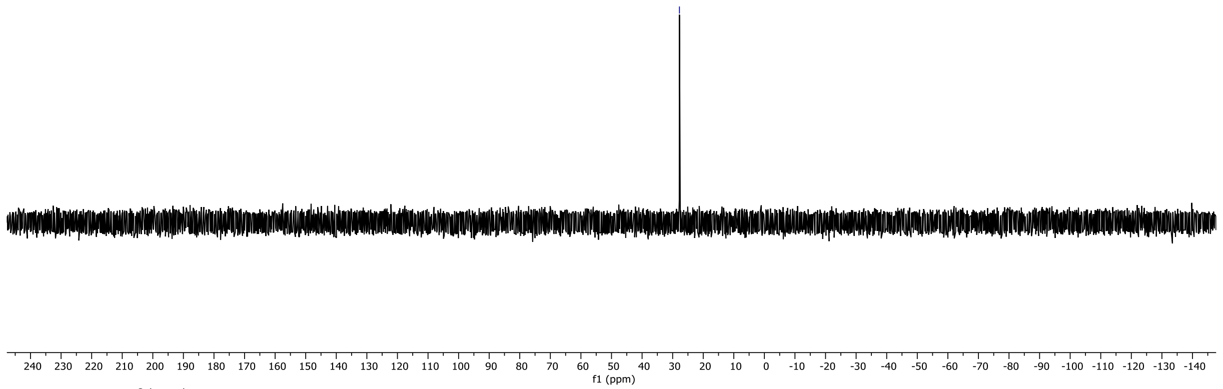


| | Parameter | Value |
|---|---|---|
| 1 | Title | 10 |
| 2 | Solvent | C6D6 |
| 3 | Temperature | 233.6 |
| 4 | Experiment | 1D |
| 5 | Number of Scans | 16 |
| 6 | Acquisition Date | 2024-11-06T18:47:24 |
| 7 | Spectrum Quality | 0.000 |
| 8 | Spectrometer Frequency | 162.06 |
| 9 | Spectral Width | 64102.6 |
| 10 | Lowest Frequency | -23948.5 |
| 11 | Nucleus | 31P |
| 12 | Acquired Size | 32768 |
| 13 | Spectral Size | 65536 |
| 14 | Digital Resolution | 0.98 |

**Figure S84** $^{31}P\{^1H\}$ NMR spectrum of compound **10** in $C_6D_6$.

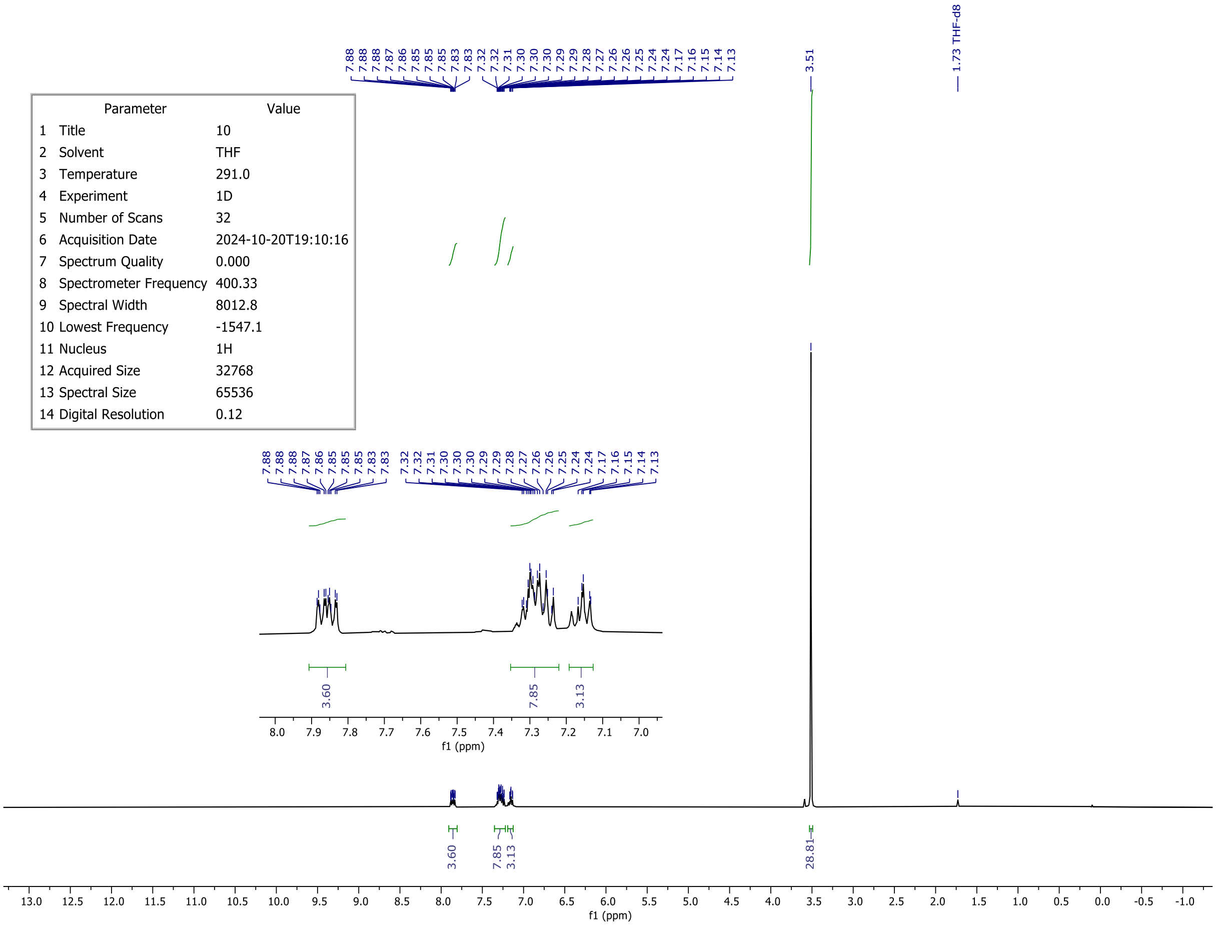


**Figure S85** $^{1}H$ NMR spectrum of compound **10** in THF-$d_8$.

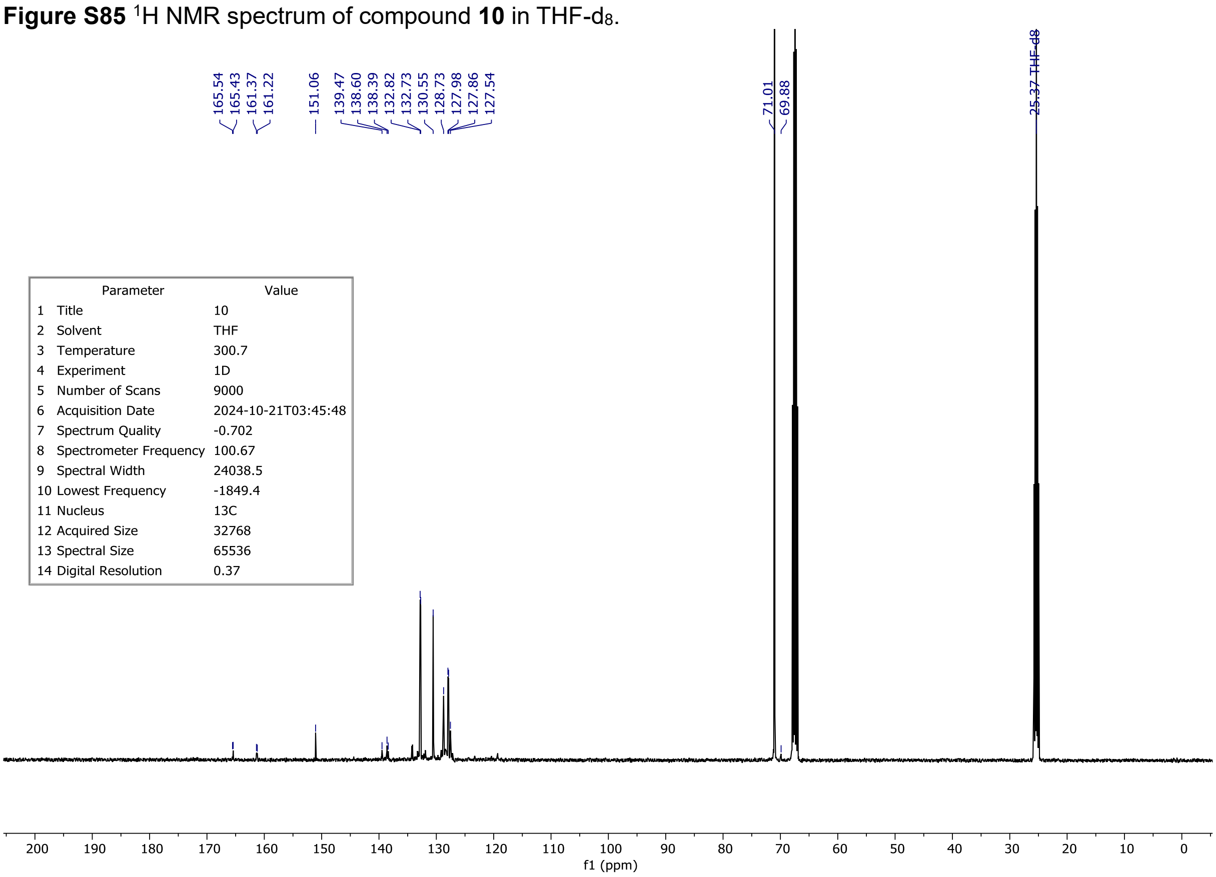


**Figure S86** $^{13}C\{^{1}H\}$ NMR spectrum of compound **10** in THF-$d_8$.

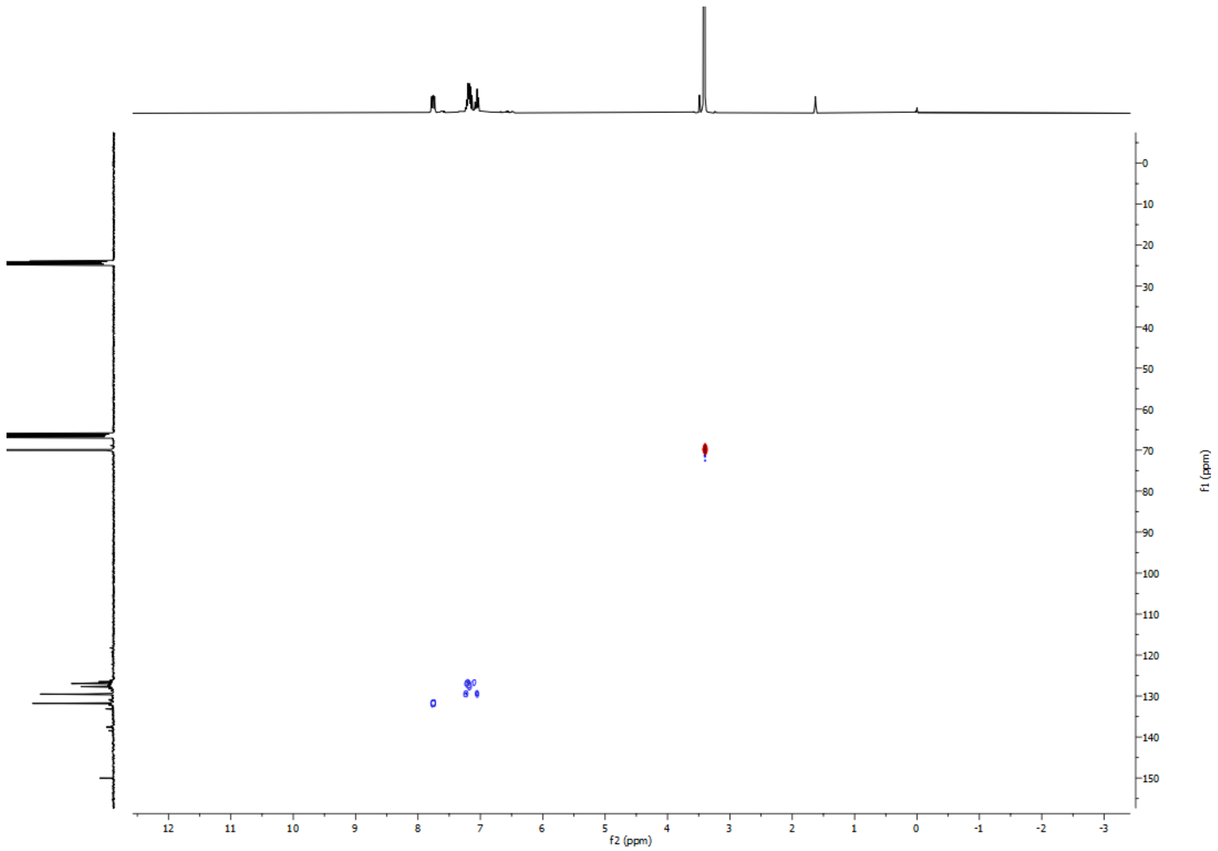

**Figure S87** $^{1}H$-$^{13}C$ HSQC NMR spectrum of compound **10** in THF-$d_8$.

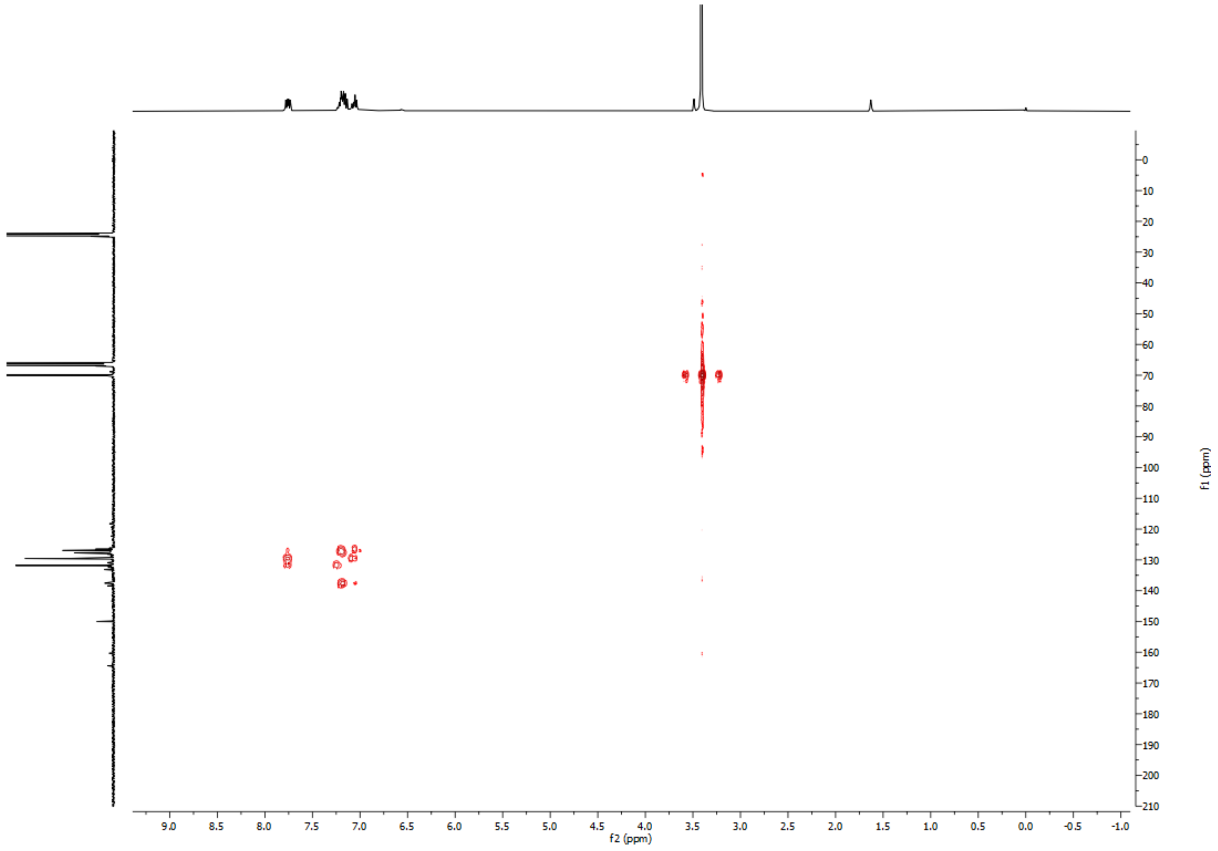

**Figure S88** $^{1}H$-$^{13}C$ HMBC NMR spectrum of compound **10** in THF-$d_8$.

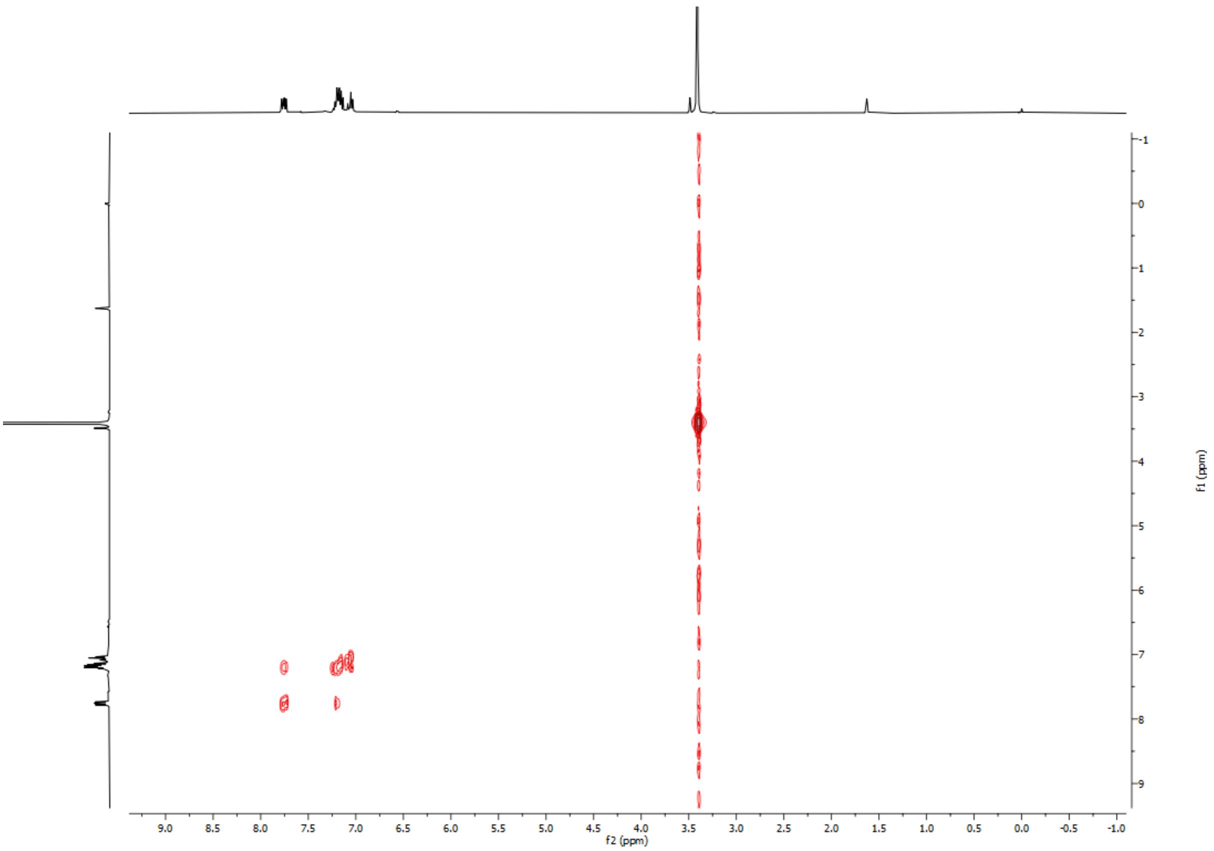


**Figure S89** $^{1}$H-$^{1}$H COSY NMR spectrum of compound **10** in THF-$d_8$.

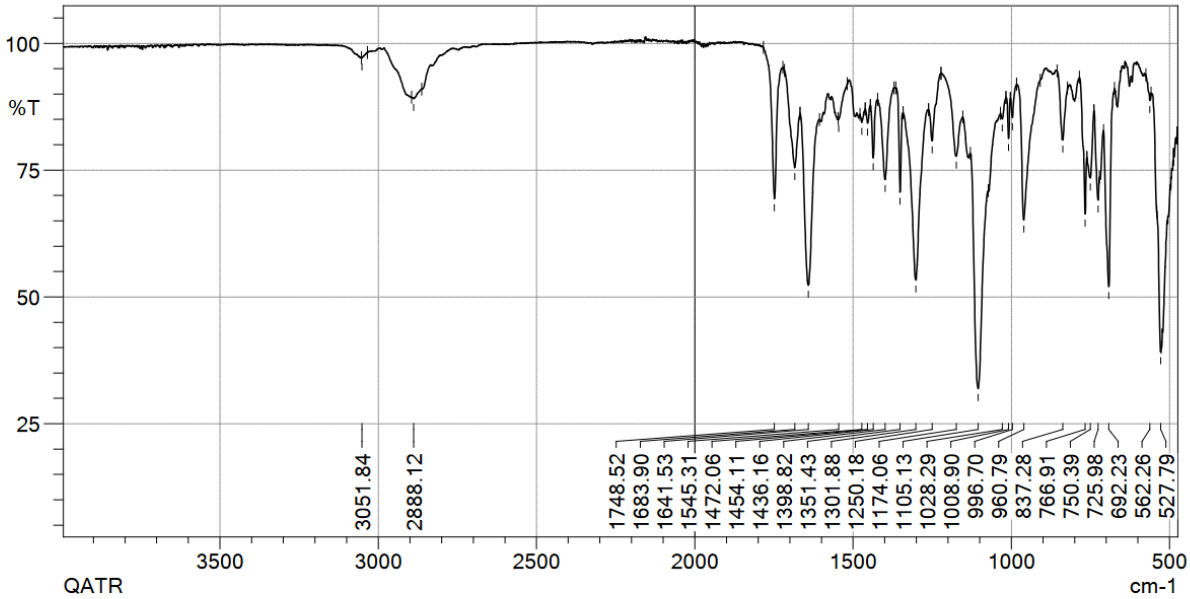


**Figure S90** IR spectrum of compound **10** (solid state).

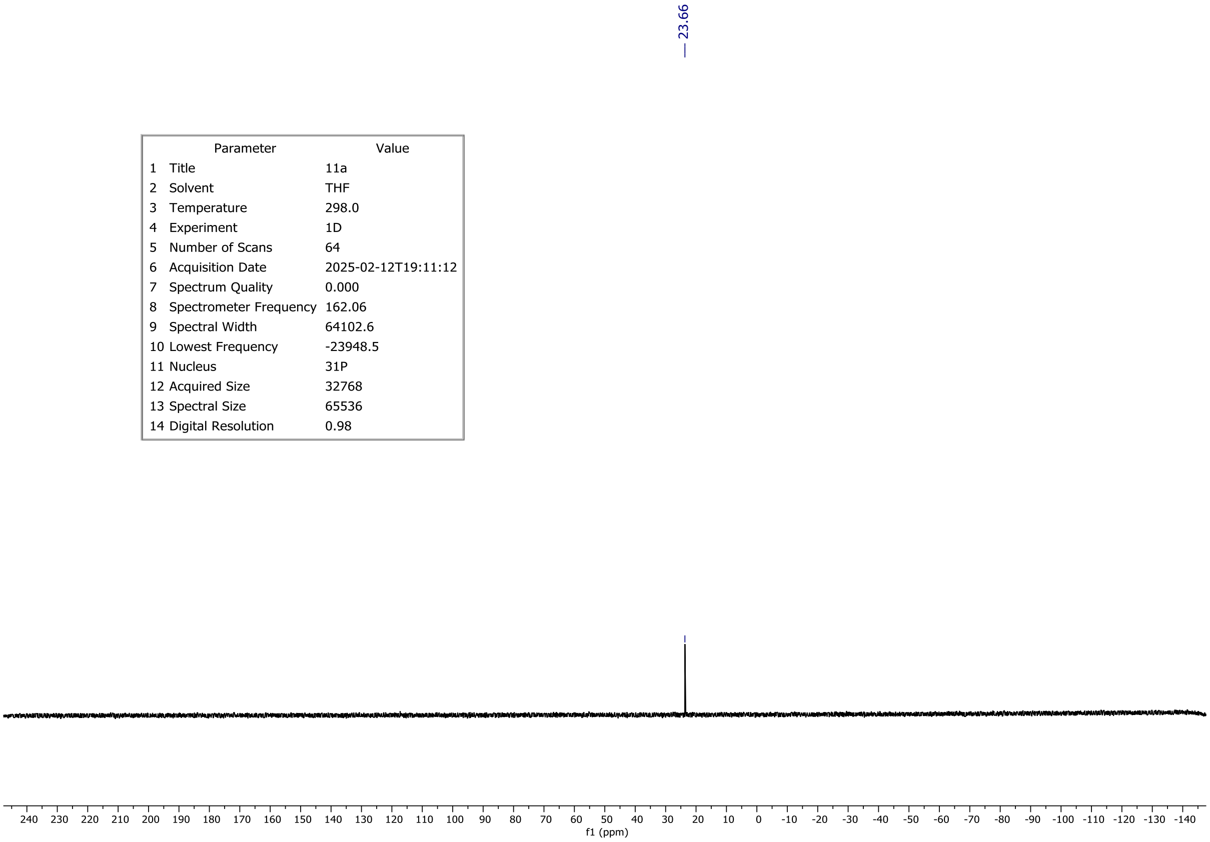


**Figure S91** $^{31}P\{^{1}H\}$ NMR spectrum of compound **11a** in THF-$d_8$.

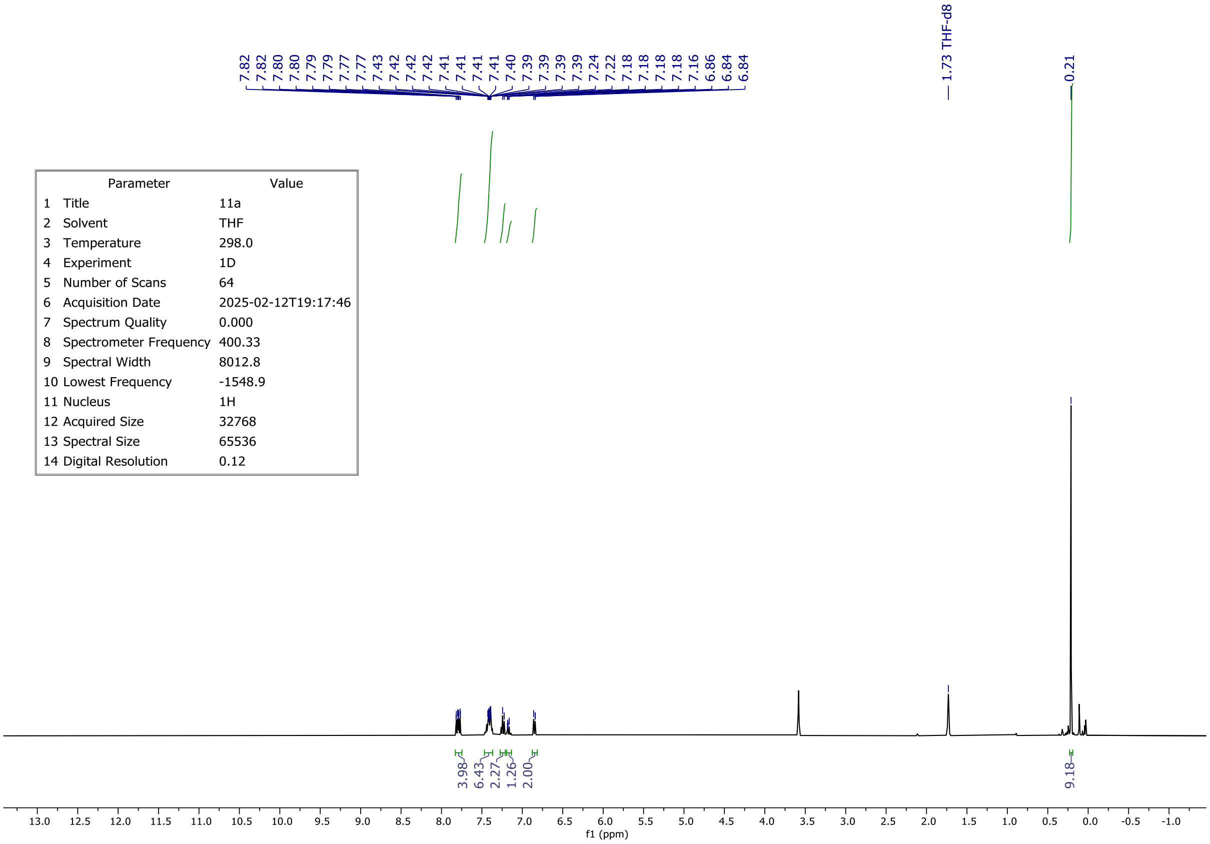


**Figure S92** $^{1}H$ NMR spectrum of compound **11a** in THF-$d_8$.

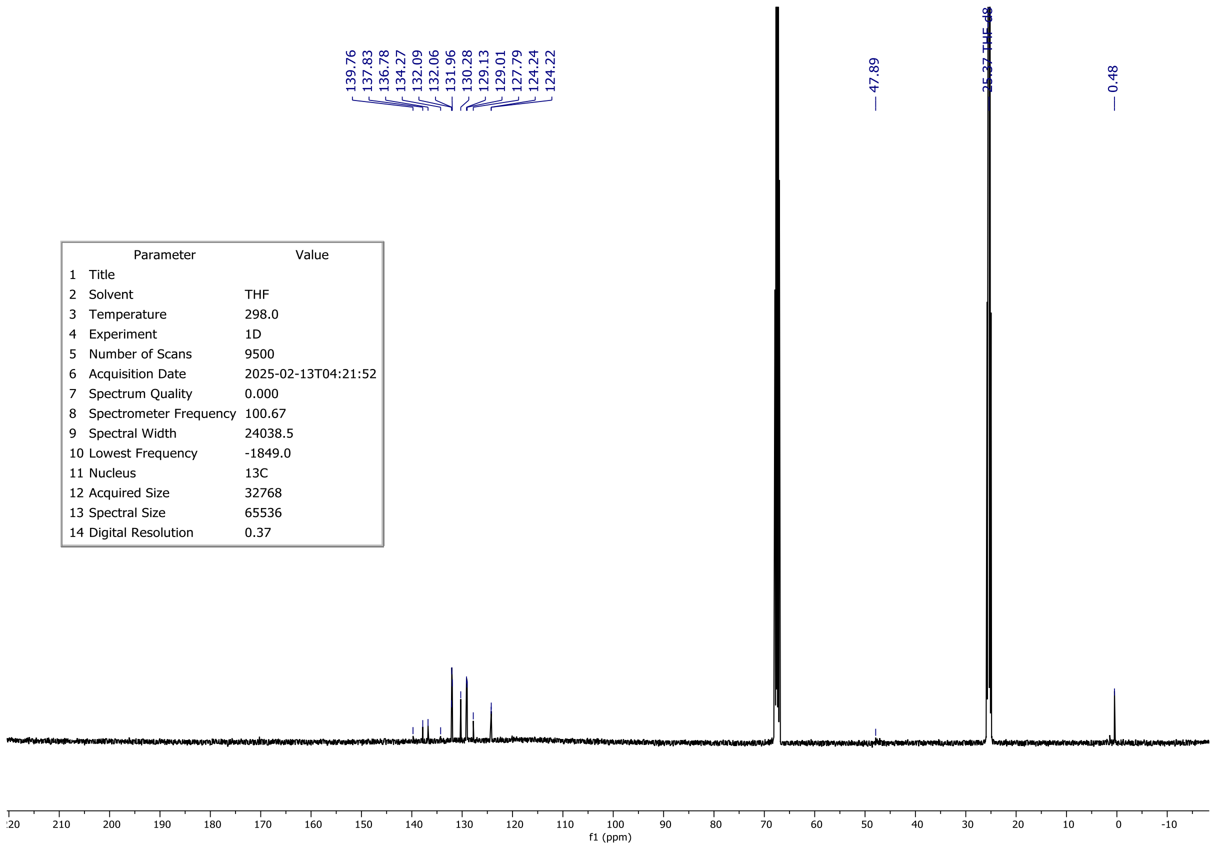


| | Parameter | Value |
|---|---|---|
| 1 | Title | |
| 2 | Solvent | THF |
| 3 | Temperature | 298.0 |
| 4 | Experiment | 1D |
| 5 | Number of Scans | 9500 |
| 6 | Acquisition Date | 2025-02-13T04:21:52 |
| 7 | Spectrum Quality | 0.000 |
| 8 | Spectrometer Frequency | 100.67 |
| 9 | Spectral Width | 24038.5 |
| 10 | Lowest Frequency | -1849.0 |
| 11 | Nucleus | 13C |
| 12 | Acquired Size | 32768 |
| 13 | Spectral Size | 65536 |
| 14 | Digital Resolution | 0.37 |

**Figure S93** $^{13}C\{^{1}H\}$ NMR spectrum of compound **11a** in THF-d$_8$.

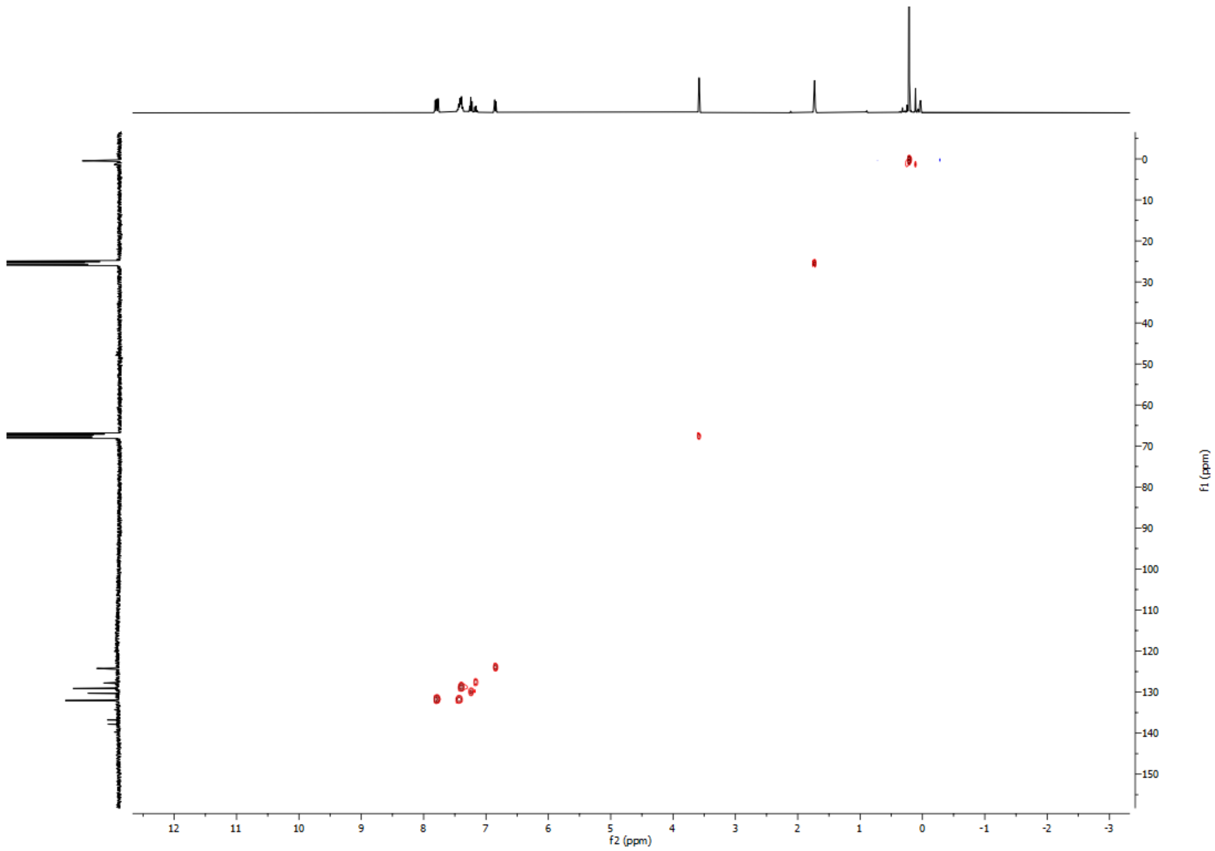


**Figure S94** $^{1}H$-$^{13}C$ HSQC NMR spectrum of compound **11a** in THF-d$_8$.

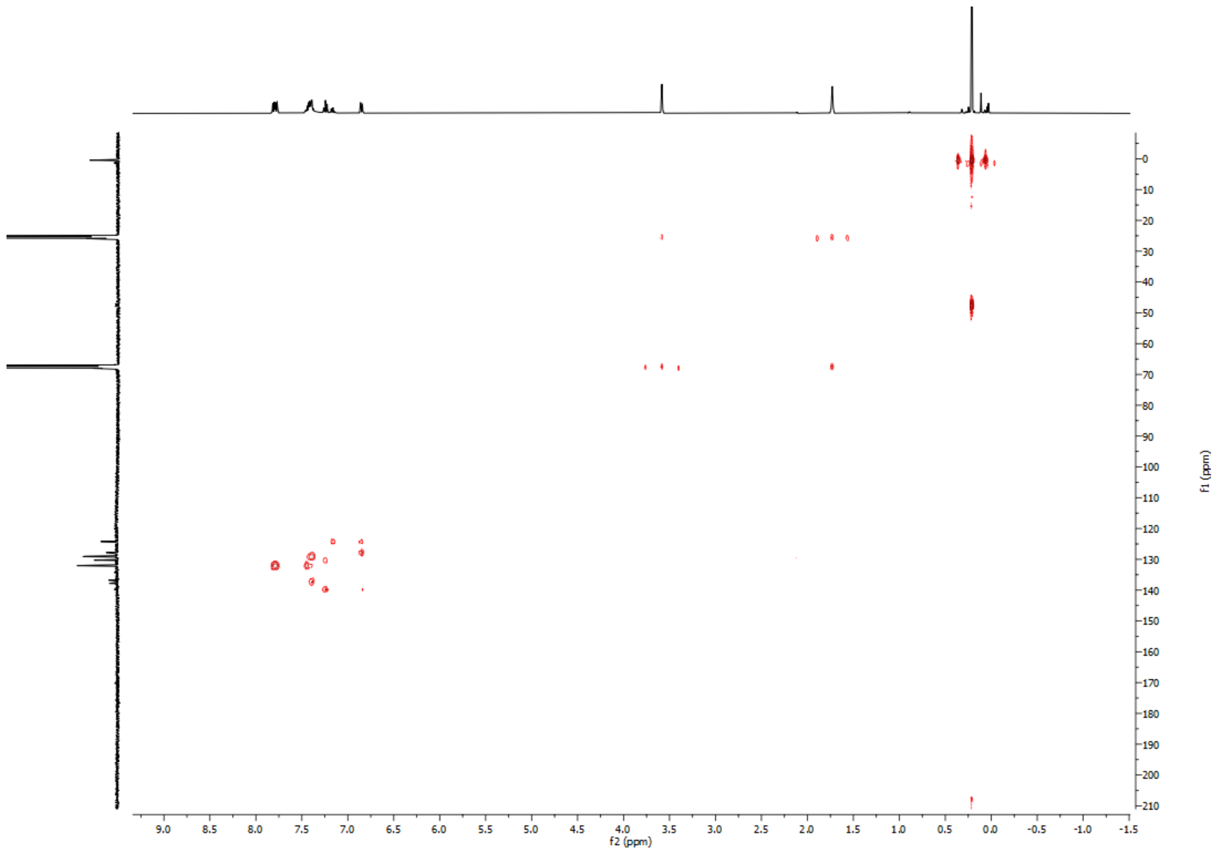


**Figure S95** $^{1}H$-$^{13}C$ HMBC NMR spectrum of compound **11a** in THF-$d_8$.

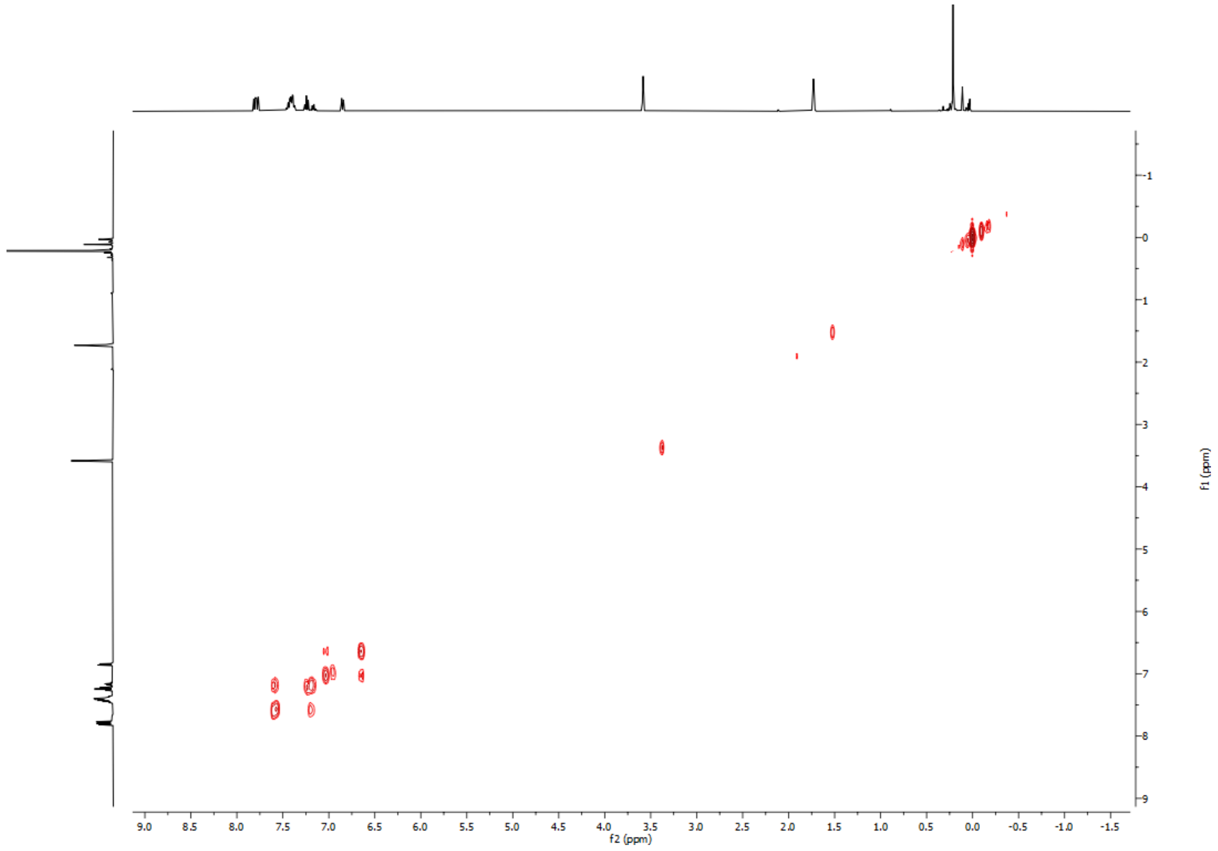


**Figure S96** $^{1}H$-$^{1}H$ COSY NMR spectrum of compound **11a** in THF-$d_8$.

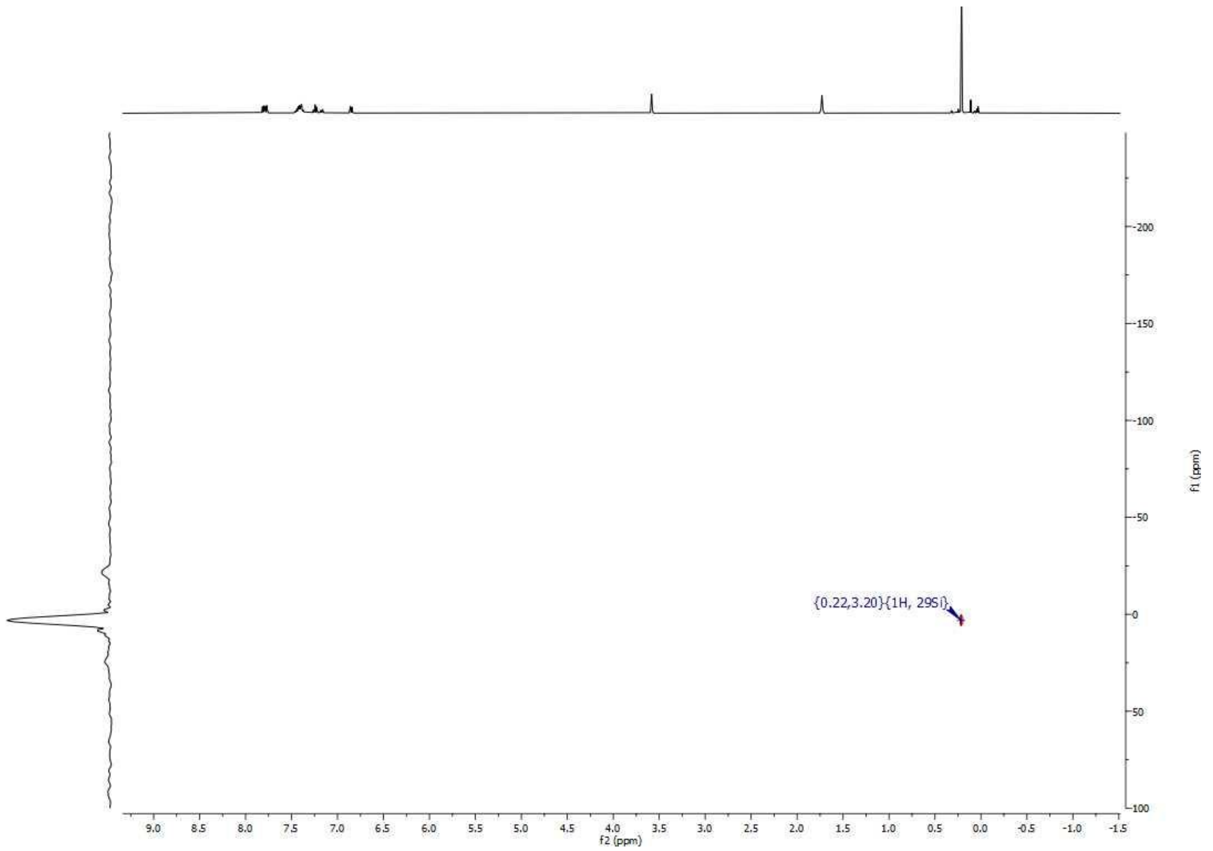


**Figure S97** $^{1}H$-$^{29}Si$ HMBC spectrum of compound **11a** in THF-$d_8$

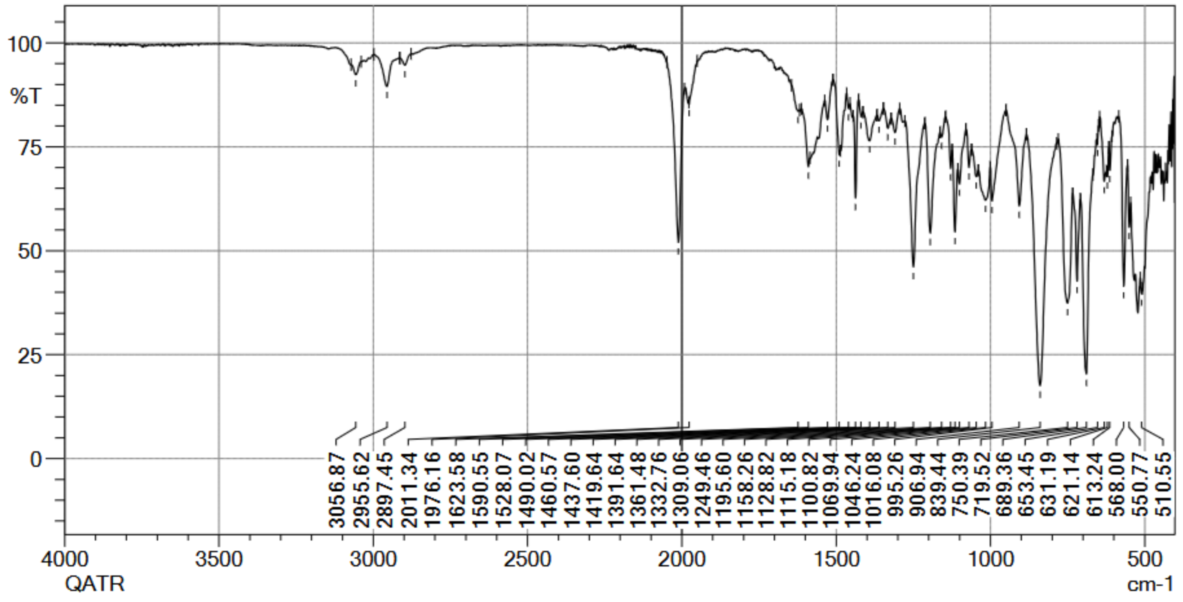


**Figure S98** IR spectrum of compound **11a** (solid state).

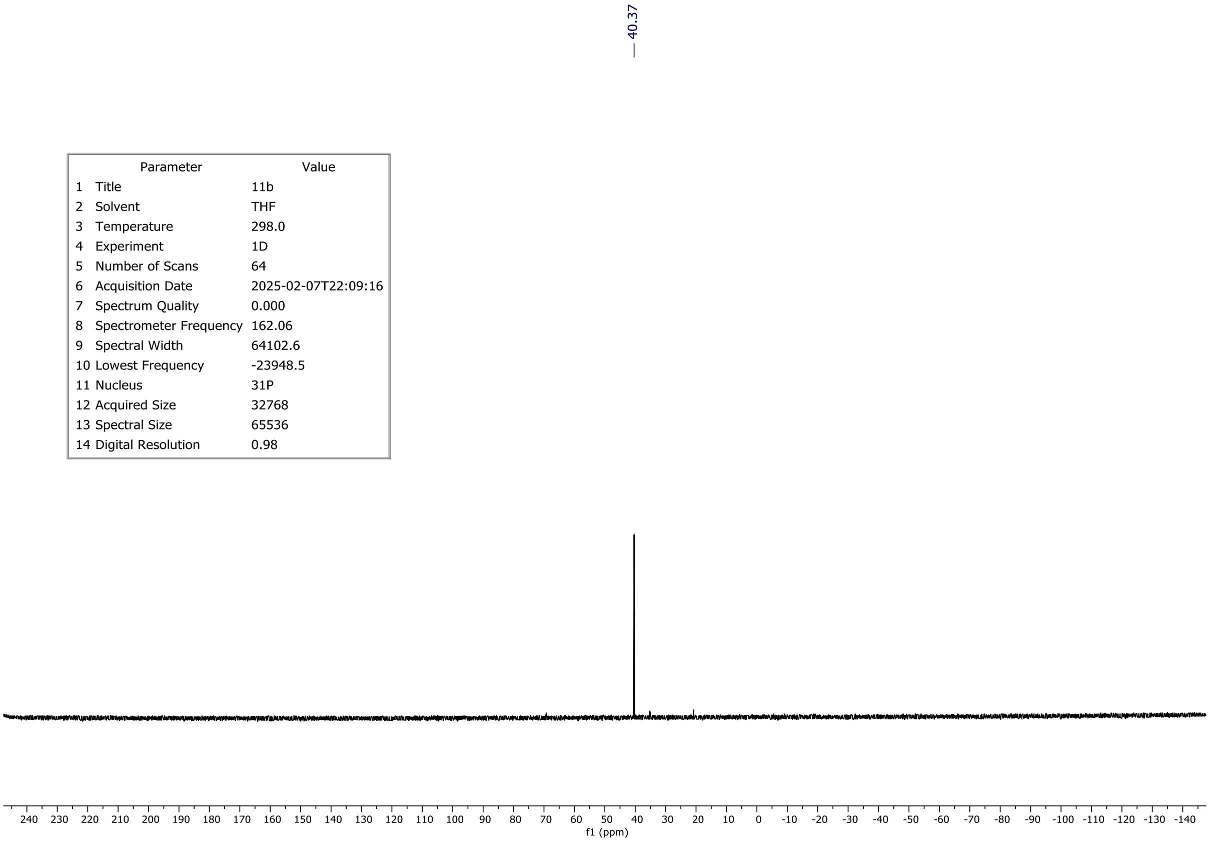


**Figure S99** $^{31}P\{^{1}H\}$ NMR spectrum of compound **11b** in THF-d8.

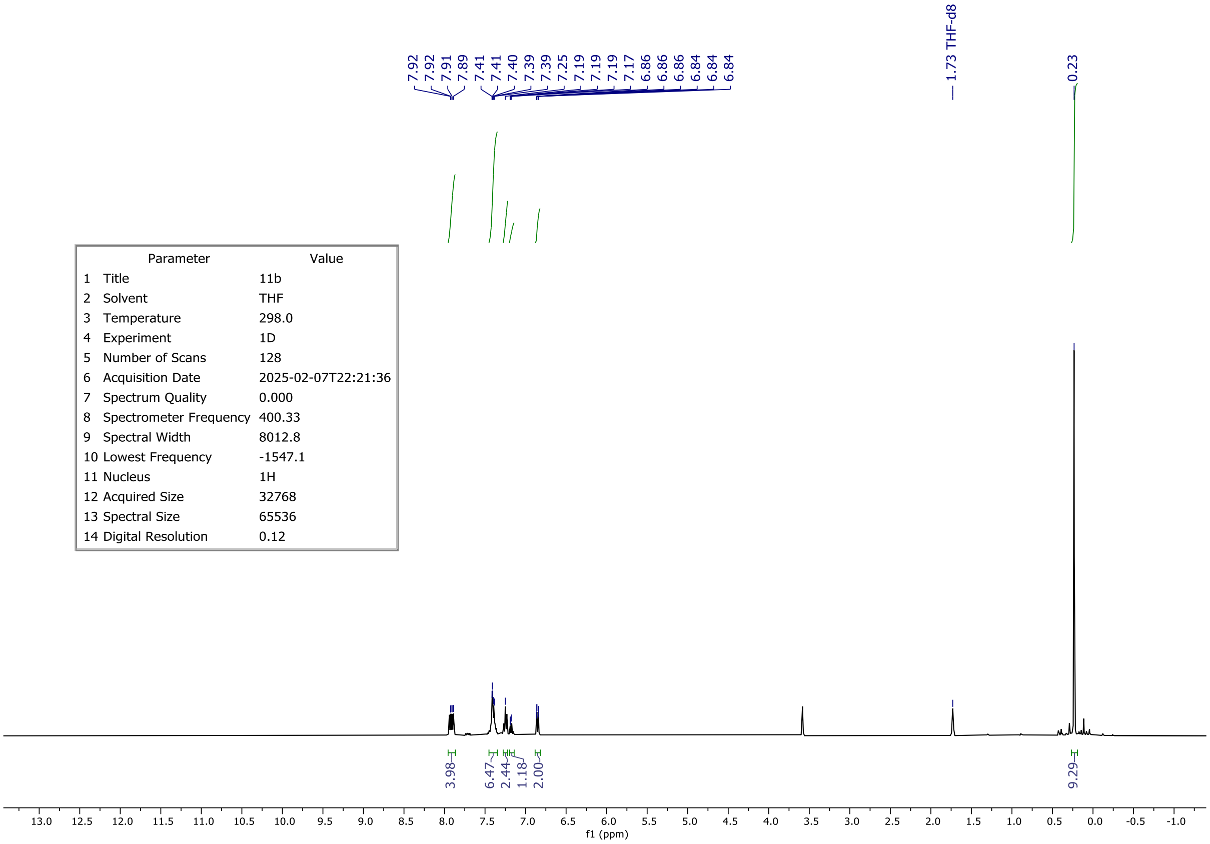


**Figure S100** $^{1}H$ NMR spectrum of compound **11b** in THF-d8.

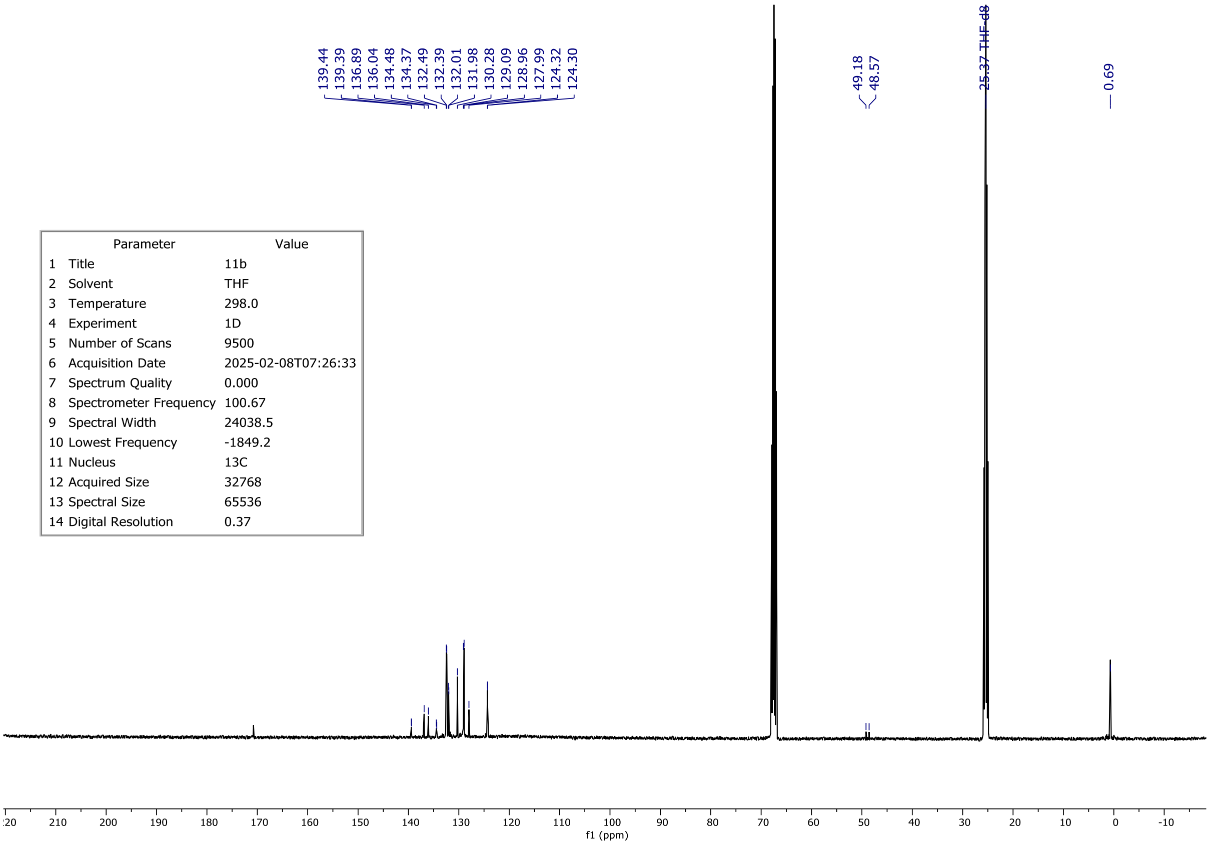


**Figure S101** $^{13}C\{^{1}H\}$ NMR spectrum of compound **11b** in THF-d$_8$.

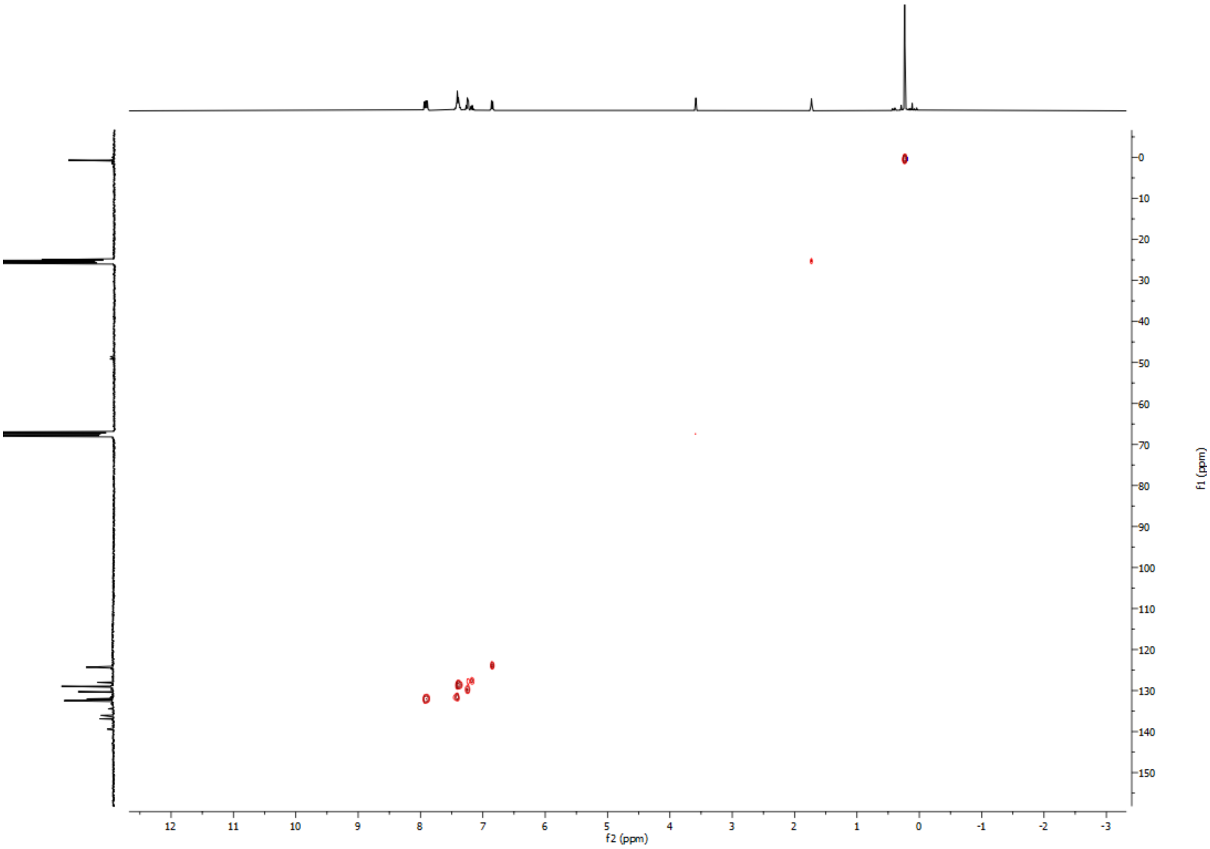


**Figure S102** $^{1}H$-$^{13}C$ HSQC NMR spectrum of compound **11b** in THF-d$_8$.

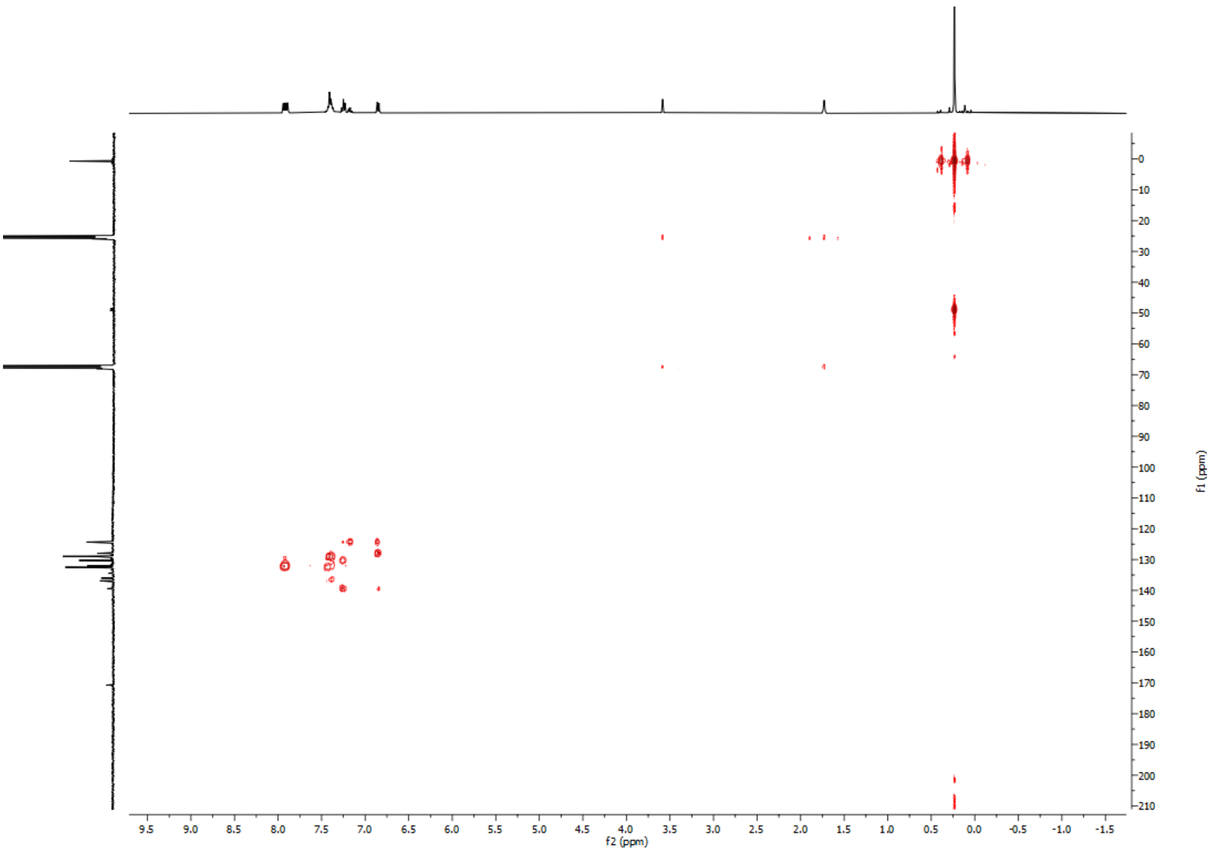


**Figure S103** $^{1}H$-$^{13}C$ HMBC NMR spectrum of compound **11b** in THF-$d_8$.

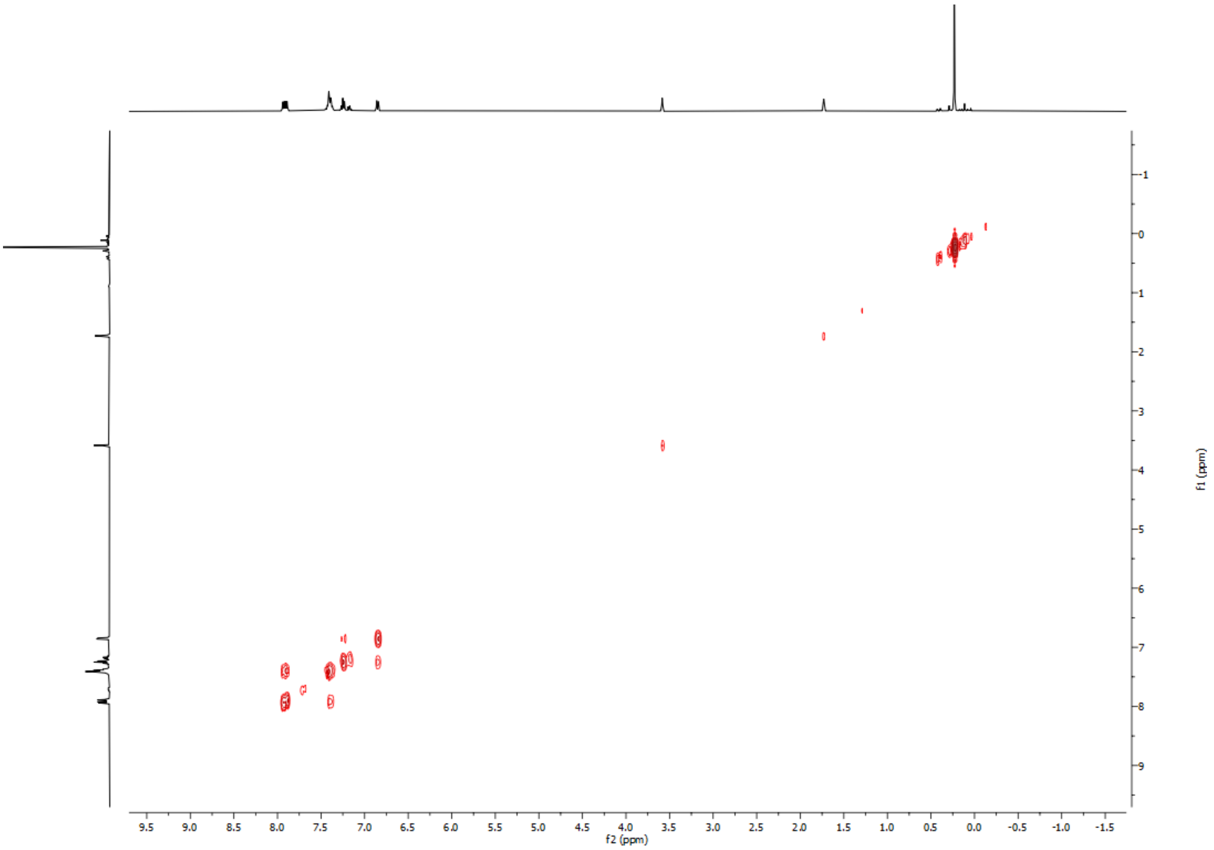


**Figure S104** $^{1}H$-$^{1}H$ COSY NMR spectrum of compound **11b** in THF-$d_8$.

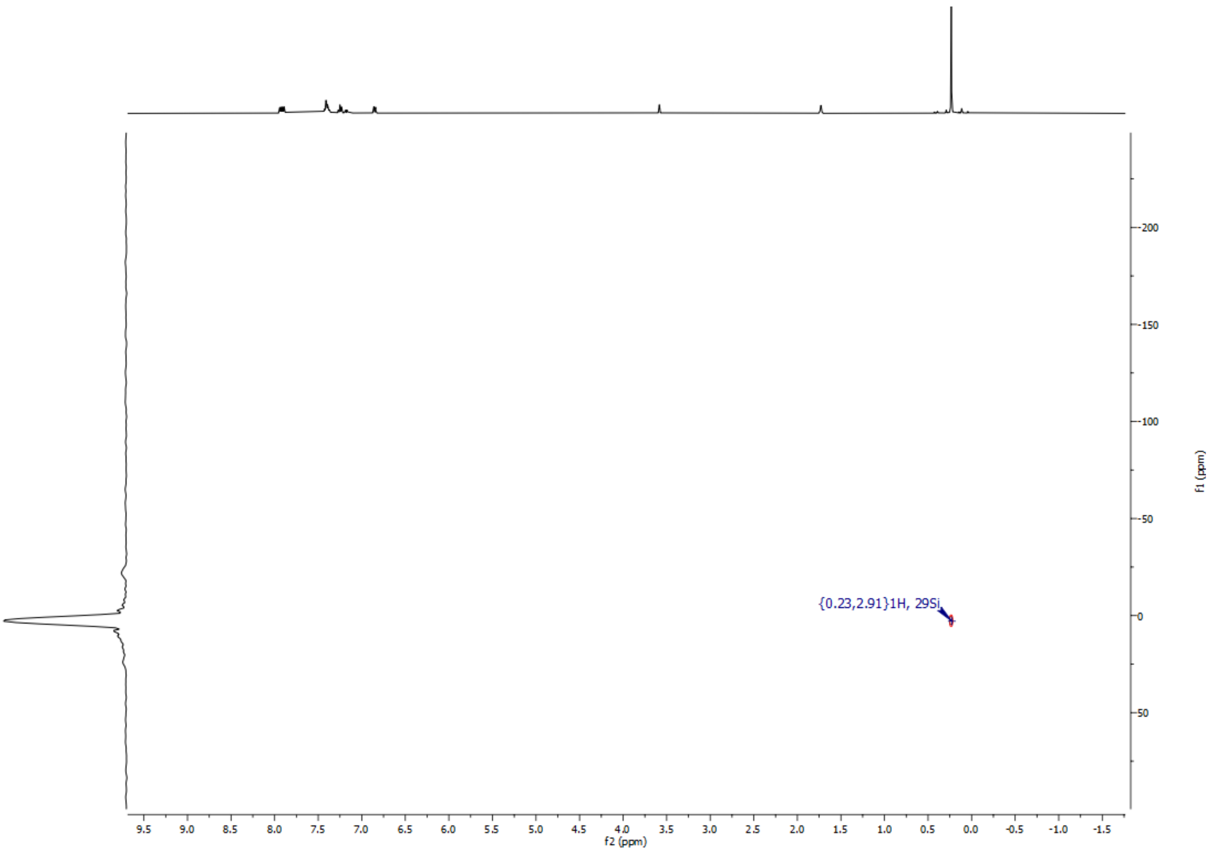


**Figure S105** $^{1}H$-$^{29}Si$ HMBC spectrum of compound **11b** in THF-$d_8$.

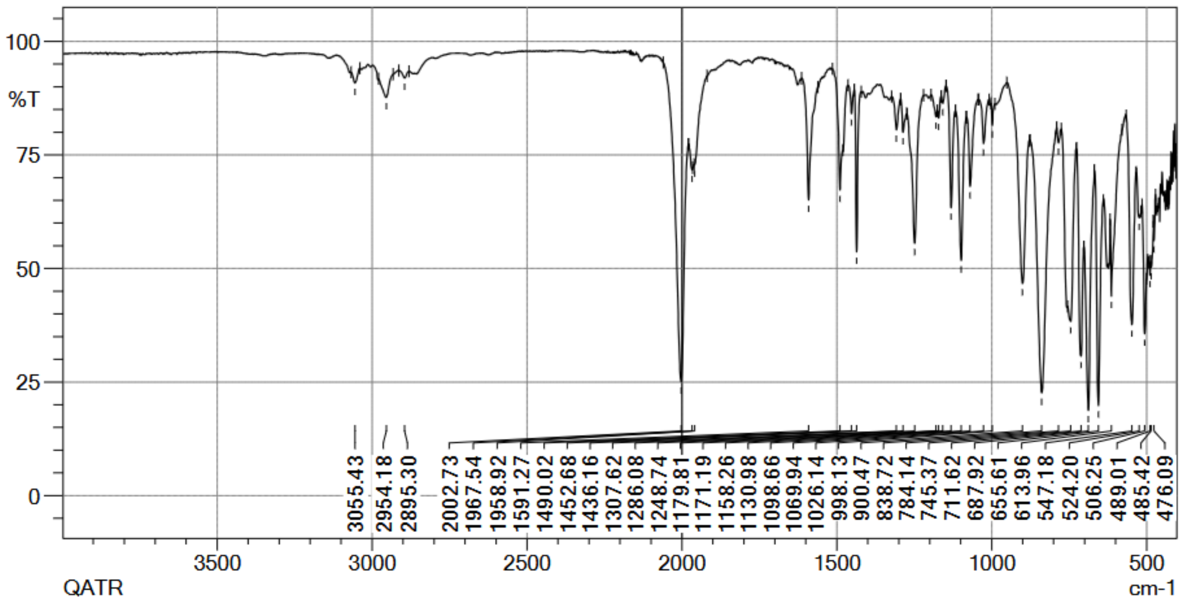


**Figure S106** IR spectrum of compound **11b** (solid state).

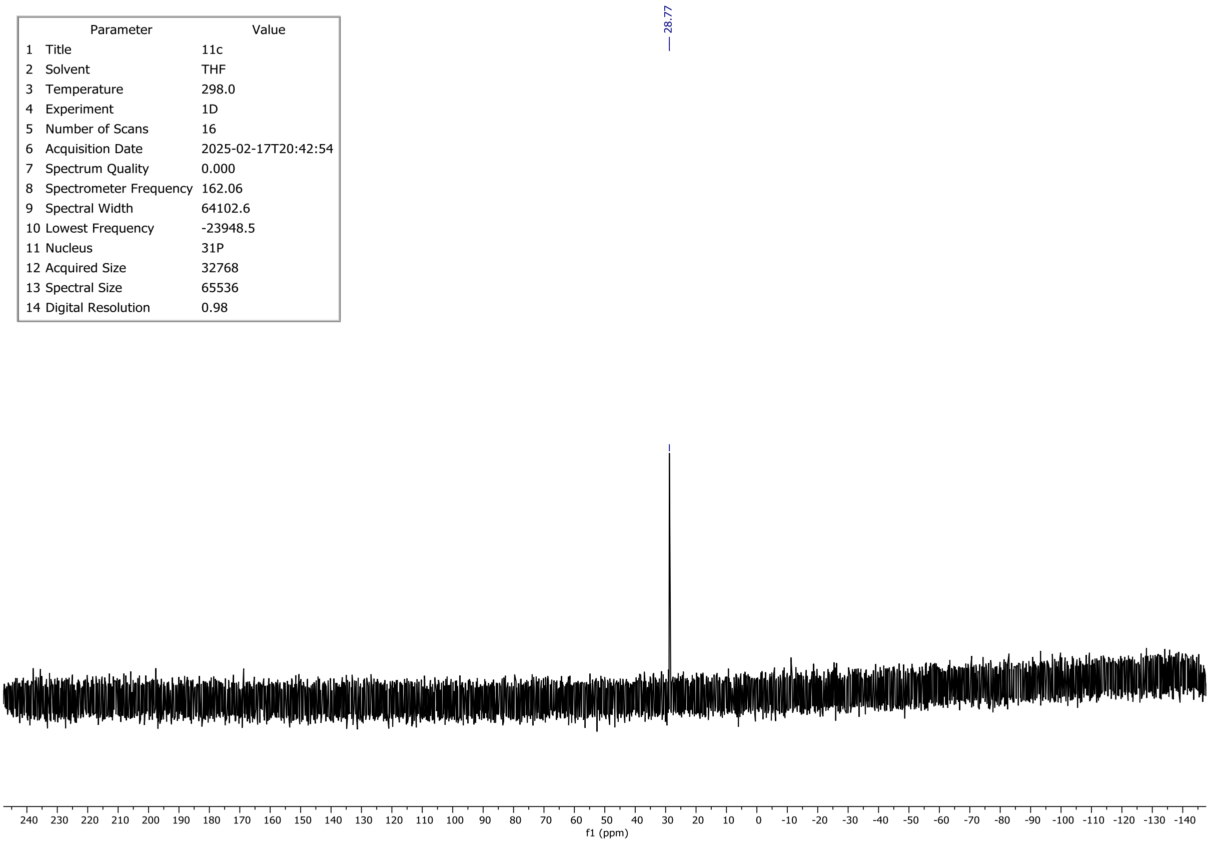


**Figure S107** $^{31}P\{^{1}H\}$ NMR spectrum of compound **11c** in THF-$d_8$.

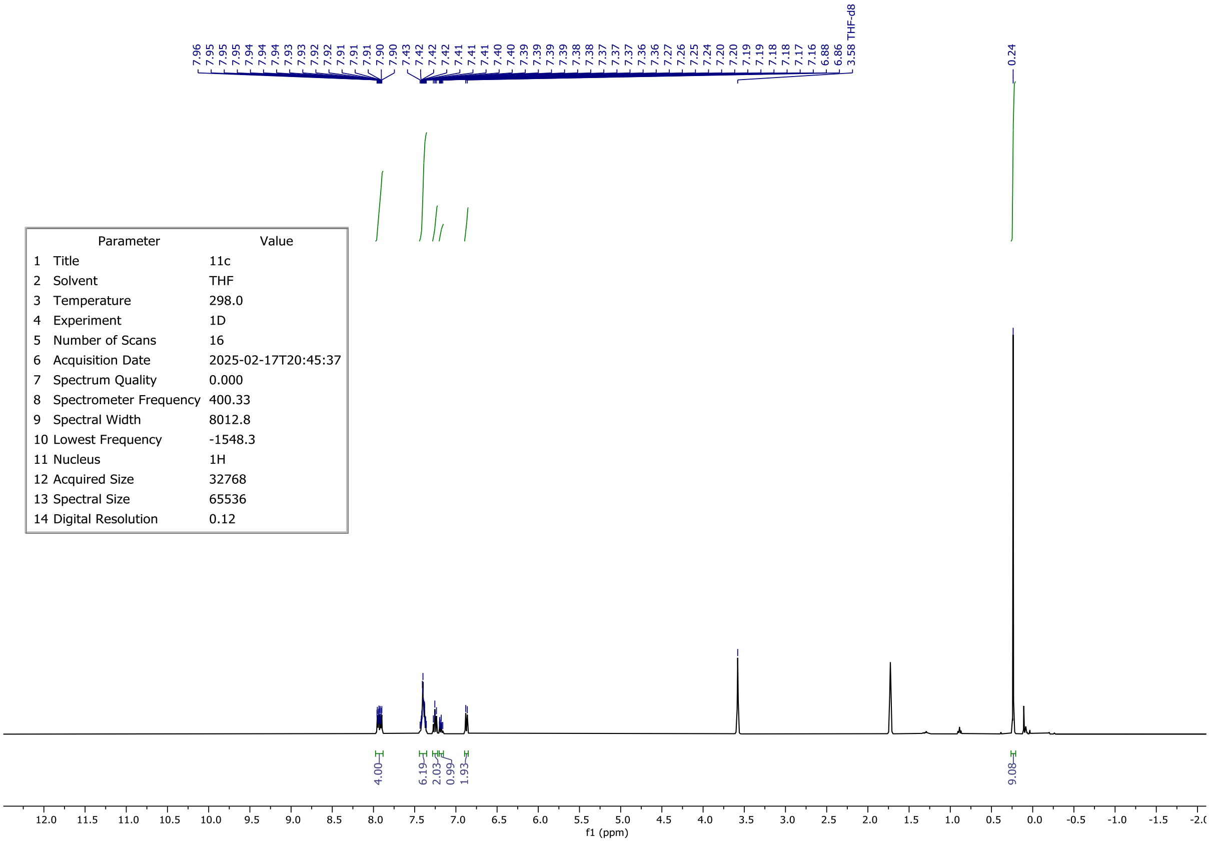


**Figure S108** $^{1}H$ NMR spectrum of compound **11c** in THF-$d_8$.

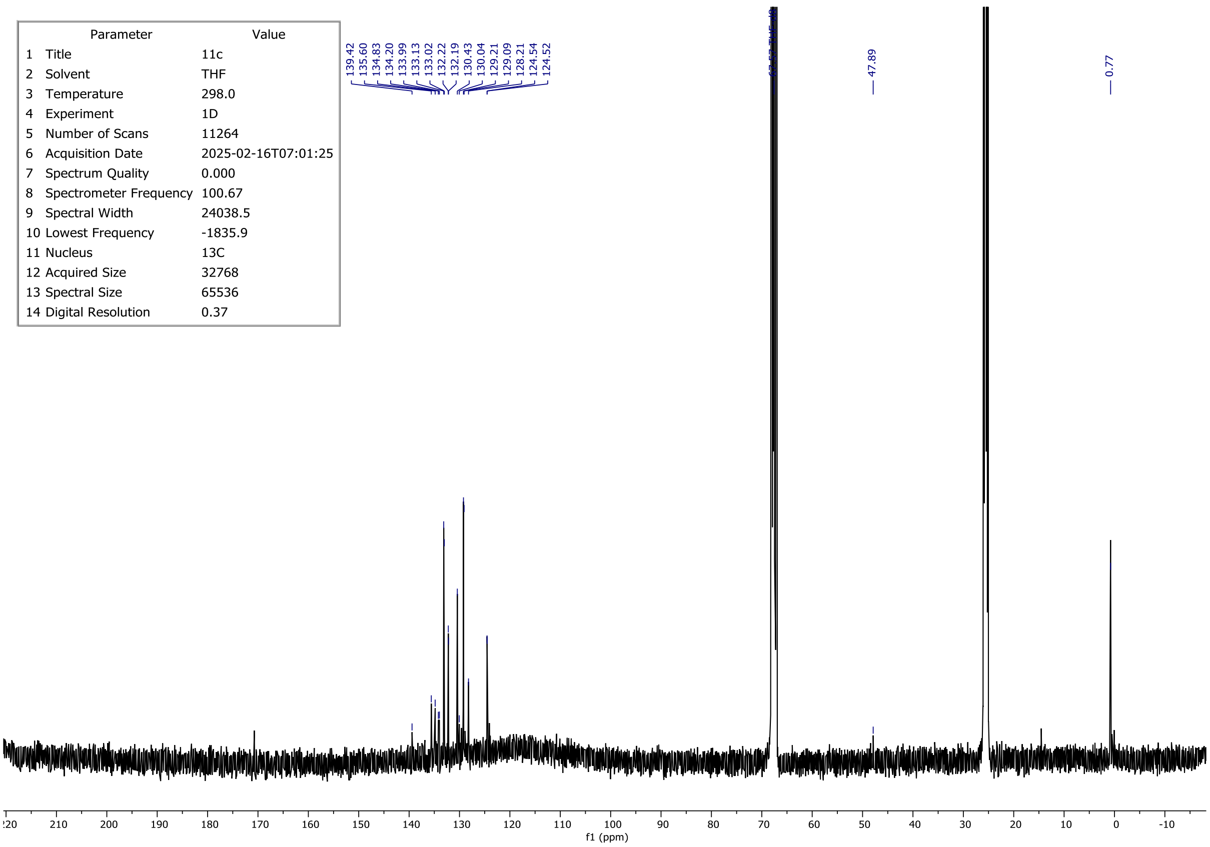


**Figure S109** $^{13}C\{^1H\}$ NMR spectrum of compound **11c** in THF-$d_8$.

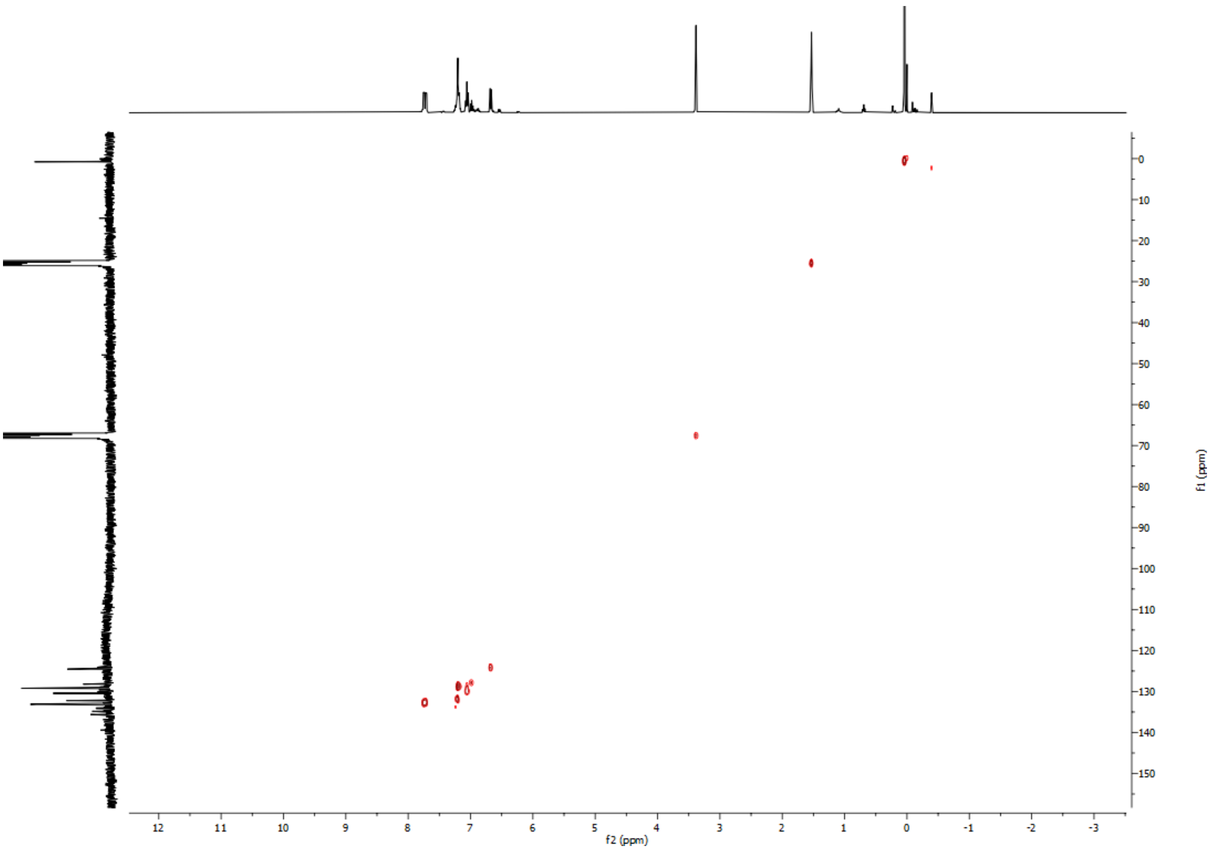


**Figure S110** $^1H$-$^{13}C$ HSQC NMR spectrum of compound **11c** in THF-$d_8$.

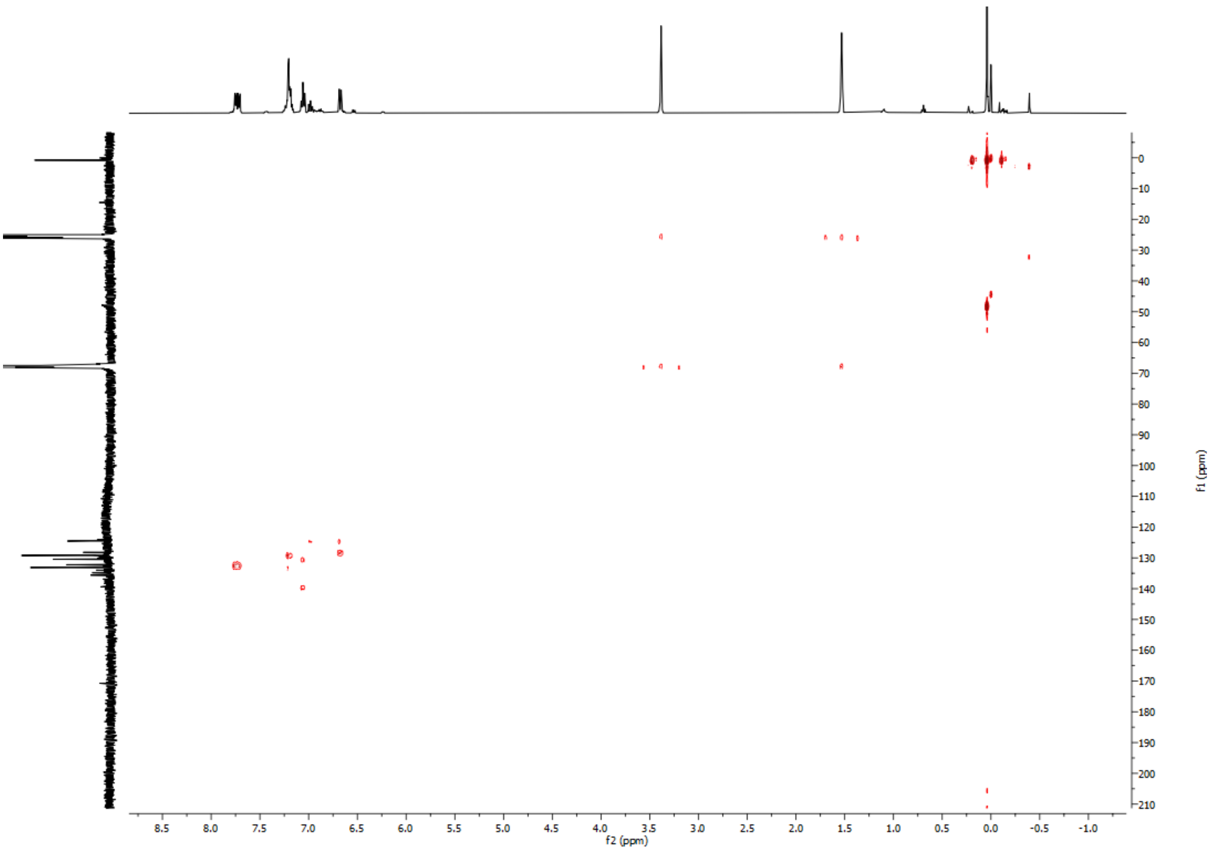


**Figure S111** $^{1}H$-$^{13}C$ HMBC NMR spectrum of compound **11c** in THF-$d_8$.

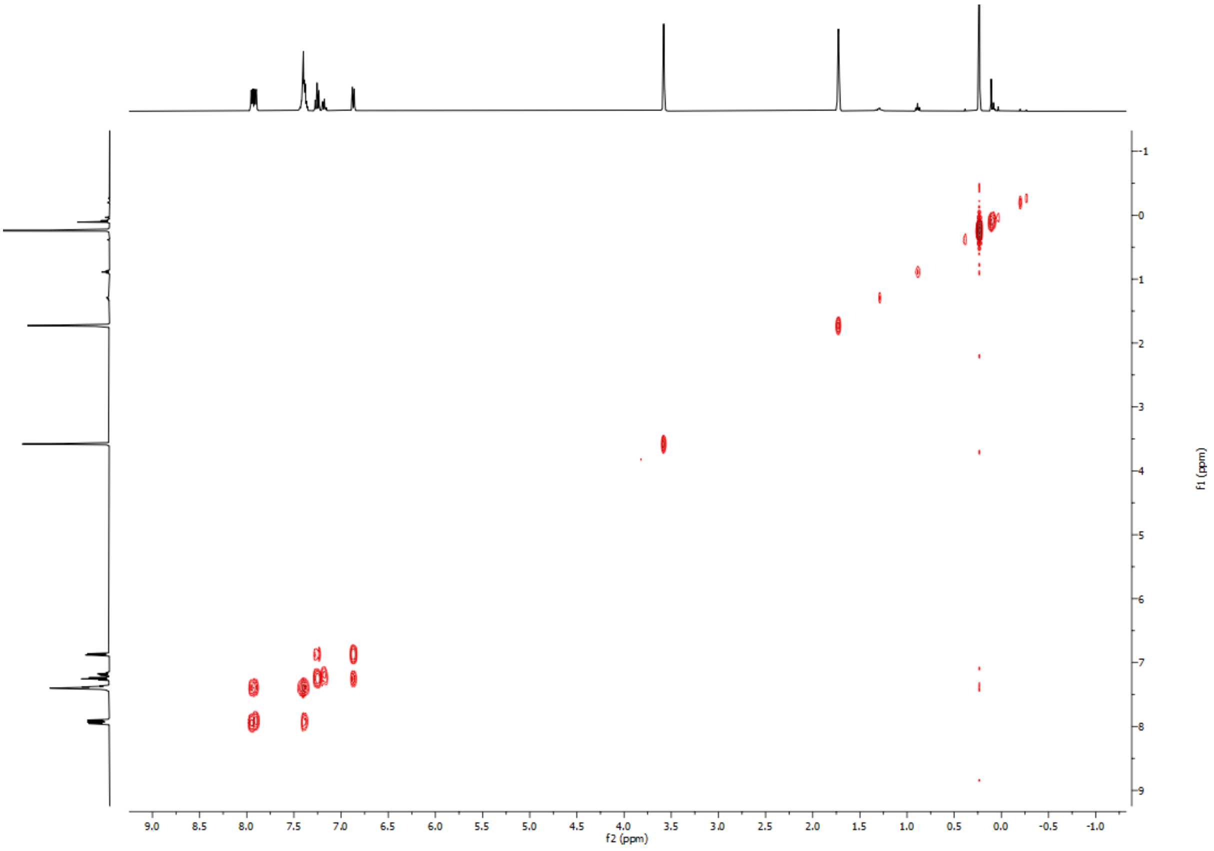


**Figure S112** $^{1}H$-$^{1}H$ COSY NMR spectrum of compound **11c** in THF-$d_8$.

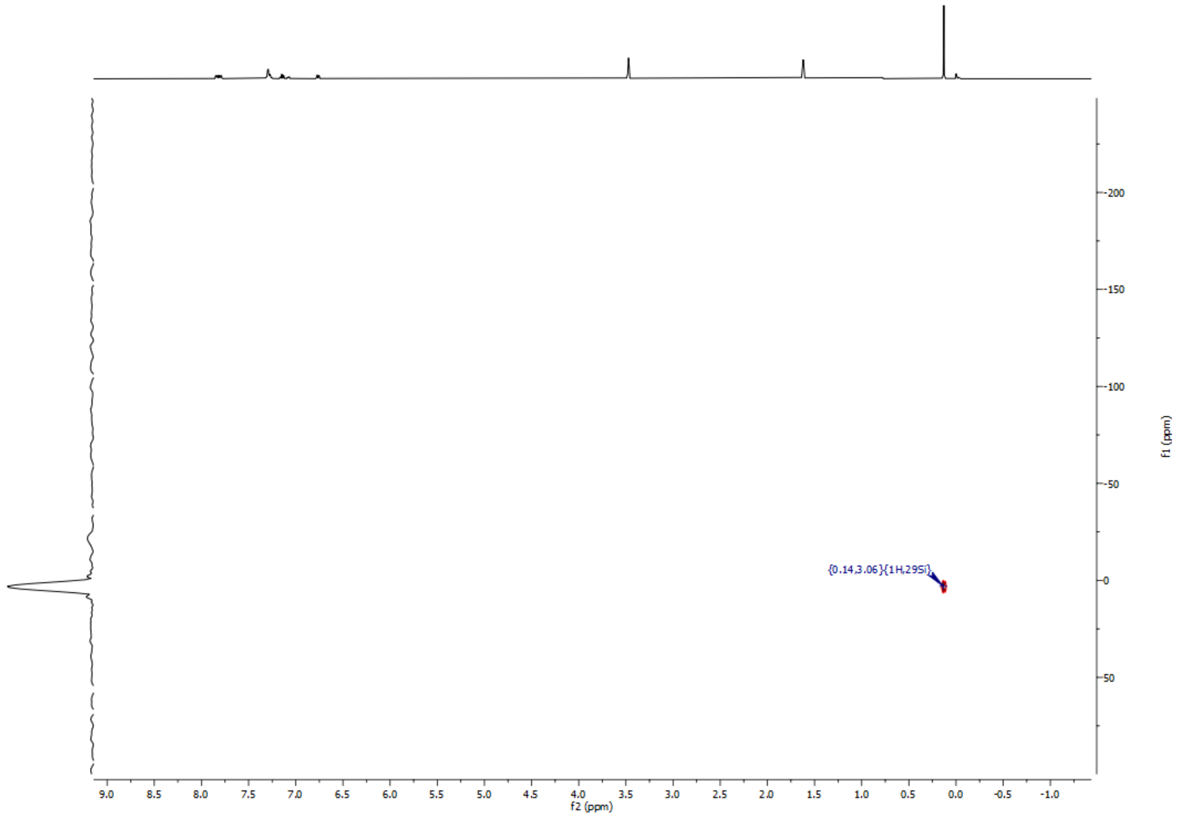

**Figure S113** $^{1}H$-$^{29}Si$ HMBC spectrum of compound **11c** in THF-$d_8$.

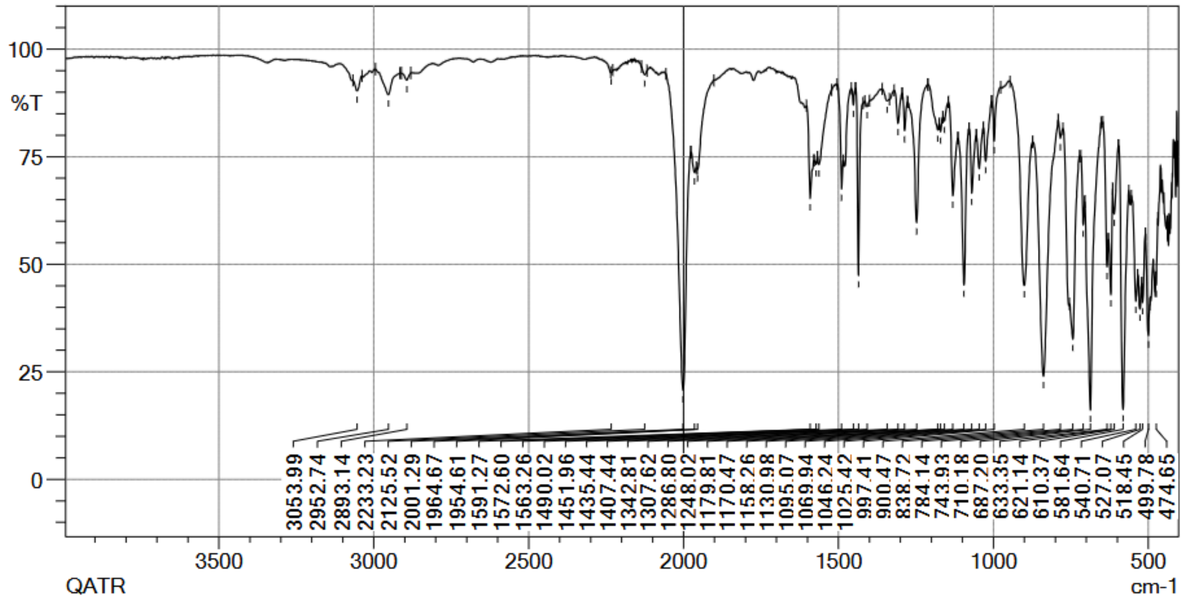

**Figure S114** IR spectrum of compound **11c** (solid state).

## 3. Crystal structure determination

### 3.1. General information

High-quality single crystals of appropriate dimensions were placed in an inert oil such as perfluoropolyalkylether, hand-picked under polarized optical microscopy and then mounted on the diffractometer. The data collection was done at 100 K. X-ray intensity data measurements of all compounds were carried out on an Oxford SuperNova diffractometer with graphite-monochromatized ($CuK_{\alpha}$= 1.54184 Å) radiation. The X-ray generator was operated at 50 kV and 30 mA.

All structures were solved by intrinsic phasing and refined by the full-matrix least-squares on $F^2$ using software package and expanded using Fourier techniques.[2,3,4,5] Non-hydrogen atoms were refined anisotropically, while all hydrogen atoms were placed on the ideal positions using riding models and refined isotropically with displacement parameters constrained to those of the parent atoms (1.5 times Ueq for methyl groups and 1.2 times Ueq for all other carbon bound H).

Data collection and structure refinement details for all compounds are given in the following tables. Further details on the structure refinement are provided in the following sections for each individual structure.

Crystallographic data including structure factors have been deposited with the Cambridge Crystallographic Data Centre as supplementary publication no. CCDC-2404561 to CCDC-404570 and CCDC-2425590 Copies of the data can be gained free of charge on application to Cambridge Crystallographic Data Centre, 12 Union Road, Cambridge CB2 1EZ, UK; [fax: (+44) 1223-336-033; email: deposit@ccdc.cam.ac.uk].

**Table S1** Data collection and structure refinement details for compounds **3a[K·(18-c-6)]**, **3b[K·(18-c-6)]** and **3c[K·(18-c-6)]**.

| **Compound** | **3a[K·(18-c-6)]** | **3b[K·(18-c-6)]** | **3c[K·(18-c-6)]** |
|---|---|---|---|
| Formula | $C_{32}H_{39}KNO_7P$ | $C_{32}H_{37}Cl_2KNO_7P$ | $C_{34}H_{43}KNO_7P$ |
| CCDC | 2404566 | 2404562 | 2404563 |
| Formula weight | 619.71 | 688.59 | 647.76 |
| Temperature [K] | 100(2) | 100(2) | 100(2) |
| Wave lenght [Å] | 1.54184 | 1.54184 | 1.54184 |
| Crystal system | Monoclinic | Monoclinic | Triclinic |
| Space group | *I*2/a | *I*2/a | *P-1* |
| a [Å] | 20.1612(7) | 20.11050(10) | 10.3759(3) |
| b [Å] | 14.0003(5) | 14.28280(10) | 12.3450(4) |
| c [Å] | 22.8665(8) | 23.3611(2) | 14.6301(5) |
| $\alpha$ [°] | 90 | 90 | 111.650(3) |
| $\beta$ [°] | 97.052(3) | 90.7060(10) | 96.913(2) |
| $\gamma$ [°] | 90 | 90 | 99.598(2) |
| Volumen [Å$^3$] | 6405.5(4) | 6709.60(8) | 1683.23(10) |
| Z | 8 | 8 | 2 |
| Calc. density [Mg·m$^{-3}$] | 1.285 | 1.363 | 1.278 |
| $\mu$ (Mo$_{K\alpha}$) [mm$^{-1}$] | 2.308 | 3.692 | 2.218 |
| F(000) | 2624 | 2880 | 688 |
| Crystal dimensions [mm] | 0.324 x 0.176 x 0.114 | 0.750 x 0.130 x 0.070 | 0.340 x 0.140 x 0.100 |
| Theta range θ [°] | 3.710 to 77.517 | 3.627 to 76.824 | 3.316 to 67.078 |
| Index ranges | -25<=h<=24 | -25<=h<=25 | -12<=h<=12 |
| | -17<=k<=16, | -17<=k<=13 | -14<=k<=14 |
| | -27<=l<=28 | -29<=l<=29 | -16<=l<=17 |
| Reflections collected | 43919 | 45922 | 19773 |
| Independent reflections | 6571 [R(int) = 0.0728] | 6911 [R(int) = 0.0310] | 6023 [R(int) = 0.0297] |
| Data/Restraints/Parameter | 6571 / 365 / 543 | 6911 / 0 / 397 | 6023 / 0 / 399 |
| Goodness-of-fit on F$^2$ | 1.107 | 1.065 | 1.046 |
| Final R indices [I>2sigma(I)] | R1 = 0.0820, wR2 = 0.1600 | R1 = 0.0427, wR2 = 0.1278 | R1 = 0.0333, wR2 = 0.0938 |
| Largest diff. peak and hole [e·Å-3] | 0.515 and -0.808 | 1.079 and -0.318 | 0.477 and -0.385 |

**Table S2** Data collection and structure refinement details for compounds **3d[K·(18-c-6)]**, **3e[K·(18-c-6)]** and **6**.

| **Compound** | **3d [K·(18-c-6)]** | **3e [K·(18-c-6)]** | **6** |
|---|---|---|---|
| Formula | $C_{32}H_{45}KNO_7P$ | $C_{30}H_{43}KNO_7P$ | $C_{39}H_{44}KN_2O_7PS$ |
| CCDC | 2404564 | 2404565 | 2404567 |
| Formula weight | 625.76 | 599.72 | 754.89 |
| Temperature [K] | 100(2) | 100(2) | 100(2) |
| Wave lenght [Å] | 1.54184 | 1.54184 | 1.54184 |
| Crystal system | Monoclinic | Monoclinic | Monoclinic |
| Space group | $P2_1$ | $P2_1$/c | $C2$/c |
| a [Å] | 9.4974(4) | 14.85260(10) | 27.0183(2) |
| b [Å] | 19.4481(5) | 10.38170(10) | 11.32120(10) |
| c [Å] | 9.5564(3) | 41.5033(3) | 24.9529(2) |
| α [°] | 90 | 90 | 90 |
| β [°] | 113.821(4) | 91.5120(10) | 99.4240(10) |
| γ [°] | 90 | 90 | 90 |
| Volumen [Å$^3$] | 1614.76(10) | 6397.38(9) | 7529.57(11) |
| Z | 2 | 8 | 8 |
| Calc. density [Mg·m$^{-3}$] | 1.287 | 1.245 | 1.332 |
| μ ($Mo_{K\alpha}$) [mm$^{-1}$] | 2.290 | 2.289 | 2.577 |
| F(000) | 668 | 2560 | 3184 |
| Crystal dimensions [mm] | 0.264 x 0.169 x 0.137 | 0.320 x 0.110 x 0.080 | 0.370 x 0.280 x 0.140 |
| Theta range θ [°] | 4.547 to 77.431 | 2.976 to 67.994 | 3.316 to 67.078 |
| Index ranges | -11<=h<=11 | -17<=h<=17 | -32<=h<=31 |
| | -23<=k<=24 | -12<=k<=11 | -13<=k<=13 |
| | -12<=l<=12 | -45<=l<=49 | -29<=l<=27 |
| Reflections collected | 9204 | 76072 | 53694 |
| Independent reflections | 9204 [R(int) = 0.0408] | 11657 [R(int) = 0.0425] | 6737 [R(int) = 0.0287] |
| Data/Restraints/Parameter | 9204 / 1 / 380 | 11657 / 349 / 890 | 6737 / 0 / 460 |
| Goodness-of-fit on F$^2$ | 1.081 | 1.020 | 1.042 |
| Final R indices [I>2sigma(I)] | R1 = 0.0327, wR2 = 0.0975 | R1 = 0.0318, wR2 = 0.0839 | R1 = 0.0472, wR2 = 0.1311 |
| Largest diff. peak and hole [e·Å-3] | 0.312 and -0.352 | 0.594 and -0.367 | 1.220 and -0.591 |

**Table S3** Data collection and structure refinement details for compounds **7b** and **8**.

| **Compound** | **7b** | **8** |
|---|---|---|
| Formula | $C_{39}H_{44}Cl_2KN_4O_7P$ | $C_{47}H_{51}KNO_8P$ |
| CCDC | 2404570 | 2404568 |
| Formula weight | 821.75 | 827.95 |
| Temperature [K] | 100(2) | 100(2) |
| Wave lenght [Å] | 1.54184 | 1.54184 |
| Crystal system | Monoclinic | Triclinic |
| Space group | $P2_1/c$ | P-1 |
| a [Å] | 16.0932(2) | 12.30160(10) |
| b [Å] | 13.5649(2) | 19.6958(2) |
| c [Å] | 18.7888(2) | 20.1885(2) |
| $\alpha$ [°] | 90 | 117.6900(10) |
| $\beta$ [°] | 101.2010(10) | 91.9050(10) |
| $\gamma$ [°] | 90 | 97.3350(10) |
| Volumen [$Å^3$] | 4023.51(9) | 4271.90(8) |
| Z | 4 | 4 |
| Calc. density [$Mg \cdot m^{-3}$] | 1.357 | 1.287 |
| $\mu$ ($Mo_{K\alpha}$) [$mm^{-1}$] | 3.193 | 1.886 |
| F(000) | 1720 | 1752 |
| Crystal dimensions [mm] | 0.390 x 0.240 x 0.070 | 0.360 x 0.230 x 0.160 |
| Theta range θ [°] | 2.799 to 77.038 | 2.567 to 67.997 |
| Index ranges | -19<=h<=20 | -14<=h<=14 |
| | -16<=k<=17 | -23<=k<=23 |
| | -23<=l<=17 | -24<=l<=24 |
| Reflections collected | 54513 | 58305 |
| Independent reflections | 8297 [R(int) = 0.0389] | 15554 [R(int) = 0.0293] |
| Data/Restraints/Parameter | 8297 / 335 / 651 | 15554 / 244 / 1085 |
| Goodness-of-fit on $F^2$ | 1.044 | 1.051 |
| Final R indices [I>2sigma(I)] | R1 = 0.0324, wR2 = 0.0847 | R1 = 0.0292, wR2 = 0.0741 |
| Largest diff. peak and hole [e·Å-3] | 0.390 and -0.394 | 0.490 and -0.309 |

**Table S4** Data collection and structure refinement details for compounds **9** ,**10** and **3g [Li·(12-c-4)]**

| **Compound** | **9** | **10** | **3g [Li·(12-c-4)]** |
|---|---|---|---|
| Formula | $C_{47}H_{51}CuN_3OP$ | $C_{74}H_{84}K_2N_2O_{22}P_2$ | $C_{28}H_{31}LiNO_4PSe$ |
| CCDC | 2404569 | 2404561 | 2425590 |
| Formula weight | 768.41 | 1493.57 | 562.41 |
| Temperature [K] | 100(2) | 100(2) | 100(2) |
| Wave lenght [Å] | 1.54184 | 1.54184 | 1.54184 |
| Crystal system | Orthorhombic | Triclinic | Monoclinic |
| Space group | $Pna2_1$ | *P*-1 | $P2_1$ |
| a [Å] | 18.99530(10) | 11.9814(2) | 11.6772(4) |
| b [Å] | 20.33750(10) | 16.2623(2) | 9.2932(3) |
| c [Å] | 21.51410(10) | 19.9279(3) | 12.4533(4) |
| α [°] | 90 | 70.1830(10) | 90 |
| β [°] | 90 | 89.2040(10) | 91.320(3) |
| γ [°] | 90 | 82.5170(10) | 90 |
| Volumen [$Å^3$] | 8311.26(7) | 3619.90(10) | 1351.06(8) |
| Z | 8 | 2 | 2 |
| Calc. density [$Mg·m^{-3}$] | 1.228 | 1.370 | 1.382 |
| μ ($Mo_{K\alpha}$) [$mm^{-1}$] | 1.389 | 2.227 | 2.711 |
| F(000) | 3248 | 1572 | 580 |
| Crystal dimensions [mm] | 0.250 x 0.160 x 0.080 | 0.196 x 0.131 x 0.071 | 0.610 x 0.080 x 0.040 |
| Theta range θ [°] | 2.990 to 67.981 | 2.358 to 67.080 | 3.550 to 77.126 |
| Index ranges | -22<=h<=22 | -14<=h<=14 | -14<=h<=14 |
| | -24<=k<=24 | -19<=k<=19 | -9<=k<=11 |
| | -25<=l<=25 | -23<=l<=23 | -15<=l<=15 |
| Reflections collected | 273764 | 47673 | 15756 |
| Independent reflections | 15148 [R(int) = 0.0511] | 12935 [R(int) = 0.0306] | 4073 [R(int) = 0.0650] |
| Data/Restraints/Parameter | 15148 / 1 / 992 | 12935 / 253 / 1158 | 4073 / 213 / 434 |
| Goodness-of-fit on $F^2$ | 1.019 | 1.034 | 1.033 |
| Final R indices [I>2sigma(I)] | R1 = 0.0341, wR2 = 0.0934 | R1 = 0.0360, wR2 = 0.0927 | R1 = 0.0500, wR2 = 0.1292 |
| Largest diff. peak and hole [e·Å-3] | 0.711 and -0.287 | 0.814 and -0.760 | 0.618 and -1.023 |

### 3.2. Molecular structure of 3a[K·(18-c-6)]

All hydrogen atoms were placed on ideal positions. The crystal structure contained a disordered 18-crown-6 ligand. The disorder was modelled using SAME, SIMU and DELU restraints and refined using the PART instructions and free variables, which optimized to occupancies of 78% and 22%.

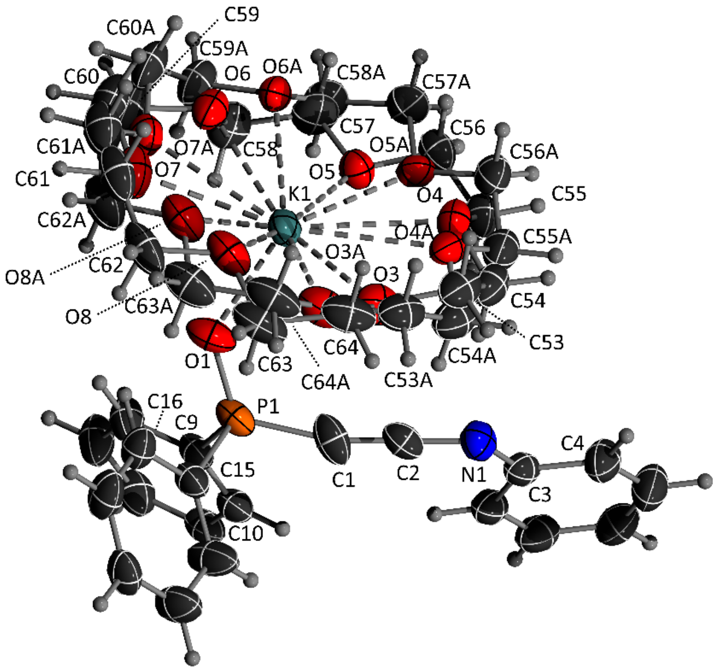


**Figure S115** Molecular structure of compound **3a[K·(18-c-6)]**. Thermal ellipsoids at 50% probability level. Selected bond lengths [Å] and angles [°]: P1-C1 1.694(5), C1-C2 1.221(6), P1-O1 1.490(3), C2-N1 1.288(5), P1-C1-C2 167.3(4).

### 3.3. Molecular structure of 3b[K·(18-c-6)]

All hydrogen atoms were placed on ideal positions.

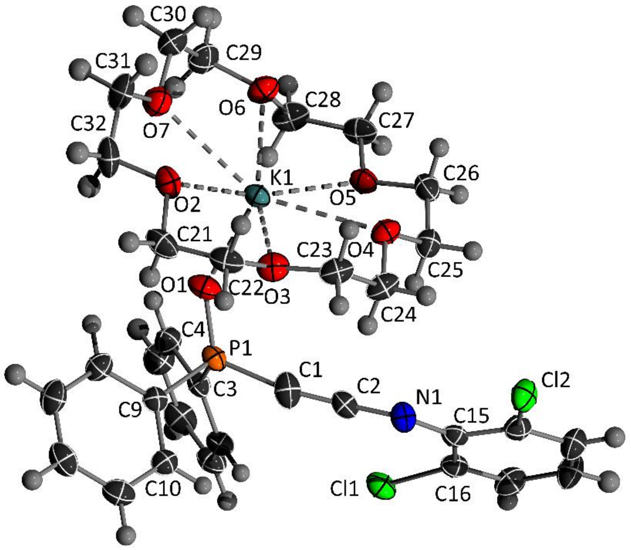


**Figure S116** Molecular structure of compound **3b[K·(18-c-6)]**. Thermal ellipsoids at 50% probability level. Selected bond lengths [Å] and angles [°]: P1-C1 1.701(2), C1-C2 1.228(3), P1-O1 1.490(2), C2-N1 1.281(3), P1-C1-C2 166.8(2).

### 3.4. Molecular structure of 3c[K·(18-c-6)]

All hydrogen atoms were placed on ideal positions.

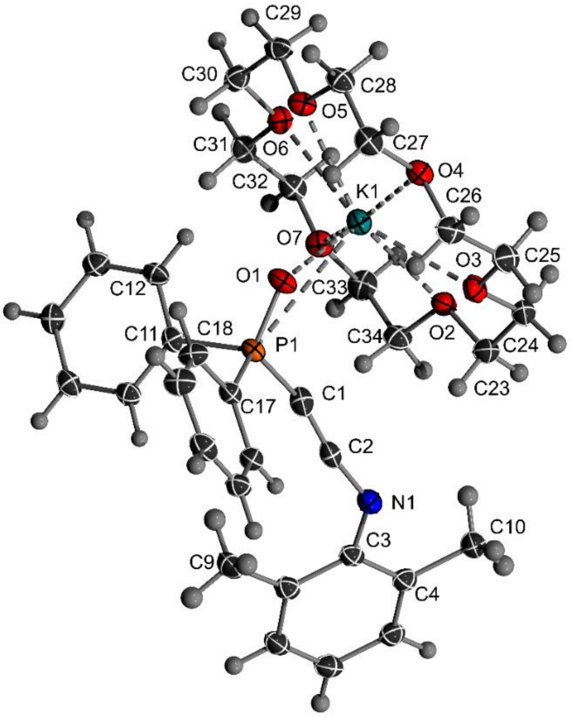


**Figure S117** Molecular structure of compound **3c[K·(18-c-6)]**. Thermal ellipsoids at 50% probability level. Thermal ellipsoids at 50% probability level. Selected bond lengths [Å] and angles [°]: P1-C1 1.693(2), C1-C2 1.239(2), P1-O1 1.498(1), C2-N1 1.271(2), P1-C1-C2 159.5(1).

### 3.5. Molecular structure of 3d[K·(18-c-6)]

All hydrogen atoms were placed on ideal positions. The structure has been solved as a two component twin using the MERG and BASF instructions refining to a ratio of 42% and 58% (twin law: -0.0087 -0.0005 -0.9951 0.0046 -1.0001 0.0047 -1.0038 -0.0001 0.0095).

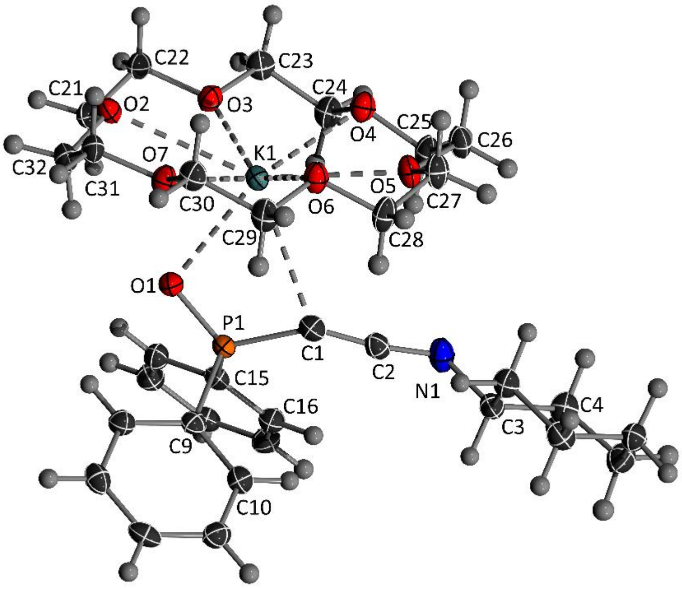


**Figure S118** Molecular structure of compound **3d[K·(18-c-6)]**. Thermal ellipsoids at 50% probability level. Thermal ellipsoids at 50% probability level. Thermal ellipsoids at 50% probability level. Selected bond lengths [Å] and angles [°]: P1-C1 1.696(5), C1-C2 1.283(6), P1-O1 1.497(3), C2-N1 1.246(6), P1-C1-C2 139.3(4).

### 3.6. Molecular structure of 3e[K·(18-c-6)]

All hydrogen atoms were placed on ideal positions. The crystal structure contained a disordered 18-crown-6 ligand. The disorder was modelled using SAME, SIMU and DELU restraints and refined using the PART instructions and free variables, which optimized to occupancies of 67% and 33%.

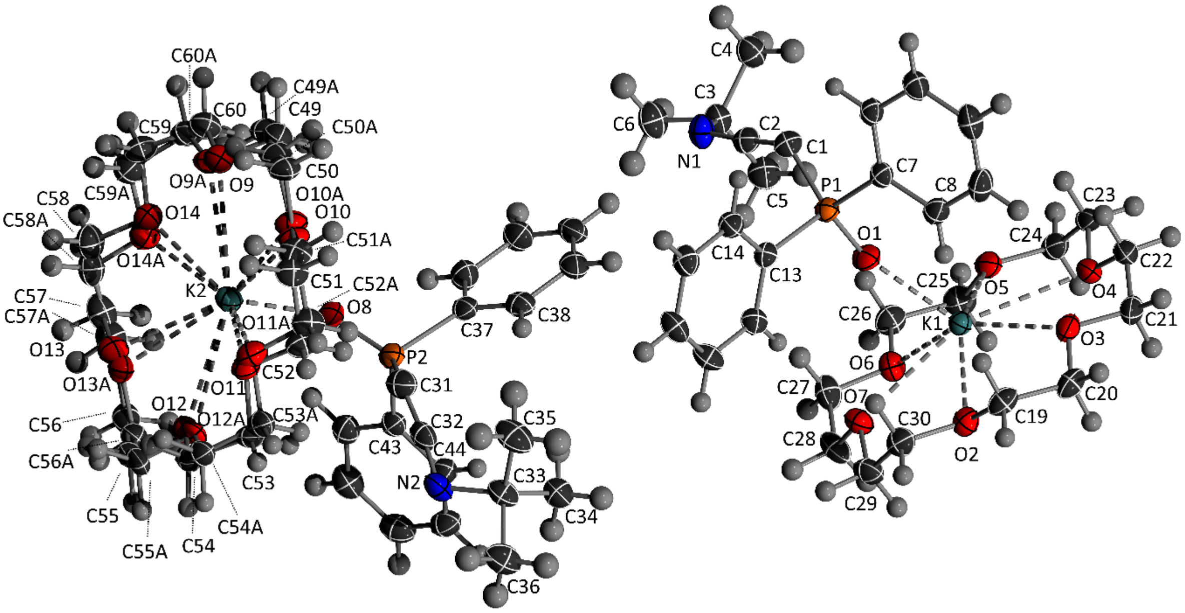


**Figure S119** Molecular structure of compound **3e[K·(18-c-6)]**. Thermal ellipsoids at 50% probability level. Thermal ellipsoids at 50% probability level. Selected bond lengths [Å] and angles [°]: P1-C1 1.699(2), P2-C31 1.700(2), C1-C2 1.271(2), C31-C32 1.266(2), P1-O1 1.498(1), P2-O8 1.499(1), C2-N1 1.253(2), C32-N2 1.259(2), P1-C1-C2 132.2(1), P2-C31-C32 137.2(1).

### 3.7. Molecular structure of 3g[Li·(12-c-4)]

All hydrogen atoms were placed on ideal positions. The crystal structure contained a disordered 12-crown-4 ligand. The disorder was modelled using the PART instructions and free variables, which optimized to occupancies of 55% and 45%.

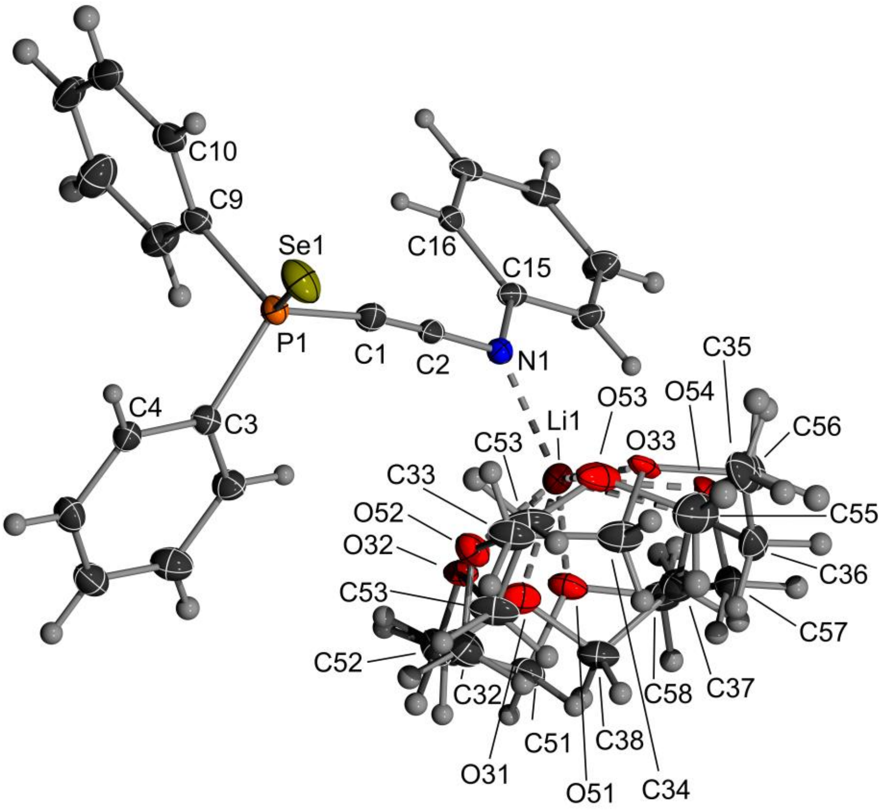


**Figure S120** Molecular structure of compound **3g[Li·(12-c-4)]**. Thermal ellipsoids at 50% probability level. Selected bond lengths [Å] and angles [°]: P1-C1 1.710(5), C1-C2 1.212(8), C2-N1 1.299(7), P1-Se1 2.117(1), P1-C1-C2 170.8(5), C1-C2-N1 175.4(5), C2-N1-C15 119.7(4).

## 3.8. Molecular structure of 6

All hydrogen atoms were placed on ideal positions.

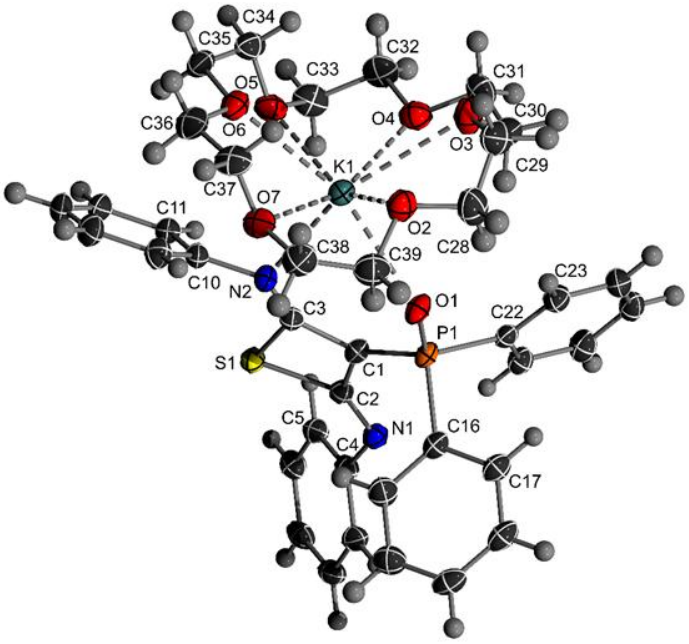


**Figure S121** Molecular structure of compound **6**. Thermal ellipsoids at 50% probability level. Selected bond lengths [Å] and angles [°]: C1-C2 1.427(3), C1-C3 1.431(3), C2-N1 1.277(3), C3-N2 1.277(3), C2-S1 1.844(2), C3-S1 1.853(2), P1-C1-C2 131.2(2), P1-C1-C3 128.3(2).

## 3.9. Molecular structure of 7b

All hydrogen atoms were placed on ideal positions. The crystal structure contained a disordered 18-crown-6 ligand. The disorder was modelled using SAME, SIMU and DELU restraints and refined using the PART instructions and free variables, which optimized to occupancies of 76% and 24%.

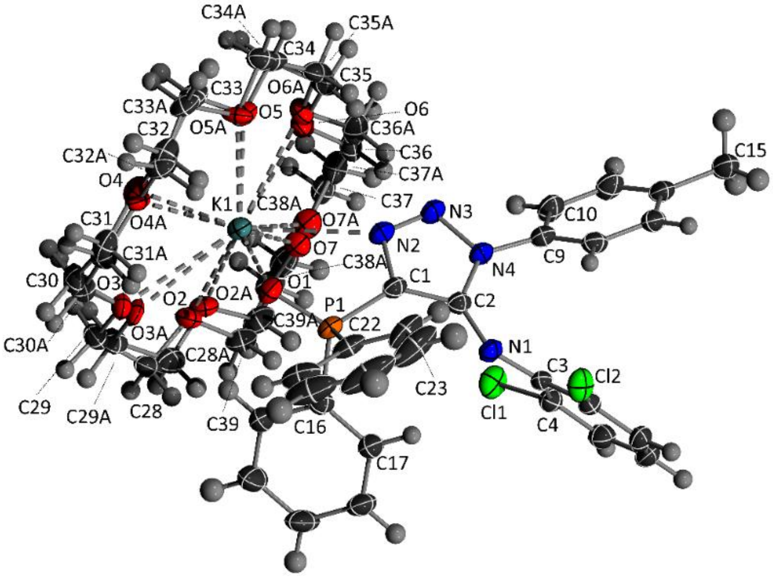


**Figure S122** Molecular structure of compound **7b**. Thermal ellipsoids at 50% probability level. Selected bond lengths [Å] and angles [°]: P1-C1 1.775(2), C1-C2 1.414(2), C2-N1 1.328(2), C2-N4 1.384(2), C1-N2 1.369(2), N3-N4 1.393(2), P1-C1-C2 132.8(1).

### 3.10. Molecular structure of 8

All hydrogen atoms were placed on ideal positions. The crystal structure contained a disorder in one of the backbone bound phenyl rings. The disorder was modelled using SADI, SIMU, DELU and AFIX66 restraints and refined using the PART instructions and free variables, which optimized to occupancies of 62% and 38%.

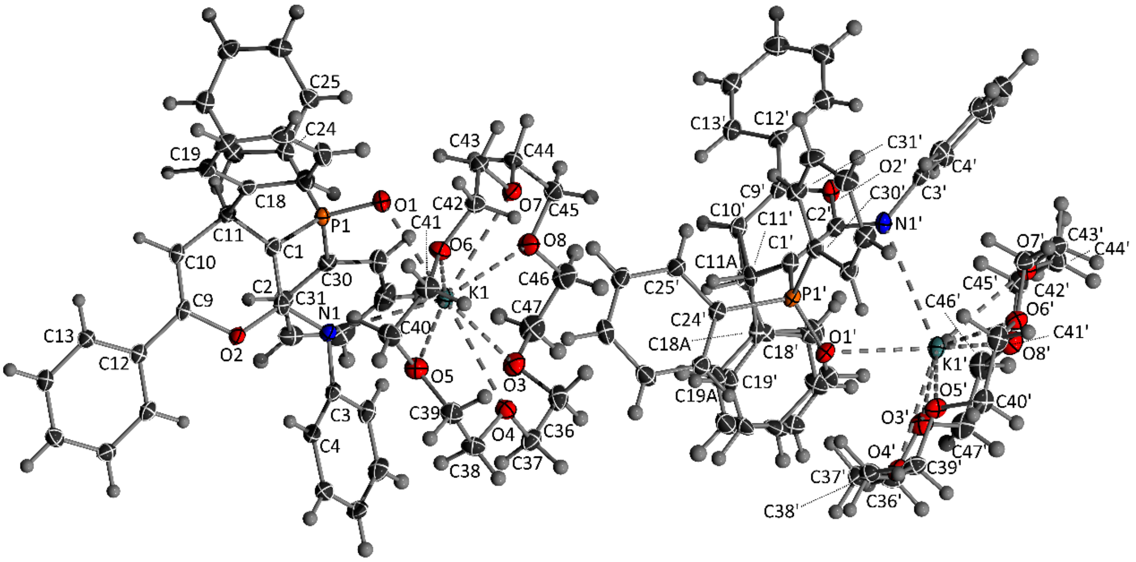


**Figure S123** Molecular structure of compound **8**. Thermal ellipsoids at 50% probability level. Selected bond lengths [Å] and angles [°]: P1-C1 1.744(1), C1-C2 1.394(2), C2-N1 1.305(2), C1-C11 1.515(2), P1'-C1' 1.741(1), C1'-C2' 1.390(2), C2'-N1' 1.310(2), C1'-C11' 1.574(9), P1'-C1'-C2' 117.8(9), P1-C1-C2 115.1(9).

### 3.11. Molecular structure of 9

All hydrogen atoms were placed on ideal positions. The crystal structure contained a disorder in one of the isopropyl moieties which was refined using the PART instructions and free variables leading to occupancies of 56% and 44%.

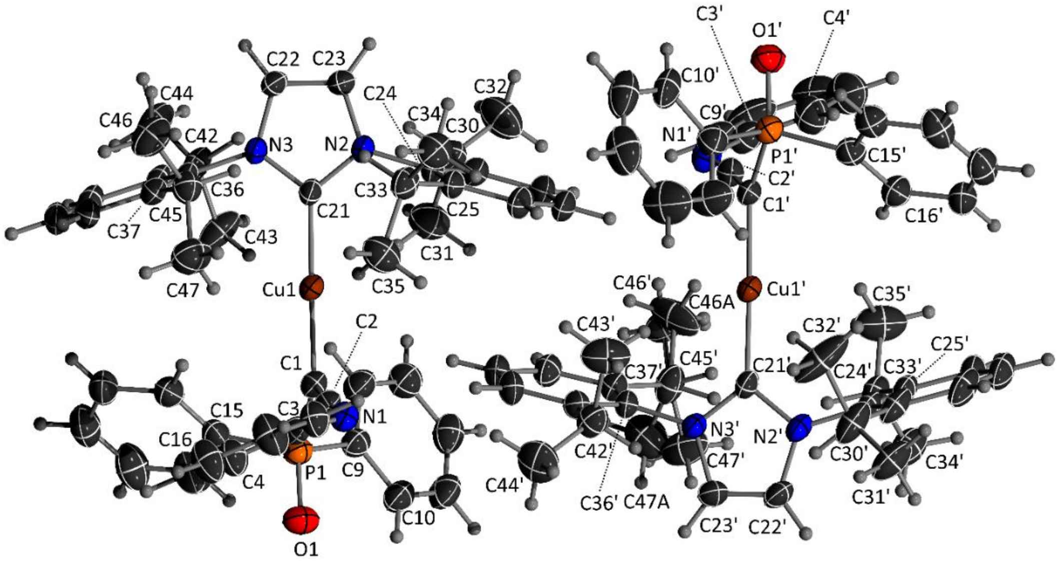


**Figure S124** Molecular structure of compound **9**. Thermal ellipsoids at 50% probability level. Selected bond lengths [Å] and angles [°]: P1-C1 1.762(3), C1-C2 1.282(5), C2-N1 1.251(4), P1'-C1' 1.771(3), C1'-C2' 1.274(5), C2'-N1' 1.242(5), P1'-C1'-C2' 116.9(3), P1-C1-C2 114.9(2).

## 3.12. Molecular structure of 10

All hydrogen atoms were placed on ideal positions. The crystal structure contained a disordered 18-crown-6 ligand. The disorder was modelled using SIMU and DELU restraints and refined using the PART instructions and free variables, which optimized to occupancies of 73% and 27%. A second threefold disorder of a phenyl ring was modelled using the AFIX66 restraint and refined using the PART and SUMP instructions and free variables, which optimized to occupancies of 33%, 43% and 24%. A third minor disorder in one 18-crown-6 ligand was refined using the PART instructions and free variables resulting in occupancies of 77% and 23%. An additional benzene molecule has been modelled with the AFIX66 restraint.

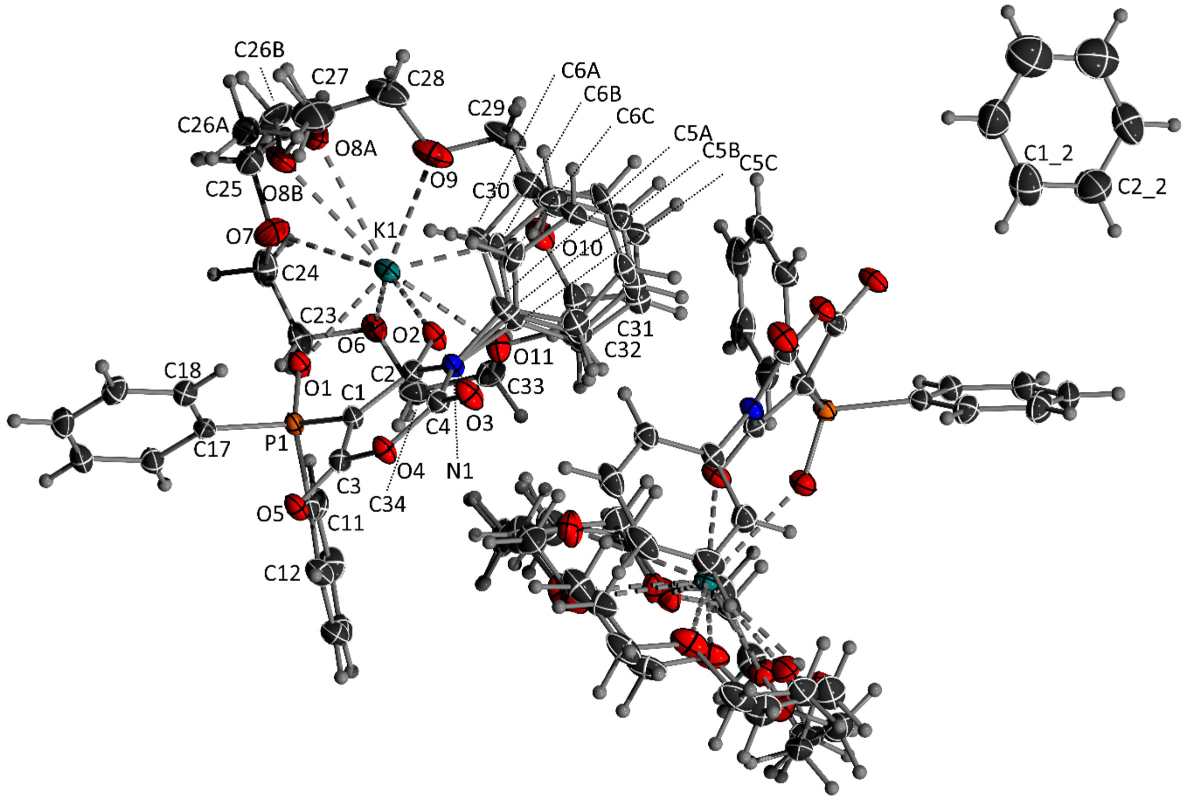


**Figure S125** Molecular structure of compound **10**. Thermal ellipsoids at 50% probability level. Selected bond lengths [Å] and angles [°]: P1-C1 1.789(2), C1-C2 1.429(2), C1-C3 1.407(2), C4-N1 1.370(2), C2-N1 1.437(2), P1-C1-C2 120.3(1)

## 4. Computational details

### 4.1. General remarks

Geometry optimization of **3b**, **3d**, **4** and **5** in the electronic singlet ground state were calculated at the BP86[6,7] level corrected by Grimme's D3 dispersion with Becke and Johnson damping (denoted as D3(BJ) hereafter)[8,9,10] together with the triple zeta basis set def2-TZVPP[11] unless otherwise specified. The linear structures were determined through constrained optimizations at the same computational level, with the P–C–C or P–C–N angles fixed at 180 degrees. Single point calculations for acquiring electron energy and frequencies analysis, validating the stability of optimized geometries, and obtaining thermal correction values are carried out at the same level. The above mentioned calculations were carried out with the program Gaussian 16.[12] Furthermore, Voronoi Deformation Density(VDD) Charges[13] and Mayer Bond Order(MBO)[14,15] is calculated by Multiwfn.[16]

The bonding situation was analysed by means of an energy decomposition analysis (denoted as EDA hereafter)[17,18] together with the natural orbitals for chemical valence (denoted as NOCV hereafter)[19,20] method by using the ADF 2022 program package.[21,22] The EDA-NOCV calculations were carried out at the BP86/TZ2P[23] level using the optimized geometries at the BP86+D3(BJ)/def2-TZVPP level. In this analysis, the intrinsic interaction energy ($\Delta E_{int}$) between considered fragments can be divided into four energy components as follows:

$$\Delta E_{int} = \Delta E_{elstat} + \Delta E_{pauli} + \Delta E_{orb} + \Delta E_{disp} \qquad (1)$$

While the electrostatic $\Delta E_{elstat}$ term represents the quasiclassical electrostatic interaction between the unperturbed charge distributions of the prepared fragments, the Pauli repulsion $\Delta E_{Pauli}$ corresponds to the energy change associated with the transformation from the superposition of the unperturbed electron densities of the isolated fragments to the wavefunction,[24] which properly obeys the Pauli principle through explicit antisymmetrization and renormalization of the production wavefunction. The orbital term $\Delta E_{orb}$ can be further decomposed into contributions from each irreducible representation of the point group of the interacting system as follows:

$$\Delta E_{orb} = \sum_r \Delta E_r \qquad (2)$$

The combination of the EDA with NOCV enables the partition of the total orbital interactions into pairwise contributions of the orbital interactions which is very vital to get a complete picture of the bonding. The charge deformation $\Delta\rho_k(r)$, resulting from the mixing of the orbital pairs $\psi_K(r)$ and $\psi_{-K}(r)$of the interacting fragments presents the amount and the shape of the charge flow due to the orbital interactions [Eq. (3)], and the associated energy term $\Delta E_{orb}$ provides with the size of stabilizing orbital energy originated from such interaction [Eq. (4)].

$$\Delta\rho_{orb}(r) = \sum_{k} \Delta\rho_k(r) = \sum_{K=1}^{N/2} V_K \left[-\psi_{-K}^2(r) + \psi_k^2(r)\right] \qquad (3)$$

$$\Delta E_{orb} = \sum_{k} \Delta E_k^{orb} = \sum_{K=1}^{N/2} V_K\left[-F_{-K,-K}^{TS} + F_{k,K}^{TS}\right] \qquad (4)$$

More details about the EDA-NOCV method and its application are given in recent reviews articles.[25,26,27,28]

## 4.2. Bonding analysis

### 4.2.1 Comparison of 3b and 3d with their protonated congeners

**Table S5** Calculated proton affinities PAs (in eV) and frontier orbital with associated energy level ε (in eV) of two isomers(ketenimine and yne-amine) of protonated **3b** (**3b-H$_C$** and **3b-H$_N$**) and protonated **3d** (**3d-H$_C$** and **3d-H$_N$**) at BP86+D3(BJ)/Def2-TZVPP level

| Isomer form | ketenimine 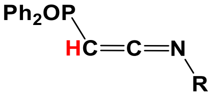  | | yne-amine 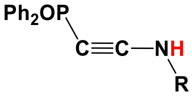  | |
|---|---|---|---|---|
| Species | **3b-H$_C$ (R=Ar)** | **3d-H$_C$ (R=Cy)** | **3b-H$_N$ (R=Ar)** | **3d-H$_N$ (R=Cy)** |
| PAs | 14.6 | 15.2 | 13.7 | 14.3 |
| LUMO | 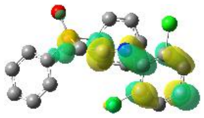 (ε= -2.82 eV) | 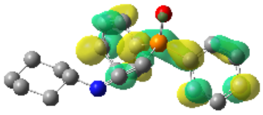 (ε= -1.99 eV) | 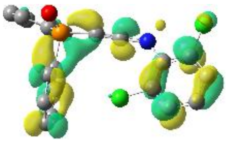 (ε= -2.08 eV) | 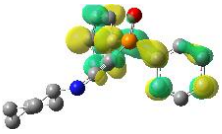 (ε= -1.91 eV) |
| HOMO | 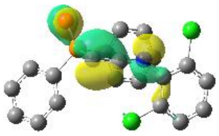 (ε= -5.88 eV) | 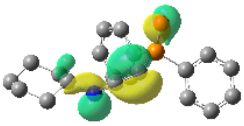 (ε= -5.79 eV) | 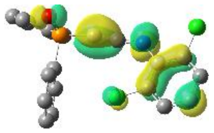 (ε= -5.57 eV) | 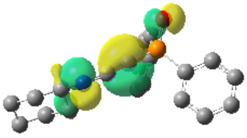 (ε= -5.46 eV) |
| HOMO-LUMO GAP | 3.06 eV | 3.80 eV | 3.49 eV | 3.55 eV |

**Table S6.** Calculated frontier orbital with associated energy level ε (in eV)of **3b**, **3d**, **4** and **5** at BP86+D3(BJ)/Def2-TZVPP level

| Species | **POPh$_2$CCNAr** | **POPh$_2$CCNCy** | **POPh$_2$CCO** | **POPh$_2$CNN** |
|---|---|---|---|---|
| LUMO | 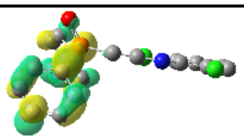 (ε= 0.84 eV) | 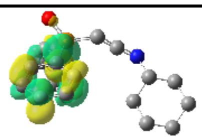 (ε= 1.24 eV) | 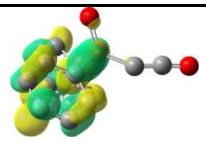 (ε= 1.21 eV) | 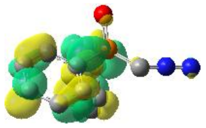 (ε= 1.24 eV) |
| HOMO | 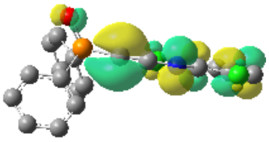 (ε= -1.35 eV) | 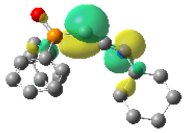 (ε= -0.88 eV) | 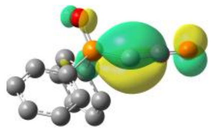 (ε= -1.13 eV) | 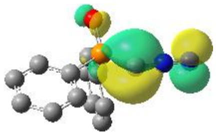 (ε= -0.68 eV) |
| HOMO-LUMO GAP | 2.19 eV | 2.12 eV | 2.34 eV | 1.93 eV |

**Table S7.** The calculated Voronoi deformation density charge(q), Mayer bond order (MBO) and Wiberg bond indices (WBI) of the optimized structures of [Ph2OPCCNAr]- (**3b**), [Ph2OPCCNCy]- (**3d**), [Ph2OPHC=C=NAr] (**3b-H$_C$**), [Ph2OPC≡C-NHAr] (**3b-H$_N$**), [Ph2OPHC=C=NCy] (**3d-H$_C$**) and [Ph2OPC≡C-NHCy] (**3d-H$_N$**) at BP86/def2-TZVPP level.

| Species | q | | WBI | | MBO | |
|---|---|---|---|---|---|---|
| | P1 | 0.38 | P1-C1 | 1.00 | P1-C1 | 1.24 |
| | C1 | -0.31 | C1-C2 | 2.26 | C1-C2 | 2.20 |
| **3b'**[Ph$_2$OPCNCAr]$^-$ | C2 | 0.04 | C2-N1 | 1.52 | C2-N1 | 1.72 |
| | N1 | -0.20 | N1-Ar | 1.26 | N1-Ar | 1.30 |
| | PCCN | -0.08 | | | | |
| | P1 | 0.38 | P1-C1 | 1.03 | P1-C1 | 1.21 |
| | C1 | -0.38 | C1-C2 | 2.14 | C1-C2 | 1.85 |
| **3d'**[Ph$_2$OPCNCCy]$^-$ | C2 | 0.03 | C2-N1 | 1.68 | C2-N1 | 1.97 |
| | N1 | -0.27 | N1-Ar | 1.00 | N1-Ar | 0.99 |
| | PCCN | -0.24 | | | | |
| | P1 | 0.40 | P1-C1 | 0.79 | P1-C1 | 0.92 |
| | C1 | -0.19 | C1-C2 | 1.77 | C1-C2 | 1.71 |
| **3b-H$_C$** [Ph$_2$OPHC=C=NAr] | C2 | 0.13 | C2-N1 | 1.92 | C2-N1 | 2.02 |
| | N1 | -0.10 | N1-Ar | 1.09 | N1-Ar | 1.07 |
| | PCCN | 0.24 | | | | |
| | P1 | 0.40 | P1-C1 | 0.87 | P1-C1 | 1.03 |
| **3b-H$_N$** [Ph$_2$OPC≡C-NHAr] | C1 | -0.17 | C1-C2 | 2.60 | C1-C2 | 2.52 |
| | C2 | 0.01 | C2-N1 | 1.18 | C2-N1 | 1.25 |

| | | | | | | |
|---|---|---|---|---|---|---|
| | N1 | -0.07 | N1-Ar | 1.08 | N1-Ar | 1.10 |
| | PCCN | 0.17 | | | | |
| | P1 | 0.38 | P1-C1 | 0.82 | P1-C1 | 0.96 |
| | C1 | -0.19 | C1-C2 | 1.75 | C1-C2 | 1.64 |
| **3d-H$_C$** [$Ph_2OPHC=C=NCy$] | C2 | 0.10 | C2-N1 | 2.05 | C2-N1 | 2.21 |
| | N1 | -0.13 | N1-Ar | 0.95 | N1-Ar | 0.83 |
| | PCCN | 0.16 | | | | |
| | P1 | 0.40 | P1-C1 | 0.89 | P1-C1 | 1.07 |
| **3d-H$_N$** | C1 | -0.19 | C1-C2 | 2.58 | C1-C2 | 2.47 |
| | C2 | 0.00 | C2-N1 | 1.24 | C2-N1 | 1.31 |
| [$Ph_2OPC\equiv C\text{-}NHCy$] | N1 | -0.11 | N1-Ar | 0.94 | N1-Ar | 0.92 |
| | PCCN | 0.10 | | | | |

### 4.2.2 Comparison of 3b and 3d with 4 and 5

We carried out DFT calculations at the BP86+D3(BJ)/def2-TZVPP level to analyze the electronic structure and bonding situation in the anionic complexes. The calculated geometries of the naked anions $[Ph_2OPC\text{-}L2]^-$ with L2 = CNAr (**3b**), L2 = CNCy (**3d**), L2 = CO (**4**) and L2 = $N_2$ (**5**) are shown in Figure S55. The most important structural values are shown in Table 2 along with the experimental results. The agreement between the calculated and the experimental data is very good. Please note that the significant reduction in the bending angle at C1 (P1-C1-C2 or P1-C1-N1) from **3b** to **3d** and from **4** to **5** observed in the experiment can also be seen in the calculated value s of the free anions. The bending angle in neutral divalent carbon (0) compounds $CL_2$ (carbones) was previously explained with the π-acceptor strength of ligand L. The dicarbonyl complex $C(CO)_2$ (carbon suboxide $C_3O_2$) has a larger bending angle of 156° than the dinitrogen complex $C(N_2)_2$ (122°) because CO is a better π-acceptor than $N_2$.[29] The same trend is observed for the anions $[C(CN)(CO)]^-$ (166°) and $[C(CN)(N_2)]^-$ (134°)[30] and for compounds **4** and **5**. The bending potential at C1 toward a linear coordination is not very deep, between 0.4 kcal for **4** and 5.5 kcal/mol for **5**. Although the bending potential is not very large, the significant deviation from a linear geometry at C1, which opposes steric repulsion between the ligands, is a structural feature that supports the description of the bonding situation as anionic carbones.

**3b**

$C_1$

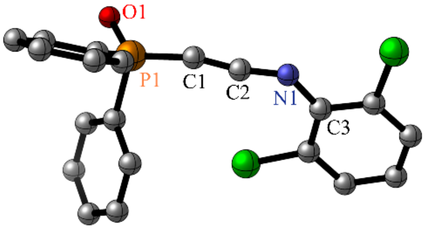


| | Bond/ angle | Calc. | Exper. |
|---|---|---|---|
| | P1-O1 | 1.507 | 1.490(2) |
| | P1-C1 | 1.695 | 1.701(2) |
| | C1-C2 | 1.254 | 1.227(3) |
| | C2-N1 | 1.275 | 1.280(3) |
| | N1-C3 | 1.350 | - |
| | P1-C1-C2 | 161.8 | 166.9(2) |
| | C1-C2-N1 | 168.7 | 168.1(2) |
| | C2-N1-C3 | 132.0 | - |
| **$D_e$** | | 122.1 (125.3) | |
| **ΔE** | | 0.7 | |

**3d**

$C_1$

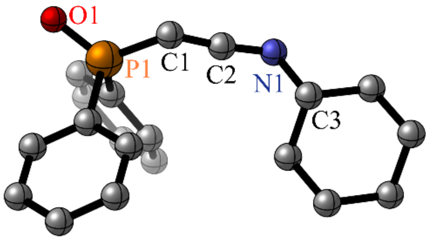


| | Bond/ angle | Calc. | Exper. |
|---|---|---|---|
| | P1-O1 | 1.508 | 1.502(3) |
| | P1-C1 | 1.705 | 1.698(5) |
| | C1-C2 | 1.278 | 1.273(7) |
| | C2-N1 | 1.262 | 1.249(6) |
| | N1-C3 | 1.459 | - |
| | P1-C1-C2 | 135.7 | 139.8(4) |
| | C1-C2-N1 | 174.4 | 173.1(5) |
| | C2-N1-C3 | 109.8 | - |
| **$D_e$** | | 104.5 (107.4) | |
| **ΔE** | | 1.4 | |

**4**

$C_1$

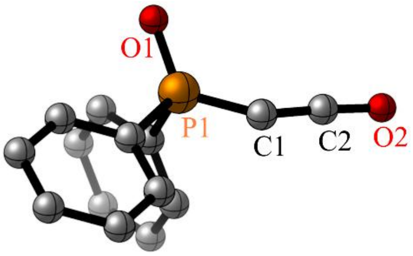


| | | |
|---|---|---|
| P1-O1 | 1.512 | 1.499(9) |
| P1-C1 | 1.686 | 1.684(1) |
| C1-C2 | 1.273 | 1.236(2) |
| C2-O2 | 1.205 | - |
| P1-C1-C2 | 146.5 | 152.8(1) |
| C1-C2-O2 | 175.2 | 176.6(1) |

| | |
|---|---|
| **$D_e$** | 105.8 (110.0) |
| **ΔE** | 0.4 |

**5**

$C_1$

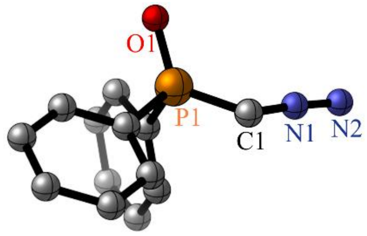


| | | |
|---|---|---|
| P1-O1 | 1.513 | 1.492(2) |
| P1-C1 | 1.716 | 1.710(3) |
| C1-N1 | 1.272 | - |
| N1-N2 | 1.166 | - |
| P1-C1-N1 | 124.6 | 125.9(2) |
| C1-N1-N2 | 173.7 | 170.3(2) |

| | |
|---|---|
| **$D_e$** | 54.2 (58.1) |
| **ΔE** | 5.5 |

**Figure S126**. Calculated and experimental bond lengths [Å] and angles [degree] of the anions **3b**, **3d**, **4** and **5.** The calculated values at BP86+D3(BJ)/def2-TZVPP come from the free anions and the experimental values come from the x-ray structure analysis of the complexes. Calculated bond dissociation energies $D_e$ to the fragments $[Ph_2OPC]^-$ + L2 in the electronic singlet states. The $D_e$ values in parentheses refer to the electronic ground states, which is a triplet state for $[Ph_2OPC]^-$. The ΔE values give the energy differences between the equilibrium geometries and the linear structures at C1 with P1-C1-C2 = 180.0.

Figure S55 shows also the calculated bond dissociation energies (BDE) for dissociation of the L2 ligands. The BDE values for the spin symmetry allow fragmentation into the electronic singlet states of $[Ph_2OPC]^-$ and L2 has the order **3b** > **4** > **3d** >> **5**. The anion $[Ph_2OPC]^-$ has a triplet ground state, which is energetically slightly lower than the singlet state. The BDE for dissociation into to the electronic ground states of $[Ph_2OPC]^-$ and L2 is therefore a bit higher than for the singlet species but is has the same order.

**Table S8.** Calculated Voronoi partial charges (q) and Mayer bond orders (MBO) of structures of the anions [C(POPh2)(L2)]- **3b** (L2 = CNAr), **3d** (L2 = CNCy), **4** (L2 = CO), and **5** (L2 = N2) at the BP86+D3(BJ)/def2-TZVPP level.

| Species | Charges q | | Bond orders MBO | |
|---|---|---|---|---|
| **3b** | C1 | -0.31 | C1- $POPh_2$ | 1.24 |
| | $POPh_2$ | -0.27 | C1-L2 | 2.20 |
| | CNAr | -0.42 | | |
| **3d** | C1 | -0.38 | C1- $POPh_2$ | 1.21 |
| | $POPh_2$ | -0.36 | C1-L2 | 1.85 |
| | CNCy | -0.26 | | |
| **4** | C1 | -0.39 | C1- $POPh_2$ | 1.26 |
| | $POPh_2$ | -0.38 | C1-L2 | 1.99 |
| | CO | -0.23 | | |
| **5** | C1 | -0.38 | C1- $POPh_2$ | 1.18 |
| | $POPh_2$ | -0.40 | C1-L2 | 1.62 |
| | $N_2$ | -0.22 | | |

Table S8 shows the calculated partial charges of the ligands $POPh_2$ and L2 and for the C1 atom as well as the Mayer bond orders (MBO) of the C1- $POPh_2$ and C1-L2 bonds of the four molecules. The negative charge of the anions is distributed over the three moieties. The CNAr ligand of **3b** has a significantly higher negative partial charge than the other L2 ligands in **3d**, **4** and **5**. It is surprising that $N_2$ carries in **4** nearly the same negative charge (-0.22 e) as CO in **5** (-0.23 e) although the latter ligand is much more strongly bonded than the former. This will be discussed below. The MBO values suggest a significantly higher multiple bond character for the C1-L2 bonds than for the C1- $POPh_2$ bonds.

**Table S9**. EDA-NOCV results of **3b**, **3d**, **4** and **5** **using** $[(POPh_2)C]^-$ + L2 as interacting fragments in the electronic singlet states. The calculations are performed at the BP86+D3(BJ)/TZ2P level using the BP86+D3(BJ)/def2-TZVPP optimized structures. Energy values are given in kcal/mol.

| Molecules | **3b** | **3d** | **4** | **5** |
|---|---|---|---|---|
| Fragments | $[(POPh_2)C]^-$ + CNAr | $[(POPh_2)C]^-$ + CNCy | $[(POPh_2)C]^-$ + CO | $[(POPh_2)C]^-$ + $N_2$ |
| $\Delta E_{int}$ | -253.4 | -239.7 | -225.6 | -177.3 |
| $\Delta E_{Pauli}$ | 454.0 | 565.0 | 508.1 | 698.3 |
| $\Delta E_{elstat}$ | -205.4(29.0%) | -270.5(33.6%) | -236.4(32.2%) | -298.9(34.1%) |
| $\Delta E_{disp}$ | -6.6(1.0%) | -6.9(0.9%) | -2.3(0.3%) | -3.9(0.4%) |

| | | | | |
|---|---|---|---|---|
| $\Delta E_{orb}$ | -495.3(70.0%) | -527.3(65.5%) | -495.0(67.5%) | -572.9(65.5%) |
| $\Delta E_{orb1}$ | -197.2(39.8%) | -302.6(57.4%) | -281.5(56.9%) | -381.7(66.6%) |
| $\Delta E_{orb2}$ | -156.2(31.5%) | -104.1(19.8%) | -90.3(18.2%) | -85.4(14.9%) |
| $\Delta E_{orb3}$ | -97.9(19.8%) | -85.9(16.3%) | -90.0(18.2%) | -70.2(12.3%) |
| $\Delta E_{orb(rest)}$ | -44.1(8.9%) | -34.6(6.5%) | -33.3(6.7%) | -35.7(6.2%) |

More detailed information about the chemical bonds is available from the EDA-NOCV analysis of the molecules. The focus is on the dative bonds of the L2 ligands. The numerical results using the fragments $[(POPh_2)C]^-$ + L2 as interacting moieties are shown in Table S9. The intrinsic interaction energies $\Delta E_{int}$ between the frozen fragments have the order **3b** > **3d** > **4** >> **5**, which is a bit different than the BDE values where **4** has a slightly higher $D_e$ value than **3d**. It indicates that the CNCy ligand of **3d** has a larger relaxation energy than CO in compound **4**, which appears reasonable in view of the different size of the ligands. The relevance of the relaxation energy of the fragments for the BDE values has been stressed by Bickelhaupt, who coined the term "activation stress" for the fragment relaxation.[31]

The breakdown of $\Delta E_{int}$ into the attractive components ($\Delta E_{orb}$, $\Delta E_{elstat}$, $\Delta E_{disp}$) and the Pauli repulsion $\Delta E_{Pauli}$ reveals the surprising result that the sum of the attractive forces in **5** between $[(POPh_2)C]^-$ and $N_2$ are even higher than in **3b** (L2 = CNAr) and **4** (L2 = CO). The significantly lower total attraction comes from the much higher Pauli repulsion in **5** than in the other molecules. It has been shown before that the Pauli repulsion between electrons with the same spin is a very important but often neglected energy term in chemical bonding. The bond lengths of chemical bonds does not come from the maximum overlap of the bonding orbitals, which is much shorter than the equilibrium distance, but from the strong influence of the Pauli repulsion on the electronic structure.[32]

Table S9 shows that the largest contribution to the bonding interactions between $[(POPh_2)C]^-$ and L2 comes from the covalent (orbital) term $\Delta E_{orb}$, provides 65 – 70% of the total attraction. There are three orbital pair interactions $\Delta E_{orb1-3}$, which give > 90 % of $\Delta E_{orb}$. The orbitals which are involved in the pairwise interactions can be identified with the shape of the deformation densities $\Delta\rho_{1-3}$ and the connected fragment orbitals, which are associated with the orbital interactions $\Delta E_{orb1-3}$. They are shown in Figure S56. It becomes obvious that the orbital interactions are dominated by the $[(POPh_2)C]^-\leftarrow$L2 σ donation and the in-plane and out-of-plane $[(POPh_2)C]^-\rightarrow$L2 π backdonation following the classical Dewar-Chatt-Duncanson model.[33,34] Note that the division of the orbital interactions into σ and π orbitals comes from the symmetry of the fragments, which is lost in the final molecules. This leads to a mixture of σ donation and π backdonation in some orbital terms. The $[(POPh_2)C]^-\leftarrow$L2 σ donation and the in-plane $[(POPh_2)C]^-\rightarrow$L2 π backdonation mix in the compounds, which have approximately $C_s$ symmetry. But the out-of-plane $[(POPh_2)C]^-\rightarrow$L2 π backdonation appears always as separate contribution ($\Delta E_{orb3}$ in **3b** and $\Delta E_{orb2}$ in **3d**, **4**, **5**) which indicate that the π-acceptor strength of the ligands L2 has the order CNCy > CNAr > CO > $N_2$.

| Deformation density $\Delta\rho$ | Orbital $[(POPh_2)C]^-$ | | Orbital L2 |
|---|---|---|---|
| **3b** | | | |
| 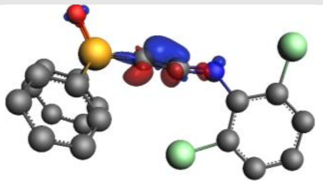 $\Delta E_{orb1}$ = -197.2 (kcal/mol) υ = ±1.110 | 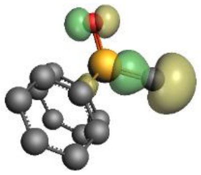 LUMO (υ = 0.612) | ← | 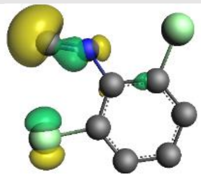 HOMO-2 (υ = -0.370) |
| 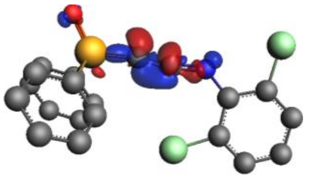 $\Delta E_{orb2}$ = -156.2 (kcal/mol) υ = ±0.933 | 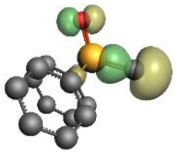 LUMO (υ = 0.573) | ← | 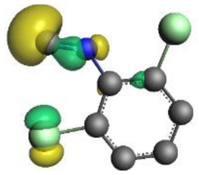 HOMO-2 (υ = -0.445) |
| | 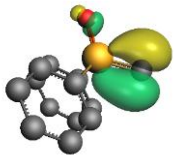 HOMO-1 (υ = -0.300) | → | 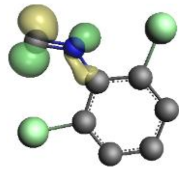 LUMO+1 (υ = 0.291) |
| 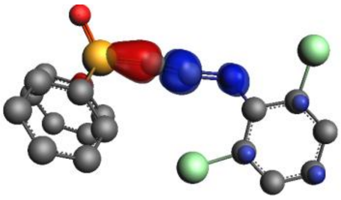 $\Delta E_{orb3}$ = -97.9 (kcal/mol) υ = ±1.085 | 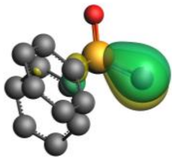 HOMO (υ = -0.778) | → | 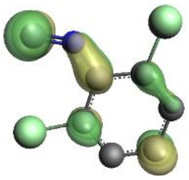 LUMO (υ = 0.633) |
| **3d** | | | |
| 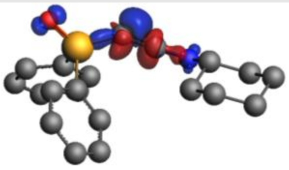 $\Delta E_{orb1}$ = -302.6 (kcal/mol) υ = ±1.292 | 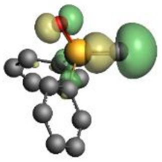 LUMO (υ = 1.138) | ← | 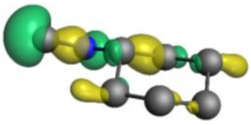 HOMO (υ = -0.373) |
| 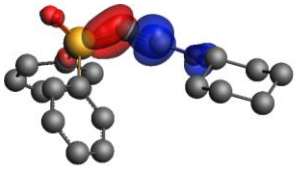 $\Delta E_{orb2}$ = -104.1 (kcal/mol) υ = ±1.040 | 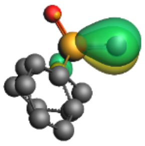 HOMO (υ = -0.695) | → | 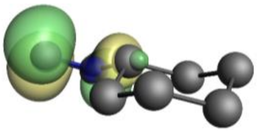 LUMO (υ = 0.730) |

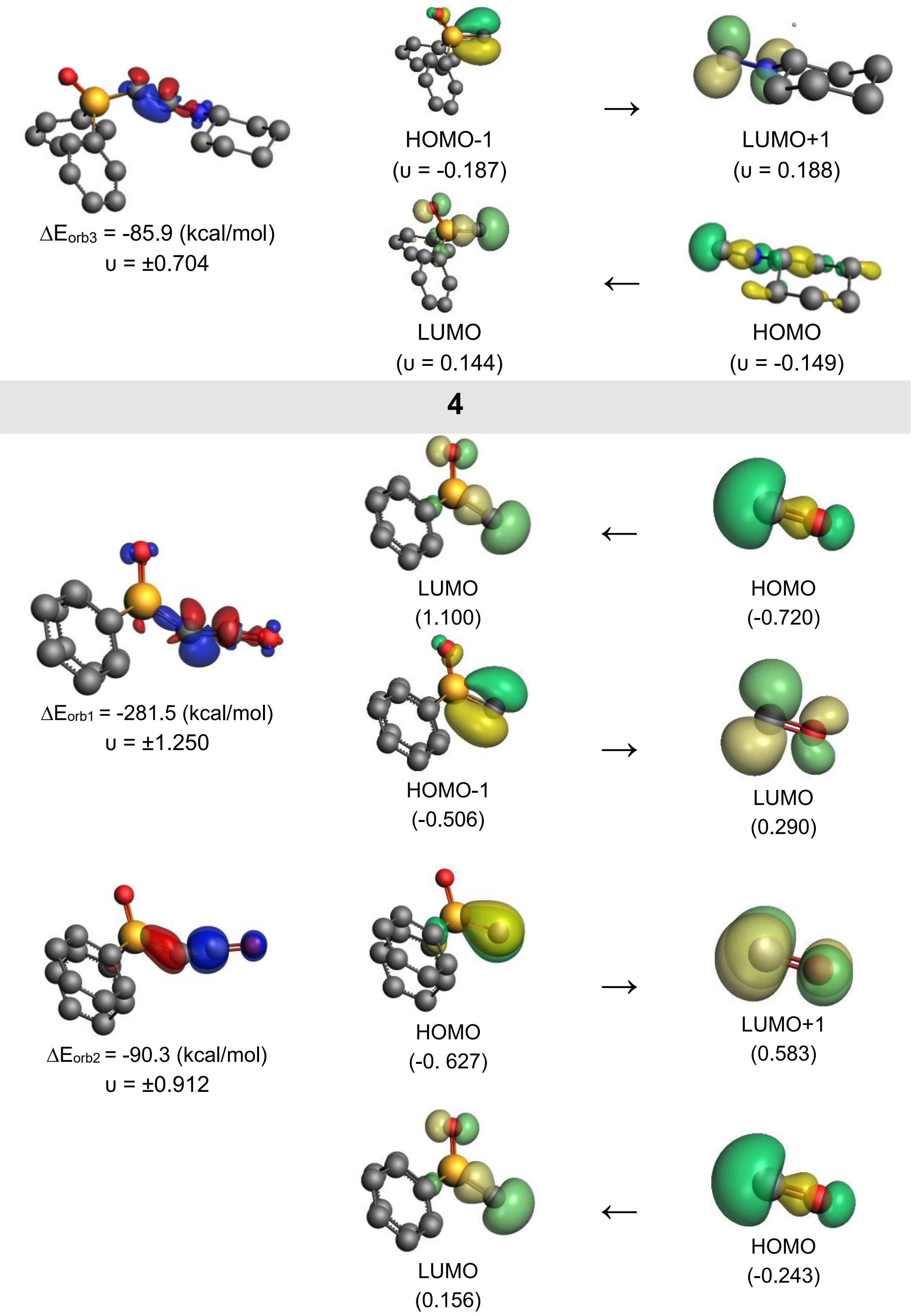
ΔEorb3 = -85.9 (kcal/mol)
υ = ±0.704
HOMO-1
(υ = -0.187)
LUMO+1
(υ = 0.188)
LUMO
(υ = 0.144)
HOMO
(υ = -0.149)
4
ΔEorb1 = -281.5 (kcal/mol)
υ = ±1.250
LUMO
(1.100)
HOMO
(-0.720)
HOMO-1
(-0.506)
LUMO
(0.290)
ΔEorb2 = -90.3 (kcal/mol)
υ = ±0.912
HOMO
(-0. 627)
LUMO+1
(0.583)
LUMO
(0.156)
HOMO
(-0.243)

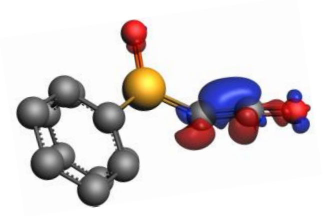

$\Delta E_{orb3}$ = -90.0 (kcal/mol)
υ = ±0.701

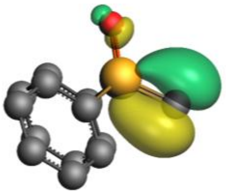

HOMO-1
(-0.177)

→

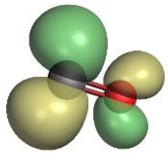

LUMO
(0.202)

## 5

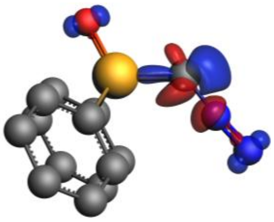

$\Delta E_{orb1}$ = -381.7 (kcal/mol)
υ = ±1.556

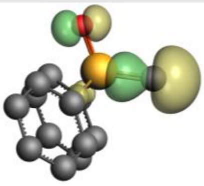

LUMO
(1.247)

←

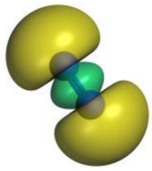

HOMO
(-0.342)

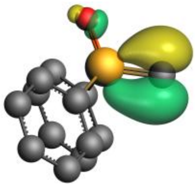

HOMO-1
(-0.903)

→

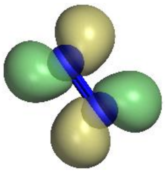

LUMO
(0.377)

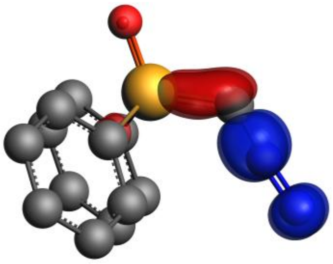

$\Delta E_{orb2}$ = -85.4 (kcal/mol)
υ = ±1.023

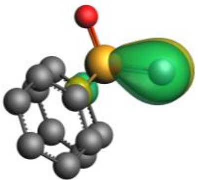

HOMO
(-0.695)

→

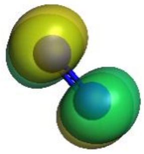

LUMO+1
(0.629)

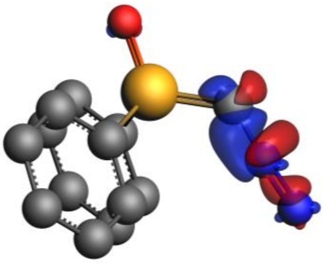

$\Delta E_{orb3}$ = -70.2 (kcal/mol)
υ = ±0.571

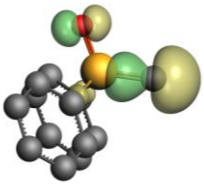

LUMO
(0.115)

←

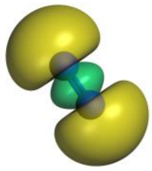

HOMO
(-0.169)

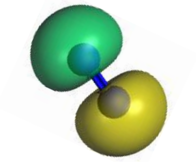

HOMO-3
(-0.133)

**Figure S127.** Deformation densities Δρ and the associated fragment orbitals of the orbital interactions $\Delta E_{orb1}$ - $\Delta E_{orb3}$ in **3b**, **3d**, **4** and **5**. The eigenvalues v indicate the amount of electronic charge which is altered.

## 4.3. Cartesian Coordinates

BP86+D3(BJ)/def2-TZVPP
E = -2162.33530337 Hartree
**3b** [Ph2OPCCNC6H3Cl2]

| | | | |
|---|---|---|---|
| Cl | 1.656796 | 1.894850 | -0.124466 |
| P | -1.927401 | -0.283067 | 1.166924 |
| O | -2.480305 | -0.170051 | 2.563727 |
| N | 2.097303 | -1.170009 | 0.424811 |
| C | -0.359300 | -0.855177 | 0.872871 |
| Cl | 4.561714 | -2.734616 | 0.286456 |
| C | 0.871138 | -0.888864 | 0.635103 |
| C | -2.036971 | 1.349231 | 0.325638 |
| C | -1.573841 | 1.543607 | -0.983233 |
| H | -1.117635 | 0.707987 | -1.516656 |
| C | -1.666700 | 2.799953 | -1.584197 |
| H | -1.293800 | 2.946101 | -2.599881 |
| C | -2.218001 | 3.876158 | -0.877805 |
| H | -2.281943 | 4.861046 | -1.345245 |
| C | -2.676688 | 3.688600 | 0.429764 |
| H | -3.099972 | 4.528373 | 0.985614 |
| C | -2.589371 | 2.427539 | 1.028564 |
| H | -2.934250 | 2.251815 | 2.049436 |
| C | -3.032766 | -1.318933 | 0.125056 |
| C | -2.534501 | -2.165449 | -0.873363 |
| H | -1.451882 | -2.230814 | -1.005053 |
| C | -3.409419 | -2.918364 | -1.662792 |
| H | -3.012794 | -3.579721 | -2.436221 |
| C | -4.790373 | -2.828762 | -1.459411 |
| H | -5.474272 | -3.416132 | -2.075922 |
| C | -5.293619 | -1.990243 | -0.457437 |
| H | -6.371148 | -1.925890 | -0.290093 |
| C | -4.418349 | -1.241090 | 0.332142 |
| H | -4.793880 | -0.597780 | 1.130231 |
| C | 3.154592 | -0.401066 | 0.090557 |
| C | 3.160832 | 1.004222 | -0.188458 |
| C | 4.315027 | 1.706609 | -0.527518 |
| H | 4.234046 | 2.776006 | -0.724217 |
| C | 5.546300 | 1.051738 | -0.614180 |
| H | 6.449092 | 1.603143 | -0.877402 |
| C | 5.601489 | -0.322787 | -0.358932 |
| H | 6.542411 | -0.869533 | -0.418546 |
| C | 4.444467 | -1.013939 | -0.021904 |

BP86+D3(BJ)/def2-TZVPP
E= -1246.61942100 Hartree
**3d** [Ph2OPCCNC6H11]-
Charge -1 Singlet

| | | | |
|---|---|---|---|
| P | 1.813161 | -0.103634 | 1.347948 |
| O | 3.060841 | -0.248253 | 2.183022 |
| N | -2.053815 | -0.979812 | 1.552546 |
| C | 0.275562 | -0.128143 | 2.085075 |
| C | -0.888602 | -0.541310 | 1.757985 |
| C | 1.860485 | -1.365644 | 0.003482 |
| C | 2.832999 | -2.374375 | 0.056035 |
| H | 3.555879 | -2.343171 | 0.873926 |
| C | 2.864952 | -3.380267 | -0.915255 |
| H | 3.624008 | -4.164898 | -0.863487 |
| C | 1.930626 | -3.381053 | -1.956429 |
| H | 1.955905 | -4.164372 | -2.717281 |
| C | 0.959614 | -2.372891 | -2.017371 |
| H | 0.225235 | -2.369704 | -2.826136 |
| C | 0.923274 | -1.376192 | -1.041111 |
| H | 0.157935 | -0.600111 | -1.080121 |
| C | 1.882093 | 1.457003 | 0.364345 |
| C | 0.760442 | 2.273699 | 0.178431 |
| H | -0.171878 | 1.985679 | 0.669720 |
| C | 0.845927 | 3.432289 | -0.600806 |
| H | -0.035839 | 4.062338 | -0.739313 |
| C | 2.058789 | 3.783860 | -1.202307 |
| H | 2.127006 | 4.686397 | -1.813907 |
| C | 3.187731 | 2.977194 | -1.012463 |
| H | 4.138562 | 3.252344 | -1.475133 |
| C | 3.099447 | 1.823108 | -0.230279 |
| H | 3.975382 | 1.193442 | -0.061363 |
| C | -3.032533 | -0.145701 | 0.864359 |
| C | -2.637957 | 0.141239 | -0.596607 |
| C | -4.406491 | -0.826003 | 0.912916 |
| H | -3.124129 | 0.837932 | 1.379158 |
| C | -3.703796 | 0.959691 | -1.335784 |
| H | -2.484911 | -0.828913 | -1.100992 |
| H | -1.669008 | 0.663228 | -0.609240 |
| C | -5.481082 | -0.012431 | 0.180749 |
| H | -4.303522 | -1.823217 | 0.449968 |
| H | -4.683500 | -0.996434 | 1.964699 |
| C | -5.075439 | 0.273515 | -1.272338 |
| H | -3.403948 | 1.125344 | -2.383715 |
| H | -3.780457 | 1.959463 | -0.871578 |
| H | -6.449753 | -0.538442 | 0.211825 |
| H | -5.630949 | 0.948835 | 0.705205 |
| H | -5.840791 | 0.889449 | -1.773643 |
| H | -5.024907 | -0.681496 | -1.825586 |

BP86+D3(BJ)/def2-TZVPP
E= -1031.75789026 Hartree
**4** [Ph2OPCCO]-
Charge -1 Singlet

| | | | |
|---|---|---|---|
| P | -0.072948 | 0.754365 | 0.836443 |
| O | -0.088587 | 0.639929 | 2.343565 |
| C | -0.307712 | 2.194415 | -0.008701 |
| C | -1.070822 | 3.192648 | -0.215787 |
| C | -1.257541 | -0.492132 | 0.151702 |
| C | -1.658640 | -0.477682 | -1.191692 |

| | | | |
|---|---|---|---|
| H | -1.277452 | 0.313388 | -1.841061 |
| C | -2.547503 | -1.440070 | -1.676742 |
| H | -2.856178 | -1.419343 | -2.724717 |
| C | -3.052462 | -2.425495 | -0.818917 |
| H | -3.752068 | -3.174794 | -1.196478 |
| C | -2.665014 | -2.439428 | 0.525165 |
| H | -3.065653 | -3.199847 | 1.200259 |
| C | -1.769277 | -1.478651 | 1.006246 |
| H | -1.460874 | -1.457638 | 2.053626 |
| C | 1.519593 | 0.071911 | 0.201263 |
| C | 2.126195 | 0.568342 | -0.959575 |
| H | 1.640080 | 1.403819 | -1.470248 |
| C | 3.324833 | 0.018414 | -1.424930 |
| H | 3.792945 | 0.415104 | -2.329199 |
| C | 3.928967 | -1.036089 | -0.731036 |
| H | 4.865458 | -1.466624 | -1.093255 |
| C | 3.332661 | -1.531601 | 0.434791 |
| H | 3.807059 | -2.347638 | 0.985407 |
| C | 2.135820 | -0.978435 | 0.897889 |
| H | 1.669195 | -1.337787 | 1.817310 |
| O | -1.724274 | 4.169945 | -0.479359 |

BP86+D3(BJ)/def2-TZVPP
E= -1027.89025948 Hartree
**5** [Ph2OPCNN]-
Charge -1 Singlet

| | | | |
|---|---|---|---|
| P | -0.040467 | 0.849739 | 0.734166 |
| O | -0.114345 | 0.877171 | 2.245011 |
| C | -0.214844 | 2.227798 | -0.273466 |
| C | -1.215897 | -0.448181 | 0.136181 |
| C | -1.397907 | -0.709303 | -1.230518 |
| H | -0.821251 | -0.133508 | -1.957569 |
| C | -2.313449 | -1.675314 | -1.652758 |
| H | -2.447409 | -1.871475 | -2.719259 |
| C | -3.068565 | -2.386419 | -0.710855 |
| H | -3.789966 | -3.137311 | -1.041075 |
| C | -2.899578 | -2.125025 | 0.652747 |
| H | -3.491378 | -2.673161 | 1.390232 |
| C | -1.974449 | -1.163638 | 1.073426 |
| H | -1.821425 | -0.937264 | 2.130725 |
| C | 1.571421 | 0.147243 | 0.200283 |
| C | 2.251982 | 0.602259 | -0.936661 |
| H | 1.801554 | 1.423649 | -1.499882 |
| C | 3.470432 | 0.026156 | -1.310253 |
| H | 3.996847 | 0.390382 | -2.195890 |
| C | 4.017250 | -1.015488 | -0.552156 |
| H | 4.967067 | -1.468485 | -0.845798 |
| C | 3.344810 | -1.470811 | 0.588280 |
| H | 3.772305 | -2.278036 | 1.187857 |
| C | 2.131197 | -0.889077 | 0.963575 |
| H | 1.606261 | -1.217628 | 1.862844 |
| N | -1.287423 | 2.900043 | -0.397899 |
| N | -2.207612 | 3.588269 | -0.598058 |

BP86+D3(BJ)/def2-TZVPP
E= -2162.88145474 Hartree
**3b-H$_c$** Ph2OP-CH=C=NAr
Charge 0 Singlet

| | | | |
|---|---|---|---|
| Cl | 0.616960 | -2.787257 | 0.741697 |
| P | -1.890189 | 1.197464 | 0.800153 |
| O | -2.388451 | 2.527247 | 1.269907 |
| N | 1.453699 | 0.067077 | 1.629986 |
| C | -1.041129 | 0.162073 | 2.024302 |
| Cl | 3.793048 | 1.638209 | 0.876481 |
| C | 0.252826 | -0.006546 | 1.851377 |
| C | -0.636209 | 1.310742 | -0.520675 |
| C | -0.324126 | 0.231652 | -1.361945 |
| H | -0.901923 | -0.691253 | -1.304211 |
| C | 0.734632 | 0.332868 | -2.265296 |
| H | 0.980609 | -0.511839 | -2.909847 |
| C | 1.485673 | 1.510787 | -2.333686 |
| H | 2.321818 | 1.583561 | -3.030330 |
| C | 1.165755 | 2.593499 | -1.509767 |
| H | 1.750908 | 3.512133 | -1.563181 |
| C | 0.104646 | 2.497724 | -0.606130 |
| H | -0.153835 | 3.329725 | 0.050292 |
| C | -3.215501 | 0.119935 | 0.174025 |
| C | -3.094197 | -1.276985 | 0.119617 |
| H | -2.190577 | -1.756504 | 0.499284 |
| C | -4.131765 | -2.048508 | -0.408764 |
| H | -4.035933 | -3.134547 | -0.448230 |
| C | -5.293738 | -1.428985 | -0.879543 |
| H | -6.104800 | -2.032773 | -1.289224 |
| C | -5.421260 | -0.037441 | -0.817025 |
| H | -6.332236 | 0.444308 | -1.174904 |
| C | -4.386229 | 0.738121 | -0.290894 |
| H | -4.474159 | 1.823059 | -0.219573 |
| C | 2.273509 | -0.611751 | 0.736629 |
| C | 2.020277 | -1.901598 | 0.219356 |
| C | 2.873619 | -2.497444 | -0.707816 |
| H | 2.641874 | -3.493566 | -1.081248 |
| C | 4.002931 | -1.805869 | -1.147165 |
| H | 4.667517 | -2.266251 | -1.878188 |
| C | 4.289323 | -0.533234 | -0.654042 |
| H | 5.166719 | 0.017108 | -0.988888 |
| C | 3.438361 | 0.048795 | 0.284166 |
| H | -1.605982 | -0.325809 | 2.817426 |

BP86+D3(BJ)/def2-TZVPP
E= -2162.85033636 Hartree
**3b-$H_N$** Ph2OP-C≡C-NHAr
Charge 0 Singlet

| | | | |
|---|---|---|---|
| Cl | -1.397647 | 1.496977 | -0.859844 |
| P | 2.004933 | -0.294142 | 1.184113 |
| O | 2.367193 | -0.349384 | 2.632932 |
| N | -2.001522 | -1.415123 | 0.128656 |
| C | 0.428665 | -0.906412 | 0.729205 |
| Cl | -4.587406 | -2.717456 | 0.550367 |
| C | -0.743759 | -1.099383 | 0.438537 |
| C | 3.171471 | -1.166399 | 0.099595 |
| C | 2.783471 | -1.757880 | -1.111066 |
| H | 1.732041 | -1.742817 | -1.403250 |
| C | 3.737341 | -2.375337 | -1.923902 |
| H | 3.433151 | -2.838754 | -2.863575 |
| C | 5.078246 | -2.405986 | -1.528762 |
| H | 5.821898 | -2.890692 | -2.163057 |
| C | 5.465931 | -1.825167 | -0.316463 |

H 6.510447 -1.859165 -0.003663
C 4.516279 -1.206726 0.498605
H 4.799851 -0.764400 1.454718
C 1.873292 1.404658 0.537999
C 1.959611 1.701515 -0.829900
H 2.169451 0.907771 -1.548120
C 1.778247 3.014000 -1.270605
H 1.847055 3.244102 -2.334739
C 1.507462 4.030944 -0.348490
H 1.362065 5.055053 -0.695218
C 1.429585 3.737109 1.016282
H 1.225298 4.531460 1.735583
C 1.613405 2.426333 1.462224
H 1.563102 2.177546 2.523215
C -3.074744 -0.546900 -0.110098
C -2.955381 0.793310 -0.537483
C -4.087899 1.583930 -0.744188
H -3.946859 2.614472 -1.066563
C -5.365231 1.058245 -0.559584
H -6.242419 1.681769 -0.727996
C -5.517944 -0.272307 -0.167793
H -6.503360 -0.712037 -0.023978
C -4.386291 -1.049251 0.052906
H -2.297249 -2.372882 0.329908

BP86+D3(BJ)/def2-TZVPP
E= -1247.18792132 Hartree
**3d-H$_c$** Ph2OP-CH=C=NCy
Charge 0 Singlet
P 1.579691 -0.206149 1.161744
O 1.859325 0.160147 2.586760
N -1.605545 -1.553202 -0.339746
C 0.585627 -1.688077 0.929203
C -0.586263 -1.637119 0.318212
C 3.097305 -0.492631 0.198436
C 4.260799 0.181303 0.600770
H 4.227492 0.805852 1.494573
C 5.440985 0.031680 -0.129660
H 6.345167 0.553887 0.186485
C 5.466401 -0.790511 -1.261335
H 6.390365 -0.907447 -1.829574
C 4.311605 -1.471165 -1.659010
H 4.334206 -2.122372 -2.534054
C 3.128768 -1.324938 -0.929946
H 2.229395 -1.868777 -1.224420
C 0.670804 1.096732 0.266507
C 0.115793 2.128844 1.036699
H 0.259511 2.109369 2.118151
C -0.593470 3.160090 0.414777
H -1.022976 3.963194 1.015375
{Grimme, 2006 #7} C -0.748781 3.163618 -0.974705
H -1.301665 3.969320 -1.459880
C -0.192818 2.136333 -1.744834
H -0.311809 2.140031 -2.829136
C 0.517837 1.106025 -1.127583
H 0.957461 0.311501 -1.731758
C -2.917481 -1.101700 0.157780
C -3.143933 0.338046 -0.322143
C -4.014870 -2.038426 -0.355843

| | | | |
|---|---|---|---|
| H | -2.914454 | -1.111936 | 1.263204 |
| C | -4.531293 | 0.840569 | 0.093980 |
| H | -3.045728 | 0.356438 | -1.420004 |
| H | -2.353375 | 0.986489 | 0.079598 |
| C | -5.402310 | -1.534183 | 0.063200 |
| H | -3.942568 | -2.081928 | -1.455407 |
| H | -3.838507 | -3.058044 | 0.018179 |
| C | -5.640853 | -0.093068 | -0.406755 |
| H | -4.681954 | 1.862020 | -0.286573 |
| H | -4.579986 | 0.907390 | 1.194769 |
| H | -6.177638 | -2.204936 | -0.336362 |
| H | -5.489796 | -1.576597 | 1.162917 |
| H | -6.624716 | 0.262229 | -0.064531 |
| H | -5.662997 | -0.070818 | -1.510265 |
| H | 0.930310 | -2.609916 | 1.400198 |

BP86+D3(BJ)/def2-TZVPP
E= -1247.15615946 Hartree
**3d-H$_N$** Ph2OP-C≡C-NHCy
Charge 0 Singlet

| | | | |
|---|---|---|---|
| P | 1.652184 | -0.034675 | 1.201897 |
| O | 2.259784 | 0.208278 | 2.546958 |
| N | -2.233887 | -1.820667 | 0.933784 |
| C | 0.130076 | -0.877140 | 1.106834 |
| C | -0.984744 | -1.378681 | 1.006674 |
| C | 2.750647 | -0.965695 | 0.086886 |
| C | 4.130853 | -0.861891 | 0.316359 |
| H | 4.481458 | -0.281891 | 1.171280 |
| C | 5.029805 | -1.511562 | -0.531935 |
| H | 6.102528 | -1.432062 | -0.349732 |
| C | 4.556323 | -2.267930 | -1.608997 |
| H | 5.260092 | -2.776548 | -2.269607 |
| C | 3.180578 | -2.381482 | -1.833576 |
| H | 2.810520 | -2.982092 | -2.666005 |
| C | 2.278557 | -1.733071 | -0.987097 |
| H | 1.203314 | -1.833069 | -1.143991 |
| C | 1.266613 | 1.524681 | 0.332702 |
| C | 0.678952 | 1.551297 | -0.940470 |
| H | 0.433685 | 0.615080 | -1.444604 |
| C | 0.409582 | 2.771971 | -1.559937 |
| H | -0.045430 | 2.791299 | -2.551511 |
| C | 0.724482 | 3.971208 | -0.909083 |
| H | 0.512060 | 4.925352 | -1.393800 |
| C | 1.311277 | 3.946413 | 0.358999 |
| H | 1.556320 | 4.880780 | 0.866277 |
| C | 1.584914 | 2.724433 | 0.981243 |
| H | 2.040050 | 2.677697 | 1.971764 |
| C | -3.316365 | -0.873831 | 0.574903 |
| C | -3.101867 | -0.233370 | -0.802725 |
| C | -4.662600 | -1.593556 | 0.655151 |
| H | -3.290160 | -0.079071 | 1.339781 |
| C | -4.249219 | 0.724926 | -1.145498 |
| H | -3.046351 | -1.037128 | -1.558424 |
| H | -2.134029 | 0.290309 | -0.812882 |
| C | -5.815072 | -0.643290 | 0.300712 |
| H | -4.654555 | -2.439344 | -0.057521 |
| H | -4.798647 | -2.018054 | 1.661194 |
| C | -5.606662 | 0.013532 | -1.070524 |
| H | -4.092905 | 1.156994 | -2.145065 |

| | | | |
|---|---|---|---|
| H | -4.239089 | 1.570277 | -0.435970 |
| H | -6.769347 | -1.190091 | 0.326618 |
| H | -5.883557 | 0.141513 | 1.073406 |
| H | -6.422994 | 0.721314 | -1.279214 |
| H | -5.653768 | -0.761524 | -1.855480 |
| H | -2.369789 | -2.782937 | 0.629139 |

## 5. References


1 a) M. Jorges, S. Mondal, M. Kumar, P. Duari, F. Krischer, J. Loffler, V. H. Gessner, *Organometallics*. **2024**, *43*, 585−593. b) M. Jorges, F. Krischer, V. H. Gessner, *Science*. **2022**, *378*, 1331-1336. c) R. J. Ward, M. Jörges, H. Remm, E. Kiliani, F. Krischer, Q. Le Dé, V. H. Gessner, *J. Am. Chem. Soc.* **2024**, *146,* 24602-24608

2 G. M. Sheldrick, *Acta Cryst. A* **2008**, *64*, 112

3 G. M. Sheldrick, *Acta Cryst. C* **2015**, *71*, 3.

4 G. M. Sheldrick, *Acta Cryst. A* **2015**, *71*, 3

5 A. Thorn, B. Dittrich, G. M. Sheldrick, *Acta Cryst. A* **2012**, *68*, 448

6 A. D. Becke, *Phys Rev A Gen Phys*. **1988**, *38***,** 3098-3100.

7 J. P. Perdew, *Phys Rev B Condens Matter*. **1986**, *33***,** 8822-8824.

8 S. Grimme, *J. Comput. Chem.* **2006**, *27*, 1787.

9 S. Grimme, S. Ehrlich, L. Goerigk, *J. Comput. Chem.* **2011**, *32*, 1456.

10 S. Grimme, J. Antony, S. Ehrlich, H. Krieg, *J. Chem. Phys.* **2010**, *132*, 154104.

11 A. Schäfer, C. Huber, R. Ahlrichs, *J. Chem. Phys.* **1994**, *100*, 5829.

12 M. J. Frisch, G. W. Trucks, H. B. Schlegel, G. E. Scuseria, M. A. Robb, J. R. Cheeseman, G. Scalmani, V. Barone, G. A. Petersson, H. Nakatsuji, X. Li, M. Caricato, A. Marenich, J. Bloino, B. G. Janesko, R. Gomperts, B. Mennucci, H. P. Hratchian, J. V. Ortiz, A. F. Izmaylov, J. L. Sonnenberg, D. Williams-Young, F. Ding, F. Lipparini, F. Egidi, J. Goings, B. Peng, A. Petrone, T. Henderson, D. Ranasinghe, V. G. Zakrzewski, J. Gao, N. Rega, G. Zheng, W. Liang, M. Hada, M. Ehara, K. Toyota, R. Fukuda, J. Hasegawa, M. Ishida, T. Nakajima, Y. Honda, O. Kitao, H. Nakai, T. Vreven, K. Throssell, J. A. Montgomery, Jr., J. E. Peralta, F. Ogliaro, M. Bearpark, J. J. Heyd, E. Brothers, K. N. Kudin, V. N. Staroverov, T. Keith, R. Kobayashi, J. Normand, K. Raghavachari, A. Rendell, J. C. Burant, S. S. Iyengar, J. Tomasi, M. Cossi, J. M. Millam, M. Klene, C. Adamo, R. Cammi, J. W. Ochterski, R. L. Martin, K. Morokuma, O. Farkas, J. B. Foresman, and D. J. Fox, Gaussian 16 Rev. C.01, Wallingford, CT, 2016.

13 C. Fonseca Guerra, J.-W. Handgraaf, E. J. Baerends, F. M. Bickelhaupt, *J. Comput. Chem.* **2004**, *25*, 189.

14 I. Mayer, *Chem. Phys. Lett.* **1983**, *97*, 270.

15 I. Mayer, *Int. J. Quantum. Chem.* **1984**, *26*, 151.

16 T. Lu, F. Chen, *J. Comput. Chem.* **2012**, *33*, 580.

17 T. Ziegler, A. Rauk, *Theoret. Chim. Acta* **1977**, *46*, 1.

18 L. Zhao, M. von Hopffgarten, D. M. Andrada, G. Frenking, *WIREs Comput. Mol. Sci.* **2018**, *8*, e1345.

19 M. Mitoraj, A. Michalak, *Organometallics* **2007**, *26*, 6576.

20 M. Mitoraj, A. Michalak, *J. Mol. Model.* **2008**, *14*, 681.

21 G. te Velde, F. M. Bickelhaupt, E. J. Baerends, C. Fonseca Guerra, S. J. A. van Gisbergen, J. G. Snijders, T. Ziegler, *J. Comput. Chem.* **2001**, *22*, 931.

22 Amsterdam S., Density Functional 2022 (ADF2022), Theoretical Chemistry, Vrije Universiteit, Amsterdam, Netherlands, http://www.scm.com.

23 E. Van Lenthe, E. J. Baerends, *J. Comput. Chem.* **2003**, *24*, 1142.

24 F. M. Bickelhaupt, N. M. M. Nibbering, E. M. van Wezenbeek, E. J. Baerends, *J. Phys. Chem.* **1992**, *96*, 4864.

25 A. Krapp, F. M. Bickelhaupt, G. Frenking, *Chem. Eur. J.* **2006**, *12*, 9196.

26 L. Zhao, M. Hermann, W. H. E. Schwarz, G. Frenking, *Nat. Rev. Chem.* **2019**, *3*, 48.

27 L. Zhao, M. Hermann, N. Holzmann, G. Frenking, *Coord. Chem. Rev.* **2017**, *344*, 163.

28 L. Zhao, S. Pan, N. Holzmann, P. Schwerdtfeger, G. Frenking, *Chem. Rev.* **2019**, *119*, 8781.

29 G. Frenking, *Angew. Chem. Int. Ed.* **2014**, *53*, 6040-6046.

30 Q. Le Dé, Y. Zhang, L. Zhao, F. Krischer, K.-S. Feichtner, G. Frenking, V. H. Gessner, *Angew. Chem. Int. Ed.* **2025**, e202422496

31 P. Vermeeren, S. C. C. van der Lubbe, C. Fonseca Guerra, F. M. Bickelhaupt, T. A. Hamlin, *Nat. Protoc*. **2020**,*15*, 649-667.

32 A. Krapp, F. M. Bickelhaupt, G. Frenking, *Chem. Eur. J*. **2006**, *12*, 9196-9216.

33 M. J. S. Dewar, *Bull. Soc. Chim. Fr.* **1951**, *18*, C79

34 J. Chatt, L. A. Duncanson, *J. Chem. Soc*. **1953**, 2939-2947.
